# HOMINID: a framework for identifying associations between host genetic variation and microbiome composition

**DOI:** 10.1093/gigascience/gix107

**Published:** 2017-11-08

**Authors:** Joshua Lynch, Karen Tang, Sambhawa Priya, Joanna Sands, Margaret Sands, Evan Tang, Sayan Mukherjee, Dan Knights, Ran Blekhman

**Affiliations:** Department of Genetics, Cell Biology, and Development, University of Minnesota, 321 Church St SE, 6-160 Jackson Hall, Minneapolis MN 55455, USA; Department of Ecology, Evolution, and Behavior, University of Minnesota, 1479 Gortner Ave, 140 Gortner Lab, Saint Paul MN 55108, USA; Departments of Statistical Science, Mathematics, and Computer Science, Duke University, 112 Old Chemistry, Box 90251, Durham NC 27708, USA; Department of Computer Science and Engineering, University of Minnesota, 200 Union St SE, 4-192 Keller Hall, Minneapolis MN 55455, USA; Biotechnology Institute, University of Minnesota, 1479 Gortner Ave, 140 Gortner Lab, Saint Paul MN 55108, USA

**Keywords:** microbiome, host genetics, association, machine learning

## Abstract

Recent studies have uncovered a strong effect of host genetic variation on the composition of host-associated microbiota. Here, we present HOMINID, a computational approach based on Lasso linear regression, that given host genetic variation and microbiome taxonomic composition data, identifies host single nucleotide polymorphisms (SNPs) that are correlated with microbial taxa abundances. Using simulated data, we show that HOMINID has accuracy in identifying associated SNPs and performs better compared with existing methods. We also show that HOMINID can accurately identify the microbial taxa that are correlated with associated SNPs. Lastly, by using HOMINID on real data of human genetic variation and microbiome composition, we identified 13 human SNPs in which genetic variation is correlated with microbiome taxonomic composition across body sites. In conclusion, HOMINID is a powerful method to detect host genetic variants linked to microbiome composition and can facilitate discovery of mechanisms controlling host-microbiome interactions.

## Background

The microbial communities found in and on the human body are influenced by multiple factors [1]. In addition to the clear effect of environmental factors on the microbiome, there is growing support for an impact of host genetics [2, 3]. Several candidate gene studies have found correlation between human genetic variation and the structure of the microbiome [4–6]. In addition, genome-wide approaches can also be useful to identify human genetic impact on the microbiome [7–10]. For example, Goodrich et al. used hundreds of twin pairs to calculate the heritability of the gut microbiome and identify bacterial taxa that are heritable, such as Christensenellaceae [8]. Researchers have also utilized quantitative trait locus (QTL)–mapping approaches in the laboratory mouse and have identified multiple loci associated with the structure of gut microbial communities, some of which overlap with genes involved in immune response [11, 12]. Moreover, studies have used joint human genetic variation and microbiome data to find associations between loci in the human genome and microbial taxa [7, 10, 13, 14]. In our recent study, in addition to showing that human genetic variation is associated with the structure of microbial communities across 10 body sites, we identified human single nucleotide polymorphisms (SNPs) associated with variation in the microbiome and found that these loci are highly enriched in immunity genes and pathways [7]. This approach, which includes the joint analysis of host genetic variation (SNPs) and microbiome taxonomic composition data (usually an operational taxonomic unit (OTU) table), has the important advantage of identifying specific host genes and pathways that may control the microbiome, thus shedding light on the biological mechanisms of the host-microbiome interaction and pinpointing potential disease-causing pathways. However, this analysis is complicated by the fact that the microbiome contains many taxa that can be used as potential molecular complex traits in the genome-wide association study (GWAS) analysis. Testing many taxa reduces the power, and multiple hypothesis testing correction makes the identification of associations challenging.

**Figure 1:**
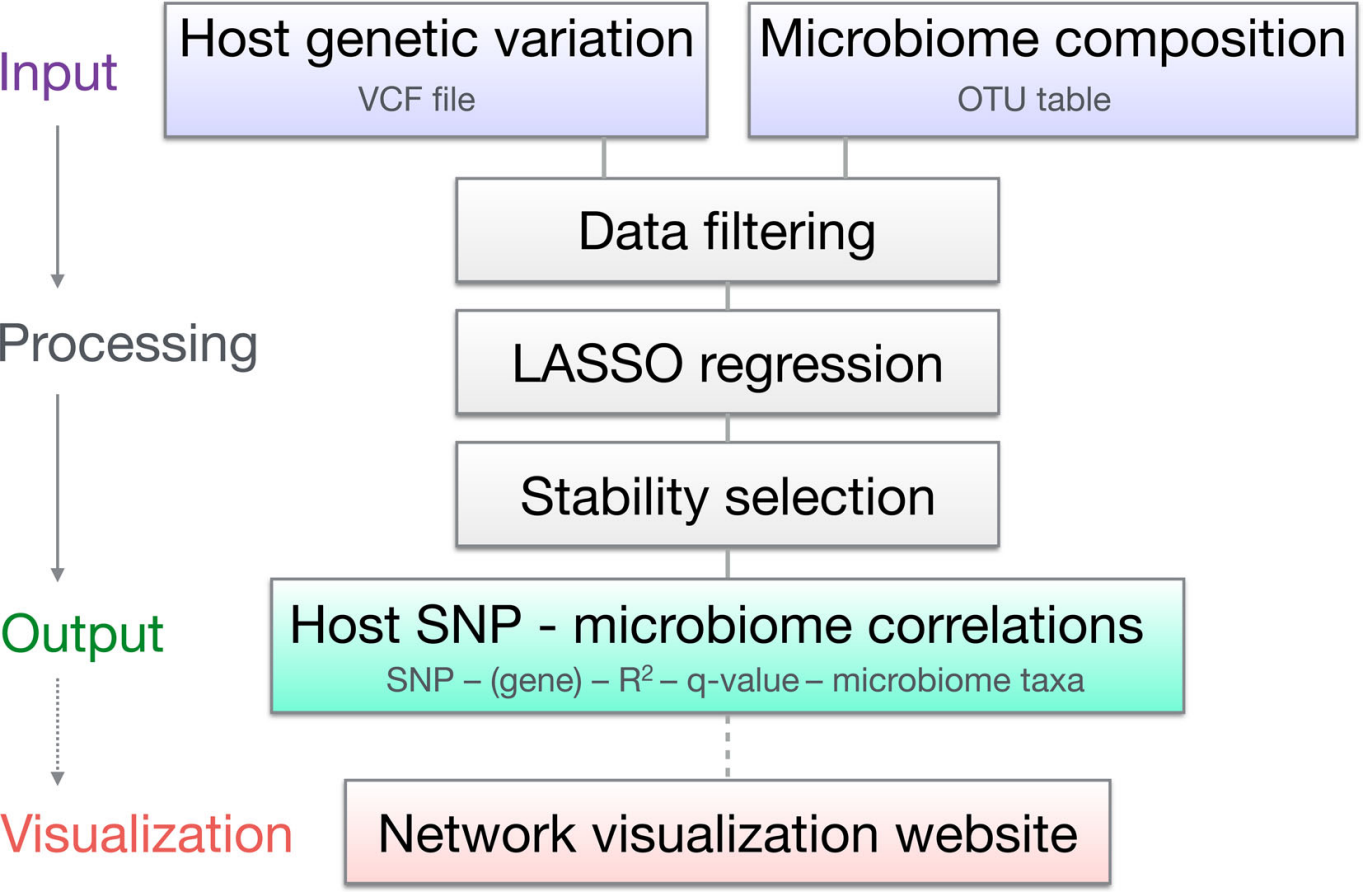
Illustration of the HOMINID pipeline.

Here, we propose a framework for identifying host SNPs associated with microbiome composition using Lasso regression, named Host-Microbiome Interaction Identification (HOMINID) (Fig. 1 and Supplementary Data). Our method has several advantages: (i) it takes as input host genetic variation data (in a modified variant call format (VCF) format) and microbiome taxonomic composition data (relative abundance data as an OTU table) to facilitate a simple analysis pipeline with no need to make new data formats; (ii) HOMINID uses Lasso regression, which is specifically designed for cases where a relatively small number of taxa are correlated with host SNP genotype, as opposed to existing methods that use all taxa abundances; and (iii) HOMINID uses stability selection with randomized Lasso to identify the specific microbial taxa that are correlated with each associated SNP.

## Materials and Methods

### HOMINID implementation

We implemented Lasso regression with the taxon relative abundances (arcsin sqrt transformed) as predictors and genetic variation at each SNP as response for the purpose of identifying an additive effect between host genotype and microbiome features (see Supplementary Data and Figs S1–S3). In most situations, we expect at most a few taxa's abundances to correlate with an SNP; therefore, ordinary least-squares (OLS) regression, which includes all taxa abundances as predictor variables, might not be an appropriate model. Instead, we need a regression algorithm that selects only the few predictors (taxa) that correlate to host genetics and discards the rest. The Lasso linear regression model used for HOMINID is similar to OLS regression, except that it includes an additional penalty term that shrinks most regression coefficients to zero, resulting in a sparse solution; thus it predicts only a few taxa to correlate with the host genetics. The Lasso regression was implemented using the Python (version 2.7/3.5+) machine-learning library scikit-learn [15], with microbiome relative abundances as predictors and SNP genotype as response variable. The penalty term was tuned via a 5-fold cross-validation. How well the host genetics correlates with the microbiome is measured with the coefficient of determination, $R_L^2$, calculated via a nested cross-validation procedure; $R_L^2$ is the median *R*^2^ from 5-fold cross-validation, with 100-times resampling. Also outputted are 95th percentile bootstrap confidence intervals from 10 000 bootstrap samples. Detailed description of the implementation of Lasso regression is available in the Supplementary Data.

### Identifying correlated SNPs and taxa

Identification of the SNPs that are predicted to be correlated to the microbiome (prediction positive) from the uncorrelated (prediction negative) HOMINID uses a *q*-value cutoff, which puts an upper bound on the false discovery rate (FDR). A cutoff value, $R_C^2$, of ${\rm{\ }}R_L^2$ is chosen such that the *q*-value, $q( {R_C^2} )$, is equal to 0.1. A given SNP is predicted positive (predicted correlated to the microbiome) if $R_L^2 \ge R_C^2$. $q( {R_C^2} )$ is determined by a permutation test, whereby for each SNP the sample labels are shuffled and Lasso regression is rerun 10 times. $q( {R_C^2} )$ is defined as the fraction of permuted SNPs predicted positive divided by the fraction of unpermuted SNPs predicted positive [16]. $R_C^2$ is chosen such that $q( {R_C^2} )$ = 0.1. The taxa that are most strongly associated with an SNP are identified using stability selection with randomized Lasso [17]. Briefly, stability selection perturbs the regression coefficients and the penalty term in the Lasso regression, and then reruns the regression thousands of times. If the same predictors (taxa) are repeatedly selected, even when the odds are against them, they are robust predictors. Full details on this procedure are available in the Supplementary Data.

### Controlling for other (non-taxon) covariates

HOMINID allows for controlling for any additional covariates (other than the microbiome) by including the covariates in the microbiome taxonomic table. This enables controlling for potentially confounding factors, such as individual age and sex. It also enables controlling for ancestry (or population substructure) by including the principal components (PCs) of the genetic variation data [18, 19] in the analysis. We performed 2 analyses using HMP data, 1 including host genetic PCs as covariates (results in Supplementary Table S1), and 1 without these covariates (Supplementary Table S2), both including sex as a covariate.

### Synthetic data sets

To test the performance of HOMINID, we generated several synthetic data sets. “Taxon” absolute abundances (“counts”) were drawn from a log-series distribution. The log-series distribution is frequently used to represent species abundances (e.g., [20]), and it allows a range of abundances that spans several orders of magnitude, mimicking both rare and abundant taxa. Often in real abundance tables, a large fraction of taxa have an abundance of zero (taxon either not present or not detected). The log-series abundance tables also had this quality; in our synthetic data, 21% of abundances have a count of zero. Synthetic data were generated such that, for each SNP independently, *N_ctc_* (“ctc” stands for correlated-taxon count) random taxa's abundances correlate with that SNP's genotype. Uncorrelated SNPs were created by permuting the sample IDs, preserving the minor allele frequency (MAF). Once the SNP and taxon abundance data were generated, a measure of the effect size was calculated: the coefficient of determination, $R_{OLS}^2,$ for an OLS multiple regression between the correlated taxa's abundances and the SNP genotype. As $R_{OLS}^2$ is a characteristic of the input data before analysis by HOMINID, we call it the “input *R*^2^” to distinguish it from the *R*^2^ output by the HOMINID Lasso regression (i.e., the “output *R*^2^” or $R_L^2$). To examine data sets with smaller effect sizes, “noise” was added to the SNP data by swapping the genotypes of pairs of samples, reducing the correlation between the *N_ctc_* correlated taxa and the host SNP genotype. In data sets with noise level *P*, the probability that a random sample's genotypes are *not* correlated with the correlated-taxa's abundances is *P.* Several data sets were created with progressively more “noise,” until $R_{OLS}^2$ → 0. We created 3 sets of synthetic data to examine the performance of HOMINID on different qualities of the input data: Data set MAF varies the minor allele frequency, with MAF ranging from 0.10 to 0.50; data set CTC varies the number of correlated taxa from 5 to 20; and data set TC varies the total number of taxa in the taxon table from 100 to 500. All data sets contain 500 SNPs each. Data in sets MAF and CTC comprise 1000 individuals; data sets in set TC contain 100 individuals. Data sets MAF and TC all have 3 correlated taxa per SNP. The MAF for data sets CTC and TC is 0.30.

### Human Microbiome Project data

In addition to the synthetic data sets described above, we also tested our method on a real data set that includes both human genetic and microbiome data [7]. This data set includes 93 individuals for whom microbiome was profiled as part of the Human Microbiome Project, and for which host genetic variation information was extracted from shotgun metagenomics sequence data, as described previously [7]. We annotated the previously described set of 4.2 million high-quality SNPs using ANNOVAR [21] and focused the analysis on a set of 32 696 protein-coding SNPs. We further filtered this set to include only SNPs with a minor allele frequency of at least 20% and SNPs for which we had data for at least 50 individuals. The number of SNPs actually tested varies across body sites, ranging from 12 400 to 14 651 SNPs, with a mean of 14 023. For the stool microbiome data, which included 107 total taxa, running HOMINID on 14 469 SNPs using 12-core Intel Xeon E5–2680 2.50 GHz processors took 16 cpu hours.

### Comparison with other methods

Permutational multivariate analysis of variance (PERMANOVA) [22, 23] analysis was done in R with the adonis function in the vegan [24] package. The model formula has the SNP genotype as numeric (not factor) predictor variables and the arcsin-sqrt transformed taxon relative abundance table as a response variable. The method used to calculate pairwise “distances” was default Bray-Curtis. Microbiome regression-based kernel association test (MiRKAT) [25] analysis was performed using the MiRKAT package in R. The Bray-Curtis dissimilarity matrix was computed on the arcsin-sqrt transformed taxon table. The matrix was then converted to a kernel matrix, and MiRKAT invoked for each SNP. As both PERMANOVA and MiRKAT output *P*-values as measures of how well the taxon abundances correlate with each SNP's genotype (whereas HOMINID outputs $R_L^2$ values), we chose a cutoff *P*-value such that *q*(*p_c_*) = 0.1 to separate the prediction positives (correlated) from the prediction negatives (uncorrelated), much in the same way we chose the cutoff $R_C^2$ to separate prediction positive/negatives such that $q( {R_C^2} )$ = 0.1 for the Lasso regression.

## Results

### Analysis using synthetic data

To assess HOMINID's performance, we first used the pipeline on a comprehensive set of synthetic data sets (described above and in the Supplementary Data). These data sets were designed to simulate variation in several important factors, such as variation of the strength of correlation (the input *R*^2^) of the associated SNP with microbiome composition, variation in MAF of the associated SNP, noise level in microbiome data, and the number of taxa associated with the SNP. After analyzing each of the data sets, we calculated and plotted the method's sensitivity, specificity, precision, negative predictive value (NPV), false positive rate (FPR), false negative rate (FNR), false discovery rate, and accuracy as a function of the input *R*^2^, highlighting the effects of the variable factors above (see Fig. 2a–d, Supplementary Data, and Supplementary Figs S5–S44).

**Figure 2:**
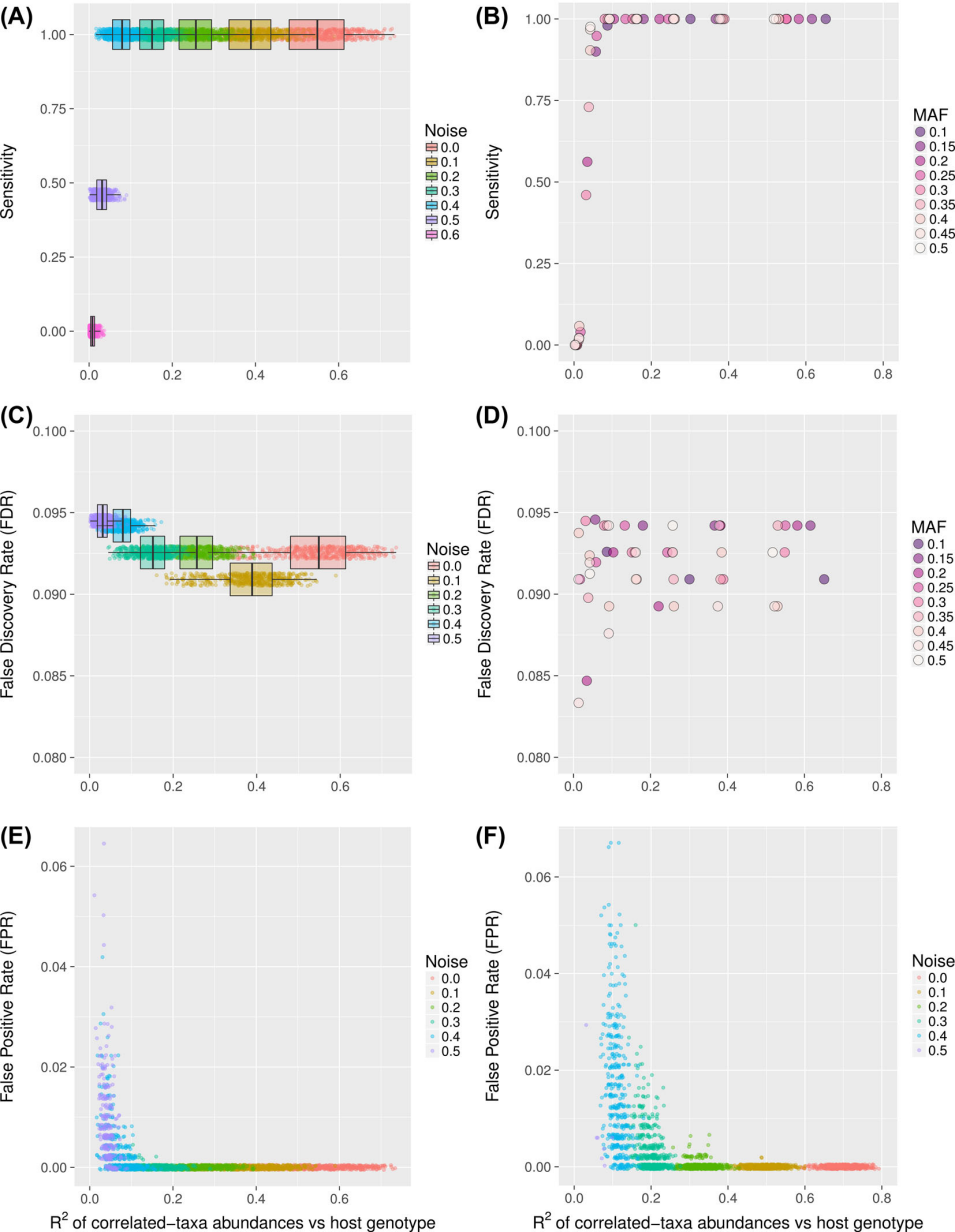
Assessment of HOMINID's performance using synthetic data. Panels (**A–D**) assess how well HOMINID predicts the SNPs whose genotypes correlate with microbiome abundances, and (**E** and **F**) assess how well HOMINID predicts the specific taxa correlated with an associated SNP. (A) Sensitivity as a function of effect size (input *R*^2^) for the data sets with MAF = 0.30. Different colored points and boxplots represent data sets with different noise levels and therefore different effect sizes. (B) Same as (A) with variation in input data MAF values represented by different colored points at each data set's median-input *R*^2^. See Supplemental Fig. S30 for a visualization of the same data with boxplots instead of medians. (C) FDR as a function of effect size (input *R*^2^) for data sets with MAF = 0.30. (D) Same as (C) with variation in input MAF values represented by different colored points at each data set's median-input *R*^2^. See Supplemental Fig. S35 for a visualization of the same data with boxplots instead of medians. (E) FPR for the stability selection step (identifying the taxa that associate with an SNP's genotype) as a function of effect size (input *R*^2^) for data sets with 3 correlated taxa. (F) Same as (E) but with 20 correlated taxa.

We found that the strength of correlation (input *R*^2^) between SNP genotype and the correlated taxa has little effect on HOMINID's ability to identify the SNP, unless the correlation is very low (Fig. 2a and b, Supplementary Data, and Supplementary Figs S5–S12). HOMINID achieved high sensitivity and specificity for *R*^2^ values of above ∼0.05. The FDR is below 0.1 by design, and variation in FDR is due to imprecision (finite number of significant digits) in calculation of $R_L^2$, and therefore imprecision in calculation of *q*. (Fig. 2c and d). Similarly, variation in MAF does not affect HOMINID's sensitivity, as data sets with different MAFs follow the same behavior (Fig. 2b).

One of HOMINID's unique features is the ability to identify the taxa that are correlated with an associated SNP. We found that this prediction performs well, with accuracy approaching 1 and a false positive rate of 0 for input *R*^2^ values larger than about 0.1, but it drops off at lower *R*^2^ values (Fig. 2e and f, Supplementary Figs S27 and S28). The number of correlated taxa had a noticeable effect, whereby SNPs that correlated with more taxa had higher FPRs (compare Fig. 2e with Fig. 2f), although in all test data sets FPR remained <0.07.

### Comparison with other methods

**Figure 3:**
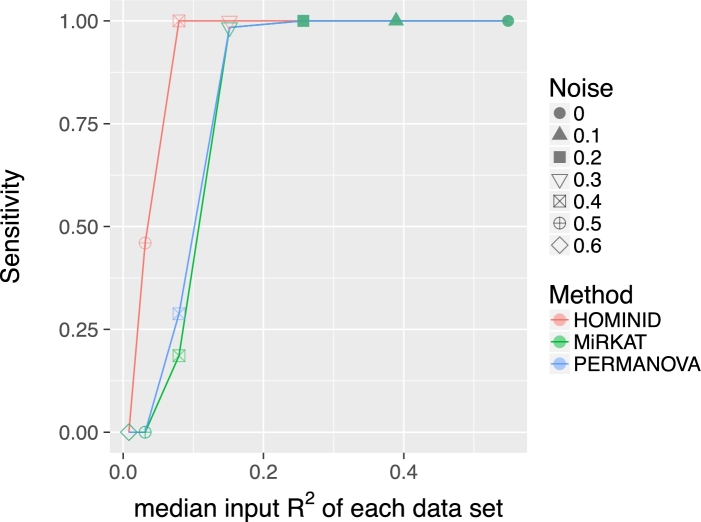
Comparison of the performance of HOMINID vs MiRKAT and PERMANOVA. Sensitivity is plotted as a function of effect size (input *R*^2^) for HOMINID (red), MirKAT (green), and PERMANOVA (blue). At high-input *R*^2^, all 3 methods perform well, finding all SNPs that correlate with the microbiome. However, at smaller effect sizes (lower-input *R*^2^), HOMINID is more sensitive.

In order to assess HOMINID's performance, we compared it with PERMANOVA [22, 23] and MiRKAT [25], 2 platforms that can be used to identify host SNPs associated with microbiome composition. We note that HOMINID has a unique feature allowing it to identify the specific microbial taxa associated with each SNP. As other approaches lack this option, the comparison centered on the ability to detect SNPs that are correlated with the microbiome, and not on the detection of correlated taxa. Our analysis included input data sets with various input *R*^2^ values and noise levels (various effect sizes) and compared the sensitivity of each method to detect the associated SNPs. We found that for median-input *R*^2^ values (correlation between associated SNP and microbiome composition) of about 0.15 or above, the 3 methods are all highly sensitive (Fig. 3). However, for lower-input *R*^2^ values, HOMINID is more sensitive. Specifically, for the data set with median-input *R*^2^ = 0.08, HOMINID's sensitivity is 1, while the sensitivities of MiRKAT and PERMANOVA are 0.19 and 0.29, respectively (Fig. 3). Similarly, for median-input *R*^2^ = 0.03, HOMINID's sensitivity is 0.46, while the other methods’ sensitivities are 0.

### Analysis of human microbiome project data

**Figure 4:**
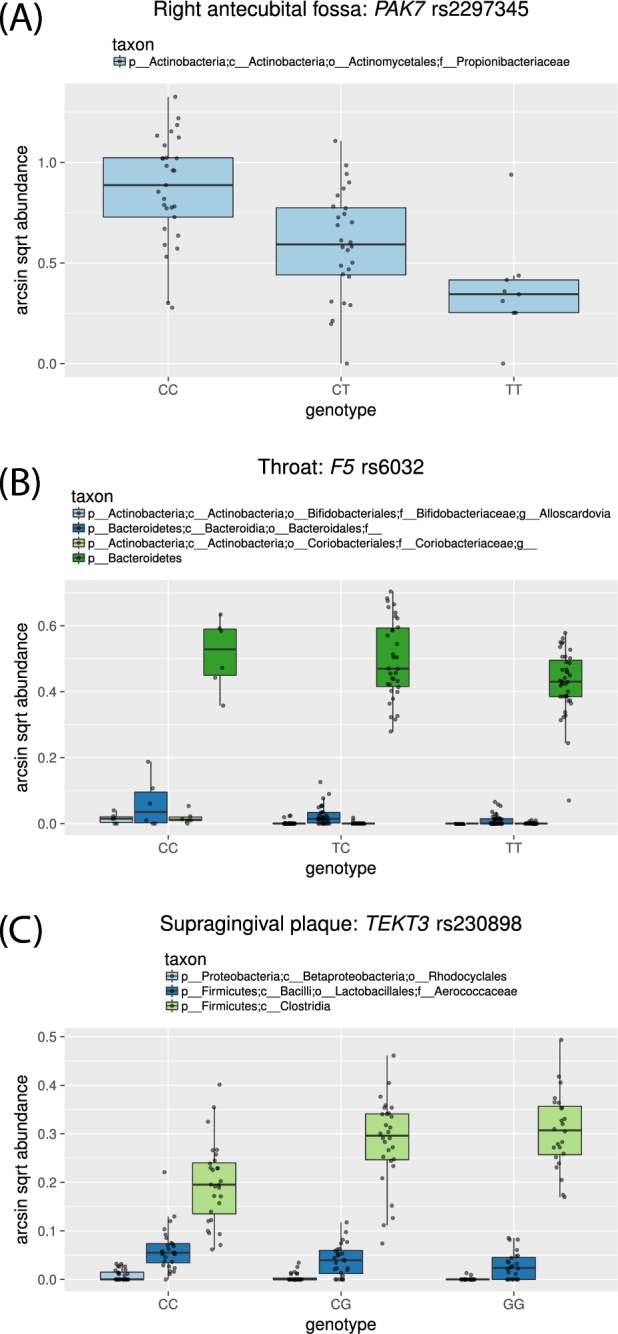
Examples of SNPs where correlations were found between host genetic variation and the microbiome. Three SNPs are shown: (**A**) rs2297345 (correlated with abundance of microbial taxa in the right antecubital fossa), (**B**) rs6032 correlated with abundance of microbial taxa in the throat), and (**C**) rs230898 (correlated with abundance of microbial taxa in the supragingival plaque). The x-axis shows the host SNP genotypes, and the y-axis shows the arcsin sqrt transformed taxon abundances. The different correlated taxa for each SNP are shown in different colors. See Fig. S59 for a visualization of the results in (4B), but omitting the highest-abundance taxon, Bacteroidetes (dark green), to better display the trends for the 3 lower-abundance taxa.

We ran the HOMINID pipeline on previously published data of microbiome and host genetic variation from the Human Microbiome Project cohort [7]. We focused our analysis on coding SNPs with minor allele frequency ≥ 0.2 and identified SNPs for which permutation-based *q*-value ≤0.1 and the 95th percentile confidence interval for $R_L^2$ does not include zero. To account for population substructure, we ran a second analysis including the genetic principal components as additional covariates [18, 19]. This resulted in the identification of 11 (regression with genetic PCs as covariates) and 6 (regression without genetic PCs) for a total of 13 unique associations between host SNP and microbiome composition across 15 body sites (see Supplementary Tables S1 and S2, respectively). As can be seen in Fig. 4, HOMINID is able to detect SNPs with the expected pattern of association between host genetic variation and the microbiome. For example, for SNP rs2297345 in the gene *PAK7*, we detected a correlation between genotype and a single microbial taxon, Propionibacteriaceae (Fig. 4a). HOMINID can also detect SNPs where multiple taxa are correlated with the same SNP (e.g., SNP rs6032 in Fig. 4b), as well as more complex patterns of association; for example, for SNP rs230898 in the gene *TEKT3* (Fig. 4c), genetic variation is positively correlated with 1 taxon (Clostridia) and negatively with others (Rhodocyclales and Aerococcaceae).

Although HOMINID performs strongly on the data used in this paper, there are several potential limitations to our method. First, as it is especially designed to identify SNPs where a number of taxa are associated, it might not be optimal for cases where there is a dramatic shift in the microbiome that includes many dozens of taxa. Moreover, as the SNP is used as the response in the HOMINID model, it is difficult to identify epistatic effects whereby genetic variation in 2 or more loci interact to affect microbiome composition. Although HOMINID could still be used to detect these interactions by including all genotype combinations as response variables, multiple hypothesis testing could be an issue, especially for microbiome association studies, where samples sizes are currently small relative to GWAS of other complex traits. Nevertheless, HOMINID might be useful for detection of interaction of between candidate loci.

Lastly, we developed a web-based tool for the visualization of host-microbiome interaction network identified in HOMINID [26]. The website, designed using D3.js with a dedicated MySQL database serving as the back end, displays a dynamic visualization of host gene–microbiome taxa interaction networks and allows the user to add and remove nodes (host gene and microbial taxa), adjust the display size and node locations, filter by body sites, and generate figures. Currently, the website includes toy data representing all SNP-microbe associations with a nominal *P*-value ≤ .01 in the Human Microbiome Project data described above. We believe that as studies using larger sample sizes materialize (for example, a recent study included 1514 subjects [13]), this tool will be useful for visualization of much larger number of associations.

## Conclusions

We present HOMINID, a framework designed for identifying associations between host genetic variation and microbiome composition. We analyze synthetic data to show HOMINID's overall strong performance, identify specific factors that may affect it, highlight HOMINID's unique features, and show HOMINID's utility with a real data set. We expect that HOMINID would be useful for studies attempting to characterize the genetic basis of host-microbiome interactions.

## Availability of supporting source code and requirements

- Project name: HOMINID (RRID: SCR_015765)
- Project home page: https://github.com/blekhmanlab/hominid
- Operating system(s): UNIX
- Programming language: Python
- Other requirements: see https://github.com/blekhmanlab/hominid/wiki/Requirements
- License: MIT

## Availability of supporting data

The real data set used in this study is from Blekhman et al. [7]; 16S rRNA gene sequence data and OTU tables are available from the NIH Human Microbiome Project DACC website [27]. SNP data including host genetic data are deposited in dbGaP under project number phs000228.

The synthetic data used in this work can be accessed from the HOMINID GitHub page [28]. HMP taxon tables and other supporting metadata are available from the *GigaScience* repository, *Giga*DB [29].

## Additional files

Supplementaryinfo.pdf

supplementalTableS1.xlsx

supplementalTableS2.xlsx

## Abbreviations

CTC: correlated-taxon count; FDR: false discovery rate; FNR: false negative rate; FPR: false positive rate; MAF: minor allele frequency; NPV: negative predictive value; OLS: ordinary least square; QTL: quantitative trait locus; OTU: operational taxonomic unit; PC: principal component; SNP: single nucleotide polymorphism; VCF: variant call format.

## Competing Interests

The authors declare that they have no competing interests.

## Funding

This work is supported in part by funds from the University of Minnesota College of Biological Sciences, The Randy Shaver Cancer Research and Community Fund, Institutional Research Grant No. 124166-IRG-58–001-55-IRG53 from the American Cancer Society, and a Research Fellowship from The Alfred P. Sloan Foundation. This work was facilitated in part by computational resources provided by the Minnesota Supercomputing Institute.

## Author contributions

J.L., K.T., and R.B. designed the study. J.L. and K.T. performed the analysis with help from S.P., J.S., M.S., and E.T. and advice from R.B., S.M., and D.K. J.L., K.T., and R.B. wrote the paper.

## Supplementary Material

GIGA-D-16-00138_Original-Submission.pdf

GIGA-D-16-00138_Revision-1.pdf

GIGA-D-16-00138_Revision-2.pdf

GIGA-D-16-00138_Revision-3.pdf

Response-to-Reviewer-Comments_Original-Submission.pdf

Response-to-Reviewer-Comments_Revision-1.pdf

Response-to-Reviewer-Comments_Revision-2.pdf

Reviewer-1-Report-(Original-Submission).pdf

Reviewer-1-Report-(Revision-1).pdf

Reviewer-2-Report-(Original-Submission).pdf

Reviewer-2-Report-(Revision-1).pdf

Supplement materials

## References

[1] Consortium, Human Microbiome Project. Structure, function and diversity of the healthy human microbiome. Nature 2012;486:207–14. 22699609 10.1038/nature11234 PMC3564958

[2] Goodrich JK, Davenport ER, Waters JL Cross-species comparisons of host genetic associations with the microbiome. Science 2016;352:532–5. 27126034 10.1126/science.aad9379 PMC5116907

[3] Morton ER, Lynch J, Froment A Variation in rural African gut microbiota is strongly correlated with colonization by entamoeba and subsistence. PLoS Genet 2015;11:e1005658. 26619199 10.1371/journal.pgen.1005658 PMC4664238

[4] Tong M, Mchardy I, Ruegger P Reprograming of gut microbiome energy metabolism by the FUT2 Crohn's disease risk polymorphism. ISME J 2014;8:2193–206. 24781901 10.1038/ismej.2014.64 PMC4992076

[5] Khachatryan ZA, Ktsoyan ZA, Manukyan GP Predominant role of host genetics in controlling the composition of gut microbiota. PLoS One 2008;3:e3064. 18725973 10.1371/journal.pone.0003064 PMC2516932

[6] Knights D, Silverberg MS, Weersma RK Complex host genetics influence the microbiome in inflammatory bowel disease. Genome Med 2014;6:107. 25587358 10.1186/s13073-014-0107-1 PMC4292994

[7] Blekhman R, Goodrich JK, Huang K Host genetic variation impacts microbiome composition across human body sites. Genome Biol 2015;16:191. 26374288 10.1186/s13059-015-0759-1 PMC4570153

[8] Goodrich JK, Waters JL, Poole AC Human genetics shape the gut microbiome. Cell 2014;159:789–99. 25417156 10.1016/j.cell.2014.09.053 PMC4255478

[9] Goodrich JK, Davenport ER, Beaumont M Genetic determinants of the gut microbiome in UK twins. Cell Host Microbe 2016;19:731–43. 27173935 10.1016/j.chom.2016.04.017 PMC4915943

[10] Davenport ER, Cusanovich DA, Michelini K Genome-wide association studies of the human gut microbiota. PLoS One 2015;10:e0140301. 26528553 10.1371/journal.pone.0140301 PMC4631601

[11] Benson AK, Kelly SA, Legge R Individuality in gut microbiota composition is a complex polygenic trait shaped by multiple environmental and host genetic factors. Proc Natl Acad Sci U S A 2010;107:18933–8. 20937875 10.1073/pnas.1007028107 PMC2973891

[12] Leamy LJ, Kelly SA, Nietfeldt J Host genetics and diet, but not immunoglobulin A expression, converge to shape compositional features of the gut microbiome in an advanced intercross population of mice. Genome Biol 2014;15:552. 25516416 10.1186/s13059-014-0552-6 PMC4290092

[13] Bonder MJ, Kurilshikov A, Tigchelaar EF The effect of host genetics on the gut microbiome. Nat Genet 2016; http://dx.doi.org/10.1038/ng.3663. 10.1038/ng.3663 27694959

[14] Turpin W, Espin-Garcia O, Xu W Association of host genome with intestinal microbial composition in a large healthy cohort. Nat Genet 2016;48:1413–7. 27694960 10.1038/ng.3693

[15] Pedregosa F, Varoquaux G, Gramfort A Scikit-learn: machine learning in python. J Mach Learn Res 2011;12:2825–30.

[16] Subramanian A, Tamayo P, Mootha VK Gene set enrichment analysis: a knowledge-based approach for interpreting genome-wide expression profiles. Proc Natl Acad Sci U S A 2005;102:15545–50. 16199517 10.1073/pnas.0506580102 PMC1239896

[17] Meinshausen N, Bühlmann P Stability selection. J R Stat Soc Series B Stat Methodol 2010;72:417–73.

[18] Price AL, Patterson NJ, Plenge RM Principal components analysis corrects for stratification in genome-wide association studies. Nat Genet 2006;38:904–9. 16862161 10.1038/ng1847

[19] Pritchard JK, Stephens M, Rosenberg NA Association mapping in structured populations. Am J Hum Genet 2000;67:170–81. 10827107 10.1086/302959 PMC1287075

[20] Baldridge E, Harris DJ, Xiao X An extensive comparison of species-abundance distribution models. PeerJ 2016;4:e2823. 28028483 10.7717/peerj.2823 PMC5183127

[21] Wang K, Li M, Hakonarson H ANNOVAR: functional annotation of genetic variants from high-throughput sequencing data. Nucleic Acids Res 2010;38:e164. 20601685 10.1093/nar/gkq603 PMC2938201

[22] Anderson MJ A new method for non-parametric multivariate analysis of variance. Austral Ecol 2001;26:32–46.

[23] McArdle BH, Anderson MJ Fitting multivariate models to community data: a comment on distance-based redundancy analysis. Ecol 2001;82:290–7.

[24] Oksanen J, Kindt R, Legendre P The vegan package. Community Ecology Package 2007;10:631–7.

[25] Zhao N, Chen J, Carroll IM Testing in microbiome-profiling studies with MiRKAT, the microbiome regression-based kernel association test. Am J Hum Genet 2015;96:797–807. 25957468 10.1016/j.ajhg.2015.04.003 PMC4570290

[26] OTU Gene Diagram. http://z.umn.edu/genemicrobe. Accessed August 2016.

[27] NIH Human Microbiome Project DACC. https://www.hmpdacc.org/. Accessed February 2014.

[28] The HOMINID Project synthetic data. https://github.com/blekhmanlab/hominid/tree/master/synthetic-data. Accessed July 2017

[29] Lynch J, Tang K, Priya S Supporting data for “HOMINID: a framework for identifying associations between host genetic variation and microbiome composition.” GigaScience Database 2017 http://dx.doi.org/10.5524/100367. 10.1093/gigascience/gix107 PMC5740987 29126115

