# Supplementary material for: HOMINID: a framework for identifying associations between host genetic variation and microbiome composition
Source: Gigascience. 2017 Nov 8;6(12):1–7. doi: 10.1093/gigascience/gix107 (PMC5740987; doi:10.1093/gigascience/gix107)
Supplement: GIGA-D-16-00138_Original-Submission.pdf [file gix107_giga-d-16-00138_original-submission.pdf]

GigaScience

HOMINID: A framework for identifying associations between host genetic variation and microbiome composition

--Manuscript Draft--

**Manuscript Number:**	GIGA-D-16-00138
**Full Title:**	HOMINID: A framework for identifying associations between host genetic variation and microbiome composition
**Article Type:**	Technical Note
**Funding Information:**	The Randy Shaver Cancer Research and Community Fund — Dr. Ran Blekhman<br>American Cancer Society (US) (124166-IRG-58-001-55-IRG53) — Dr. Ran Blekhman<br>Alfred P. Sloan Foundation — Dr. Ran Blekhman
**Abstract:**	Recent studies have uncovered a strong effect of host genetic variation on the composition of host-associated microbiota. Here, we present HOMINID, a computational approach based on Lasso linear regression, that given host genetic variation and microbiome composition data, identifies host SNPs that are correlated with microbial taxa abundances. By using HOMINID on data from the Human Microbiome Project, we identified 2,127 human SNPs in which genetic variation is correlated with microbiome taxonomic composition in 15 body sites. We also present a tool for visualization of host-microbiome association network identified in HOMINID.
**Corresponding Author:**	Ran Blekhman<br>University of Minnesota Twin Cities<br>UNITED STATES
**Corresponding Author Secondary Information:**	
**Corresponding Author's Institution:**	University of Minnesota Twin Cities
**Corresponding Author's Secondary Institution:**	
**First Author:**	Joshua Lynch
**First Author Secondary Information:**	
**Order of Authors:**	Joshua Lynch<br>Karen Tang<br>Joanna Sands<br>Margaret Sands<br>Evan Tang<br>Sayan Mukherjee<br>Dan Knights<br>Ran Blekhman
**Order of Authors Secondary Information:**	
**Opposed Reviewers:**	
**Additional Information:**	
**Question**	**Response**
Are you submitting this manuscript to a special series or article collection?	No
**Experimental design and statistics**	Yes

Powered by Editorial Manager® and ProduXion Manager® from Aries Systems Corporation

Full details of the experimental design and statistical methods used should be given in the Methods section, as detailed in our Minimum Standards Reporting Checklist. Information essential to interpreting the data presented should be made available in the figure legends.<br><br>Have you included all the information requested in your manuscript?	
**Resources**<br><br>A description of all resources used, including antibodies, cell lines, animals and software tools, with enough information to allow them to be uniquely identified, should be included in the Methods section. Authors are strongly encouraged to cite Research Resource Identifiers (RRIDs) for antibodies, model organisms and tools, where possible.<br><br>Have you included the information requested as detailed in our Minimum Standards Reporting Checklist?	Yes
**Availability of data and materials**<br><br>All datasets and code on which the conclusions of the paper rely must be either included in your submission or deposited in publicly available repositories (where available and ethically appropriate), referencing such data using a unique identifier in the references and in the "Availability of Data and Materials" section of your manuscript.<br><br>Have you have met the above requirement as detailed in our Minimum Standards Reporting Checklist?	Yes

Manuscript
Click here to download Manuscript Lynchmaintext.wFig1.pdf

HOMINID: A framework for identifying associations between host genetic variation and microbiome composition

Joshua Lynch[1,2], Karen Tang[1,2], Joanna Sands[1,2], Margaret Sands[1,2], Evan Tang[1,2], Sayan Mukherjee[3], Dan Knights[4,5,*], Ran Blekhman[1,2,*]

[1] Department of Genetics, Cell Biology, and Development, University of Minnesota, Minneapolis, MN, USA

[2] Department of Ecology, Evolution, and Behavior, University of Minnesota, Minneapolis, MN

[3] Departments of Statistical Science, Mathematics, and Computer Science, Duke University, Durham, NC

[4] Department of Computer Science and Engineering, University of Minnesota, Minneapolis, MN, USA

[5] Biotechnology Institute, University of Minnesota, Minneapolis, MN, USA

*To whom correspondence should be addressed: dknights@umn.edu (DK), blekhman@umn.edu (RB)

Abstract

Summary: Recent studies have uncovered a strong effect of host genetic variation on the composition of host-associated microbiota. Here, we present HOMINID, a computational approach based on Lasso linear regression, that given host genetic variation and microbiome composition data, identifies host SNPs that are correlated with microbial taxa abundances. By using HOMINID on data from the Human Microbiome Project, we identified 2,127 human SNPs in which genetic variation is correlated with microbiome taxonomic composition in 15 body sites. We also present a tool for visualization of host-microbiome association network identified in HOMINID.

Availability and implementation: Software and code are available at https://github.com/blekhmanlab/hominid, online visualization tool at http://z.umn.edu/genemicrobe.

Contact: blekhman@umn.edu

Supplementary information: Supplementary data are available at *Bioinformatics* online

1. Introduction

The microbial communities found in and on the human body are influenced by multiple factors. In addition to the clear effect of environmental factors on the microbiome, there is growing support for an impact of host genetics. Several candidate gene studies have found correlation between human genetic variation and the structure of the microbiome (Tong *et al.*, 2014; Khachatryan *et al.*, 2008; Knights *et al.*, 2014). In addition, genome-wide approaches can also be useful to identify human genetic impact on the microbiome (Goodrich *et al.*, 2014; Blekhman *et al.*, 2015; Goodrich *et al.*, 2016). For example, Goodrich et al. used hundreds of twin pairs to calculate the heritability of the gut microbiome, and identify bacterial taxa that are highly heritable, such as Christensenellaceae (Goodrich *et al.*, 2014). Researchers have also utilized quantitative trait locus (QTL)-mapping approaches in the laboratory mouse and have identified multiple loci associated with the structure of gut microbial communities, some of which overlap genes involved in immune response (Benson *et al.*, 2010; Leamy *et al.*, 2014). Moreover, studies have used joint human genetic variation and microbiome data to find associations between loci in the human genome and microbial taxa (Blekhman *et al.*, 2015; Davenport *et al.*, 2015). In our recent study, in addition to showing that human genetic variation is associated with the structure of microbial communities across ten body sites, we have identified human single nucleotide polymorphisms (SNPs) associated with variation in the microbiome, and found that these loci are highly enriched in immunity genes and pathways (Blekhman *et al.*, 2015). This approach, which includes the joint analysis of host genetic variation (SNPs) and microbiome taxonomic composition data (usually an OTU table), has the important advantage of identifying specific host genes and pathways that may control the microbiome, thus shedding light on the biological mechanisms of host-microbiome interaction, and pinpointing potential disease-causing pathways. However, this analysis is complicated by the fact that the microbiome contains many taxa that can be used as potential molecular complex traits in the GWAS analysis. Testing many taxa reduces the power and multiple hypothesis testing correction makes the identification of associations challenging.

2. Approach

Here, we propose a framework for identifying host SNPs associated with microbiome composition using Lasso regression, named **HOMINID** (**Ho**st-**Mi**crobiome **In**teraction **Id**entification; see **Figure 1A** and **Supplementary Information**). Our method takes as input host genetic variation data (in a modified VCF format) and microbiome composition data (as an OTU table), and uses Lasso regression plus stability selection with randomized Lasso to identify

associations between host SNPs and microbiome taxa. We implemented Lasso regression using the Python machine-learning library scikit-learn (Pedregosa *et al.*, 2011) with the taxon relative abundances (arcsin sqrt transformed) as predictors, and genetic variation at each SNP as response, for the purpose of identifying an additive effect between allele count and microbiome features (see **Supplementary Information** and **Figures S1-S3**). In addition, our model can easily include any other factors that can affect the microbiome (e.g., sex) as covariates. We use five-fold cross-validation and use the coefficient of determination from the Lasso model, R^2, as a measure of correlation. To reduce variability in R^2 we reshuffle the data and calculate the median in R^2, as well as a 95th percentile bootstrap confidence interval of the median using 10,000 bootstrap samples. Lastly, we assign a P- and Q-values to the R^2 using permutation of the sample labels (see **Supplementary Information)**. Using synthetic data, we show that the accuracy of our approach is robust with respect to allele frequency (**Figures S4** and **S5**), and variation in microbiome composition within each allele group has a relatively minor effect on accuracy (**Figures S6** and **S7**).

3. Results

We ran the HOMINID pipeline on a previously published data of microbiome and host genetic variation from the Human Microbiome Project cohort (Blekhman *et al.*, 2015). We focused our analysis on coding SNPs with minor allele frequency ≥ 0.2, and identified SNPs for which permutation-based P-value ≤ 0.01 and the 95th percentile confidence interval for R^2 does not include zero. This resulted in the identification of 2,127 associations between host SNP and microbiome composition across 15 body sites (see **Fig. 1B**, **Table S1**, and Supplementary methods). On average, we identified 142 associated SNPs in each body site, with most found in the gut (197 SNPs) and fewest in the airways (107 SNPs). These SNPs were located within 1,532 distinct human genes (see **Table S2** for the detailed list). Of these, 296 genes harbor SNPs that are correlated with the microbiome in more than one body site, with 13 genes in which we find correlations in at least 4 different body sites (including *STAB1*, *ADAMTS17*, *AHNAK2*, *AKAP12*, *CD109*, *FBN3*, *HAUS6*, *MKI67*, *MUC5B*, *OBSCN*, *TBC1D10A*, *TEX15,* and *UNC79*; **Table S2**). Only six SNPs remained significant after multiple hypothesis testing correction (at Q-value < 0.1; see **Table S3**). However, as studies using larger sample sizes materialize (for example, a recent study included 1,514 subjects (Bonder *et al.*, 2016)), we expect our method to be useful in allowing the detection of a much larger number of associations.

We find that among these genes there are several interesting candidates; for example, one of the most significant correlations we find with the gut microbiome is a SNP located within *ABCC8*, a gene that is expressed in the colon and encodes a protein involved in insulin release with a strong link to diabetes mellitus (Hlavata *et al.*, 2012; Haghverdizadeh *et al.*, 2014). We

find that genetic variation in SNP rs1799859 within *ABCC8* is correlated with the abundance of taxa Veillonella, Lentisphaerae, Clostridiaceae, and Dialister (R^2=0.194, P = 1.11×10^{-4}, q=0.5 via permutation test), all of which have been previously associated with diabetes (Murri *et al.*, 2013; Fugmann *et al.*, 2015; Kostic *et al.*, 2015). We find another interesting correlation between microbiome composition in the throat and SNPs within both *HLA-DRA* and *HLA-DQB1*, two genes involved in the regulation of the immune system (P = 5×10^{-4} and P = 6×10^{-4}, respectively, using permutation test). We provide an example of the patterns that can be detected using our approach as seen in the SNP rs2305243 in *ATL2* in the airway (**Figure 1C**; R^2=0.201, P = 1.22×10^{-4}), where genetic variation is positively correlated with some taxa (e.g., Bacillales) and negatively with others (e.g., Corynebacteriaceae).

Lastly, we developed a web-based tool for the visualization of host-microbiome interaction network identified in HOMINID, available at http://z.umn.edu/genemicrobe. The website, designed using D3.js with a dedicated MySQL database serving as the back-end, displays a dynamic visualization of host gene-microbiome taxa interaction networks, and allows the user to add and remove nodes (host gene and microbial taxa), adjust the display size and node locations, filter by body sites, and generate figures.

Funding

This work is supported in part by funds from the University of Minnesota College of Biological Sciences, The Randy Shaver Cancer Research and Community Fund, Institutional Research Grant #124166-IRG-58-001-55-IRG53 from the American Cancer Society, and a Research Fellowship from The Alfred P. Sloan Foundation. This work was facilitated in part by computational resources provided by the Minnesota Supercomputing Institute.

Figure Legend

Figure 1. Overview of the HOMINID approach and results. A. Outline of the pipeline. **B.** The number of SNPs identified (x-axis) as correlated with microbiome composition in each body site (y-axis). Colors denote skin (purple), airways (blue), oral (green), and gut (orange) sites. **C.** Visualization of the correlations found between genetic variation in SNP rs2305243 (x-axis) and microbiome composition (y-axis). Each of five correlated taxa is shown using a different color.

References

Benson,A.K. *et al.* (2010) Individuality in gut microbiota composition is a complex polygenic trait shaped by multiple environmental and host genetic factors. *Proc. Natl. Acad. Sci. U. S. A.*, **107**, 18933–18938.

Blekhman,R. *et al.* (2015) Host genetic variation impacts microbiome composition across human body sites. *Genome Biol.*, **16**, 191.

Bonder,M.J. *et al.* (2016) The effect of host genetics on the gut microbiome. *Nat. Genet.*

Davenport,E.R. *et al.* (2015) Genome-Wide Association Studies of the Human Gut Microbiota. *PLoS One*, **10**, e0140301.

Fugmann,M. *et al.* (2015) The stool microbiota of insulin resistant women with recent gestational diabetes, a high risk group for type 2 diabetes. *Sci. Rep.*, **5**, 13212.

Goodrich,J.K. *et al.* (2016) Genetic Determinants of the Gut Microbiome in UK Twins. *Cell Host Microbe*, **19**, 731–743.

Goodrich,J.K. *et al.* (2014) Human genetics shape the gut microbiome. *Cell*, **159**, 789–799.

Haghverdizadeh,P. *et al.* (2014) ABCC8 genetic variants and risk of diabetes mellitus. *Gene*, **545**, 198–204.

Hlavata,I. *et al.* (2012) The role of ABC transporters in progression and clinical outcome of colorectal cancer. *Mutagenesis*, **27**, 187–196.

Khachatryan,Z.A. *et al.* (2008) Predominant role of host genetics in controlling the composition of gut microbiota. *PLoS One*, **3**, e3064.

Knights,D. *et al.* (2014) Complex host genetics influence the microbiome in inflammatory bowel disease. *Genome Med.*, **6**, 107.

Kostic,A.D. *et al.* (2015) The dynamics of the human infant gut microbiome in development and in progression toward type 1 diabetes. *Cell Host Microbe*, **17**, 260–273.

Leamy,L.J. *et al.* (2014) Host genetics and diet, but not immunoglobulin A expression, converge to shape compositional features of the gut microbiome in an advanced intercross population of mice. *Genome Biol.*, **15**, 552.

Murri,M. *et al.* (2013) Gut microbiota in children with type 1 diabetes differs from that in healthy children: a case-control study. *BMC Med.*, **11**, 46.

Pedregosa,F. *et al.* (2011) Scikit-learn: Machine Learning in Python. *J. Mach. Learn. Res.*, **12**, 2825–2830.

Tong,M. *et al.* (2014) Reprograming of gut microbiome energy metabolism by the FUT2 Crohn's disease risk polymorphism. *ISME J.*, **8**, 2193–2206.

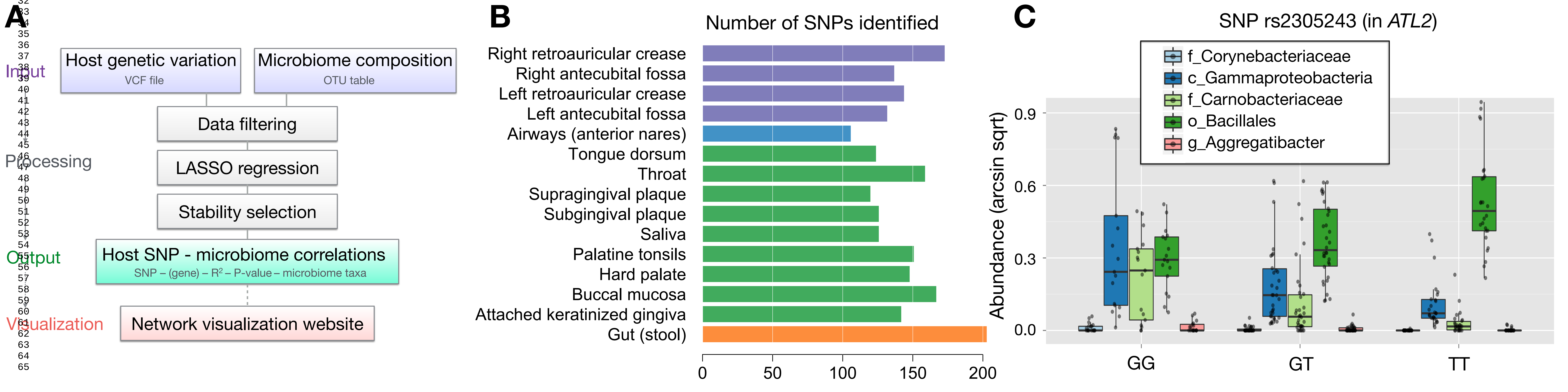
A
Input
Host genetic variation
VCF file
Microbiome composition
OTU table
Processing
Data filtering
LASSO regression
Stability selection
Output
Host SNP - microbiome correlations
SNP – (gene) – R² – P-value – microbiome taxa
Visualization
Network visualization website
B
Number of SNPs identified
Right retroauricular crease
Right antecubital fossa
Left retroauricular crease
Left antecubital fossa
Airways (anterior nares)
Tongue dorsum
Throat
Supragingival plaque
Subgingival plaque
Saliva
Palatine tonsils
Hard palate
Buccal mucosa
Attached keratinized gingiva
Gut (stool)
0
50
100
150
200
C
SNP rs2305243 (in ATL2)
f_Corynebacteriaceae
c_Gammaproteobacteria
f_Carnobacteriaceae
o_Bacillales
g_Aggregatibacter
Abundance (arcsin sqrt)
0.9
0.6
0.3
0.0
GG
GT
TT

Figure

Click here to download Figure fig1.pdf

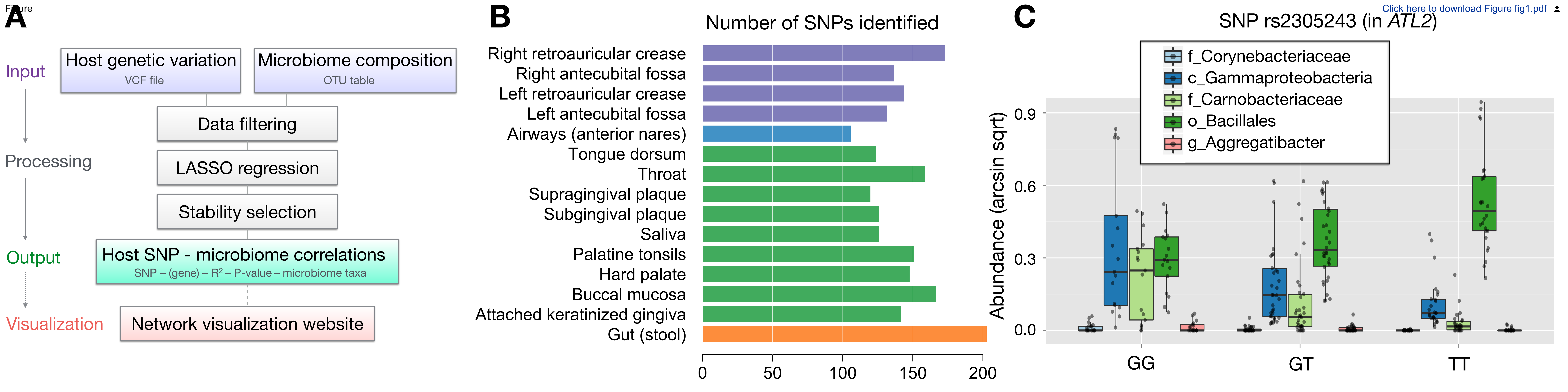

Supplementary Methods

Click here to access/download

Supplementary Material

supplMethods.pdf

Supplementary Table 1

Click here to access/download
Supplementary Material
supplTable1.xlsx

Supplementary Table 2

Click here to access/download
Supplementary Material
supplTable2.xlsx

Supplementary Table 3

Click here to access/download
Supplementary Material
supplTable3.xlsx

Personal Cover
Click here to download Personal Cover Lynch et al 2016 Cover Letter.pdf

University of Minnesota

Ran Blekhman, Ph.D.
Assistant Professor

Genetics, Cell Biology, and Development
Ecology, Evolution, and Behavior

MCB 6-126
321 Church St. SE
Minneapolis, MN 55455
Email: blekhman@umn.edu
Tel: (612) 624-4092
Web: BlekhmanLab.org

November 2, 2016
Nicole Nogoy, Ph.D.
Editor
GigaScience

Dear Dr. Nogoy,

We are delighted to submit our manuscript, "**HOMINID: A framework for identifying associations between host genetic variation and microbiome composition**", for consideration for publication in *GigaScience*.

In this manuscript, we present a novel approach for identification of host genetic variants that are associated with microbiome composition. Our approach is based on machine learning (LASSO regression) to identify SNPs in host genetic variation data that are associated with microbiome composition, and also characterize the specific microbial taxa that drive the association. We provide open source software, as well as a online tool for visualization of the associations.

We believe that our approach would be highly relevant for the field. In the last few years there has been increasing interest in host genomic control of the microbiome (Goodrich et al., *Cell* 2013; Blekhman et al., *Genome Biology* 2015; Goodrich et al., *Science* 2016; and Bonder et al. *Nature Genetics* 2016, among many others). One of the main challenges in these studies is integrating joint datasets of host genetic variation and microbiome taxonomic composition. Traditional population genetics approaches are not optimal, due to the complexity of the microbiome data and difficulty in defining microbiome "quantitative traits" to use for mapping.

To our knowledge, our work represents the first software tool specifically designed for the purpose of mapping host genetic variants associated with microbiome composition. Thus, we believe that our paper would be of high interest to the field, and that the HOMINID tool would be highly used by the microbiome research community. We believe that our paper will be of interest to a broad audience and hope that you will find this the manuscript suitable for publication in *GigaScience*.

Thank you in advance for your consideration, and we look forward to hearing from you soon.

Sincerely,

Ran Blekhman, Ph.D.
Alfred P. Sloan Research Fellow and Assistant Professor
University of Minnesota, Twin Cities
Dept. of Genetics, Cell Biology, and Development | Dept. of Ecology, Evolution, and Behavior
Cargill 222, 1500 Gortner Ave., St. Paul, MN 55108
BlekhmanLab.org | Twitter: @blekhman | Phone: (612) 624-4092 | Fax:(612) 624-6264
