# Supplementary material for: HOMINID: a framework for identifying associations between host genetic variation and microbiome composition
Source: Gigascience. 2017 Nov 8;6(12):1–7. doi: 10.1093/gigascience/gix107 (PMC5740987; doi:10.1093/gigascience/gix107)
Supplement: GIGA-D-16-00138_Revision-1.pdf [file gix107_giga-d-16-00138_revision-1.pdf]

GigaScience

HOMINID: A framework for identifying associations between host genetic variation and microbiome composition

--Manuscript Draft--

**Manuscript Number:**	GIGA-D-16-00138R1
**Full Title:**	HOMINID: A framework for identifying associations between host genetic variation and microbiome composition
**Article Type:**	Research
**Funding Information:**	The Randy Shaver Cancer Research and Community Fund — Dr. Ran Blekhman<br>American Cancer Society (US) (124166-IRG-58-001-55-IRG53) — Dr. Ran Blekhman<br>Alfred P. Sloan Foundation — Dr. Ran Blekhman
**Abstract:**	Recent studies have uncovered a strong effect of host genetic variation on the composition of host-associated microbiota. Here, we present HOMINID, a computational approach based on Lasso linear regression, that given host genetic variation and microbiome composition data, identifies host SNPs that are correlated with microbial taxa abundances. Using simulated data we show that HOMINID has accuracy in identifying associated SNPs, and performs better compared to existing methods. We also show that HOMINID can accurately identify the microbial taxa that are correlated with associated SNPs. Lastly, by using HOMINID on real data of human genetic variation and microbiome composition, we identified 13 human SNPs in which genetic variation is correlated with microbiome taxonomic composition across body sites. In conclusion, HOMINID is a powerful method to detect host genetic variants linked to microbiome composition, and can facilitate discovery of mechanisms controlling host-microbiome interactions.
**Corresponding Author:**	Ran Blekhman<br>University of Minnesota Twin Cities<br>UNITED STATES
**Corresponding Author Secondary Information:**	
**Corresponding Author's Institution:**	University of Minnesota Twin Cities
**Corresponding Author's Secondary Institution:**	
**First Author:**	Joshua Lynch
**First Author Secondary Information:**	
**Order of Authors:**	Joshua Lynch<br>Karen Tang<br>Sambhawa Priya<br>Joanna Sands<br>Margaret Sands<br>Evan Tang<br>Sayan Mukherjee<br>Dan Knights<br>Ran Blekhman
**Order of Authors Secondary Information:**	
**Response to Reviewers:**	Response to Reviewers is appended to the Cover Letter, at the end of the PDF.

Powered by Editorial Manager® and ProduXion Manager® from Aries Systems Corporation

Additional Information:	
**Question**	**Response**
Are you submitting this manuscript to a special series or article collection?	No
**Experimental design and statistics**<br><br>Full details of the experimental design and statistical methods used should be given in the Methods section, as detailed in our Minimum Standards Reporting Checklist. Information essential to interpreting the data presented should be made available in the figure legends.<br><br>Have you included all the information requested in your manuscript?	Yes
**Resources**<br><br>A description of all resources used, including antibodies, cell lines, animals and software tools, with enough information to allow them to be uniquely identified, should be included in the Methods section. Authors are strongly encouraged to cite Research Resource Identifiers (RRIDs) for antibodies, model organisms and tools, where possible.<br><br>Have you included the information requested as detailed in our Minimum Standards Reporting Checklist?	Yes
**Availability of data and materials**<br><br>All datasets and code on which the conclusions of the paper rely must be either included in your submission or deposited in publicly available repositories (where available and ethically appropriate), referencing such data using a unique identifier in the references and in the “Availability of Data and Materials” section of your manuscript.<br><br>Have you have met the above requirement as detailed in our Minimum Standards Reporting Checklist?	Yes

Powered by Editorial Manager® and ProduXion Manager® from Aries Systems Corporation

Manuscript Click here to download Manuscript Lynch_main-text-revision.pdf

HOMINID: A framework for identifying associations between host genetic variation and microbiome composition

Joshua Lynch[1,2,#], Karen Tang[1,2], Sambhawa Priya[1,2], Joanna Sands[1,2], Margaret Sands[1,2], Evan Tang[1,2], Sayan Mukherjee[3], Dan Knights[4,5,*], Ran Blekhman[1,2,*]

[1] Department of Genetics, Cell Biology, and Development, University of Minnesota, Minneapolis, MN, USA

[2] Department of Ecology, Evolution, and Behavior, University of Minnesota, Minneapolis, MN, USA

[3] Departments of Statistical Science, Mathematics, and Computer Science, Duke University, Durham, NC, USA

[4] Department of Computer Science and Engineering, University of Minnesota, Minneapolis, MN, USA

[5] Biotechnology Institute, University of Minnesota, Minneapolis, MN, USA

*To whom correspondence should be addressed: blekhman@umn.edu (RB), dknights@umn.edu (DK)

[#]Current affiliation: Department of Agricultural and Biosystems Engineering, University of Arizona, Tucson, AZ, USA

Keywords: microbiome, host genetics, association, machine learning

Abstract

Recent studies have uncovered a strong effect of host genetic variation on the composition of host-associated microbiota. Here, we present HOMINID, a computational approach based on Lasso linear regression, that given host genetic variation and microbiome composition data, identifies host SNPs that are correlated with microbial taxa abundances. Using simulated data we show that HOMINID has accuracy in identifying associated SNPs, and performs better compared to existing methods. We also show that HOMINID can accurately identify the microbial taxa that are correlated with associated SNPs. Lastly, by using HOMINID on real data of human genetic variation and microbiome composition, we identified 13 human SNPs in which genetic variation is correlated with microbiome taxonomic composition across body sites. In conclusion, HOMINID is a powerful method to detect host genetic variants linked to microbiome composition, and can facilitate discovery of mechanisms controlling host-microbiome interactions.

Availability and implementation

Software, code, tutorial, installation and setup details, and synthetic data are available in the project homepage: https://github.com/blekhmanlab/hominid.

Real dataset used here is from Blekhman et al. (Blekhman et al. 2015); 16S rRNA gene sequence data and OTU tables are available on the HMP DACC website (www.hmpdacc.org), and host genetic data are deposited in dbGaP under project number phs000228.

Background

The microbial communities found in and on the human body are influenced by multiple factors (Consortium, Human Microbiome Project 2012). In addition to the clear effect of environmental factors on the microbiome, there is growing support for an impact of host genetics (Goodrich, Davenport, Waters, et al. 2016; Morton et al. 2015). Several candidate gene studies have found correlation between human genetic variation and the structure of the microbiome (Tong et al. 2014; Khachatryan et al. 2008; Knights et al. 2014). In addition, genome-wide approaches can also be useful to identify human genetic impact on the microbiome (Goodrich et al. 2014; Blekhman et al. 2015; Goodrich, Davenport, Beaumont, et al. 2016; Davenport et al. 2015). For example, Goodrich et al. used hundreds of twin pairs to calculate the heritability of the gut microbiome, and identify bacterial taxa that are heritable, such as Christensenellaceae (Goodrich et al. 2014). Researchers have also utilized quantitative trait locus (QTL)-mapping approaches in the laboratory mouse and have identified multiple loci associated with the structure of gut microbial communities, some of which overlap genes involved in immune response (Benson et al. 2010; Leamy et al. 2014). Moreover, studies have used joint human genetic variation and microbiome data to find associations between loci in the human genome and microbial taxa (Blekhman et al. 2015; Davenport et al. 2015; Bonder et al. 2016; Turpin et al. 2016). In our recent study, in addition to showing that human genetic variation is associated with the structure of microbial communities across ten body sites, we have identified human single nucleotide polymorphisms (SNPs) associated with variation in the microbiome, and found that these loci are highly enriched in immunity genes and pathways (Blekhman et al. 2015). This approach, which includes the joint analysis of host genetic variation (SNPs) and microbiome taxonomic composition data (usually an OTU table), has the important advantage of identifying specific host genes and pathways that may control the microbiome, thus shedding light on the biological mechanisms of host-microbiome interaction, and pinpointing potential disease-causing pathways. However, this analysis is complicated by the fact that the microbiome contains many taxa that can be used as potential molecular complex traits in the GWAS analysis. Testing many

taxa reduces the power and multiple hypothesis testing correction makes the identification of associations challenging.

Here, we propose a framework for identifying host SNPs associated with microbiome composition using Lasso regression, named **HOMINID** (**Ho**st-**M**icrobiome **In**teraction **Id**entification; see **Figure 1** and Supplementary Information). Our method has several advantages: (1) it takes as input host genetic variation data (in a modified VCF format) and microbiome composition data (as an OTU table), to facilitate a simple analysis pipeline with no need to make new data formats; (2) HOMINID uses Lasso regression, which is specifically designed for cases where a relatively small number of taxa are correlated with host SNP genotype, as opposed to existing methods that use all taxa abundances; and (3) HOMINID uses stability selection with randomized Lasso to identify the specific microbial taxa that are correlated with each associated SNP.

Materials and Methods

HOMINID implementation. We implemented Lasso regression with the taxon relative abundances (arcsin sqrt transformed) as predictors and genetic variation at each SNP as response, for the purpose of identifying an additive effect between host genotype and microbiome features (see Supplementary Information and Figures S1-S3). In most situations, we expect at most a few taxa's abundances to correlate with a SNP, therefore ordinary least-squares (OLS) regression, which includes all taxa abundances as predictor variables, might not be an appropriate model. Instead, we need a regression algorithm that selects only the few predictors (taxa) that correlate to host genetics and discards the rest. The Lasso linear regression model used for HOMINID is similar to OLS regression, except that it includes an additional penalty term that shrinks most regression coefficients to zero, resulting in a sparse solution; thus it predicts only a few taxa to correlate with the host genetics. The Lasso regression was implemented using the Python (version 2.7/3.5+) machine-learning library scikit-learn (Pedregosa et al. 2011), with microbiome relative abundances as predictors and SNP genotype as response variable. The penalty term was

tuned via a five-fold cross-validation. How well the host genetics correlates with the microbiome is measured with the coefficient of determination, R^2_L. R^2_L is the median R^2 from five-fold cross-validation, with 100-times resampling. Also outputted are 95th percentile bootstrap confidence intervals from 10,000 bootstrap samples. Detailed description of the implementation of Lasso regression is available in the Supplementary Information.

Identifying correlated SNPs and taxa. To identify SNPs that are predicted correlated to the microbiome (prediction positive) from the uncorrelated (prediction negative) HOMINID uses a q-value cutoff, which puts an upper bound on the False Discovery Rate (FDR). A cutoff value, R^2_c, of R^2_L is chosen such that the q-value, $q(R^2_c)$, is equal to 0.1. A given SNP is predicted positive (predicted correlated to the microbiome) if $R^2_L \geq R^2_c$. $q(R^2_c)$ is determined by a permutation test, whereby for each SNP the sample labels are shuffled and Lasso regression is rerun ten times. $q(R^2_c)$ is defined as the fraction of permuted SNPs predicted positive divided by the fraction of unpermuted SNPs predicted positive (Subramanian et al. 2005). R^2_c is chosen such that $q(R^2_c) = 0.1$. The taxa that are most strongly associated with a SNP are identified using Stability Selection with randomized Lasso (Meinshausen and Bühlmann 2010). Briefly, stability selection perturbs the regression coefficients and the penalty term in the Lasso regression, and then reruns the regression thousands of times. If the same predictors (taxa) are repeatedly selected, even when the odds are against them, then they are robust predictors. Full details on this procedure are available in the Supplementary Information.

Controlling for other (non-taxon) covariates. HOMINID allows for controlling for any additional covariates (other than the microbiome) by including the covariates in the microbiome taxonomic table. This enables controlling for potentially confounding factors, such as individual age and sex. It also enables controlling for ancestry (or population substructure) by including the principal components (PCs) of the genetic variation data (Price et al. 2006; Pritchard et al. 2000). in the analysis. We performed two analyses, one including host genetic PCs as covariates (results in Supplementary Table S1), and one without these covariates (Supplementary Table S2). We excluded from the results SNPs for which there is a strong correlation with sex.

Synthetic datasets. To test the performance of HOMINID we generated several synthetic datasets. "Taxon" absolute abundances ("counts") were drawn from a log-series distribution. The log-series distribution is frequently used to represent species abundances (see, e.g., (Baldridge et al. 2016)), and it allows a range of abundances that spans several orders of magnitude, mimicking both rare and abundant taxa. Often in real abundance tables a large fraction of taxa have an abundance of zero (taxon either not present or not detected). The log-series abundance tables also had this quality; in our synthetic data, 21% of abundances are count zero. Synthetic SNP data were generated such that, for each SNP, N_{ctc} random taxa's abundances correlate with that SNP's genotype. Uncorrelated SNPs were created by permuting the sample IDs, preserving the minor allele frequency. Effect size was varied by adding "noise" to the SNP genotype data. Once the SNP and taxon-abundance data were generated, a measure of the effect size was calculated: the coefficient of determination, R^2_{OLS}, for an ordinary least square (OLS) multiple regression between the correlated taxa's abundances and the SNP genotype. Since R^2_{OLS} is a characteristic of the input data before analysis by HOMINID, we call it the "input R^2" to distinguish it from the R^2 output by the HOMINID Lasso regression (aka the "output R^2" or R^2_L). To examine data sets with smaller effect sizes, "noise" was added to the SNP data by swapping the genotypes of pairs of samples, reducing the correlation between the N_{ctc} correlated taxa and the host SNP genotype. Several data sets were created with progressively more "noise", until $R^2_{OLS} \rightarrow 0$. We created three sets of synthetic data to examine the performance of HOMINID on different qualities of the input data: Data set MAF varies the minor allele frequency, with MAF ranging from 0.10 to 0.50; data set CTC varies the number of correlated taxa from five to twenty; and data set TC varies the total number of taxa in the taxon table from 100 to 500. All data sets contain 500 SNPs each. Data in sets MAF and CTC comprise 1000 individuals; data sets in set TC contain 100 individuals. Data sets MAF and TC all have three correlated taxa per SNP. The MAF for data sets CTC and TC is 0.30.

Human Microbiome Project data. In addition to the synthetic datasets described above, we also tested our method on a real dataset that includes both human genetic and microbiome data (Blekhman et al. 2015). This dataset includes 93 individuals for whom microbiome was profiled as part of the Human Microbiome Project, and for which host genetic variation

information was extracted from shotgun metagenomics sequence data as described previously (Blekhman et al. 2015). We annotated the previously described set of 4.2 million high-quality single nucleotide polymorphisms (SNPs) using ANNOVAR (Wang, Li, and Hakonarson 2010) and focused the analysis on a set of 32,696 protein-coding SNPs. We further filtered this set to include only SNPs with minor allele frequency of at least 20% and SNPs for which we had data for at least 50 individuals. The number of SNPs actually tested varies across body sites, ranging from 12,400 to 14,651 SNPs, with a mean of 14,023. For the Stool microbiome data, which included 107 total taxa, running HOMINID on 14,469 SNPs using 12-core Intel Xeon E5-2680 2.50 GHz processors took 16 cpu hours.

Comparison to other methods. The PERMANOVA (Anderson 2001; McArdle and Anderson 2001) analysis was done in R with the adonis function in the vegan (Oksanen et al. 2007) package. The model formula has the SNP genotype as numeric (not factor) predictor variables and the arcsin-sqrt transformed taxon relative abundance table as response variable. The method used to calculate pairwise "distances" was the default Bray-Curtis. The MiRKAT (Zhao et al. 2015) analysis was performed using the MiRKAT package in R. The Bray-Curtis dissimilarity matrix was computed on the arcsin-sqrt transformed taxon table. The matrix was then converted to a kernel matrix, and MiRKAT invoked for each SNP. Since both PERMANOVA and MiRKAT output p-values as measures of how well the taxon abundances correlate with each SNP's genotype (whereas HOMINID outputs R^2_L values) we chose a cutoff value of p-value such that $q(p_c) = 0.1$ to separate the prediction positives (correlated) from the prediction negatives (uncorrelated), much in the same way we chose the cutoff R^2_c to separate prediction positive/negative such that $q(R^2_c) = 0.1$ for the Lasso regression.

Results

Analysis using synthetic data. To assess HOMINID's performance, we first used the pipeline on a comprehensive set of synthetic datasets (described above and in the Supplementary Information). These datasets were designed to simulate variation in several important factors,

such as variation of the strength of correlation (the input R^2) of the associated SNP with microbiome composition, variation in minor allele frequency (MAF) of the associated SNP, noise level in microbiome data, and the number of taxa associated with the SNP. After analyzing each of the datasets we calculated and plotted the method's sensitivity, specificity, precision, negative predictive value (NPV), false positive rate (FPR), false negative rate (FNR), false discovery rate (FDR), and accuracy, as a function of the input R^2, highlighting the effects of the variable factors above (see **Figures 2A-D**, Supplementary Information and Supplementary Figures S4 - S43).

We found that the strength of correlation (input R^2) between SNP genotype and the correlated taxa has little effect on HOMINID's ability to identify the SNP, unless the correlation is very low (**Figures 2A** and **2B**, Supplementary Information, and Supplementary Figures S4 - S11). HOMINID achieved high sensitivity and specificity for R^2 values of above ~ 0.05. The False Discovery Rate (FDR) is below 0.1 by design, and variation in FDR is due to imprecision (finite number of significant digits) in calculation of $R^2_{L,}$ and therefore imprecision in calculation of q. (**Figures 2C** and **2D)**. Similarly, variation in MAF does not affect HOMINID's sensitivity, as data sets with different MAF follow the same behavior (**Figure 2B**).

One of HOMINID's unique features is the ability to identify the taxa that are correlated with an associated SNP. We found that this prediction performs well, with accuracy approaching 1 and a false positive rate (FPR) of 0 for input R^2 values larger than about 0.1, but drops off at lower R^2 values (**Figures 2E** and **2F**, Supplementary Figures S26 and S27). The number of correlated taxa had a noticeable effect, whereby SNPs that correlated with more taxa had higher FPR (compare **Figure 2E** with **Figure 2F**), although in all test datasets' FPR remained < 0.07.

Comparison to other methods. In order to assess HOMINID's performance, we compared it to PERMANOVA (Anderson 2001; McArdle and Anderson 2001) and MiRKAT (Zhao et al. 2015), two platforms that can be used to identify host SNPs associated with microbiome composition. We note that HOMINID has a unique feature allowing it to identify the specific microbial taxa associated with each SNP. Since other approaches lack this option, the comparison centered around the ability to detect SNPs that are correlated with the microbiome,

and not on the detection of correlated taxa. Our analysis included input datasets with various input R^2 values and noise levels (various effect sizes), and compared the sensitivity of each method to detect the associated SNPs. We found that for median input R^2 values (correlation between associated SNP and microbiome composition) of about 0.15 or above the three methods are all highly sensitive (**Figure 3**). However, for lower input R^2 values, HOMINID is more sensitive. Specifically, for the data set with median input $R^2 = 0.08$ HOMINID's sensitivity is 1, while the sensitivity of MiRKAT and PERMANOVA is 0.19 and 0.29, respectively (**Figure 3**). Similarly, for median input $R^2 = 0.03$ HOMINID's sensitivity is 0.46, while the other methods' sensitivities are 0.

Human Microbiome Project data. We ran the HOMINID pipeline on a previously published data of microbiome and host genetic variation from the Human Microbiome Project cohort (Blekhman et al. 2015). We focused our analysis on coding SNPs with minor allele frequency ≥ 0.2, and identified SNPs for which permutation-based q-value ≤ 0.1 and the 95th percentile confidence interval for R^2 does not include zero. To account for population substructure, we ran a second analysis including the genetic principal components (PCs) as additional covariates (Price et al. 2006; Pritchard et al. 2000). This resulted in the identification of 11 (regression with genetic PCs as covariates) and 6 (regression without genetic PCs) for a total of 13 unique associations between host SNP and microbiome composition across 15 body sites (see Supplementary Tables S1 and S2, respectively). As can be seen in Figure 4, HOMINID is able to detect SNPs with the expected pattern of association between host genetic variation and the microbiome. For example, for SNP rs2297345 in the gene *PAK7* we detected a correlation between genotype and a single microbial taxon, Propionibacteriaceae (**Figure 4A**). HOMINID can also detect SNPs where multiple taxa are correlated with the same SNP (e.g., SNP rs6032 in **Figure 4B**), as well as more complex patterns of association; for example, for SNP rs230898 in the gene *TEKT3* (**Figure 4C**) genetic variation is positively correlated with one taxon (Clostridia) and negatively with others (Rhodocyclales and Aerococcaceae).

Although HOMINID performs strongly on the data used in this paper, there are several potential limitations to our method. First, since it is especially designed to identify SNPs where a

number of taxa are associated, it might not be optimal for cases where there is a dramatic shift in the microbiome that includes many dozens of taxa. Moreover, since the SNP is used as the response in the HOMINID model, it is difficult to identify epistatic effects, whereby genetic variation in two or more loci interact to affect microbiome composition. Although HOMINID could still be used to detect these interactions, by including all genotype combinations as response variables; however, multiple hypothesis testing could be an issue, especially for microbiome association studies, where samples sizes are currently small relative to GWAS of other complex traits. Nevertheless, HOMINID might be useful for detection of interaction of between candidate loci.

Lastly, we developed a web-based tool for the visualization of host-microbiome interaction network identified in HOMINID, available at http://z.umn.edu/genemicrobe. The website, designed using D3.js with a dedicated MySQL database serving as the back-end, displays a dynamic visualization of host gene-microbiome taxa interaction networks, and allows the user to add and remove nodes (host gene and microbial taxa), adjust the display size and node locations, filter by body sites, and generate figures. Currently, the website includes toy data representing all SNP-microbe associations with a nominal p-value <= 0.1 in the Human Microbiome Project data described above. We believe that as studies using larger sample sizes materialize (for example, a recent study included 1,514 subjects (Bonder et al. 2016)), we expect this tool to be useful for visualization of much larger number of associations.

Conclusions

We present HOMINID, a framework designed for identifying associations between host genetic variation and microbiome composition. We analyze synthetic data to show HOMINID's overall strong performance, identify specific factors that may affect it, highlight HOMINID's unique features, and show HOMINID's utility with a real dataset. We expect that HOMINID would be useful for studies attempting to characterize the genetic basis of host-microbiome interactions.

Funding

This work is supported in part by funds from the University of Minnesota College of Biological Sciences, The Randy Shaver Cancer Research and Community Fund, Institutional Research Grant #124166-IRG-58-001-55-IRG53 from the American Cancer Society, and a Research Fellowship from The Alfred P. Sloan Foundation. This work was facilitated in part by computational resources provided by the Minnesota Supercomputing Institute.

Figure Legends

Figure 1. Illustration of the HOMINID pipeline

Figure 2. Assessment of HOMINID's performance using synthetic data. Panels **A-D** assess how well HOMINID predicts the SNPs whose genotypes correlate with microbiome abundances, and panels **E** and **F** assess how well HOMINID predicts the specific taxa correlated with an associated SNP. **(A)** Sensitivity as a function of effect size (input R^2) for the data sets with MAF=0.30. Different colored points and boxplots represent data sets with different noise levels and therefore different effect sizes. (**B**) Same as A with variation in input data MAF values represented by different colored boxplots. (**C)** FDR as a function of effect size (input R^2) for data sets with just MAF=0.30. (**D**) Same as C with variation in input MAF values represented by different colored boxplots. **(E)** FPR for the stability selection step (identifying the taxa that associate with a SNP's genotype), as a function of effect size (input R^2) for data sets with three correlated taxa. (**F**) Same as E but with twenty correlated taxa.

Figure 3. Comparison of the performance of HOMINID versus MiRKAT and PERMANOVA. Sensitivity is plotted as a function of effect size (input R^2) for HOMINID (red), MirKAT (green), and PERMANOVA (blue). At high input R^2 all three methods perform

well, finding all SNPs that correlate with the microbiome. However, at smaller effect sizes (lower input R^2), HOMINID is more sensitive.

Figure 4. Examples of SNPs where correlations were found between host genetic variation and the microbiome. Three SNPs are shown: rs2297345 (correlated with abundance of microbial taxa in the right antecubital fossa), rs6032 correlated with abundance of microbial taxa in the throat), and rs230898 (correlated with abundance of microbial taxa in the supragingival plaque). The x-axis shows the host SNP genotypes, and the y-axis shows the arcsin sqrt transformed taxon abundances. The different correlated taxa for each SNP are shown in different colors.

References

Anderson, Marti J. 2001. "A New Method for Non-Parametric Multivariate Analysis of Variance." *Austral Ecology* 26 (1). Wiley Online Library: 32–46.

Baldridge, Elita, David J. Harris, Xiao Xiao, and Ethan P. White. 2016. "An Extensive Comparison of Species-Abundance Distribution Models." *PeerJ* 4 (December): e2823.

Benson, Andrew K., Scott A. Kelly, Ryan Legge, Fangrui Ma, Soo Jen Low, Jaehyoung Kim, Min Zhang, et al. 2010. "Individuality in Gut Microbiota Composition Is a Complex Polygenic Trait Shaped by Multiple Environmental and Host Genetic Factors." *Proceedings of the National Academy of Sciences of the United States of America* 107 (44): 18933–38.

Blekhman, Ran, Julia K. Goodrich, Katherine Huang, Qi Sun, Robert Bukowski, Jordana T. Bell, Timothy D. Spector, et al. 2015. "Host Genetic Variation Impacts Microbiome Composition across Human Body Sites." *Genome Biology* 16 (September): 191.

Bonder, Marc Jan, Alexander Kurilshikov, Ettje F. Tigchelaar, Zlatan Mujagic, Floris Imhann, Arnau Vich Vila, Patrick Deelen, et al. 2016. "The Effect of Host Genetics on the Gut Microbiome." *Nature Genetics*, October. Nature Research. doi:10.1038/ng.3663.

Consortium, Human Microbiome Project. 2012. "Structure, Function and Diversity of the Healthy Human Microbiome." *Nature* 486: 207–14.

Davenport, Emily R., Darren A. Cusanovich, Katelyn Michelini, Luis B. Barreiro, Carole Ober, and Yoav Gilad. 2015. "Genome-Wide Association Studies of the Human Gut Microbiota." *PloS One* 10 (10). dx.plos.org: e0140301.

Goodrich, Julia K., Emily R. Davenport, Michelle Beaumont, Matthew A. Jackson, Rob Knight, Carole Ober, Tim D. Spector, Jordana T. Bell, Andrew G. Clark, and Ruth E. Ley. 2016. "Genetic Determinants of the Gut Microbiome in UK Twins." *Cell Host & Microbe* 19 (5): 731–43.

Goodrich, Julia K., Emily R. Davenport, Jillian L. Waters, Andrew G. Clark, and Ruth E. Ley. 2016. "Cross-Species Comparisons of Host Genetic Associations with the Microbiome." *Science* 352 (6285): 532–35.

Goodrich, Julia K., Jillian L. Waters, Angela C. Poole, Jessica L. Sutter, Omry Koren, Ran Blekhman, Michelle Beaumont, et al. 2014. "Human Genetics Shape the Gut Microbiome." *Cell* 159 (4): 789–99.

Khachatryan, Zaruhi A., Zhanna A. Ktsoyan, Gayane P. Manukyan, Denise Kelly, Karine A. Ghazaryan, and Rustam I. Aminov. 2008. "Predominant Role of Host Genetics in Controlling the Composition of Gut Microbiota." *PloS One* 3 (8): e3064.

Knights, Dan, Mark S. Silverberg, Rinse K. Weersma, Dirk Gevers, Gerard Dijkstra, Hailiang Huang, Andrea D. Tyler, et al. 2014. "Complex Host Genetics Influence the Microbiome in Inflammatory Bowel Disease." *Genome Medicine* 6 (12): 107.

Leamy, Larry J., Scott A. Kelly, Joseph Nietfeldt, Ryan M. Legge, Fangrui Ma, Kunjie Hua, Rohita Sinha, et al. 2014. "Host Genetics and Diet, but Not Immunoglobulin A Expression, Converge to Shape Compositional Features of the Gut Microbiome in an Advanced Intercross Population of Mice." *Genome Biology* 15 (12): 552.

McArdle, Brian H., and Marti J. Anderson. 2001. "Fitting Multivariate Models to Community Data: A Comment on Distance-Based Redundancy Analysis." *Ecology* 82 (1). Wiley Online Library: 290–97.

Meinshausen, Nicolai, and Peter Bühlmann. 2010. “Stability Selection.” *Journal of the Royal Statistical Society. Series B, Statistical Methodology* 72 (4). Blackwell Publishing Ltd: 417–73.

Morton, Elise R., Joshua Lynch, Alain Froment, Sophie Lafosse, Evelyne Heyer, Molly Przeworski, Ran Blekhman, and Laure Ségurel. 2015. “Variation in Rural African Gut Microbiota Is Strongly Correlated with Colonization by Entamoeba and Subsistence.” *PLoS Genetics* 11 (11): e1005658.

Oksanen, Jari, Roeland Kindt, Pierre Legendre, Bob O’Hara, M. Henry H. Stevens, Maintainer Jari Oksanen, and Mass Suggests. 2007. “The Vegan Package.” *Community Ecology Package* 10: 631–37.

Pedregosa, Fabian, Gaël Varoquaux, Alexandre Gramfort, Vincent Michel, Bertrand Thirion, Olivier Grisel, Mathieu Blondel, et al. 2011. “Scikit-Learn: Machine Learning in Python.” *Journal of Machine Learning Research: JMLR* 12 (Oct): 2825–30.

Price, Alkes L., Nick J. Patterson, Robert M. Plenge, Michael E. Weinblatt, Nancy A. Shadick, and David Reich. 2006. “Principal Components Analysis Corrects for Stratification in Genome-Wide Association Studies.” *Nature Genetics* 38 (8): 904–9.

Pritchard, J. K., M. Stephens, N. A. Rosenberg, and P. Donnelly. 2000. “Association Mapping in Structured Populations.” *American Journal of Human Genetics* 67 (1): 170–81.

Subramanian, Aravind, Pablo Tamayo, Vamsi K. Mootha, Sayan Mukherjee, Benjamin L. Ebert, Michael A. Gillette, Amanda Paulovich, et al. 2005. “Gene Set Enrichment Analysis: A Knowledge-Based Approach for Interpreting Genome-Wide Expression Profiles.” *Proceedings of the National Academy of Sciences of the United States of America* 102 (43): 15545–50.

Tong, Maomeng, Tong Maomeng, Mchardy Ian, Ruegger Paul, Goudarzi Maryam, Purna C. Kashyap, Haritunians Talin, et al. 2014. “Reprograming of Gut Microbiome Energy Metabolism by the FUT2 Crohn’s Disease Risk Polymorphism.” *The ISME Journal* 8 (11): 2193–2206.

Turpin, Williams, Osvaldo Espin-Garcia, Wei Xu, Mark S. Silverberg, David Kevans, Michelle I. Smith, David S. Guttman, et al. 2016. “Association of Host Genome with Intestinal Microbial Composition in a Large Healthy Cohort.” *Nature Genetics* 48 (11): 1413–17.

Wang, Kai, Mingyao Li, and Hakon Hakonarson. 2010. “ANNOVAR: Functional Annotation of Genetic Variants from High-Throughput Sequencing Data.” *Nucleic Acids Research* 38 (16): e164.

Zhao, Ni, Jun Chen, Ian M. Carroll, Tamar Ringel-Kulka, Michael P. Epstein, Hua Zhou, Jin J. Zhou, Yehuda Ringel, Hongzhe Li, and Michael C. Wu. 2015. “Testing in Microbiome-Profiling Studies with MiRKAT, the Microbiome Regression-Based Kernel Association Test.” *American Journal of Human Genetics* 96 (5): 797–807.

Click here to download Figure fig1.pdf

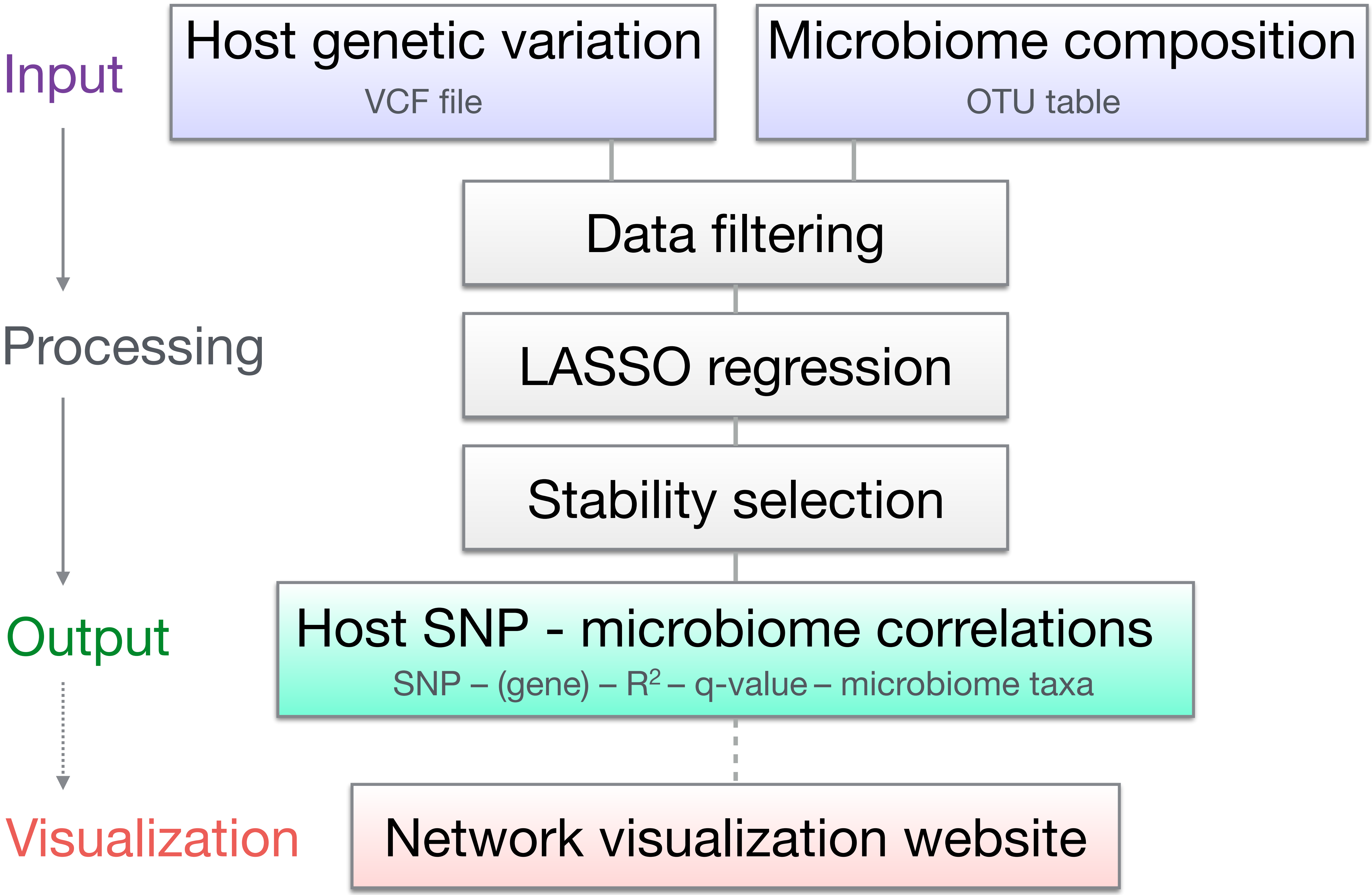

Figure 2

Click here to download Figure fig2.pdf

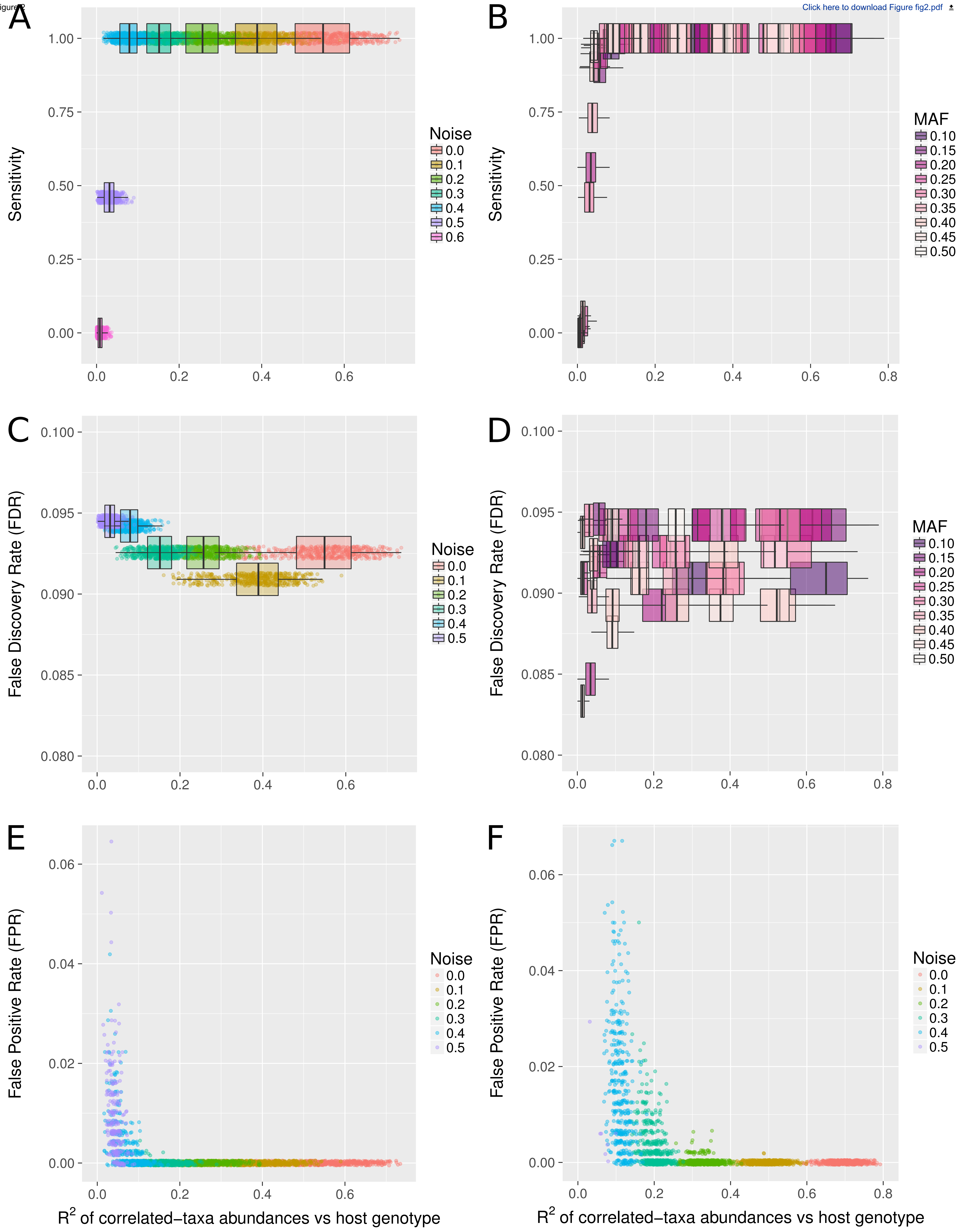

Figure 3

Click here to download Figure fig3.pdf

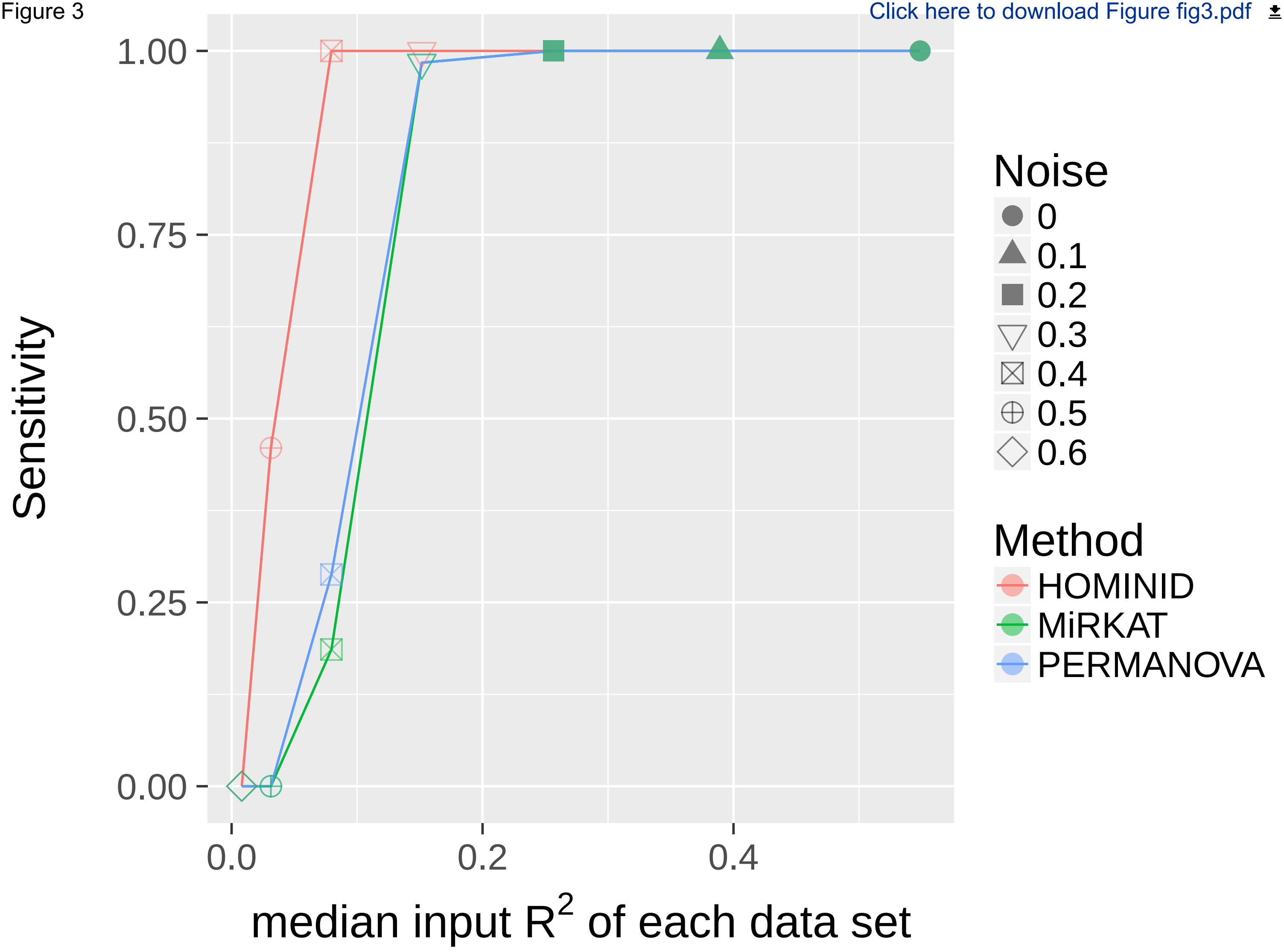

Figure 4

Click here to download Figure fig4.pdf

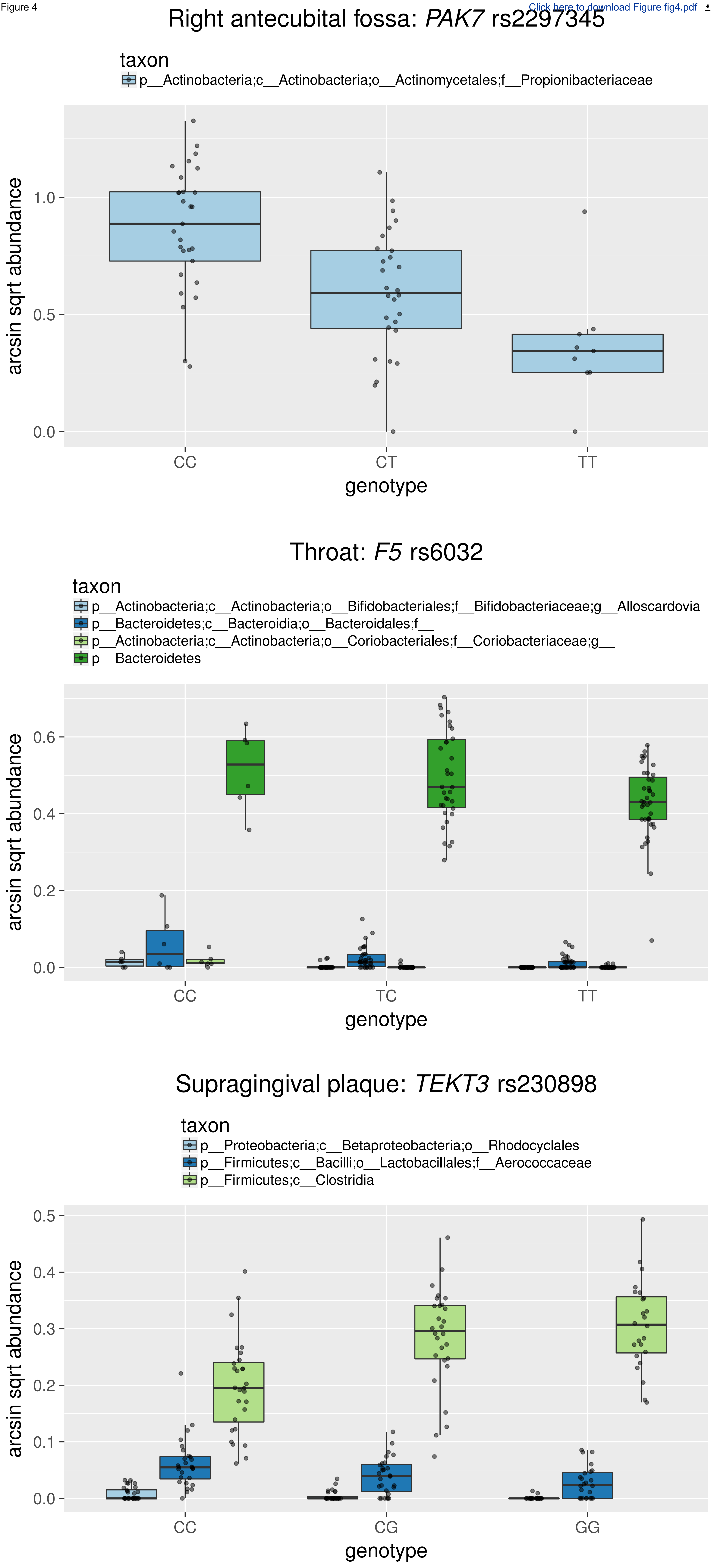

Supplemental Table S1

Click here to access/download
Supplementary Material
supplementalTableS1.xlsx

Supplemental Table S2

Click here to access/download
Supplementary Material
supplementalTableS2.xlsx

Click here to access/download
Supplementary Material
supplementaryInformation.pdf

Cover Letter + Response to Reviewers

Click here to download Personal Cover
Lynch_et_al_2017_Cover_Letter-

University of Minnesota

Ran Blekhman, Ph.D.
Assistant Professor

Genetics, Cell Biology, and Development
Ecology, Evolution, and Behavior

MCB 6-126
321 Church St. SE
Minneapolis, MN 55455
Email: blekhman@umn.edu
Tel: (612) 624-4092
Web: BlekhmanLab.org

July 15, 2017
Nicole Nogoy, Ph.D.
Editor
GigaScience

Dear Dr. Nogoy,

I would like to thank you for the thoughtful review of our manuscript, "**HOMINID: A framework for identifying associations between host genetic variation and microbiome composition**" (GIGA-D-16-00138). We agree with the criticism and the concerns raised by the reviewers. We have now prepared a comprehensively revised (and in our opinion, much improved) version of our manuscript, thoroughly addressing the comments from reviewers. We have included a complete point-by-point description of the modifications, attached to this document. In brief, the revised version now includes the following:

(1) A thorough evaluation of the performance of HOMINID using a comprehensive and more realistic set of synthetic data. We use a parameter sweep to evaluate the effect of various factors, including the correlation between host SNP and taxa abundances, host SNP minor allele frequency, the number of correlated taxa, and noise in microbiome data, and assess the effect on performance by calculating sensitivity, specificity, precision, negative predictive value, false positive rate, false negative rate, false discovery rate, and accuracy.

(2) We now include a comparison of HOMINID to other software, including PERMANOVA and MiRKAT.

(3) We have revised statistical procedure to correct for multiple tests, and now use false discovery rate to identify associated SNPs.

To our knowledge, HOMINID still represents the first software tool specifically designed for the purpose of mapping host genetic variants associated with microbiome composition. Since studies that attempt this analysis have become increasingly common, we believe that our paper would be of interest to the field, and that the HOMINID tool would be highly used by the microbiome research community.

Please do not hesitate to contact us if there are any remaining issues or concerns. We are excited to publish our work in *GigaScience*, and would like to thank you again for the positive review experience and helpful feedback that has substantially improved the paper.

Thank you in advance for your consideration, and we look forward to hearing from you soon.

Sincerely,

Ran Blekhman, Ph.D.
Alfred P. Sloan Research Fellow
Assistant Professor
University of Minnesota, Twin Cities
Dept. of Genetics, Cell Biology, and Development | Dept. of Ecology, Evolution, and Behavior
Cargill 222, 1500 Gortner Ave., St. Paul, MN 55108
BlekhmanLab.org | Twitter: @blekhman | Phone: (612) 624-4092 | Fax:(612) 624-6264

HOMINID: A framework for identifying associations between host genetic variation and microbiome composition

Response to Reviewers - GigaScience manuscript GIGA-D-16-00138

<u>Reviewer 1</u>

In this manuscript, the authors presented a novel approach for identification of host genetic variants that are associated with microbiome composition based on a machine learning approach. An online tool for visualization of the associations has also been provided to facilitate the presentation and interpretation of the findings. The strength of their method is the achievement of both overall association tests and variable selection using lasso and stability selection. However, the major concern is the lack of rigor in statistics and weakness of the real data example. The current criteria for identifying "significant" SNPs without multiple testing correction might result in too many false positives and have an undesired impact on the field if it is widely adopted by the field. Overall, the procedure could be of potential interest to the community for future large-sample size studies if the authors stick to common statistical practice for high-dimensional data, use more realistic simulations, and compare to existing methods. I have the following specific comments:

We thank the reviewer for the positive comments, and completely agree with the critique. We have drastically enhanced the manuscript with more rigorous statistics, including an appropriate correction for multiple testing, highly strengthened simulation data, and include a comparison to existing methods. We believe our manuscript has much improved and appreciate the helpful comments; responses to specific points are below.

1. About the statistics.
(1) The traditional p-value concerns one single hypothesis test. The current "p-value" based on permutation is misleading, and it is, in fact, an estimate of "false positive rate". The author should refrain from using the term "p-value" if it is different from the traditional meaning.

This is an important point and we agree that using the term p-value is misleading. The manuscript and approach now only considers q-value and false discovery rate. Unsurprisingly, this has changed the results of the real data, which now includes 13 SNPs with FDR q-value < 0.1 (Supplementary Tables S1 and S2). This is described in the methods section in the main text (pasted below) and in greater detail in the Supplementary Information (page 4).

Page 5: Identifying correlated SNPs and taxa. To identify SNPs that are predicted correlated to the microbiome (prediction positive) from the uncorrelated (prediction negative) HOMINID uses a q-value cutoff, which puts an upper bound on the False Discovery Rate (FDR). A cutoff value, R^2_c, of R^2_L is chosen such that the q-value, $q(R^2_c)$, is equal to 0.1. A given SNP is predicted positive (predicted correlated to the microbiome) if $R^2_L \geq R^2_c$. $q(R^2_c)$ is determined by a permutation test, whereby for each SNP the sample labels are shuffled and Lasso regression is rerun ten times. $q(R^2_c)$ is defined as the fraction of permuted SNPs predicted positive divided by the fraction of unpermuted SNPs predicted positive. R^2_c is chosen such that $q(R^2_c) = 0.1$.

(2) For high-dimensional data sets, the importance of control for false positives can never be overstated. Or the field will be full of false positives. That is the reason why the statistical genetics field usually uses the most conservative Bonferroni correction for GWAS. The author should make clear that the identification of significant SNPs should correct for multiple testing. At least, q-values should be used to control the false discovery rate if family-wise error rate control is not used due to low statistical power. Raw p-values should never be used. I also suggest the author implement the permutation-based family-wise error rate control.

We agree and realize we made a mistake by including nominal p-values in the original version of the paper. We now control for false discovery rate and do not include raw p-values. We have also removed the word “significant” from the text. Moreover, our analysis now includes permutations to estimate FDR. Please see our response to the previous point, and the description in the Methods section (page 5; text pasted above) and in the Supplementary Information (page 4).

2. About the simulations.

(1) Excessive zeros may be generated to mimics real microbiota data by using an overdispersed count model. The performance of linear models may be very sensitive to a large number of 0's or a large number of identical values after the proposed transformation. The data generated by the current exponential distribution could become very normal after arcsin sqrt transformation, which may not reflect the real data.

We agree and have expanded the synthetic data analysis to address this and the following points regarding the simulations. As suggested by the reviewer, all simulated data is now generated using an overdispersed count model, which better mimics the zero-inflated distribution of real microbiome relative abundance data. Specifically, across all synthetic datasets 21% of taxa have zero counts. This is described on Page 5 in the main text Methods section (pasted below), and in greater detail in the Supplementary Information.

Page 5: Synthetic datasets. To test the performance of HOMINID we generated several synthetic datasets. "Taxon" absolute abundances ("counts") were drawn from a log-series distribution. The log-series distribution is frequently used to represent species abundances (see, e.g., (Baldridge et al. 2016)), and it allows a range of abundances that spans several orders of magnitude, mimicking both rare and abundant taxa. Often in real abundance tables a large fraction of taxa have an abundance of zero (taxon either not present or not detected). The log-series abundance tables also had this quality; in our synthetic data, 21% of abundances are count zero.

(2) The simulated signal may be too strong. It is less likely that an SNP can have an R2 larger than 0.35. The human microbiome is highly variable and is strongly affected by numerous environmental factors such as diet. Based on existing epidemiological research, the R2 is usually very small (e.g. 2%). Thus much weaker correlation is expected. The sensitivity (a power of 0.95) is extraordinarily high.

This is an important point, and we have extended and improved our simulation to assess a wide variation in R2, as well as other factors, including variation in minor allele frequency (MAF) of the associated SNP, noise level in microbiome data, and the number of taxa associated with the SNP. To assess the performance of our approach, we calculated and plotted the method's sensitivity, specificity, precision, negative predictive value (NPV), false positive rate (FPR), false negative rate (FNR), false discovery rate (FDR), and accuracy, as a function of the input R2. This analysis is described in the Supplementary Information (pages 10 & 11) and the resulting plots are shown in Supplementary Figures S4 - S43. Specifically, our new analysis includes a large variation of R2 and shows that, as expected, R2 values have an effect on the performance of HOMINID. Indeed, lower effect sizes indeed produce a much lower sensitivity, as suggested by the reviewer. A summary of this analysis is provided in the text (page 8; copied below for convenience) and displayed in **Figure 2** (copied below as well):

Page 8: We found that the strength of correlation (input R^2) between SNP genotype and the correlated taxa has little effect on HOMINID's ability to identify the SNP, unless the correlation is very low (**Figs. 2A** and **2B**, Supplementary Information, and Supplementary Figures S4 - S11). HOMINID achieved high sensitivity and specificity for R^2 values of above ~ 0.05. The False Discovery Rate (FDR) is below 0.1 by design, and variation in FDR is due to imprecision (finite number of significant digits) in calculation of $R^2_{L,}$ and therefore imprecision in calculation of q. (**Figs. 2C** and **2D)**. Similarly, variation in MAF does not affect HOMINID's sensitivity, as data sets with different MAF follow the same behavior (**Fig. 2B**).

One of HOMINID's unique features is the ability to identify the taxa that are correlated with an associated SNP. We found that this prediction performs well, with accuracy approaching 1 and a false positive rate (FPR) of 0 for input R^2 values larger than about 0.1, but drops off at lower R^2 values (**Fig. 2E** and **Fig. 2F**, Supplementary Figures S26

and S27). The number of correlated taxa had a noticeable effect, whereby SNPs that correlated with more taxa had higher FPR (compare **Fig. 2E** with **Fig 2F:** see legend following figure below), although in all test datasets FPR remained < 0.07.

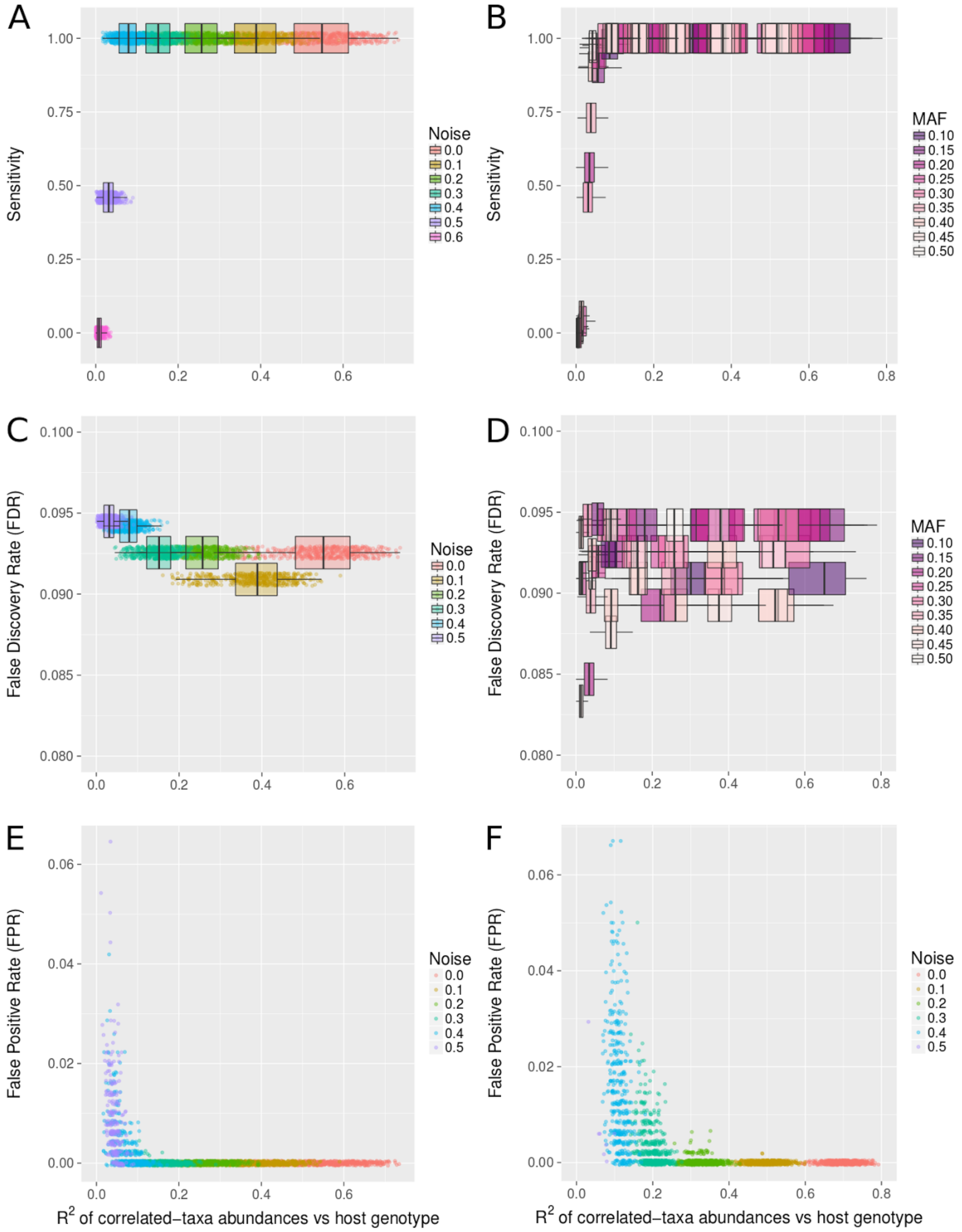

Figure 2 Legend. Assessment of HOMINID's performance using synthetic data. Panels **A-D** assess how well HOMINID predicts the SNPs whose genotypes correlate with microbiome abundances, and panels **E** and **F** assess how well HOMINID predicts the specific taxa correlated with an associated SNP. **(A)** Sensitivity as a function of effect size (input R^2) for the data sets with MAF=0.30. Different colored points and boxplots represent data sets with different noise levels and therefore different effect sizes. (**B**) Same as A with variation in input data MAF values represented by different colored boxplots. **(C)** FDR as a function of effect size (input R^2) for data sets with just MAF=0.30. (**D**) Same as C with variation in input MAF values represented by different colored boxplots. (**E)** FPR for the stability selection step (identifying the taxa that associate with a SNP's genotype), as a function of effect size (input R^2) for data sets with three correlated taxa. (**F**) Same as E but with twenty correlated taxa.

3. About the performance evaluation.
(1) The evaluation of the performance should be based on type I error and power if the proposed method is a statistical test. Sensitivity and specificity may not be the best measure when there is multiple testing. A specificity of 98% will translate to a very low precision. Suppose we test 10,000 SNPs with 5% signals, a sensitivity of 30% and a false positive rate (1 - specificity) of 2% will translate to ~200 false positives and 150 true positives. Thus the false discovery rate is more than 50%. In practice, the signal density and power may be much lower, and thus the majority may be false positives. Such high false positive rates are not acceptable, or the field will be full of false findings. The authors should tighten their criteria and use multiple testing correction procedure. The evaluation should be focused on the type I error control. For example, whether their method can control the false discovery rate at the nominal level using q-value approach. If not, cautions should be taken when applying the method.

We agree, and now include a comprehensive performance evaluation of the method. As described above, we performed a parameter sweep across several dimensions of synthetic datasets that vary in R2, MAF, and number of taxa, and assessed the effect on performance by calculating sensitivity, specificity, precision, negative predictive value (NPV), false positive rate (FPR), false negative rate (FNR), false discovery rate (FDR), and accuracy. This analysis is described in the Supplementary Information (page 4) and the resulting plots are shown in Supplementary Figures S4 - S43. Also, as described above, we delimit the prediction positive (SNP predicted correlated) from the prediction negative (SNP predicted uncorrelated) via q-value that puts an upper bound on the FDR, and no longer report nominal p-values. Our results show that FDR is lower than the q-value cutoff of 0.1 regardless of R2, noise, MAF, and the number of correlated taxa -- see Figure 2, pasted in the previous page, as well as the accompanying text.

(2) The proposed method has not been compared to more straightforward methods such as PERMANOVA or MiRKAT test for each SNP locus, coupled by multiple testing correction. The power evaluation may not be very meaningful without comparing to some existing simple methods. The current simulation strategy may favor their method due to the model sparsity and unclustered signals. However, even in such scenarios, compared to PERMANOVA or MiRKAT with non-tree-based distance such as Bray-Curtis or Euclidean distance on their transformed abundance data will be very informative. I recommend the authors to compare to existing methods on both simulated data sets and real data sets.

We now perform a comparison of our method with PERMANOVA and MiRKAT, as suggested by the reviewer. Our analysis shows that HOMINID, PERMANOVA, and MiRKAT perform equally well at R^2 about 0.15 or higher, but at lower effect sizes ($R^2 < 0.15$) HOMINID is more sensitive. The approach is described in the Methods section (page 7) and Supplementary Information (pages 63 & 64 and Supplementary Figures S49-S56). Results of this analysis are described in page 8 and visualized in Fig. 3 (which we paste below for convenience).

> **Page 8:** Comparison to other methods. In order to assess HOMINID's performance, we compared it to PERMANOVA (Anderson 2001; McArdle and Anderson 2001) and MiRKAT (Zhao et al. 2015), two platforms that can be used to identify host SNPs associated with microbiome composition. We note that HOMINID has a unique feature allowing it to identify the specific microbial taxa associated with each SNP. Since other approaches lack this option, the comparison centered around the ability to detect SNPs that are correlated with the microbiome, and not on the detection of correlated taxa. Our analysis included input datasets with various input R^2 values and noise levels (various effect sizes), and compared the sensitivity of each method to detect the associated SNPs. We found that for median input R^2 values (correlation between associated SNP and microbiome composition) of about 0.15 or above the three methods are all highly sensitive (**Fig. 3**). However, for lower input R^2 values, HOMINID is more sensitive. Specifically, for the data set with median input $R^2 = 0.08$ HOMINID's sensitivity is 1, while the sensitivity of MiRKAT and PERMANOVA is 0.19 and 0.29, respectively (**Fig. 3**). Similarly, for median input $R^2 = 0.03$ HOMINID's sensitivity is 0.46, while the other methods' sensitivities are 0.

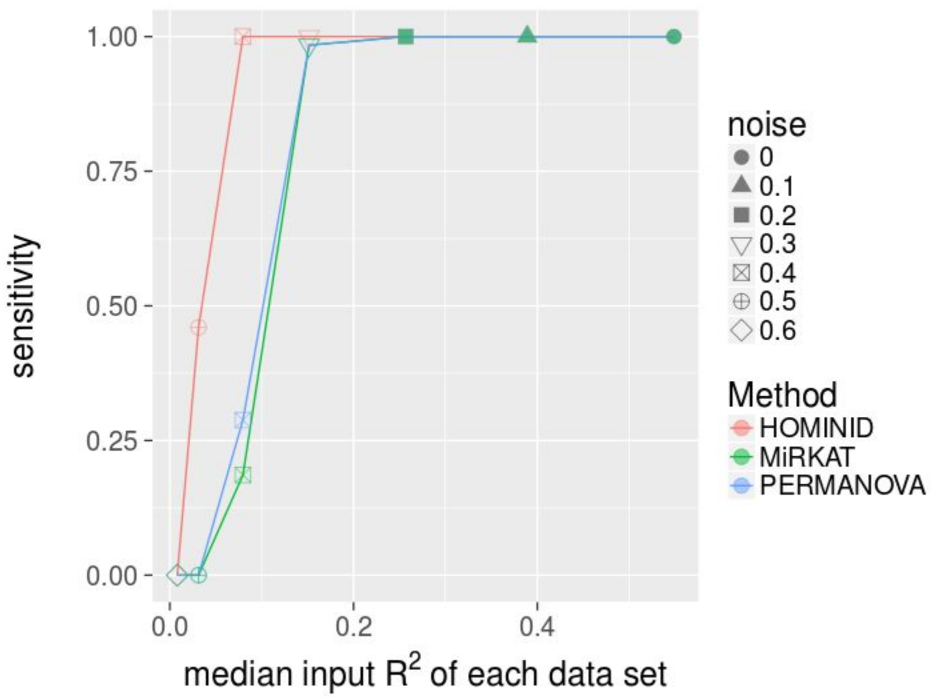

4. About the real data example.

(1) The real data demonstration is very week. The authors used the raw "p-value" to select the "significant" SNP, which clearly results in many false positives. Possibly, the majority of the SNPs may be false positives. It is based on the observation that if their "p-value" (false positive rate) cutoff of 0.01 is used, there will be on average 1% false positives and approximately 32,696 * 0.01=327 "false" SNPs identified for each body loci. Looking at the number of identified SNPs for different body sites, it is similar (100 - 200 SNPs) to the number of expected false positives.

We agree with this important point. As described above (see our response to point 1), we no longer use raw p-values, and instead use q-values to cap the FDR and only report SNPs at an FDR <= 0.1. The paragraphs in the main text that describe this analysis are copied and pasted below.

> **Page 6:** Human Microbiome Project data. In addition to the synthetic datasets described above, we also tested our method on a real dataset that includes both human genetic and microbiome data (Blekhman et al. 2015). This dataset includes 93 individuals for whom microbiome was profiled as part of the Human Microbiome Project, and for which host genetic variation information was extracted from shotgun metagenomics sequence data as described previously (Blekhman et al. 2015). We annotated the previously described set of 4.2 million high-quality single nucleotide polymorphisms (SNPs) using ANNOVAR (Wang, Li, and Hakonarson 2010) and focused the analysis on a set of 32,696 protein-coding SNPs. We further filtered this set to include only SNPs with minor allele

frequency of at least 20% and SNPs for which we had data for at least 50 individuals. The number of SNPs actually tested varies across body sites, ranging from 12400 to 14651 SNPs, with a mean of 14023.

Page 9: Human Microbiome Project data. We ran the HOMINID pipeline on a previously published data of microbiome and host genetic variation from the Human Microbiome Project cohort (Blekhman et al. 2015). We focused our analysis on coding SNPs with minor allele frequency ≥ 0.2, and identified SNPs for which permutation-based q-value ≤ 0.1 and the 95th percentile confidence interval for R^2 does not include zero. To account for population substructure, we ran a second analysis including the genetic principal components (PCs) as additional covariates (Price et al. 2006; Pritchard et al. 2000). This resulted in the identification of 11 (regression with genetic PCs as covariates) and 6 (regression without genetic PCs) for a total of 13 unique associations between host SNP and microbiome composition across 15 body sites (see Supplementary Tables S1 and S2, respectively). As can be seen in Figure 4, HOMINID is able to detect SNPs with the expected pattern of association between host genetic variation and the microbiome. For example, for SNP rs2297345 in the gene PAK3 we detected a correlation between genotype and a single microbial taxon, Propionibacteriaceae (**Fig 4A**). HOMINID can also detect SNPs where multiple taxa are correlated with the same SNP (e.g., SNP rs6032 in **Fig. 4B**), as well as more complex patterns of association; for example, for SNP rs230898 in the gene TEKT3 (**Fig 4C**) genetic variation is positively correlated with one taxon (Clostridia) and negatively with others (Rhodocyclales and Aerococcaceae).

(2) The number of genes that harbor SNPs that are correlated with the microbiome in more than one body site may also be a result of random noises. Since there are 15 body sites, there are 105 pairs. Assume that there is no real signal and that each body site identifies 1% significant genes randomly (using false positive rate of 0.01), there will be roughly1% * 1% * 105=1% genes show association with more than one body site. Suppose that we have 20,000 genes, and then it amounts to 200 genes, which is similar to the reported number.

After updating the analysis as described above, there are now 13 total SNPs that are correlated with microbiome composition at FDR of 0.1. The overlap across body sites is now small, with no SNP found in more than one body site, as expected. See the text above from the main text

(3) The authors might consider permuting the genotypes and rerun the same procedure. If they can demonstrate the "NULL" model produces way less number of "significant" hits, the results will be more convincing.

Our method now includes permutation for estimation of the q-value, as suggested by the reviewer. The description of this approach is brought in page 5:

Page 5: Identifying correlated SNPs and taxa. To identify SNPs that are predicted correlated to the microbiome (prediction positive) from the uncorrelated (prediction negative) HOMINID uses a q-value cutoff, which puts an upper bound on the False Discovery Rate (FDR). A cutoff value, R^2_c, of R^2_L is chosen such that the q-value, $q(R^2_c)$, is equal to 0.1. A given SNP is predicted positive (predicted correlated to the microbiome) if $R^2_L \geq R^2_c$. $q(R^2_c)$ is determined by a permutation test, whereby for each SNP the sample labels are shuffled and Lasso regression is rerun ten times. $q(R^2_c)$ is defined as the fraction of permuted SNPs predicted positive divided by the fraction of unpermuted SNPs predicted positive. R^2_c is chosen such that $q(R^2_c) = 0.1$.

(4) The author may consider making the real data available so others can reproduce their findings and compare to their method in the future.

All data and code used in this paper is available online. Here is the statement from the main text (page 2 under Availability and Implementation):

Page 2: Software, code, tutorial, installation and setup details, and synthetic data are available in the project homepage: https://github.com/blekhmanlab/hominid

Real dataset used here is from Blekhman et al., (Blekhman et al. 2015); 16S rRNA gene sequence data and OTU tables are available on the HMP DACC website (www.hmpdacc.org), and host genetic data are deposited in dbGaP under project number phs000228.

Minor

1. The author should state what q-value means and what type of error it controls. More detailed description of the procedure may be helpful. Some reference on the permutation-based false discovery control procedure may also be added.

This is now updated in the text:

Page 5: To identify SNPs that are predicted correlated to the microbiome (prediction positive) from the uncorrelated (prediction negative) HOMINID uses a q-value cutoff, which puts an upper bound on the False Discovery Rate (FDR). A cutoff value, R^2_c, of R^2_L is chosen such that the q-value, $q(R^2_c)$, is equal to 0.1. A given SNP is predicted positive (predicted correlated to the microbiome) if $R^2_L \geq R^2_c$. $q(R^2_c)$ is determined by a permutation test, whereby for each SNP the sample labels are shuffled and Lasso

regression is rerun ten times. $q(R^2_c)$ is defined as the fraction of permuted SNPs predicted positive divided by the fraction of unpermuted SNPs predicted positive. R^2_c is chosen such that $q(R^2_c) = 0.1$.

2. When using subsampling to calculate the median R2, the percentage of data used for test and training should be stated.

We used five-fold cross-validation. This is now mentioned in the main text:

Page 5: R^2_L is the median R^2 from five-fold cross-validation, with 100-times resampling.

3. Typo in "Supplementary data are available at Bioinformatics online."

This has been removed from the text.

4. "Taxon count" may change to "number of taxa" for better clarity.

We have changed "taxon count" to "number of taxa" throughout the text.

5. Computation time should also be mentioned in the manuscript.

This is now included in the manuscript:

Page 7 (Methods): For the Stool data with 107 total taxa in the taxon table, running HOMINID on 14,469 SNPs using Intel Xeon E5-2680 2.50 GHz processors took 16 cpu hours.

Reviewer #2: HOMINID: A framework for identifying associations between host genetic variation and microbiome composition

Reviewers: Katherine Pollard and Nandita Garud

Summary:
HOMINID is a Lasso regression method for identifying human polymorphisms associated with the species' composition in the human microbiome. The authors applied the method to the Human Microbiome Project data to identify several candidate host SNPs associated with different species, some of which are already implicated with roles in diabetes and immunity. The authors generate synthetic data to test three different parameters that may impact the method's

abilities: the allele frequency of the associated SNPs, the number of taxa associated with a SNP, and the total number of taxa present in a sample.

Positives:
With the extensive availability of microbiome data these days and the keen interest from the community to understand how human-associated microbiota impact human health, HOMINID is a valuable contribution to the community. Future work will certainly benefit from the framework laid out in this paper, especially if there are going to be extensions of this method for application to shotgun data, i.e. identifying associations between human SNPs and abundances of microbiome genes, and applications to other data sets. As it is in its current state, it is likely that HOMINID will be useful for scientists looking for associations of taxa with human SNPs without much further work. Finally, the online tool for searching and querying the results of this paper is easy to use, further enriching the resources available to the microbiome community.

We thank the reviewers for the positive comments, and appreciate the constructive critique. We have made significant changes to the the manuscript, which now includes much more rigorous statistics, highly enhanced simulation data and analysis, and include a comparison to existing methods. We believe our manuscript is much improved and appreciate the helpful comments; responses to specific points are below.

Major Critiques:

1. The main suggestion we have is that the authors did not sufficiently justify the Lasso method through a comparison to other models. They mention some of the other methods in the literature in the introduction, but the simulations only show performance of their method. We recommend some benchmarking against other models or at least a discussion of why they chose this method.

This is a critical point, and we agree with the reviewers. We have added a justification of the Lasso method; this is found in two places in the text:

> **Background, Page 4:** Here, we propose a framework for identifying host SNPs associated with microbiome composition using Lasso regression, named **HOMINID** (**Ho**st-**M**icrobiome **In**teraction **Id**entification; see **Figure 1** and **Supplementary Information**). Our method has several advantages: (1) it takes as input host genetic variation data (in a modified VCF format) and microbiome composition data (as an OTU table), to facilitate a simple analysis pipeline with no need to make new data formats; (2) HOMINID uses Lasso regression, which is specifically designed for cases where a relatively small number of taxa are correlated with host SNP genotype, as opposed to existing methods that use all taxa abundances; and (3) HOMINID uses stability selection

with randomized Lasso to identify the specific microbial taxa that are correlated with each associated SNP.

Methods, Page 4: We implemented Lasso regression with the taxon relative abundances (arcsin sqrt transformed) as predictors and genetic variation at each SNP as response, for the purpose of identifying an additive effect between allele count and microbiome features (see **Supplementary Information** and **Figures S1-S3**). In most situations, we expect at most a few taxa's abundances to correlate with a SNP, therefore ordinary least-squares (OLS) regression, which includes all taxa abundances as predictor variables, might not be an inappropriate model. Instead, we need a regression algorithm that selects only the few predictors (taxa) that correlate to host genetics and discards the rest. The Lasso linear regression model used for HOMINID is similar to OLS regression, except that it includes an additional penalty term that shrinks most regression coefficients to zero, resulting in a sparse solution; thus it predicts only a few taxa to correlate with the host genetics.

We have also added an analysis comparing HOMINID and two additional methods, PERMANOVA and MiRKAT, as also suggested by reviewer 1. Our analysis shows that HOMINID, PERMANOVA, and MiRKAT perform equally well at R^2 about 0.15 or higher, but at lower effect sizes ($R^2 < 0.15$) HOMINID is more sensitive. The approach is described in the Methods section (page 7) and Supplementary Information (page 63 & 64 and Supplementary Figures S49-56). Results of this analysis are described in page 8 and visualized in Fig. 3 (which we paste below for convenience).

Page 8: Comparison to other methods. In order to assess HOMINID's performance, we compared it to PERMANOVA (Anderson 2001; McArdle and Anderson 2001) and MiRKAT (Zhao et al. 2015), two platforms that can be used to identify host SNPs associated with microbiome composition. We note that HOMINID has a unique feature allowing it to identify the specific microbial taxa associated with each SNP. Since other approaches lack this option, the comparison centered around the ability to detect SNPs that are correlated with the microbiome, and not on the detection of correlated taxa. Our analysis included input datasets with various input R^2 values and noise levels (various effect sizes), and compared the sensitivity of each method to detect the associated SNPs. We found that for median input R^2 values (correlation between associated SNP and microbiome composition) of about 0.15 or above the three methods are all highly sensitive (**Fig. 3**). However, for lower input R^2 values, HOMINID is more sensitive. Specifically, for the data set with median input R^2 = 0.08 HOMINID's sensitivity is 1, while the sensitivity of MiRKAT and PERMANOVA is 0.19 and 0.29, respectively (**Fig. 3**). Similarly, for median input R^2 = 0.03 HOMINID's sensitivity is 0.46, while the other methods' sensitivities are 0.

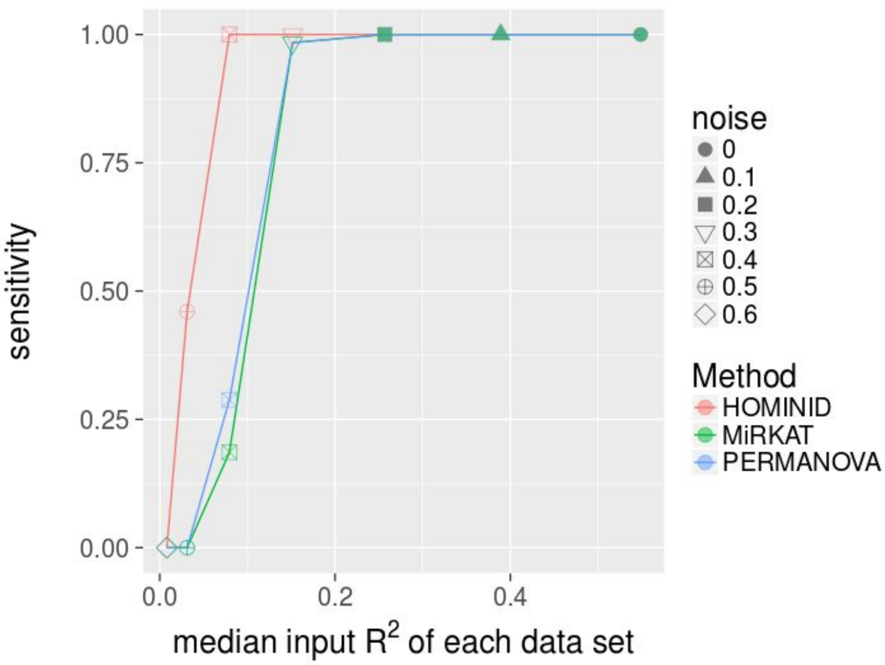

2. Population substructure: Blekhman et al. 2015 showed that the human polymorphisms most associated with microbiome composition were those that have the highest FST values across populations. How does HOMINID perform if there is population substructure? Does the number of false positives increase? A fourth simulation category exploring the effects of substructure would be helpful as most of the time human populations have complex demographic histories and hidden substructure and this may have an impact on results.

We agree with the reviewers on this issue. Population substructure is an important issue, and we have made several additions to the manuscript to address this. First, we included a description of how population structure can be accounted for in HOMINID. This is described in the Methods section (pasted below):

> **Methods, page 5:** Controlling for other (non-taxon) covariates. HOMINID allows for controlling for any additional covariates (other than the microbiome) by including the covariates in the microbiome taxonomic table. This enables controlling for potentially confounding factors, such as individual age and sex. It also enables controlling for ancestry (or population substructure) by including the principal components (PCs) of the genetic variation data (Price et al. 2006; Pritchard et al. 2000). in the analysis. We performed two analyses, one including host genetic PCs as covariates (results in Supplementary Table S1), and one without these covariates (Supplementary Table S2). We excluded from the results SNPs for which there is a strong correlation with sex.

In addition, to see whether substructure could affect the results, we used the fact that substructure likely exists in our real data. Using the approach described above, we performed two analyses with HOMINID, one controlling for population substructure (by including the host genetic principal components as covariates as described above) and one without controlling for population substructure. The results are We found that the number of SNPs identified (at a False Discovery Rate of 0.1) is slightly higher when host genetic PCs are accounted for, with 11 SNPs identified with PCs as covariates vs 6 without. Four SNPs overlap in the two lists. This suggests that accounting for population substructure -- as expected -- improves HOMINID's performance. This analysis is described in the text:

> **Results, page 9:** To account for population substructure, we ran a second analysis including the genetic principal components (PCs) as additional covariates (Price et al. 2006; Pritchard et al. 2000). This resulted in the identification of 11 (regression with genetic PCs as covariates) and 6 (regression without genetic PCs) for a total of 13 unique associations between host SNP and microbiome composition across 15 body sites (see Supplementary Tables S1 and S2, respectively).

3. Polygenic host effects on the microbiome: Many GWAS studies have the host SNPs as predictors rather than as response variables so that interactions (i.e. epistasis) between SNPs can be tested. It doesn't seem possible to test for epistasis in the author's framework with species as the predictors, yet it seems to be a very plausible that there would be epistatic effects. Could the authors comment on this limitation of their method in the main text as well explain as how testing for epistasis along with multiple species might further impact the multiple hypothesis-testing problem? Could the authors propose some suggestions on how one might address this?

We agree that epistatic effects are entirely possible. Factors that control host interaction with the microbiome are just beginning to be understood, and it seems that a complex network of pathways and genes are involved. Moreover, these host genes and pathways might depend on the specific body site and host health status. Thus, interaction between genes is expected, as it is for many complex human traits. We agree that our method is not ideal to detect epistatic effects, given that the SNP is the response variable. The rationale behind using SNPs as response variable, as discussed in the paper (and see our response to point 1 above), is that it allows us to detect multiple taxa associated with each SNP. Nevertheless, epistatic effect could still potentially be identified by including the allele combinations of all interacting SNPs as the model response variable - this might be useful for detecting interactions between candidate SNPs, but unlikely to be sufficiently powered for a genome-wide test. We have added this point to the conclusions section of the paper, and included a short discussion of possible ways to address this, as suggested:

Results, page 9: Although HOMINID performs strongly on the data used in this paper, there are several potential limitations to our method. First, since it is especially designed to identify SNPs where a number of taxa are associated, it might not be optimal for cases where there is a dramatic shift in the microbiome that includes many dozens of taxa. Moreover, since the SNP is used as the response in the HOMINID model, it is difficult to identify epistatic effects, whereby genetic variation in two or more loci interact to affect microbiome composition. Although HOMINID could still be used to detect these interactions, by including all genotype combinations as response variables; however, multiple hypothesis testing could be an issue, especially for microbiome association studies, where samples sizes are currently small relative to GWAS of other complex traits. Nevertheless, HOMINID might be useful for detection of interaction of between candidate loci.

4. Synthetic dataset improvements:
* Data set 1: We recommend increasing the range of MAFs tested. It would be good to see a drop off in performance for Figures S4 and S5 (if there is a dropoff). We expect there to be lower performance for lower MAFs.

We agree with this point. To address this and relevant points from reviewer 1, we have enhanced simulation analysis, which now assesses a wide of variation in R2, as well as other factors, including variation in minor allele frequency (MAF) of the associated SNP. MAF values in the simulated data now range from 0.10 to 0.50. To assess the performance of our approach, we calculated and plotted the method's sensitivity, specificity, precision, negative predictive value (NPV), false positive rate (FPR), false negative rate (FNR), false discovery rate (FDR), and accuracy, as a function of the input R2. This analysis is described in the Supplementary Information (page 38) and the resulting plots for MAF are shown in Supplementary Figures S28 - S35. We also include a representative figure in the main text (Figure 2) to show these effects. Our analysis indicates that although several of the factors tested have an effect on HOMINID's performance, variation in MAF does not seem to have a strong effect. A summary of this analysis is provided in the text (page 8; copied below for convenience) and displayed in **Figure 2** (copied below as well):

Page 8, Results: We found that the strength of correlation (input R^2) between SNP genotype and the correlated taxa has little effect on HOMINID's ability to identify the SNP, unless the correlation is very low (**Figs. 2A** and **2B**, Supplementary Information, and Supplementary Figures S4 - S11). HOMINID achieved high sensitivity and specificity for R^2 values of above ~ 0.05. The False Discovery Rate (FDR) is below 0.1 by design, and variation in FDR is due to imprecision (finite number of significant digits) in calculation of $R^2_{L,}$ and therefore imprecision in calculation of q. (**Figs. 2C** and **2D**).

Similarly, variation in MAF does not affect HOMINID's sensitivity, as data sets with different MAF follow the same behavior (**Fig. 2B**).

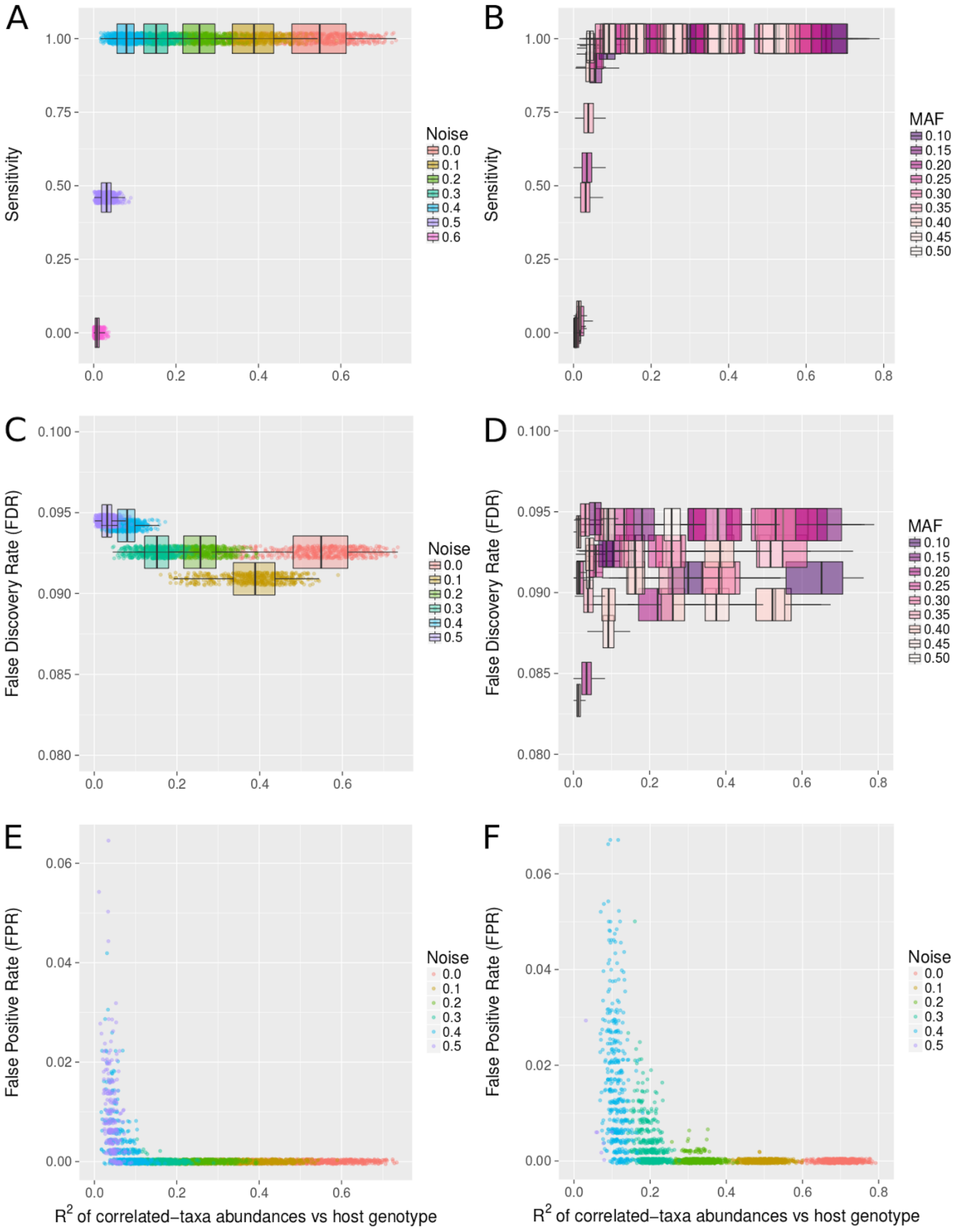

* What is the impact of relative abundances of associated species? Do very rare species have less predictive power? It would be helpful to have another synthetic data set to test this as it is very possible that the rare species in the microbiome have important impacts on human health.

Thank you for pointing out this important question. To find out if taxa relative abundances have an effect on prediction, we compared the relative abundances of false negative and true positive taxa. Our results indicate that relative abundance does not affect HOMINID's ability to predict the taxa that are correlated. We show this in Fig. S47, which we include below. The taxa that are predicted false negatives (correlated taxa that were incorrectly predicted to be uncorrelated, on the left) vs true positives (correlated taxa that were correctly predicted to be correlated, on the right) have the same abundance distributions, indicating that relative abundance does not affect whether a taxon is predicted to be correlated.

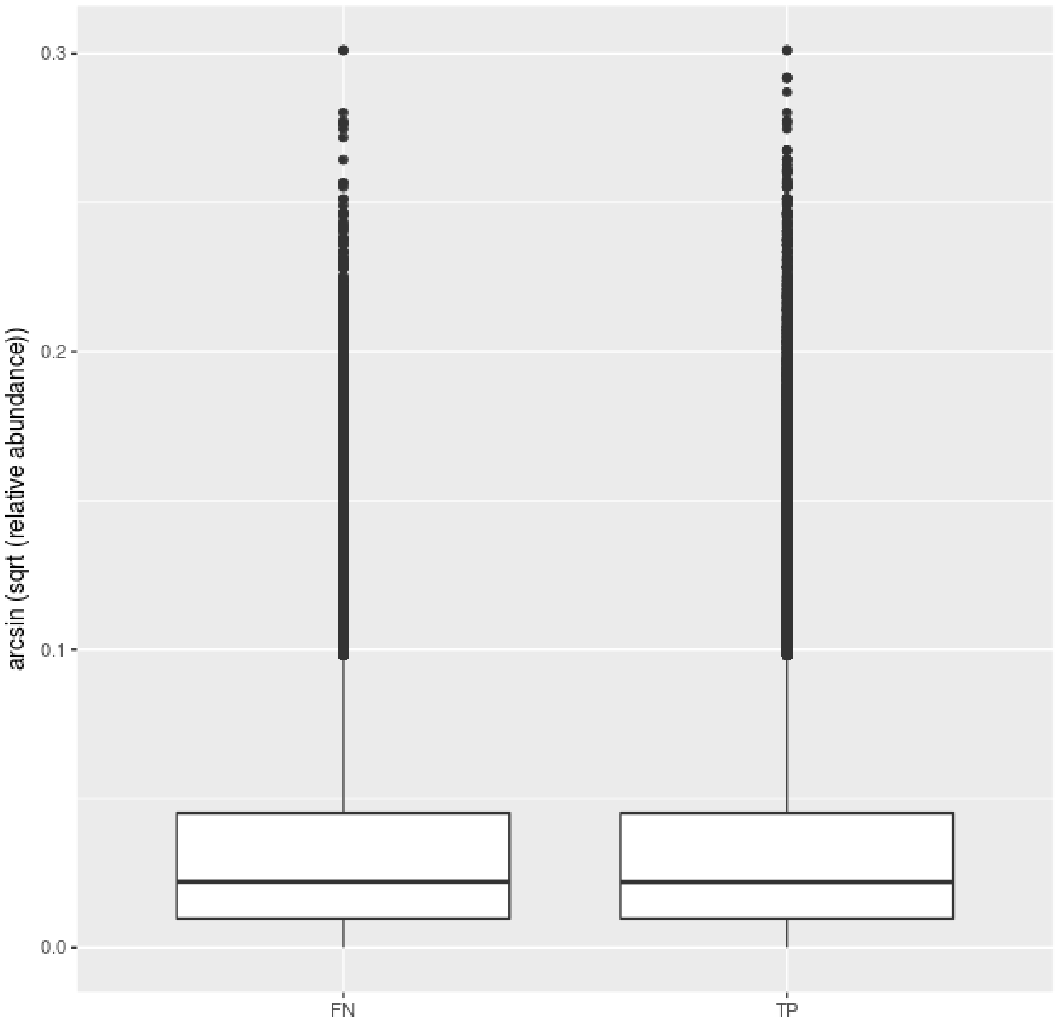

What affects whether a correlated taxon gets detected is the degree to which that taxon correlates with the host genotype, weakly correlated taxa being less likely to be detected. We show this in Fig. S48 (pasted below), which shows the taxon coefficient -- the degree of correlation -- for false negatives versus true positives. The absolute value of the taxon coefficient is smaller for false negatives (correlated taxa that were not detected by HOMINID) than true positives (correlated taxa that were detected).

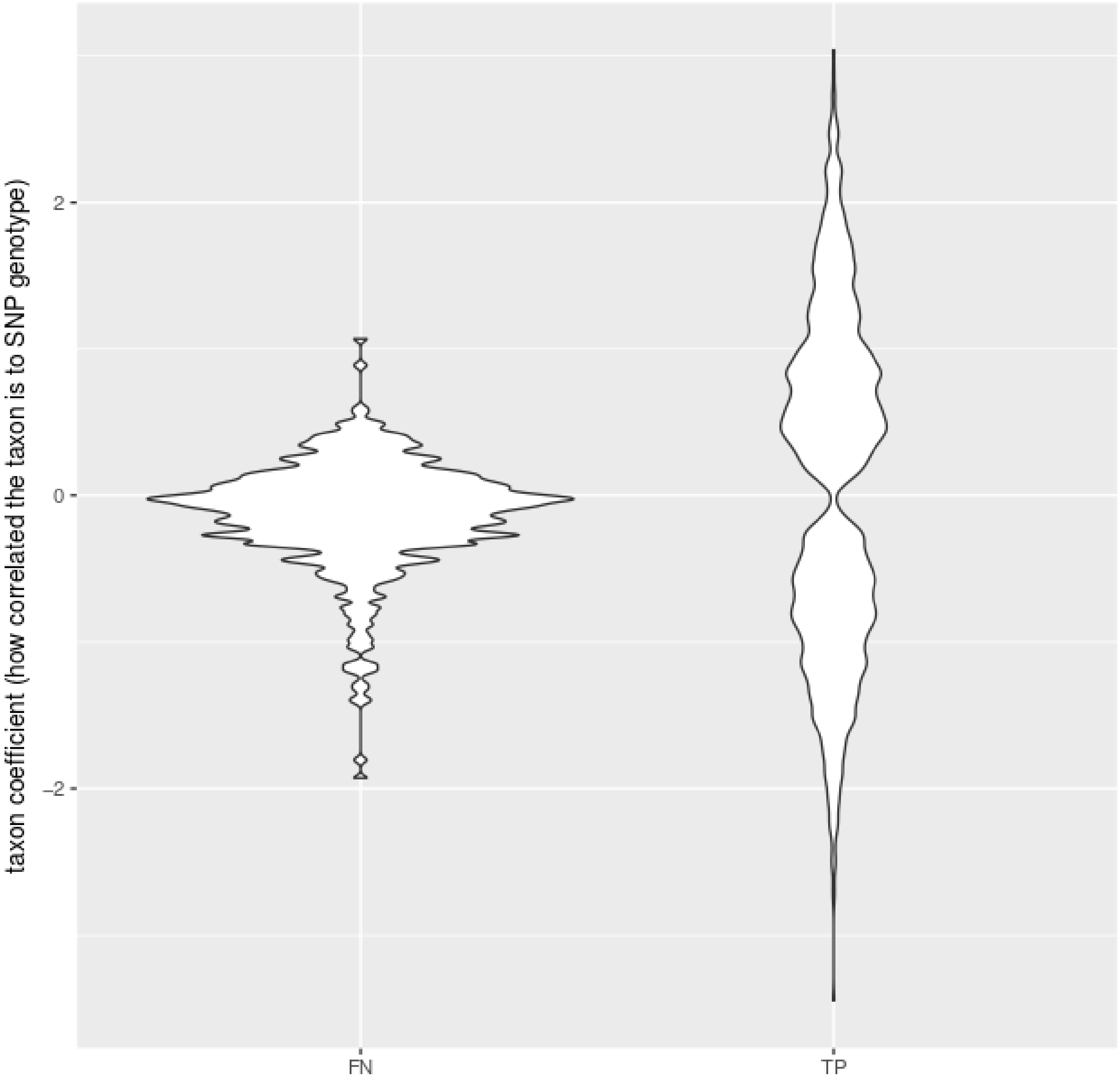

Minor Critiques:

1) Make it clearer that the predictor is the species rather than human SNP in the main text-usually it is the other way around

We agree that important details were missing about the specifics of the method in the previous version. The paper now includes a much enhanced Methods section, as well as a comprehensive Supplementary Information document. Specifically, using the taxa abundances rather than SNP as predictor is now described in the main text Methods section:

> **Page 4, Methods section:** The Lasso regression was implemented using the Python (version 2.7) machine-learning library scikit-learn (Pedregosa et al. 2011), with microbiome relative abundances as predictors and SNP genotype as response variable.

2) In the methods and the main text, could the authors make clearer that the predictors are species abundances rather than presence/absence (if true)?

Yes - please see the response to the previous point. We have also included a rationale for using this specific approach as described in our response to point 1.

3) In the main text, page 5: would be worth identifying which species are associated with the significant SNPs identified in ABCC8, the HLA genes, and ATL2.

As described above, we have updated the analysis to no longer use nominal p-values to determine which SNPs are considered significant, and instead use q-values that put an upper bound on the FDR. As we now report SNPs at an FDR of <= 0.1, only 13 SNPs are reported in the paper, and the SNPs listed above (in *ABCC8*, *HLA* genes, and *ATL2*) are no longer included in the results. For the associated SNPs that are reported in the revised version and visualized in **Figure 4**, we made sure to include the taxa that are associated with each in the figure. Also, in Supplementary Tables 1 and 2, which provide additional details on the associated SNPs, we list the taxa correlated with each SNPs.

4) Introduction, page 3, line 21: Goodrich et al. calculated heritability for Christensenellaceae to be <0.4, so, it is heritable but not highly heritable.

We have updated the text in this sentence, which now reads "identify bacterial taxa that are heritable, such as Christensenellaceae".

5) What was the rationale for testing for associations in different body sites separately? Couldn't the same host SNPs impact species composition in different body sites?

The rationale for testing each body site separately is that microbial communities could be very different across body sites (Consortium, Human Microbiome Project 2012). Thus, it is possible

that different mechanisms control host-microbiome interactions in different body sites, and thus many associated SNPs could be body-site specific. However, we agree that in some cases the same host pathway can control the microbiome across body sites - for example, immune-related pathways might have a universal effect through a common mechanism. Nevertheless, since microbiome composition across body sites is mostly different (depending on the specific body sites), the species will likely be different. Since HOMINID is designed to detect individual species correlated with host SNP genotypes, adding species abundances from multiple body sites might dilute the signal.

References

Anderson, Marti J. 2001. "A New Method for Non-Parametric Multivariate Analysis of Variance." *Austral Ecology* 26 (1). Wiley Online Library: 32–46.

Baldridge, Elita, David J. Harris, Xiao Xiao, and Ethan P. White. 2016. "An Extensive Comparison of Species-Abundance Distribution Models." *PeerJ* 4 (December): e2823.

Blekhman, Ran, Julia K. Goodrich, Katherine Huang, Qi Sun, Robert Bukowski, Jordana T. Bell, Timothy D. Spector, et al. 2015. "Host Genetic Variation Impacts Microbiome Composition across Human Body Sites." *Genome Biology* 16 (September): 191.

Consortium, Human Microbiome Project. 2012. "Structure, Function and Diversity of the Healthy Human Microbiome." *Nature* 486: 207–14.

McArdle, Brian H., and Marti J. Anderson. 2001. "Fitting Multivariate Models to Community Data: A Comment on Distance-Based Redundancy Analysis." *Ecology* 82 (1). Wiley Online Library: 290–97.

Pedregosa, Fabian, Gaël Varoquaux, Alexandre Gramfort, Vincent Michel, Bertrand Thirion, Olivier Grisel, Mathieu Blondel, et al. 2011. "Scikit-Learn: Machine Learning in Python." *Journal of Machine Learning Research: JMLR* 12 (Oct): 2825–30.

Price, Alkes L., Nick J. Patterson, Robert M. Plenge, Michael E. Weinblatt, Nancy A. Shadick, and David Reich. 2006. "Principal Components Analysis Corrects for Stratification in Genome-Wide Association Studies." *Nature Genetics* 38 (8): 904–9.

Pritchard, J. K., M. Stephens, N. A. Rosenberg, and P. Donnelly. 2000. "Association Mapping in Structured Populations." *American Journal of Human Genetics* 67 (1): 170–81.

Wang, Kai, Mingyao Li, and Hakon Hakonarson. 2010. "ANNOVAR: Functional Annotation of Genetic Variants from High-Throughput Sequencing Data." *Nucleic Acids Research* 38 (16): e164.

Zhao, Ni, Jun Chen, Ian M. Carroll, Tamar Ringel-Kulka, Michael P. Epstein, Hua Zhou, Jin J. Zhou, Yehuda Ringel, Hongzhe Li, and Michael C. Wu. 2015. "Testing in Microbiome-Profiling Studies with MiRKAT, the Microbiome Regression-Based Kernel Association Test." *American Journal of Human Genetics* 96 (5): 797–807.
