# Supplementary material for: HOMINID: a framework for identifying associations between host genetic variation and microbiome composition
Source: Gigascience. 2017 Nov 8;6(12):1–7. doi: 10.1093/gigascience/gix107 (PMC5740987; doi:10.1093/gigascience/gix107)
Supplement: GIGA-D-16-00138_Revision-2.pdf [file gix107_giga-d-16-00138_revision-2.pdf]

GigaScience

HOMINID: A framework for identifying associations between host genetic variation and microbiome composition

--Manuscript Draft--

**Manuscript Number:**	GIGA-D-16-00138R2
**Full Title:**	HOMINID: A framework for identifying associations between host genetic variation and microbiome composition
**Article Type:**	Research
**Funding Information:**	The Randy Shaver Cancer Research and Community Fund — Dr. Ran Blekhman<br>American Cancer Society (US) (124166-IRG-58-001-55-IRG53) — Dr. Ran Blekhman<br>Alfred P. Sloan Foundation — Dr. Ran Blekhman
**Abstract:**	Recent studies have uncovered a strong effect of host genetic variation on the composition of host-associated microbiota. Here, we present HOMINID, a computational approach based on Lasso linear regression, that given host genetic variation and microbiome taxonomic composition data, identifies host SNPs that are correlated with microbial taxa abundances. Using simulated data we show that HOMINID has accuracy in identifying associated SNPs, and performs better compared to existing methods. We also show that HOMINID can accurately identify the microbial taxa that are correlated with associated SNPs. Lastly, by using HOMINID on real data of human genetic variation and microbiome composition, we identified 13 human SNPs in which genetic variation is correlated with microbiome taxonomic composition across body sites. In conclusion, HOMINID is a powerful method to detect host genetic variants linked to microbiome composition, and can facilitate discovery of mechanisms controlling host-microbiome interactions.
**Corresponding Author:**	Ran Blekhman<br>University of Minnesota Twin Cities<br>UNITED STATES
**Corresponding Author Secondary Information:**	
**Corresponding Author's Institution:**	University of Minnesota Twin Cities
**Corresponding Author's Secondary Institution:**	
**First Author:**	Joshua Lynch
**First Author Secondary Information:**	
**Order of Authors:**	Joshua Lynch
	Karen Tang
	Sambhawa Priya
	Joanna Sands
	Margaret Sands
	Evan Tang
	Sayan Mukherjee
	Dan Knights
	Ran Blekhman
**Order of Authors Secondary Information:**	
**Response to Reviewers:**	The Response to Reviewers is attached to the end of the cover letter.

Powered by Editorial Manager® and ProduXion Manager® from Aries Systems Corporation

Additional Information:	
**Question**	**Response**
Are you submitting this manuscript to a special series or article collection?	No
**Experimental design and statistics**<br><br>Full details of the experimental design and statistical methods used should be given in the Methods section, as detailed in our Minimum Standards Reporting Checklist. Information essential to interpreting the data presented should be made available in the figure legends.<br><br>Have you included all the information requested in your manuscript?	Yes
**Resources**<br><br>A description of all resources used, including antibodies, cell lines, animals and software tools, with enough information to allow them to be uniquely identified, should be included in the Methods section. Authors are strongly encouraged to cite Research Resource Identifiers (RRIDs) for antibodies, model organisms and tools, where possible.<br><br>Have you included the information requested as detailed in our Minimum Standards Reporting Checklist?	Yes
**Availability of data and materials**<br><br>All datasets and code on which the conclusions of the paper rely must be either included in your submission or deposited in publicly available repositories (where available and ethically appropriate), referencing such data using a unique identifier in the references and in the “Availability of Data and Materials” section of your manuscript.<br><br>Have you have met the above requirement as detailed in our Minimum Standards Reporting Checklist?	Yes

Powered by Editorial Manager® and ProduXion Manager® from Aries Systems Corporation

Manuscript docx
Click here to download Manuscript Lynch_main_text.2nd_revision.docx

HOMINID: A framework for identifying associations between host genetic variation and microbiome composition

Joshua Lynch[1,2,#], Karen Tang[1,2], Sambhawa Priya[1,2], Joanna Sands[1,2], Margaret Sands[1,2], Evan Tang[1,2], Sayan Mukherjee[3], Dan Knights[4,5,*], Ran Blekhman[1,2,*]

[1] Department of Genetics, Cell Biology, and Development, University of Minnesota, Minneapolis, MN, USA

[2] Department of Ecology, Evolution, and Behavior, University of Minnesota, Minneapolis, MN, USA

[3] Departments of Statistical Science, Mathematics, and Computer Science, Duke University, Durham, NC, USA

[4] Department of Computer Science and Engineering, University of Minnesota, Minneapolis, MN, USA

[5] Biotechnology Institute, University of Minnesota, Minneapolis, MN, USA

*To whom correspondence should be addressed: blekhman@umn.edu (RB), dknights@umn.edu (DK)

[#]Current affiliation: Department of Agricultural and Biosystems Engineering, University of Arizona, Tucson, AZ, USA

Keywords: microbiome, host genetics, association, machine learning

Abstract

Recent studies have uncovered a strong effect of host genetic variation on the composition of host-associated microbiota. Here, we present HOMINID, a computational approach based on Lasso linear regression, that given host genetic variation and microbiome taxonomic composition data, identifies host SNPs that are correlated with microbial taxa abundances. Using simulated data we show that HOMINID has accuracy in identifying associated SNPs, and performs better compared to existing methods. We also show that HOMINID can accurately identify the microbial taxa that are correlated with associated SNPs. Lastly, by using HOMINID on real data of human genetic variation and microbiome composition, we identified 13 human SNPs in which genetic variation is correlated with microbiome taxonomic composition across body sites. In conclusion, HOMINID is a powerful method to detect host genetic variants linked to microbiome composition, and can facilitate discovery of mechanisms controlling host-microbiome interactions.

Availability and implementation

Software, code, tutorial, installation and setup details, and synthetic data are available in the project homepage: https://github.com/blekhmanlab/hominid.

Real dataset used here is from Blekhman et al. [1]; 16S rRNA gene sequence data and OTU tables are available on the HMP DACC website (www.hmpdacc.org), and host genetic data are deposited in dbGaP under project number phs000228.

Background

The microbial communities found in and on the human body are influenced by multiple factors [2]. In addition to the clear effect of environmental factors on the microbiome, there is growing support for an impact of host genetics [3,4]. Several candidate gene studies have found correlation between human genetic variation and the structure of the microbiome [5–7]. In addition, genome-wide approaches can also be useful to identify human genetic impact on the microbiome [1,8–10]. For example, Goodrich et al. used hundreds of twin pairs to calculate the heritability of the gut microbiome, and identify bacterial taxa that are heritable, such as Christensenellaceae [8]. Researchers have also utilized quantitative trait locus (QTL)-mapping approaches in the laboratory mouse and have identified multiple loci associated with the structure of gut microbial communities, some of which overlap genes involved in immune response [11,12]. Moreover, studies have used joint human genetic variation and microbiome data to find associations between loci in the human genome and microbial taxa [1,10,13,14]. In our recent study, in addition to showing that human genetic variation is associated with the structure of microbial communities across ten body sites, we have identified human single nucleotide polymorphisms (SNPs) associated with variation in the microbiome, and found that these loci are highly enriched in immunity genes and pathways [1]. This approach, which includes the joint analysis of host genetic variation (SNPs) and microbiome taxonomic composition data (usually an OTU table), has the important advantage of identifying specific host genes and pathways that may control the microbiome, thus shedding light on the biological mechanisms of host-microbiome interaction, and pinpointing potential disease-causing pathways. However, this analysis is complicated by the fact that the microbiome contains many taxa that can be used as potential molecular complex traits in the GWAS analysis. Testing many taxa

reduces the power and multiple hypothesis testing correction makes the identification of associations challenging.

Here, we propose a framework for identifying host SNPs associated with microbiome composition using Lasso regression, named **HOMINID** (**Ho**st-**M**icrobiome **In**teraction **Id**entification; see **Figure 1** and Supplementary Information). Our method has several advantages: (1) it takes as input host genetic variation data (in a modified VCF format) and microbiome taxonomic composition data (relative abundance data as an OTU table), to facilitate a simple analysis pipeline with no need to make new data formats; (2) HOMINID uses Lasso regression, which is specifically designed for cases where a relatively small number of taxa are correlated with host SNP genotype, as opposed to existing methods that use all taxa abundances; and (3) HOMINID uses stability selection with randomized Lasso to identify the specific microbial taxa that are correlated with each associated SNP.

Materials and Methods

HOMINID implementation. We implemented Lasso regression with the taxon relative abundances (arcsin sqrt transformed) as predictors and genetic variation at each SNP as response, for the purpose of identifying an additive effect between host genotype and microbiome features (see Supplementary Information and Figures S1-S3). In most situations, we expect at most a few taxa's abundances to correlate with a SNP, therefore ordinary least-squares (OLS) regression, which includes all taxa abundances as predictor variables, might not be an appropriate model. Instead, we need a regression algorithm that selects only the few predictors (taxa) that correlate to host genetics and discards the rest. The Lasso linear regression model used for HOMINID is

similar to OLS regression, except that it includes an additional penalty term that shrinks most regression coefficients to zero, resulting in a sparse solution; thus it predicts only a few taxa to correlate with the host genetics. The Lasso regression was implemented using the Python (version 2.7/3.5+) machine-learning library scikit-learn [15], with microbiome relative abundances as predictors and SNP genotype as response variable. The penalty term was tuned via a five-fold cross-validation. How well the host genetics correlates with the microbiome is measured with the coefficient of determination, $\square^2_\square$, calculated via a nested cross-validation procedure; $\square^2_\square$ is the median $\square^2$ from five-fold cross-validation, with 100-times resampling. Also outputted are 95th percentile bootstrap confidence intervals from 10,000 bootstrap samples. Detailed description of the implementation of Lasso regression is available in the Supplementary Information.

Identifying correlated SNPs and taxa. To identify SNPs that are predicted correlated to the microbiome (prediction positive) from the uncorrelated (prediction negative) HOMINID uses a q-value cutoff, which puts an upper bound on the False Discovery Rate (FDR). A cutoff value, $\square^2_\square$, of $\square^2_\square$ is chosen such that the q-value, $\square(\square^2_\square)$, is equal to 0.1. A given SNP is predicted positive (predicted correlated to the microbiome) if $\square^2_\square \geq \square^2_\square$. $\square(\square^2_\square)$ is determined by a permutation test, whereby for each SNP the sample labels are shuffled and Lasso regression is rerun ten times. $\square(\square^2_\square)$ is defined as the fraction of permuted SNPs predicted positive divided by the fraction of unpermuted SNPs predicted positive [16]. $\square^2_\square$ is chosen such that $\square(\square^2_\square) = 0.1$. The taxa that are most strongly associated with a SNP are identified using Stability Selection with randomized Lasso [17]. Briefly, stability selection perturbs the regression coefficients and the penalty term in the Lasso regression, and then reruns the regression thousands of times. If the same predictors (taxa) are repeatedly selected, even when the odds are against them, then they

are robust predictors. Full details on this procedure are available in the Supplementary Information.

Controlling for other (non-taxon) covariates. HOMINID allows for controlling for any additional covariates (other than the microbiome) by including the covariates in the microbiome taxonomic table. This enables controlling for potentially confounding factors, such as individual age and sex. It also enables controlling for ancestry (or population substructure) by including the principal components (PCs) of the genetic variation data [18,19] in the analysis. We performed two analyses using HMP data, one including host genetic PCs as covariates (results in Supplementary Table S1), and one without these covariates (Supplementary Table S2), both including sex as covariate.

Synthetic datasets. To test the performance of HOMINID we generated several synthetic datasets. "Taxon" absolute abundances ("counts") were drawn from a log-series distribution. The log-series distribution is frequently used to represent species abundances (see, e.g., [20]), and it allows a range of abundances that spans several orders of magnitude, mimicking both rare and abundant taxa. Often in real abundance tables a large fraction of taxa have an abundance of zero (taxon either not present or not detected). The log-series abundance tables also had this quality; in our synthetic data, 21% of abundances are count zero. Synthetic data were generated such that, for each SNP independently, $\square_{\square\square\square}$ ("ctc" stands for correlated-taxon count) random taxa's abundances correlate with that SNP's genotype. Uncorrelated SNPs were created by permuting the sample IDs, preserving the minor allele frequency. Once the SNP and taxon-abundance data were generated, a measure of the effect size was calculated: the coefficient of determination, $\square^{2}_{\square\square\square}$, for an ordinary least square (OLS) multiple regression between the correlated taxa's abundances and the SNP genotype. Since $\square^{2}_{\square\square\square}$ is a characteristic of the input data before

analysis by HOMINID, we call it the "input $\square^{2}$" to distinguish it from the $\square^{2}$output by the HOMINID Lasso regression (aka the "output $\square^{2}$" or $\square^{2}_{\square}$). To examine data sets with smaller effect sizes, "noise" was added to the SNP data by swapping the genotypes of pairs of samples, reducing the correlation between the $\square_{\square\square\square}$correlated taxa and the host SNP genotype. In datasets with noise level *P*, the probability that a random sample's genotypes are *not* correlated with the correlated-taxa's abundances is *P*. Several data sets were created with progressively more "noise", until $\square^{2}_{\square\square\square} \rightarrow 0$. We created three sets of synthetic data to examine the performance of HOMINID on different qualities of the input data: Data set MAF varies the minor allele frequency, with MAF ranging from 0.10 to 0.50; data set CTC varies the number of correlated taxa from five to twenty; and data set TC varies the total number of taxa in the taxon table from 100 to 500. All data sets contain 500 SNPs each. Data in sets MAF and CTC comprise 1000 individuals; data sets in set TC contain 100 individuals. Data sets MAF and TC all have three correlated taxa per SNP. The MAF for data sets CTC and TC is 0.30.

Human Microbiome Project data. In addition to the synthetic datasets described above, we also tested our method on a real dataset that includes both human genetic and microbiome data [1]. This dataset includes 93 individuals for whom microbiome was profiled as part of the Human Microbiome Project, and for which host genetic variation information was extracted from shotgun metagenomics sequence data as described previously [1]. We annotated the previously described set of 4.2 million high-quality single nucleotide polymorphisms (SNPs) using ANNOVAR [21] and focused the analysis on a set of 32,696 protein-coding SNPs. We further filtered this set to include only SNPs with minor allele frequency of at least 20% and SNPs for which we had data for at least 50 individuals. The number of SNPs actually tested varies across body sites, ranging from 12,400 to 14,651 SNPs, with a mean of 14,023. For the

Stool microbiome data, which included 107 total taxa, running HOMINID on 14,469 SNPs using 12-core Intel Xeon E5-2680 2.50 GHz processors took 16 cpu hours.

Comparison to other methods. The PERMANOVA [22,23] analysis was done in R with the adonis function in the vegan [24] package. The model formula has the SNP genotype as numeric (not factor) predictor variables and the arcsin-sqrt transformed taxon relative abundance table as response variable. The method used to calculate pairwise “distances” was the default Bray-Curtis. The MiRKAT [25] analysis was performed using the MiRKAT package in R. The Bray-Curtis dissimilarity matrix was computed on the arcsin-sqrt transformed taxon table. The matrix was then converted to a kernel matrix, and MiRKAT invoked for each SNP. Since both PERMANOVA and MiRKAT output p-values as measures of how well the taxon abundances correlate with each SNP’s genotype (whereas HOMINID outputs $\square^{2}_{\square}$ values) we chose a cutoff value of p-value such that $\square(\square_{\square})$= 0.1 to separate the prediction positives (correlated) from the prediction negatives (uncorrelated), much in the same way we chose the cutoff $\square^{2}_{\square}$ to separate prediction positive/negative such that $\square(\square^{2}_{\square})$= 0.1 for the Lasso regression.

Results

Analysis using synthetic data. To assess HOMINID’s performance, we first used the pipeline on a comprehensive set of synthetic datasets (described above and in the Supplementary Information). These datasets were designed to simulate variation in several important factors, such as variation of the strength of correlation (the input $\square^{2}$) of the associated SNP with microbiome composition, variation in minor allele frequency (MAF) of the associated SNP,

noise level in microbiome data, and the number of taxa associated with the SNP. After analyzing each of the datasets we calculated and plotted the method's sensitivity, specificity, precision, negative predictive value (NPV), false positive rate (FPR), false negative rate (FNR), false discovery rate (FDR), and accuracy, as a function of the input $\square^2$, highlighting the effects of the variable factors above (see **Figures 2A-D**, Supplementary Information and Supplementary Figures S5 - S44).

We found that the strength of correlation (input $\square^2$) between SNP genotype and the correlated taxa has little effect on HOMINID's ability to identify the SNP, unless the correlation is very low (**Figures 2A** and **2B**, Supplementary Information, and Supplementary Figures S5 - S12). HOMINID achieved high sensitivity and specificity for $\square^2$values of above ~ 0.05. The False Discovery Rate (FDR) is below 0.1 by design, and variation in FDR is due to imprecision (finite number of significant digits) in calculation of $\square^2_\square$, and therefore imprecision in calculation of q. (**Figures 2C** and **2D)**. Similarly, variation in MAF does not affect HOMINID's sensitivity, as data sets with different MAF follow the same behavior (**Figure 2B**).

One of HOMINID's unique features is the ability to identify the taxa that are correlated with an associated SNP. We found that this prediction performs well, with accuracy approaching 1 and a false positive rate (FPR) of 0 for input $\square^2$values larger than about 0.1, but drops off at lower $\square^2$values (**Figures 2E** and **2F**, Supplementary Figures S27 and S28). The number of correlated taxa had a noticeable effect, whereby SNPs that correlated with more taxa had higher FPR (compare **Figure 2E** with **Figure 2F**), although in all test datasets' FPR remained < 0.07.

Comparison to other methods. In order to assess HOMINID's performance, we compared it to PERMANOVA [22,23] and MiRKAT [25], two platforms that can be used to identify host

SNPs associated with microbiome composition. We note that HOMINID has a unique feature allowing it to identify the specific microbial taxa associated with each SNP. Since other approaches lack this option, the comparison centered around the ability to detect SNPs that are correlated with the microbiome, and not on the detection of correlated taxa. Our analysis included input datasets with various input $\square^{2}$values and noise levels (various effect sizes), and compared the sensitivity of each method to detect the associated SNPs. We found that for median input $\square^{2}$values (correlation between associated SNP and microbiome composition) of about 0.15 or above the three methods are all highly sensitive (**Figure 3**). However, for lower input $\square^{2}$values, HOMINID is more sensitive. Specifically, for the data set with median input $\square^{2}$= 0.08 HOMINID's sensitivity is 1, while the sensitivity of MiRKAT and PERMANOVA is 0.19 and 0.29, respectively (**Figure 3**). Similarly, for median input $\square^{2}$= 0.03 HOMINID's sensitivity is 0.46, while the other methods' sensitivities are 0.

Analysis of Human Microbiome Project data. We ran the HOMINID pipeline on a previously published data of microbiome and host genetic variation from the Human Microbiome Project cohort [1]. We focused our analysis on coding SNPs with minor allele frequency ≥ 0.2, and identified SNPs for which permutation-based q-value ≤ 0.1 and the 95th percentile confidence interval for $\square^{2}$does not include zero. To account for population substructure, we ran a second analysis including the genetic principal components (PCs) as additional covariates [18,19]. This resulted in the identification of 11 (regression with genetic PCs as covariates) and 6 (regression without genetic PCs) for a total of 13 unique associations between host SNP and microbiome composition across 15 body sites (see Supplementary Tables S1 and S2, respectively). As can be seen in Figure 4, HOMINID is able to detect SNPs with the expected pattern of association between host genetic variation and the

microbiome. For example, for SNP rs2297345 in the gene *PAK7* we detected a correlation between genotype and a single microbial taxon, Propionibacteriaceae (**Figure 4A**). HOMINID can also detect SNPs where multiple taxa are correlated with the same SNP (e.g., SNP rs6032 in **Figure 4B**), as well as more complex patterns of association; for example, for SNP rs230898 in the gene *TEKT3* (**Figure 4C**) genetic variation is positively correlated with one taxon (Clostridia) and negatively with others (Rhodocyclales and Aerococcaceae).

Although HOMINID performs strongly on the data used in this paper, there are several potential limitations to our method. First, since it is especially designed to identify SNPs where a number of taxa are associated, it might not be optimal for cases where there is a dramatic shift in the microbiome that includes many dozens of taxa. Moreover, since the SNP is used as the response in the HOMINID model, it is difficult to identify epistatic effects, whereby genetic variation in two or more loci interact to affect microbiome composition. Although HOMINID could still be used to detect these interactions, by including all genotype combinations as response variables; however, multiple hypothesis testing could be an issue, especially for microbiome association studies, where samples sizes are currently small relative to GWAS of other complex traits. Nevertheless, HOMINID might be useful for detection of interaction of between candidate loci.

Lastly, we developed a web-based tool for the visualization of host-microbiome interaction network identified in HOMINID, available at http://z.umn.edu/genemicrobe. The website, designed using D3.js with a dedicated MySQL database serving as the back-end, displays a dynamic visualization of host gene-microbiome taxa interaction networks, and allows the user to add and remove nodes (host gene and microbial taxa), adjust the display size and node locations, filter by body sites, and generate figures. Currently, the website includes toy data

representing all SNP-microbe associations with a nominal p-value ≤ 0.1 in the Human Microbiome Project data described above. We believe that as studies using larger sample sizes materialize (for example, a recent study included 1,514 subjects [13]), we expect this tool to be useful for visualization of much larger number of associations.

Conclusions

We present HOMINID, a framework designed for identifying associations between host genetic variation and microbiome composition. We analyze synthetic data to show HOMINID's overall strong performance, identify specific factors that may affect it, highlight HOMINID's unique features, and show HOMINID's utility with a real dataset. We expect that HOMINID would be useful for studies attempting to characterize the genetic basis of host-microbiome interactions.

Funding

This work is supported in part by funds from the University of Minnesota College of Biological Sciences, The Randy Shaver Cancer Research and Community Fund, Institutional Research Grant #124166-IRG-58-001-55-IRG53 from the American Cancer Society, and a Research Fellowship from The Alfred P. Sloan Foundation. This work was facilitated in part by computational resources provided by the Minnesota Supercomputing Institute.

Figure Legends

Figure 1. Illustration of the HOMINID pipeline

Figure 2. Assessment of HOMINID's performance using synthetic data. Panels **A-D** assess how well HOMINID predicts the SNPs whose genotypes correlate with microbiome abundances, and panels **E** and **F** assess how well HOMINID predicts the specific taxa correlated with an associated SNP. **(A)** Sensitivity as a function of effect size (input $\square^{2}$) for the data sets with MAF=0.30. Different colored points and boxplots represent data sets with different noise levels and therefore different effect sizes. (**B**) Same as A with variation in input data MAF values represented by different colored points at each dataset's median input $\square^{2}$. See Supplemental Figure S30 for a visualization of the same data with boxplots instead of medians. (**C)** FDR as a function of effect size (input $\square^{2}$) for data sets with just MAF=0.30. (**D**) Same as C with variation in input MAF values represented by different colored points at each dataset's median input $\square^{2}$. See Supplemental Figure S35 for a visualization of the same data with boxplots instead of medians. (**E)** FPR for the stability selection step (identifying the taxa that associate with a SNP's genotype), as a function of effect size (input $\square^{2}$) for data sets with three correlated taxa. (**F**) Same as E but with twenty correlated taxa.

Figure 3. Comparison of the performance of HOMINID versus MiRKAT and PERMANOVA. Sensitivity is plotted as a function of effect size (input $\square^{2}$) for HOMINID (red), MirKAT (green), and PERMANOVA (blue). At high input $\square^{2}$all three methods perform well, finding all SNPs that correlate with the microbiome. However, at smaller effect sizes (lower input $\square^{2}$), HOMINID is more sensitive.

Figure 4. Examples of SNPs where correlations were found between host genetic variation and the microbiome. Three SNPs are shown: **(A)** rs2297345 (correlated with abundance of

microbial taxa in the right antecubital fossa), **(B)** rs6032 correlated with abundance of microbial taxa in the throat), and **(C)** rs230898 (correlated with abundance of microbial taxa in the supragingival plaque). The x-axis shows the host SNP genotypes, and the y-axis shows the arcsin sqrt transformed taxon abundances. The different correlated taxa for each SNP are shown in different colors. See Figure S59 for a visualization of the results in panel 4B, but omitting the highest abundance taxon, Bacteroidetes (dark green), to better display the trends for the three lower abundance taxa.

References

1. Blekhman R, Goodrich JK, Huang K, Sun Q, Bukowski R, Bell JT, et al. Host genetic variation impacts microbiome composition across human body sites. Genome Biol. 2015;16:191.

2. Consortium, Human Microbiome Project. Structure, function and diversity of the healthy human microbiome. Nature. 2012;486:207–14.

3. Goodrich JK, Davenport ER, Waters JL, Clark AG, Ley RE. Cross-species comparisons of host genetic associations with the microbiome. Science. 2016;352:532–5.

4. Morton ER, Lynch J, Froment A, Lafosse S, Heyer E, Przeworski M, et al. Variation in Rural African Gut Microbiota Is Strongly Correlated with Colonization by Entamoeba and Subsistence. PLoS Genet. 2015;11:e1005658.

5. Tong M, McHardy I, Ruegger P, Goudarzi M, Kashyap PC, Haritunians T, et al. Reprograming of gut microbiome energy metabolism by the FUT2 Crohn's disease risk polymorphism. ISME J. 2014;8:2193–206.

6. Khachatryan ZA, Ktsoyan ZA, Manukyan GP, Kelly D, Ghazaryan KA, Aminov RI. Predominant role of host genetics in controlling the composition of gut microbiota. PLoS One. 2008;3:e3064.

7. Knights D, Silverberg MS, Weersma RK, Gevers D, Dijkstra G, Huang H, et al. Complex host genetics influence the microbiome in inflammatory bowel disease. Genome Med. 2014;6:107.

8. Goodrich JK, Waters JL, Poole AC, Sutter JL, Koren O, Blekhman R, et al. Human genetics shape the gut microbiome. Cell. 2014;159:789–99.

9. Goodrich JK, Davenport ER, Beaumont M, Jackson MA, Knight R, Ober C, et al. Genetic Determinants of the Gut Microbiome in UK Twins. Cell Host Microbe. 2016;19:731–43.

10. Davenport ER, Cusanovich DA, Michelini K, Barreiro LB, Ober C, Gilad Y. Genome-Wide Association Studies of the Human Gut Microbiota. PLoS One. dx.plos.org; 2015;10:e0140301.

11. Benson AK, Kelly SA, Legge R, Ma F, Low SJ, Kim J, et al. Individuality in gut microbiota composition is a complex polygenic trait shaped by multiple environmental and host genetic factors. Proc. Natl. Acad. Sci. U. S. A. 2010;107:18933–8.

12. Leamy LJ, Kelly SA, Nietfeldt J, Legge RM, Ma F, Hua K, et al. Host genetics and diet, but not immunoglobulin A expression, converge to shape compositional features of the gut microbiome in an advanced intercross population of mice. Genome Biol. 2014;15:552.

13. Bonder MJ, Kurilshikov A, Tigchelaar EF, Mujagic Z, Imhann F, Vila AV, et al. The effect of host genetics on the gut microbiome. Nat. Genet. [Internet]. Nature Research; 2016 [cited 2016 Oct 5]; Available from: http://dx.doi.org/10.1038/ng.3663

14. Turpin W, Espin-Garcia O, Xu W, Silverberg MS, Kevans D, Smith MI, et al. Association of host genome with intestinal microbial composition in a large healthy cohort. Nat. Genet. 2016;48:1413–7.

15. Pedregosa F, Varoquaux G, Gramfort A, Michel V, Thirion B, Grisel O, et al. Scikit-learn: Machine Learning in Python. J. Mach. Learn. Res. 2011;12:2825–30.

16. Subramanian A, Tamayo P, Mootha VK, Mukherjee S, Ebert BL, Gillette MA, et al. Gene set enrichment analysis: a knowledge-based approach for interpreting genome-wide expression profiles. Proc. Natl. Acad. Sci. U. S. A. 2005;102:15545–50.

17. Meinshausen N, Bühlmann P. Stability selection. J. R. Stat. Soc. Series B Stat. Methodol. Blackwell Publishing Ltd; 2010;72:417–73.

18. Price AL, Patterson NJ, Plenge RM, Weinblatt ME, Shadick NA, Reich D. Principal components analysis corrects for stratification in genome-wide association studies. Nat. Genet. 2006;38:904–9.

19. Pritchard JK, Stephens M, Rosenberg NA, Donnelly P. Association mapping in structured populations. Am. J. Hum. Genet. 2000;67:170–81.

20. Baldridge E, Harris DJ, Xiao X, White EP. An extensive comparison of species-abundance distribution models. PeerJ. 2016;4:e2823.

21. Wang K, Li M, Hakonarson H. ANNOVAR: functional annotation of genetic variants from high-throughput sequencing data. Nucleic Acids Res. 2010;38:e164.

22. Anderson MJ. A new method for non-parametric multivariate analysis of variance. Austral Ecol. Wiley Online Library; 2001;26:32–46.

23. McArdle BH, Anderson MJ. Fitting multivariate models to community data: a comment on distance-based redundancy analysis. Ecology. Wiley Online Library; 2001;82:290–7.

24. Oksanen J, Kindt R, Legendre P, O'Hara B, Stevens MHH, Oksanen MJ, et al. The vegan package. Community ecology package. 2007;10:631–7.

25. Zhao N, Chen J, Carroll IM, Ringel-Kulka T, Epstein MP, Zhou H, et al. Testing in Microbiome-Profiling Studies with MiRKAT, the Microbiome Regression-Based Kernel Association Test. Am. J. Hum. Genet. 2015;96:797–807.

Manuscript pdf, with correct equations & math symbols
Click here to download Manuscript
Lynch_main_text.2nd_revision.pdf

HOMINID: A framework for identifying associations between host genetic variation and microbiome composition

Joshua Lynch[1,2,#], Karen Tang[1,2], Sambhawa Priya[1,2], Joanna Sands[1,2], Margaret Sands[1,2], Evan Tang[1,2], Sayan Mukherjee[3], Dan Knights[4,5,*], Ran Blekhman[1,2,*]

[1] Department of Genetics, Cell Biology, and Development, University of Minnesota, Minneapolis, MN, USA

[2] Department of Ecology, Evolution, and Behavior, University of Minnesota, Minneapolis, MN, USA

[3] Departments of Statistical Science, Mathematics, and Computer Science, Duke University, Durham, NC, USA

[4] Department of Computer Science and Engineering, University of Minnesota, Minneapolis, MN, USA

[5] Biotechnology Institute, University of Minnesota, Minneapolis, MN, USA

*To whom correspondence should be addressed: blekhman@umn.edu (RB), dknights@umn.edu (DK)

#Current affiliation: Department of Agricultural and Biosystems Engineering, University of Arizona, Tucson, AZ, USA

Keywords: microbiome, host genetics, association, machine learning

Abstract

Recent studies have uncovered a strong effect of host genetic variation on the composition of host-associated microbiota. Here, we present HOMINID, a computational approach based on Lasso linear regression, that given host genetic variation and microbiome taxonomic composition data, identifies host SNPs that are correlated with microbial taxa abundances. Using simulated data we show that HOMINID has accuracy in identifying associated SNPs, and performs better compared to existing methods. We also show that HOMINID can accurately identify the microbial taxa that are correlated with associated SNPs. Lastly, by using HOMINID on real data of human genetic variation and microbiome composition, we identified 13 human SNPs in which genetic variation is correlated with microbiome taxonomic composition across body sites. In conclusion, HOMINID is a powerful method to detect host genetic variants linked to microbiome

composition, and can facilitate discovery of mechanisms controlling host-microbiome interactions.

Availability and implementation

Software, code, tutorial, installation and setup details, and synthetic data are available in the project homepage: https://github.com/blekhmanlab/hominid.

Real dataset used here is from Blekhman et al. [1]; 16S rRNA gene sequence data and OTU tables are available on the HMP DACC website (www.hmpdacc.org), and host genetic data are deposited in dbGaP under project number phs000228.

Background

The microbial communities found in and on the human body are influenced by multiple factors [2]. In addition to the clear effect of environmental factors on the microbiome, there is growing support for an impact of host genetics [3,4]. Several candidate gene studies have found correlation between human genetic variation and the structure of the microbiome [5–7]. In addition, genome-wide approaches can also be useful to identify human genetic impact on the microbiome [1,8–10]. For example, Goodrich et al. used hundreds of twin pairs to calculate the heritability of the gut microbiome, and identify bacterial taxa that are heritable, such as Christensenellaceae [8]. Researchers have also utilized quantitative trait locus (QTL)-mapping approaches in the laboratory mouse and have identified multiple loci associated with the structure of gut microbial communities, some of which overlap genes involved in immune response [11,12]. Moreover, studies have used joint human genetic variation and microbiome data to find associations between loci in the human genome and microbial taxa [1,10,13,14]. In our recent study, in addition to showing that human genetic variation is associated with the structure of microbial communities across ten body sites, we have identified human single nucleotide polymorphisms (SNPs) associated with variation in the microbiome, and found that these loci are highly enriched in immunity genes and pathways [1]. This approach, which includes the joint analysis of host genetic variation (SNPs) and microbiome taxonomic composition data (usually an OTU table), has the important advantage of identifying specific host genes and pathways that may control the microbiome, thus shedding light on the biological mechanisms of host-microbiome interaction, and pinpointing potential disease-causing pathways. However, this analysis is complicated by the fact that the microbiome contains many taxa that

can be used as potential molecular complex traits in the GWAS analysis. Testing many taxa reduces the power and multiple hypothesis testing correction makes the identification of associations challenging.

Here, we propose a framework for identifying host SNPs associated with microbiome composition using Lasso regression, named **HOMINID** (**Ho**st-**M**icrobiome **In**teraction **Id**entification; see **Figure 1** and Supplementary Information). Our method has several advantages: (1) it takes as input host genetic variation data (in a modified VCF format) and microbiome taxonomic composition data (relative abundance data as an OTU table), to facilitate a simple analysis pipeline with no need to make new data formats; (2) HOMINID uses Lasso regression, which is specifically designed for cases where a relatively small number of taxa are correlated with host SNP genotype, as opposed to existing methods that use all taxa abundances; and (3) HOMINID uses stability selection with randomized Lasso to identify the specific microbial taxa that are correlated with each associated SNP.

Materials and Methods

HOMINID implementation. We implemented Lasso regression with the taxon relative abundances (arcsin sqrt transformed) as predictors and genetic variation at each SNP as response, for the purpose of identifying an additive effect between host genotype and microbiome features (see Supplementary Information and Figures S1-S3). In most situations, we expect at most a few taxa's abundances to correlate with a SNP, therefore ordinary least-squares (OLS) regression, which includes all taxa abundances as predictor variables, might not be an appropriate model.

Instead, we need a regression algorithm that selects only the few predictors (taxa) that correlate to host genetics and discards the rest. The Lasso linear regression model used for HOMINID is similar to OLS regression, except that it includes an additional penalty term that shrinks most regression coefficients to zero, resulting in a sparse solution; thus it predicts only a few taxa to correlate with the host genetics. The Lasso regression was implemented using the Python (version 2.7/3.5+) machine-learning library scikit-learn [15], with microbiome relative abundances as predictors and SNP genotype as response variable. The penalty term was tuned via a five-fold cross-validation. How well the host genetics correlates with the microbiome is measured with the coefficient of determination, R_L^2, calculated via a nested cross-validation procedure; R_L^2 is the median R^2 from five-fold cross-validation, with 100-times resampling. Also outputted are 95th percentile bootstrap confidence intervals from 10,000 bootstrap samples. Detailed description of the implementation of Lasso regression is available in the Supplementary Information.

Identifying correlated SNPs and taxa. To identify SNPs that are predicted correlated to the microbiome (prediction positive) from the uncorrelated (prediction negative) HOMINID uses a q-value cutoff, which puts an upper bound on the False Discovery Rate (FDR). A cutoff value, R_C^2, of R_L^2 is chosen such that the q-value, $q(R_C^2)$, is equal to 0.1. A given SNP is predicted positive (predicted correlated to the microbiome) if $R_L^2 \geq R_C^2$. $q(R_C^2)$ is determined by a permutation test, whereby for each SNP the sample labels are shuffled and Lasso regression is rerun ten times. $q(R_C^2)$ is defined as the fraction of permuted SNPs predicted positive divided by the fraction of unpermuted SNPs predicted positive [16]. R_C^2 is chosen such that $q(R_C^2) = 0.1$. The taxa that are most strongly associated with a SNP are identified using Stability Selection

with randomized Lasso [17]. Briefly, stability selection perturbs the regression coefficients and the penalty term in the Lasso regression, and then reruns the regression thousands of times. If the same predictors (taxa) are repeatedly selected, even when the odds are against them, then they are robust predictors. Full details on this procedure are available in the Supplementary Information.

Controlling for other (non-taxon) covariates. HOMINID allows for controlling for any additional covariates (other than the microbiome) by including the covariates in the microbiome taxonomic table. This enables controlling for potentially confounding factors, such as individual age and sex. It also enables controlling for ancestry (or population substructure) by including the principal components (PCs) of the genetic variation data [18,19] in the analysis. We performed two analyses using HMP data, one including host genetic PCs as covariates (results in Supplementary Table S1), and one without these covariates (Supplementary Table S2), both including sex as covariate.

Synthetic datasets. To test the performance of HOMINID we generated several synthetic datasets. "Taxon" absolute abundances ("counts") were drawn from a log-series distribution. The log-series distribution is frequently used to represent species abundances (see, e.g., [20]), and it allows a range of abundances that spans several orders of magnitude, mimicking both rare and abundant taxa. Often in real abundance tables a large fraction of taxa have an abundance of zero (taxon either not present or not detected). The log-series abundance tables also had this quality; in our synthetic data, 21% of abundances are count zero. Synthetic data were generated such that, for each SNP independently, N_{ctc} ("ctc" stands for correlated-taxon count) random taxa's

abundances correlate with that SNP's genotype. Uncorrelated SNPs were created by permuting the sample IDs, preserving the minor allele frequency. Once the SNP and taxon-abundance data were generated, a measure of the effect size was calculated: the coefficient of determination, R^2_{OLS}, for an ordinary least square (OLS) multiple regression between the correlated taxa's abundances and the SNP genotype. Since R^2_{OLS} is a characteristic of the input data before analysis by HOMINID, we call it the "input R^2" to distinguish it from the R^2 output by the HOMINID Lasso regression (aka the "output R^2" or R^2_L). To examine data sets with smaller effect sizes, "noise" was added to the SNP data by swapping the genotypes of pairs of samples, reducing the correlation between the N_{ctc} correlated taxa and the host SNP genotype. In datasets with noise level *P*, the probability that a random sample's genotypes are *not* correlated with the correlated-taxa's abundances is *P.* Several data sets were created with progressively more "noise", until $R^2_{OLS} \rightarrow 0$. We created three sets of synthetic data to examine the performance of HOMINID on different qualities of the input data: Data set MAF varies the minor allele frequency, with MAF ranging from 0.10 to 0.50; data set CTC varies the number of correlated taxa from five to twenty; and data set TC varies the total number of taxa in the taxon table from 100 to 500. All data sets contain 500 SNPs each. Data in sets MAF and CTC comprise 1000 individuals; data sets in set TC contain 100 individuals. Data sets MAF and TC all have three correlated taxa per SNP. The MAF for data sets CTC and TC is 0.30.

Human Microbiome Project data. In addition to the synthetic datasets described above, we also tested our method on a real dataset that includes both human genetic and microbiome data [1]. This dataset includes 93 individuals for whom microbiome was profiled as part of the

Human Microbiome Project, and for which host genetic variation information was extracted from shotgun metagenomics sequence data as described previously [1]. We annotated the previously described set of 4.2 million high-quality single nucleotide polymorphisms (SNPs) using ANNOVAR [21] and focused the analysis on a set of 32,696 protein-coding SNPs. We further filtered this set to include only SNPs with minor allele frequency of at least 20% and SNPs for which we had data for at least 50 individuals. The number of SNPs actually tested varies across body sites, ranging from 12,400 to 14,651 SNPs, with a mean of 14,023. For the Stool microbiome data, which included 107 total taxa, running HOMINID on 14,469 SNPs using 12-core Intel Xeon E5-2680 2.50 GHz processors took 16 cpu hours.

Comparison to other methods. The PERMANOVA [22,23] analysis was done in R with the adonis function in the vegan [24] package. The model formula has the SNP genotype as numeric (not factor) predictor variables and the arcsin-sqrt transformed taxon relative abundance table as response variable. The method used to calculate pairwise "distances" was the default Bray-Curtis. The MiRKAT [25] analysis was performed using the MiRKAT package in R. The Bray-Curtis dissimilarity matrix was computed on the arcsin-sqrt transformed taxon table. The matrix was then converted to a kernel matrix, and MiRKAT invoked for each SNP. Since both PERMANOVA and MiRKAT output p-values as measures of how well the taxon abundances correlate with each SNP's genotype (whereas HOMINID outputs R_L^2 values) we chose a cutoff value of p-value such that $q(p_c) = 0.1$ to separate the prediction positives (correlated) from the prediction negatives (uncorrelated), much in the same way we chose the cutoff R_C^2 to separate prediction positive/negative such that $q(R_C^2) = 0.1$ for the Lasso regression.

Results

Analysis using synthetic data. To assess HOMINID's performance, we first used the pipeline on a comprehensive set of synthetic datasets (described above and in the Supplementary Information). These datasets were designed to simulate variation in several important factors, such as variation of the strength of correlation (the input R^2) of the associated SNP with microbiome composition, variation in minor allele frequency (MAF) of the associated SNP, noise level in microbiome data, and the number of taxa associated with the SNP. After analyzing each of the datasets we calculated and plotted the method's sensitivity, specificity, precision, negative predictive value (NPV), false positive rate (FPR), false negative rate (FNR), false discovery rate (FDR), and accuracy, as a function of the input R^2, highlighting the effects of the variable factors above (see **Figures 2A-D**, Supplementary Information and Supplementary Figures S5 - S44).

We found that the strength of correlation (input R^2) between SNP genotype and the correlated taxa has little effect on HOMINID's ability to identify the SNP, unless the correlation is very low (**Figures 2A** and **2B**, Supplementary Information, and Supplementary Figures S5 - S12). HOMINID achieved high sensitivity and specificity for R^2 values of above ~ 0.05. The False Discovery Rate (FDR) is below 0.1 by design, and variation in FDR is due to imprecision (finite number of significant digits) in calculation of R_L^2, and therefore imprecision in calculation

of q. (**Figures 2C** and **2D)**. Similarly, variation in MAF does not affect HOMINID's sensitivity, as data sets with different MAF follow the same behavior (**Figure 2B**).

One of HOMINID's unique features is the ability to identify the taxa that are correlated with an associated SNP. We found that this prediction performs well, with accuracy approaching 1 and a false positive rate (FPR) of 0 for input R^2 values larger than about 0.1, but drops off at lower R^2 values (**Figures 2E** and **2F**, Supplementary Figures S27 and S28). The number of correlated taxa had a noticeable effect, whereby SNPs that correlated with more taxa had higher FPR (compare **Figure 2E** with **Figure 2F**), although in all test datasets' FPR remained < 0.07.

Comparison to other methods. In order to assess HOMINID's performance, we compared it to PERMANOVA [22,23] and MiRKAT [25], two platforms that can be used to identify host SNPs associated with microbiome composition. We note that HOMINID has a unique feature allowing it to identify the specific microbial taxa associated with each SNP. Since other approaches lack this option, the comparison centered around the ability to detect SNPs that are correlated with the microbiome, and not on the detection of correlated taxa. Our analysis included input datasets with various input R^2 values and noise levels (various effect sizes), and compared the sensitivity of each method to detect the associated SNPs. We found that for median input R^2 values (correlation between associated SNP and microbiome composition) of about 0.15 or above the three methods are all highly sensitive (**Figure 3**). However, for lower input R^2 values, HOMINID is more sensitive. Specifically, for the data set with median input R^2 = 0.08 HOMINID's sensitivity is 1, while the sensitivity of MiRKAT and PERMANOVA is 0.19 and

0.29, respectively (**Figure 3**). Similarly, for median input $R^2 = 0.03$ HOMINID's sensitivity is 0.46, while the other methods' sensitivities are 0.

Analysis of Human Microbiome Project data. We ran the HOMINID pipeline on a previously published data of microbiome and host genetic variation from the Human Microbiome Project cohort [1]. We focused our analysis on coding SNPs with minor allele frequency ≥ 0.2, and identified SNPs for which permutation-based q-value ≤ 0.1 and the 95th percentile confidence interval for R^2 does not include zero. To account for population substructure, we ran a second analysis including the genetic principal components (PCs) as additional covariates [18,19]. This resulted in the identification of 11 (regression with genetic PCs as covariates) and 6 (regression without genetic PCs) for a total of 13 unique associations between host SNP and microbiome composition across 15 body sites (see Supplementary Tables S1 and S2, respectively). As can be seen in Figure 4, HOMINID is able to detect SNPs with the expected pattern of association between host genetic variation and the microbiome. For example, for SNP rs2297345 in the gene *PAK7* we detected a correlation between genotype and a single microbial taxon, Propionibacteriaceae (**Figure 4A**). HOMINID can also detect SNPs where multiple taxa are correlated with the same SNP (e.g., SNP rs6032 in **Figure 4B**), as well as more complex patterns of association; for example, for SNP rs230898 in the gene *TEKT3* (**Figure 4C**) genetic variation is positively correlated with one taxon (Clostridia) and negatively with others (Rhodocyclales and Aerococcaceae).

Although HOMINID performs strongly on the data used in this paper, there are several potential limitations to our method. First, since it is especially designed to identify SNPs where a

number of taxa are associated, it might not be optimal for cases where there is a dramatic shift in the microbiome that includes many dozens of taxa. Moreover, since the SNP is used as the response in the HOMINID model, it is difficult to identify epistatic effects, whereby genetic variation in two or more loci interact to affect microbiome composition. Although HOMINID could still be used to detect these interactions, by including all genotype combinations as response variables; however, multiple hypothesis testing could be an issue, especially for microbiome association studies, where samples sizes are currently small relative to GWAS of other complex traits. Nevertheless, HOMINID might be useful for detection of interaction of between candidate loci.

Lastly, we developed a web-based tool for the visualization of host-microbiome interaction network identified in HOMINID, available at http://z.umn.edu/genemicrobe. The website, designed using D3.js with a dedicated MySQL database serving as the back-end, displays a dynamic visualization of host gene-microbiome taxa interaction networks, and allows the user to add and remove nodes (host gene and microbial taxa), adjust the display size and node locations, filter by body sites, and generate figures. Currently, the website includes toy data representing all SNP-microbe associations with a nominal p-value $\leq$ 0.1 in the Human Microbiome Project data described above. We believe that as studies using larger sample sizes materialize (for example, a recent study included 1,514 subjects [13]), we expect this tool to be useful for visualization of much larger number of associations.

Conclusions

We present HOMINID, a framework designed for identifying associations between host genetic variation and microbiome composition. We analyze synthetic data to show HOMINID's overall strong performance, identify specific factors that may affect it, highlight HOMINID's unique features, and show HOMINID's utility with a real dataset. We expect that HOMINID would be useful for studies attempting to characterize the genetic basis of host-microbiome interactions.

Funding

This work is supported in part by funds from the University of Minnesota College of Biological Sciences, The Randy Shaver Cancer Research and Community Fund, Institutional Research Grant #124166-IRG-58-001-55-IRG53 from the American Cancer Society, and a Research Fellowship from The Alfred P. Sloan Foundation. This work was facilitated in part by computational resources provided by the Minnesota Supercomputing Institute.

Figure Legends

Figure 1. Illustration of the HOMINID pipeline

Figure 2. Assessment of HOMINID's performance using synthetic data. Panels **A-D** assess how well HOMINID predicts the SNPs whose genotypes correlate with microbiome abundances, and panels **E** and **F** assess how well HOMINID predicts the specific taxa correlated with an associated SNP. **(A)** Sensitivity as a function of effect size (input R^2) for the data sets with

MAF=0.30. Different colored points and boxplots represent data sets with different noise levels and therefore different effect sizes. (**B**) Same as A with variation in input data MAF values represented by different colored points at each dataset's median input R^2. See Supplemental Figure S30 for a visualization of the same data with boxplots instead of medians. **(C)** FDR as a function of effect size (input R^2) for data sets with just MAF=0.30. (**D**) Same as C with variation in input MAF values represented by different colored points at each dataset's median input R^2. See Supplemental Figure S35 for a visualization of the same data with boxplots instead of medians. **(E)** FPR for the stability selection step (identifying the taxa that associate with a SNP's genotype), as a function of effect size (input R^2) for data sets with three correlated taxa. (**F**) Same as E but with twenty correlated taxa.

Figure 3. Comparison of the performance of HOMINID versus MiRKAT and PERMANOVA. Sensitivity is plotted as a function of effect size (input R^2) for HOMINID (red), MirKAT (green), and PERMANOVA (blue). At high input R^2 all three methods perform well, finding all SNPs that correlate with the microbiome. However, at smaller effect sizes (lower input R^2), HOMINID is more sensitive.

Figure 4. Examples of SNPs where correlations were found between host genetic variation and the microbiome. Three SNPs are shown: **(A)** rs2297345 (correlated with abundance of microbial taxa in the right antecubital fossa), **(B)** rs6032 correlated with abundance of microbial taxa in the throat), and **(C)** rs230898 (correlated with abundance of microbial taxa in the supragingival plaque). The x-axis shows the host SNP genotypes, and the y-axis shows the arcsin sqrt transformed taxon abundances. The different correlated taxa for each SNP are shown in

different colors. See Figure S59 for a visualization of the results in panel 4B, but omitting the highest abundance taxon, Bacteroidetes (dark green), to better display the trends for the three lower abundance taxa.

References

1. Blekhman R, Goodrich JK, Huang K, Sun Q, Bukowski R, Bell JT, et al. Host genetic variation impacts microbiome composition across human body sites. Genome Biol. 2015;16:191.

2. Consortium, Human Microbiome Project. Structure, function and diversity of the healthy human microbiome. Nature. 2012;486:207–14.

3. Goodrich JK, Davenport ER, Waters JL, Clark AG, Ley RE. Cross-species comparisons of host genetic associations with the microbiome. Science. 2016;352:532–5.

4. Morton ER, Lynch J, Froment A, Lafosse S, Heyer E, Przeworski M, et al. Variation in Rural African Gut Microbiota Is Strongly Correlated with Colonization by Entamoeba and Subsistence. PLoS Genet. 2015;11:e1005658.

5. Tong M, McHardy I, Ruegger P, Goudarzi M, Kashyap PC, Haritunians T, et al. Reprograming of gut microbiome energy metabolism by the FUT2 Crohn's disease risk polymorphism. ISME J. 2014;8:2193–206.

6. Khachatryan ZA, Ktsoyan ZA, Manukyan GP, Kelly D, Ghazaryan KA, Aminov RI. Predominant role of host genetics in controlling the composition of gut microbiota. PLoS One. 2008;3:e3064.

7. Knights D, Silverberg MS, Weersma RK, Gevers D, Dijkstra G, Huang H, et al. Complex host genetics influence the microbiome in inflammatory bowel disease. Genome Med. 2014;6:107.

8. Goodrich JK, Waters JL, Poole AC, Sutter JL, Koren O, Blekhman R, et al. Human genetics shape the gut microbiome. Cell. 2014;159:789–99.

9. Goodrich JK, Davenport ER, Beaumont M, Jackson MA, Knight R, Ober C, et al. Genetic Determinants of the Gut Microbiome in UK Twins. Cell Host Microbe. 2016;19:731–43.

10. Davenport ER, Cusanovich DA, Michelini K, Barreiro LB, Ober C, Gilad Y. Genome-Wide Association Studies of the Human Gut Microbiota. PLoS One. dx.plos.org; 2015;10:e0140301.

11. Benson AK, Kelly SA, Legge R, Ma F, Low SJ, Kim J, et al. Individuality in gut microbiota composition is a complex polygenic trait shaped by multiple environmental and host genetic factors. Proc. Natl. Acad. Sci. U. S. A. 2010;107:18933–8.

12. Leamy LJ, Kelly SA, Nietfeldt J, Legge RM, Ma F, Hua K, et al. Host genetics and diet, but not immunoglobulin A expression, converge to shape compositional features of the gut microbiome in an advanced intercross population of mice. Genome Biol. 2014;15:552.

13. Bonder MJ, Kurilshikov A, Tigchelaar EF, Mujagic Z, Imhann F, Vila AV, et al. The effect of host genetics on the gut microbiome. Nat. Genet. [Internet]. Nature Research; 2016 [cited

2016 Oct 5]; Available from: http://dx.doi.org/10.1038/ng.3663

14. Turpin W, Espin-Garcia O, Xu W, Silverberg MS, Kevans D, Smith MI, et al. Association of host genome with intestinal microbial composition in a large healthy cohort. Nat. Genet. 2016;48:1413–7.

15. Pedregosa F, Varoquaux G, Gramfort A, Michel V, Thirion B, Grisel O, et al. Scikit-learn: Machine Learning in Python. J. Mach. Learn. Res. 2011;12:2825–30.

16. Subramanian A, Tamayo P, Mootha VK, Mukherjee S, Ebert BL, Gillette MA, et al. Gene set enrichment analysis: a knowledge-based approach for interpreting genome-wide expression profiles. Proc. Natl. Acad. Sci. U. S. A. 2005;102:15545–50.

17. Meinshausen N, Bühlmann P. Stability selection. J. R. Stat. Soc. Series B Stat. Methodol. Blackwell Publishing Ltd; 2010;72:417–73.

18. Price AL, Patterson NJ, Plenge RM, Weinblatt ME, Shadick NA, Reich D. Principal components analysis corrects for stratification in genome-wide association studies. Nat. Genet. 2006;38:904–9.

19. Pritchard JK, Stephens M, Rosenberg NA, Donnelly P. Association mapping in structured populations. Am. J. Hum. Genet. 2000;67:170–81.

20. Baldridge E, Harris DJ, Xiao X, White EP. An extensive comparison of species-abundance distribution models. PeerJ. 2016;4:e2823.

21. Wang K, Li M, Hakonarson H. ANNOVAR: functional annotation of genetic variants from high-throughput sequencing data. Nucleic Acids Res. 2010;38:e164.

22. Anderson MJ. A new method for non-parametric multivariate analysis of variance. Austral Ecol. Wiley Online Library; 2001;26:32–46.

23. McArdle BH, Anderson MJ. Fitting multivariate models to community data: a comment on distance-based redundancy analysis. Ecology. Wiley Online Library; 2001;82:290–7.

24. Oksanen J, Kindt R, Legendre P, O'Hara B, Stevens MHH, Oksanen MJ, et al. The vegan package. Community ecology package. 2007;10:631–7.

25. Zhao N, Chen J, Carroll IM, Ringel-Kulka T, Epstein MP, Zhou H, et al. Testing in Microbiome-Profiling Studies with MiRKAT, the Microbiome Regression-Based Kernel Association Test. Am. J. Hum. Genet. 2015;96:797–807.

Figure 1 Click here to download Figure fig1.pdf

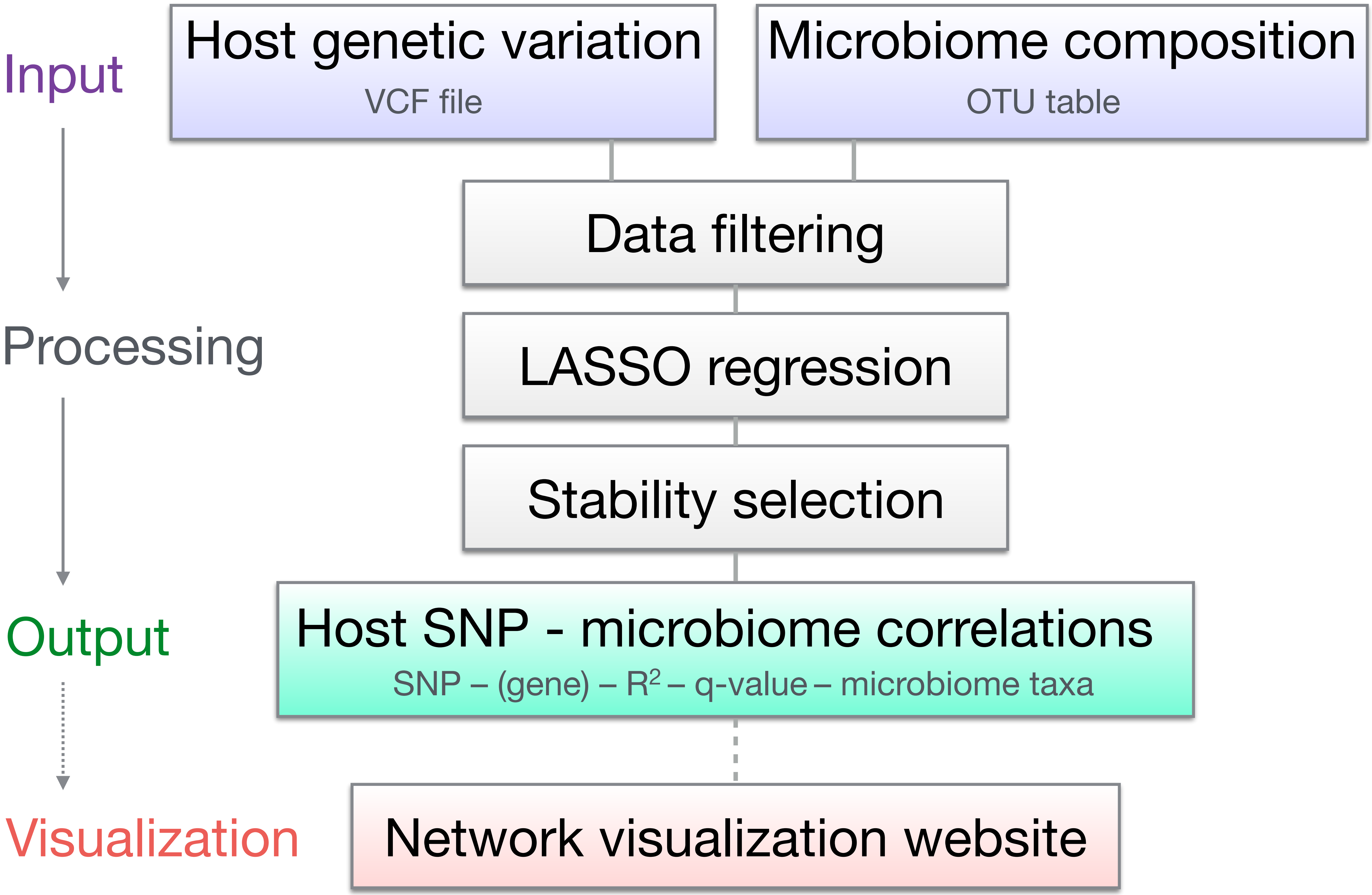

Figure 2

Click here to download Figure fig2.pdf

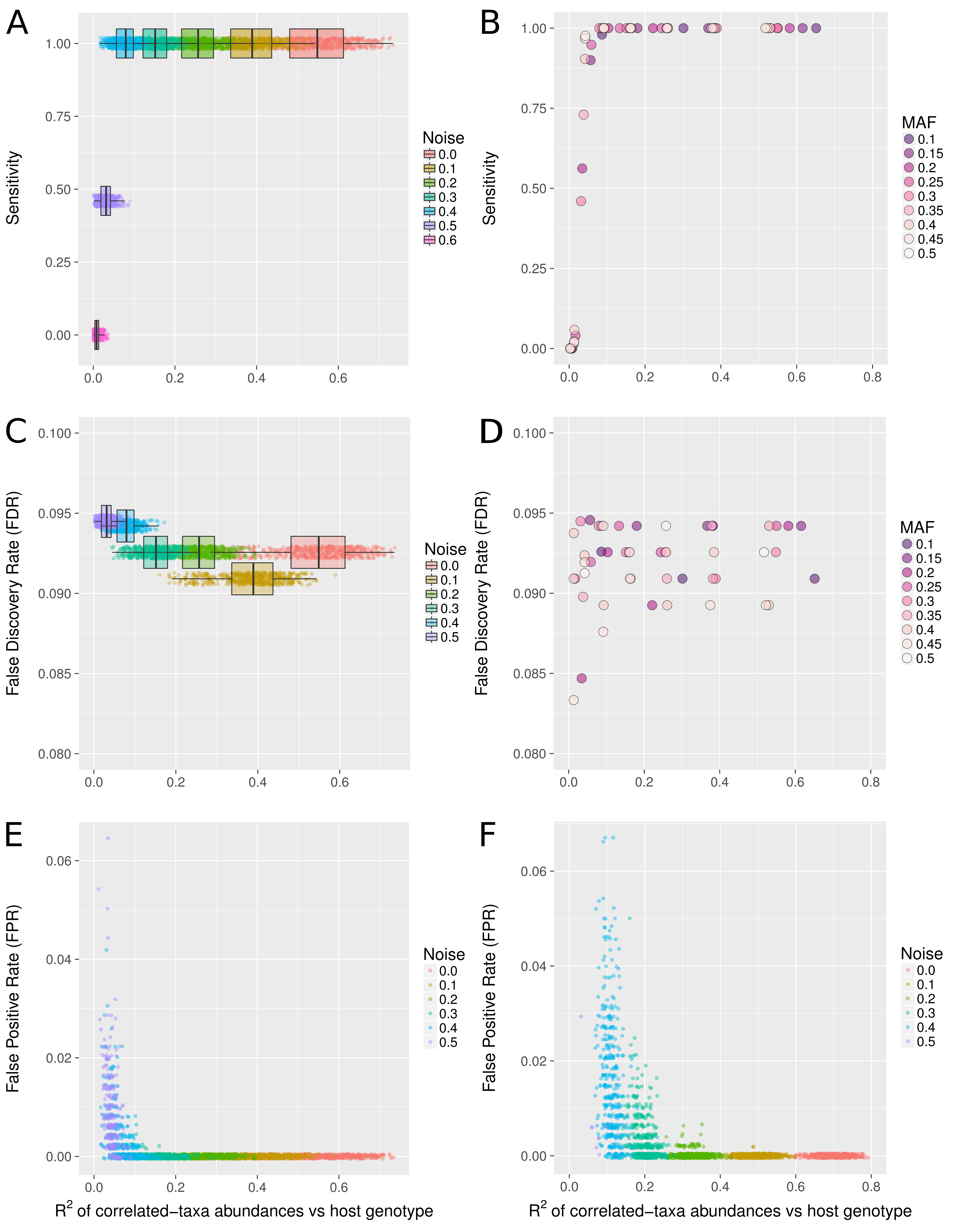

Figure 3

Click here to download Figure fig3.pdf

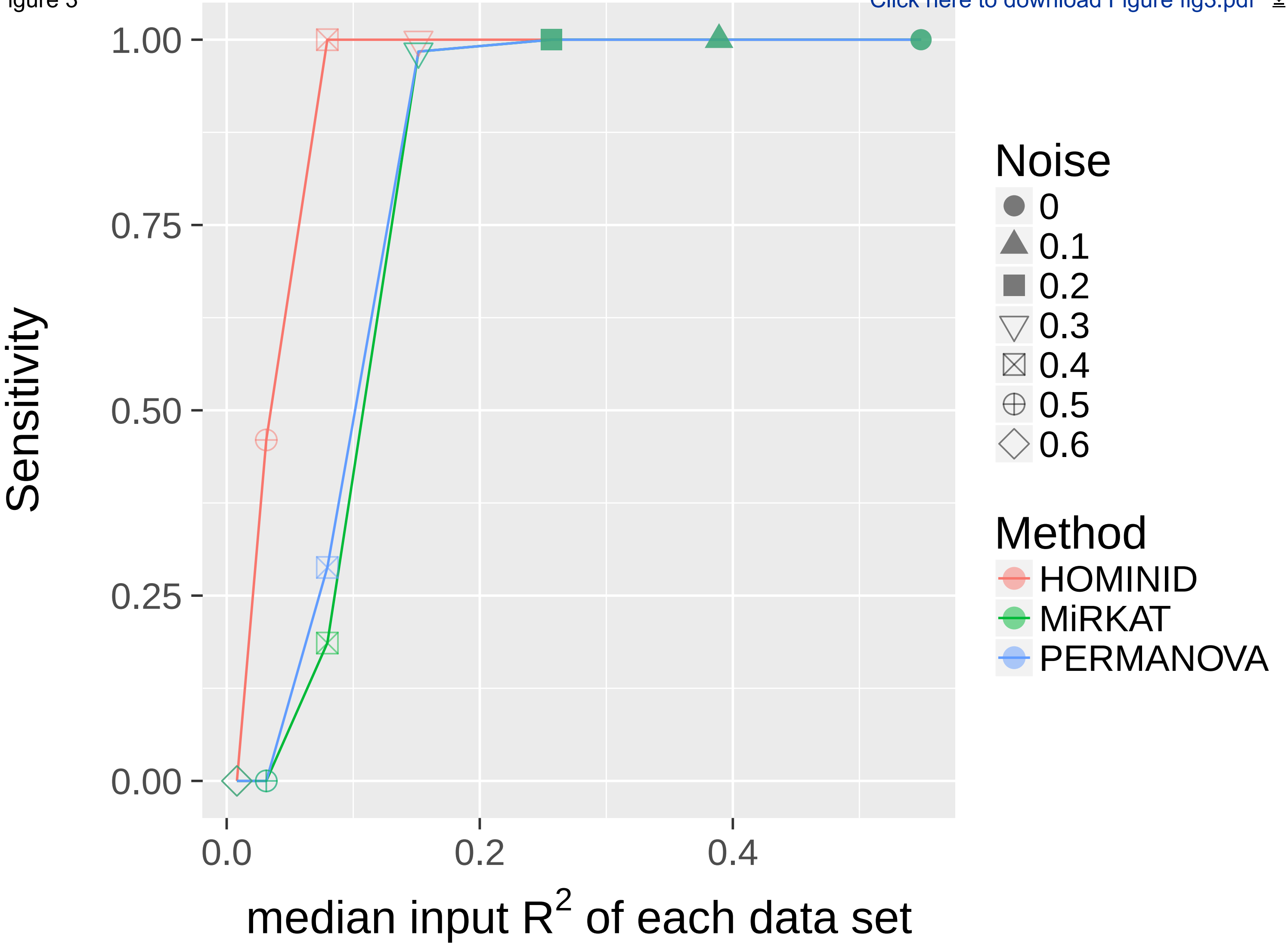

Figure 4

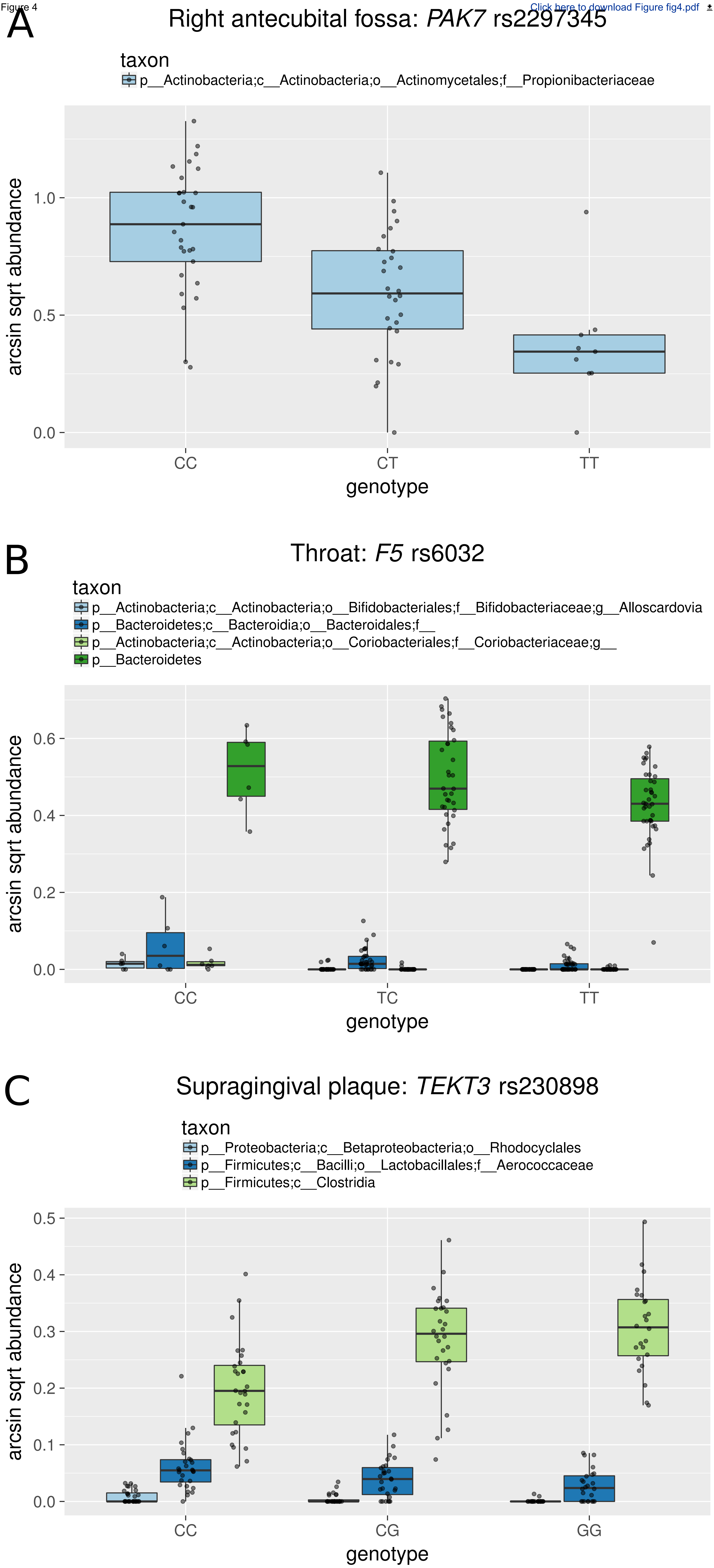
A
Right antecubital fossa: PAK7 rs2297345
taxon
p__Actinobacteria;c__Actinobacteria;o__Actinomycetales;f__Propionibacteriaceae
arcsin sqrt abundance
1.0
0.5
0.0
CC
CT
TT
genotype
B
Throat: F5 rs6032
taxon
p__Actinobacteria;c__Actinobacteria;o__Bifidobacteriales;f__Bifidobacteriaceae;g__Alloscardovia
p__Bacteroidetes;c__Bacteroidia;o__Bacteroidales;f__
p__Actinobacteria;c__Actinobacteria;o__Coriobacteriales;f__Coriobacteriaceae;g__
p__Bacteroidetes
arcsin sqrt abundance
0.6
0.4
0.2
0.0
CC
TC
TT
genotype
C
Supragingival plaque: TEKT3 rs230898
taxon
p__Proteobacteria;c__Betaproteobacteria;o__Rhodocyclales
p__Firmicutes;c__Bacilli;o__Lactobacillales;f__Aerococcaceae
p__Firmicutes;c__Clostridia
arcsin sqrt abundance
0.5
0.4
0.3
0.2
0.1
0.0
CC
CG
GG
genotype

Supplemental Table S1

Click here to access/download
Supplementary Material
supplementalTableS1.xlsx

Supplemental Table S2

Click here to access/download
Supplementary Material
supplementalTableS2.xlsx

Click here to access/download
Supplementary Material
supplementaryInfo.pdf

Cover Letter with Response to Reviewers

Click here to download Personal Cover Lynch.CoverLetter.revision2.withResponse.pdf

University of Minnesota

Ran Blekhman, Ph.D.
Assistant Professor

Genetics, Cell Biology, and Development
Ecology, Evolution, and Behavior

Cargill 222
1500 Gortner Ave.
Saint Paul, MN 55108
Email: blekhman@umn.edu
Tel: (612) 624-4092
Web: BlekhmanLab.org

October 5, 2017

Nicole Nogoy, Ph.D.
Editor
GigaScience

Dear Dr. Nogoy,

Thank you for the thoughtful reviews of our manuscript, "**HOMINID: A framework for identifying associations between host genetic variation and microbiome composition"** (GIGA-D-16-00138)**.** We have prepared a revised version of our manuscript, comprehensively addressing the comments raised by the reviewers during the second review. We have included a detailed description of the modifications, attached to the end of this letter.

Please do not hesitate to contact us if there are any remaining issues or concerns. We are excited to publish our work in *GigaScience*, and would like to thank you again for the positive review experience and helpful feedback that has undoubtedly improved the paper. We look forward to working with you again in the future.

Sincerely,

Ran Blekhman, Ph.D.
Alfred P. Sloan Research Fellow
Assistant Professor
University of Minnesota, Twin Cities
Dept. of Genetics, Cell Biology, and Development | Dept. of Ecology, Evolution, and Behavior
Cargill 222, 1500 Gortner Ave., St. Paul, MN 55108
BlekhmanLab.org | Twitter: @blekhman | Phone: (612) 624-4092 | Fax:(612) 624-6264

HOMINID: A framework for identifying associations between host genetic variation and microbiome composition

Response to 2nd Review - GigaScience manuscript GIGA-D-16-00138

__Review 1__

I really appreciate the authors' tremendous efforts to make their method more rigorous. They have addressed most of my concerns, and I am very pleased to see this new version. Certainly, the work will be a valuable contribution to the field. I only have very minor comments:

1. I could not find the plots of simulated R2 (truth) v.s. the estimated R2 (R^2_L). It 's nice to include a supplementary figure showing the relationship, which will give some idea about the potential bias;

> We agree, and have added this as Figure S4 in the Supplementary Information. We also copy the figure below for convenience. This displays the correlation between the input R^2 (truth) versus the R^2_L output by the HOMINID program. Results are for the dataset with MAF=0.30 and three correlated taxa.

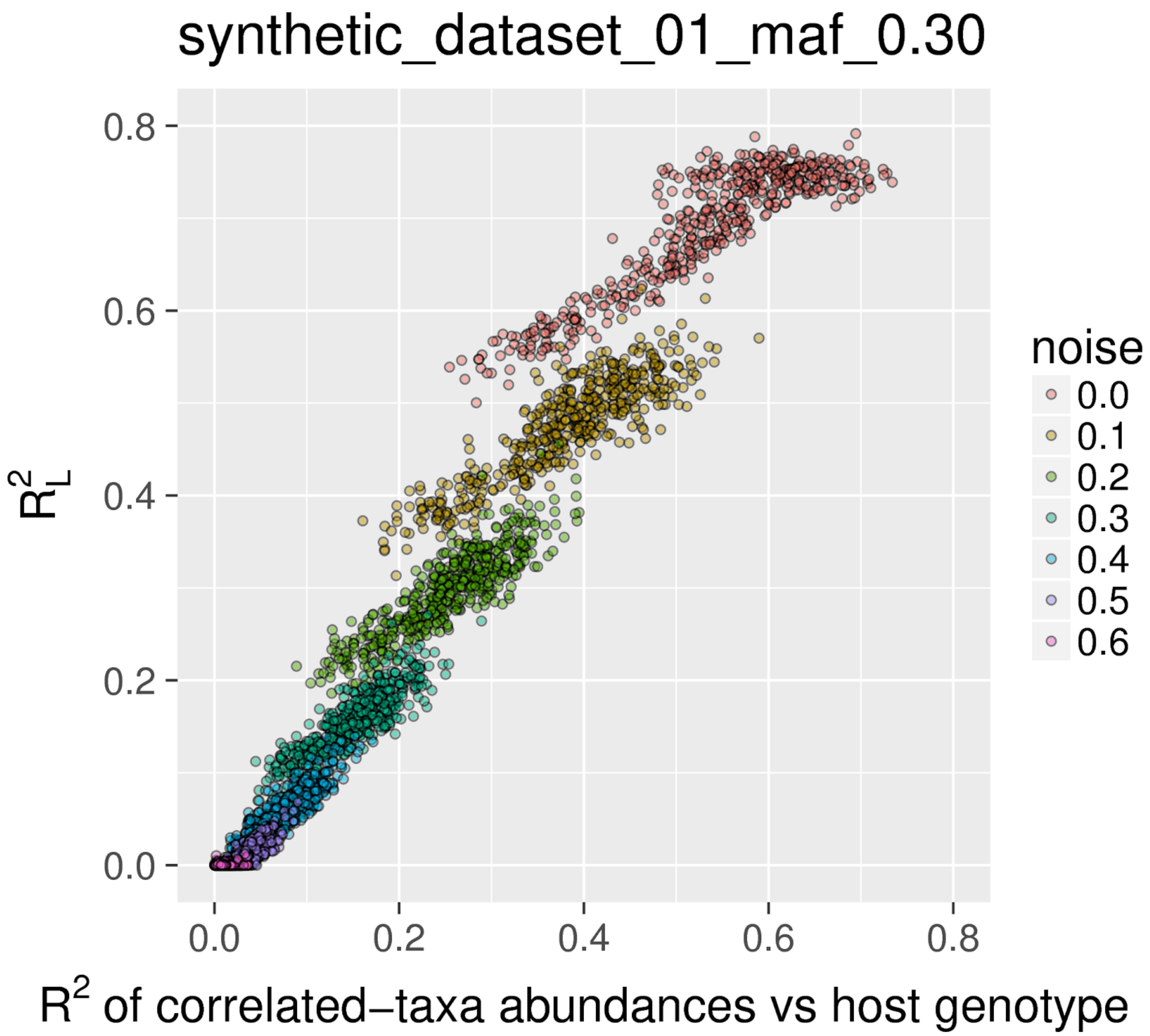

2. The sample splitting for the calculation of R^2_L is still not very clear to me. I assume that the authors performed inner 5-fold CV on the training dataset (4/5 of the data) for tuning parameter selection and model building and used the remaining 1/ 5 of the data for R^2_L calculation (outer CV). The process was repeated by 100 times. If so, it is a nested cross-validation procedure.

We agree that this point wasn't clear and have edited the description of the HOMINID implementation in the main text to read, "How well the host genetics correlates with the

microbiome is measured with the coefficient of determination, R^2_L, **calculated via a nested cross-validation procedure**; R^2_L is the median R^2 from five-fold cross-validation, with 100-times resampling."

3. I would suggest the author use 'alpha' instead of a fixed value such as '0.1' for the q-value cutoff. The users should decide the level of significance to use. I would also recommend the authors to set the default value of 'alpha' as '0.05' in their software.

The HOMINID software outputs the R^2_L values only, for both the real data (unpermuted SNPs) and permuted SNPs. It doesn't produce a list of significant SNPs. Determining the significant SNPs is for the user to do, based on the two sets of R^2_L values and their preferred 'alpha' value.

4. For my previous comment, 'The authors might consider permuting the genotypes and rerun the same procedure. If they can demonstrate the "NULL" model produces way less number of "significant" hits, the results will be more convincing'. What I mean is to permute the sample labels of the HMP data (either SNP data or OTU data) to disrupt the SNP-Microbiome association and repeat the same process as you have done in the manuscript. This serves as a 'sanity check'. If the method is very robust, you should get much less or probably no hits.

This is an important point. To clarify, we do permute the SNP labels to create SNPs whose SNP-Microbiome associations have been disrupted. These permuted SNPs are used to calculate q-values and determine the SNPs that have a significant association with the microbiome (at the alpha level chosen by the user). In the Supplementary Information, we added a Q-Q plot (figure S58 and below) showing the R^2_L values of the permuted and unpermuted (real) SNPs from one body site. This plot is also pasted below.

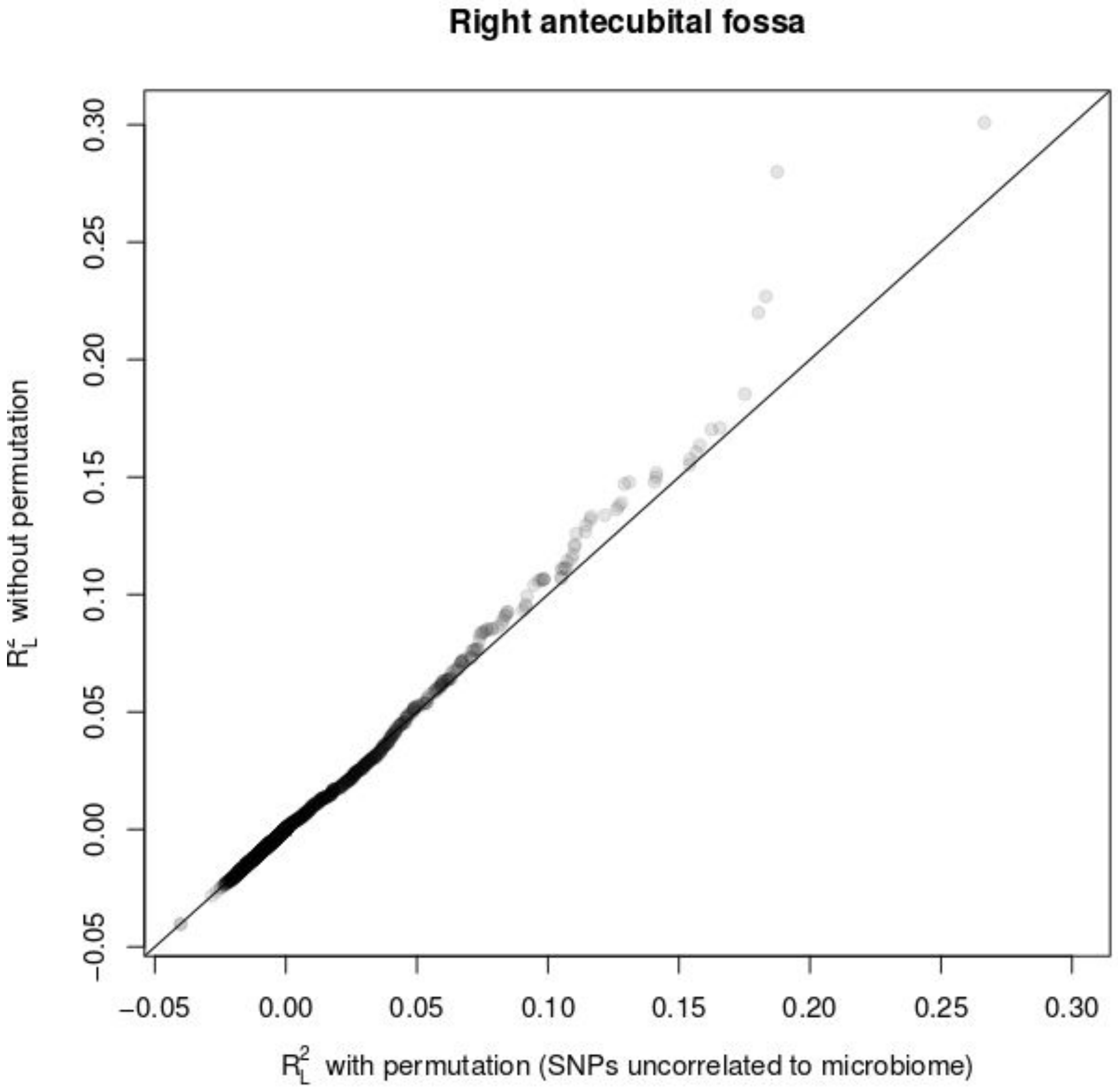

5. In the results section, the authors wrote: "Currently, the website includes toy data representing all SNP-microbe associations with a nominal p-value <= 0.1 in the Human Microbiome Project data described above." However, in this new version, only q-value calculation was described. Did the author mean 'q-value'?

For the purpose of network visualization on the website, we indeed included SNPs with p-value <= 0.1. Since only a few SNPs are found to be significant using the q-value

cutoff, the visualization is not informative; thus, only for visualization purposes, we include a larger set of SNPs using the nominal p-value cutoff.

Review 2

Summary:
The authors propose a new method called HOMINID to identify host SNPs that are associated with taxonomic abundances in the human microbiome. This paper is a resubmission in which the authors made some key modifications including (1) comparison to two existing methods, and (2) and expansion of the range of parameters used for the simulated data set to test the ability of their method. The manuscript is much improved, though we have a few further suggestions that we recommend addressing before acceptance for publication.

Minor Suggestions:

In the introduction, be explicit that taxon abundance (versus presence or some other characteristic) is the quantity that is tested for associations with SNPs or for heritability.

We changed the wording of the introduction to read: "[HOMINID] takes as input host genetic variation data (in a modified VCF format) and microbiome taxonomic composition data (**relative abundance data** as an OTU table) ...."

In the methods, "noise" is defined as "swapping the genotypes of pairs of samples, reducing the correlation between the N_ctc correlated taxa and the host SNP genotypes." In Figure 2, "noise" varies from 0.1 to 0.6. However, we cannot figure out what these numbers correspond to.

We agree that this was not clearly described, and have added an explanation to the section on Synthetic datasets. It now reads, "To examine data sets with smaller effect sizes, “noise” was added to the SNP data by swapping the genotypes of pairs of samples, reducing the correlation between the N_{ctc} correlated taxa and the host SNP genotype. **In datasets with noise level *P*, the probability that a random sample’s genotypes are *not* correlated with the correlated-taxa’s abundances is *P*.**"

In the methods, explain what "ctc" means in N_ctc. This appears to be the number of taxa that are associated with a SNP, but the definition could be clearer.

We changed the wording in the methods to read, "Synthetic SNPs data were generated such that, for each SNP **independently**, N_{ctc} **("ctc" stands for correlated-taxon count)** random taxa's abundances correlate with that SNP's genotype.

It appears that SNPs in the synthetic data set are uncorrelated. Is the idea that these are tag SNPs per LD block? Clarify if they are indeed simulated independently and why.

Yes, this is correct. We clarify in the main text that, "Synthetic SNPs were generated such that, for each SNP **independently**, N_{ctc} **("ctc" stands for correlated-taxon count)** random taxa's abundances correlate with that SNP's genotype.

In the Supplementary Information, section 2.1, we added the extra sentence, "**All SNPs are independent of each other (there is no SNP-SNP correlation), mimicking tag SNPs.**" Section 2.2, was also altered to read, "Synthetic SNP data were generated such that, for each SNP **independently (there are no SNP-SNP correlations)**, N_{ctc} random taxa's abundances correlate with that SNP's genotypes."

We recommend discussing the assumptions for using the q-value multiple testing correction and why it may be a good choice in the presence of correlation between tests (i.e., SNPs with high LD).

We added to the Supplementary Information this paragraph, on pp. 4 & 5:

The program does not account for correlation between SNPs. If there are many SNPs with high linkage disequilibrium (LD) q may be affected. Calculation of $q(R_C^2)$ is determined from the distributions of R_L^2 for both the permuted and unpermuted SNPs. The numerator in the above equation, the fraction of permuted SNPs predicted positive, is unaffected by SNP-SNP correlations because permutation of SNP labels breaks both the SNP-SNP correlations and the SNP-microbiome correlations; thus all permuted SNPs become independent both of each other and of the microbiome. However, the denominator, the fraction of unpermuted SNPs predicted positive, may be affected by SNP-SNP correlations depending on how the correlated SNPs' R_L^2 values are distributed. It's the distribution of SNPs at high R_L^2 that primarily determine $q(R_C^2)$. If the SNPs with high LD have R_L^2 values that are randomly distributed (i.e., follow the same R_L^2 distribution as the low-LD SNPs), the numerator and hence q will be unaffected; if the SNPs with high LD are enriched or depleted at high R_L^2, $q(R_C^2)$ will be affected.

Whether this is the case can be checked after the analysis is done when the user examines the SNPs that are predicted positive.

Bottom of p.5 - In which analysis (synthetic or HMP) are SNPs associated with sex and why?

We clarified in the main text that adding sex as a covariate was done in the HMP analysis: "We performed two analyses using HMP data, one including host genetic PCs as covariates (results in Supplementary Table S1), and one without these covariates (Supplementary Table S2), **both including sex as covariate**."

In the Supplementary Information, we explained that in the HMP analysis, "We also controlled for individual sex, setting the data values in the "taxon" table to 0 or 1 for female or male. Adding sex to the model rather than regressing it out of the predictors allows the Lasso regression process to determine if sex is a relevant predictor on a SNP-by-SNP basis. **None of the SNPs that made a q-value cutoff of 0.2 associated with sex**."

In Figure 2, it is difficult to tell whether there is any trend with MAF, R^2, and FDR/Sensitivity. In the text, you state that there is no trend. Perhaps this figure would be more easily interpretable if each boxplot was a point representing the mean of each category, rather than a distribution (in the legend, please state how many points went into each boxplot distribution). Alternatively, different simulations could be plotted separately. As presented, the overlapping boxplots are difficult to interpret, especially in the second (MAF) column.

We thank the reviewer for this comment, and agree that this was confusing. We changed figures 2B and D so that boxplots are now points plotted at the median input R^2_L of each dataset. The new figure is shown, below. The versions of the figures with boxplots is retained in the Supplementary Information.

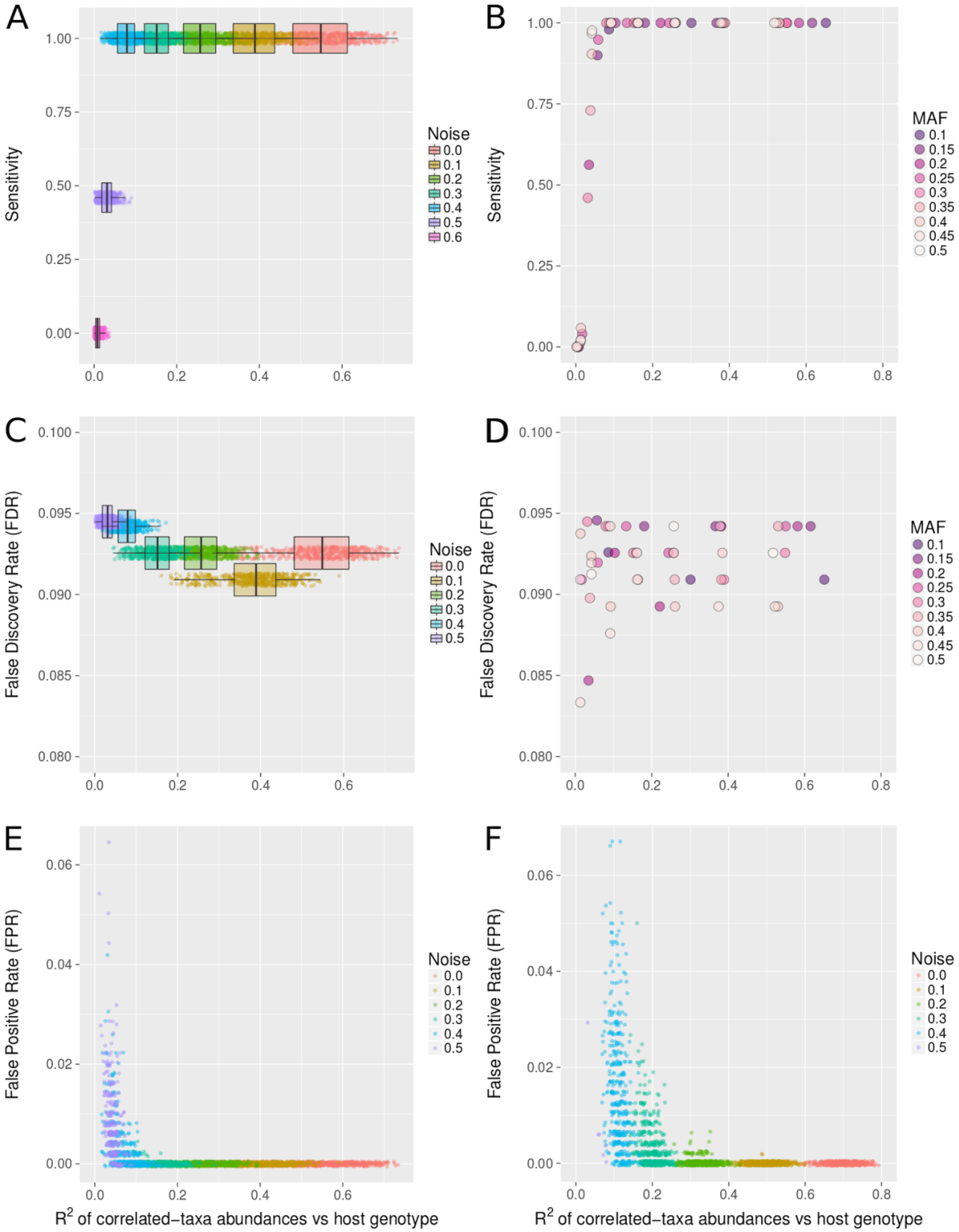
A
B
C
D
E
F
Sensitivity
False Discovery Rate (FDR)
False Positive Rate (FPR)
Noise
0.0
0.1
0.2
0.3
0.4
0.5
0.6
MAF
0.1
0.15
0.2
0.25
0.3
0.35
0.4
0.45
0.5
R² of correlated–taxa abundances vs host genotype

In Figure 2, it seems that MAF has little effect on the results. But later you threshold SNPs in the human microbiome project analysis to have a MAF of at least 0.2. What was the rationale for this?

> We discuss this in the Supplementary Information on p. 75:
>
> **In the HOMINID analysis of HMP data, we focused our analysis on coding SNPs with MAF ≥ 0.2. The rationale for this cutoff is to make sure there is a sufficient number of individuals with each genotype. Specifically, HOMINID needs at least one individual who is homozygous for the minor allele in each of five cross-validation folds. The MAF cutoff that enables this will depend on the total number of individuals included in the analysis—the more individuals, the lower the MAF cutoff can be.**

The reference for Tong, Maomeng et al. 2014 has a typo: Tong, Maomeng shows up twice.

> Thank you for pointing out the error. We've corrected it.

In Figure 4B, it is hard to see the boxplot trends for Alloscardocia and Coriobacterieraceae. Plotting on a log scale may help show the trend better for these bacteria.

> In the Supplementary Information, we've added a second visualization (figure S59, shown below) of the results in panel 4B, but omitting the highest abundance taxon, Bacteroidetes (dark green), to better display the trends for the three lower abundance taxa.

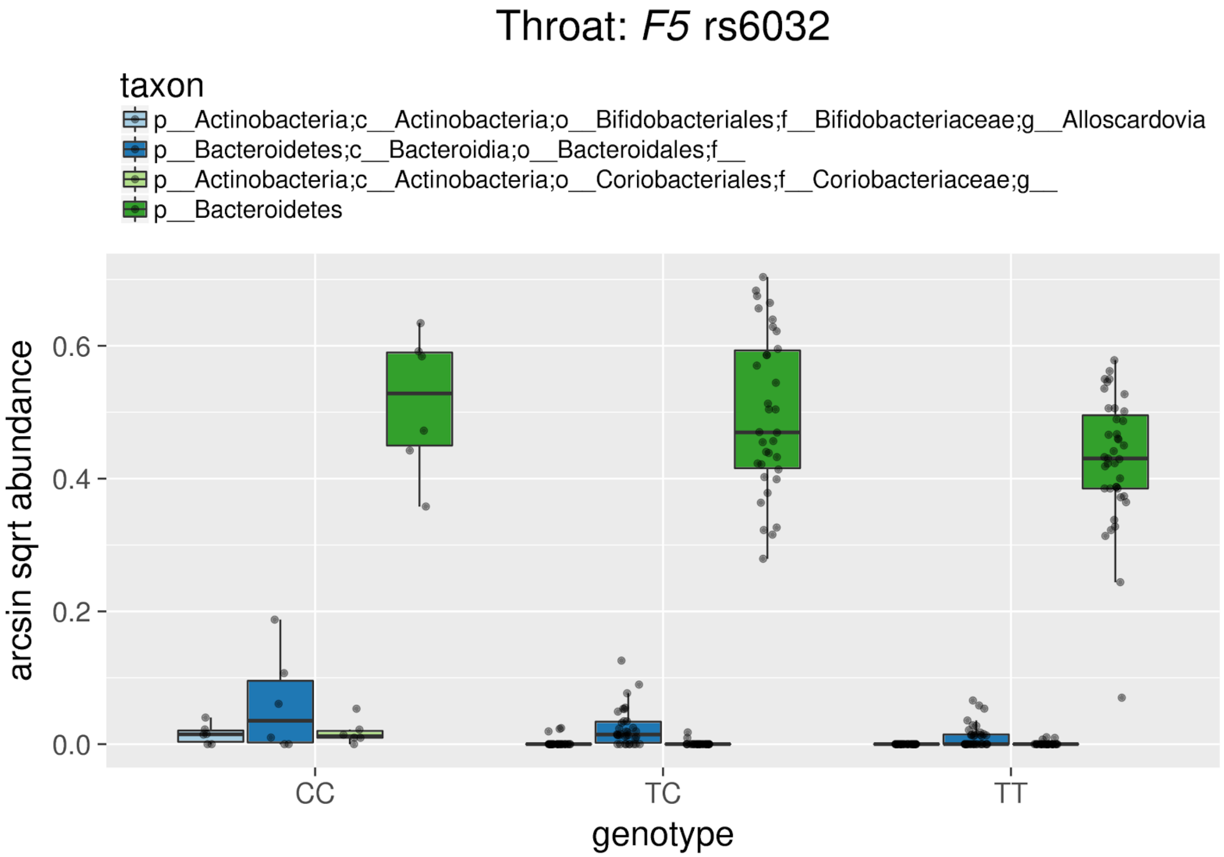
Throat: F5 rs6032
taxon
p__Actinobacteria;c__Actinobacteria;o__Bifidobacteriales;f__Bifidobacteriaceae;g__Alloscardovia
p__Bacteroidetes;c__Bacteroidia;o__Bacteroidales;f__
p__Actinobacteria;c__Actinobacteria;o__Coriobacteriales;f__Coriobacteriaceae;g__
p__Bacteroidetes
arcsin sqrt abundance
0.6
0.4
0.2
0.0
CC
TC
TT
genotype
