# Supplementary material for: HOMINID: a framework for identifying associations between host genetic variation and microbiome composition
Source: Gigascience. 2017 Nov 8;6(12):1–7. doi: 10.1093/gigascience/gix107 (PMC5740987; doi:10.1093/gigascience/gix107)
Supplement: Supplement materials [file gix107_supp.zip › supplementaryInfo.pdf]

HOMINID: A framework for identifying associations between host genetic variation and microbiome composition

Supplementary Information

Joshua Lynch, Karen Tang, Sambhawa Priya, Joanna Sands, Margaret Sands, Evan Tang, Sayan Mukherjee, Dan Knights, Ran Blekhman

Some of the main text has been repeated here for readability and completeness.

1 Methodology and Data

1.1 Lasso Linear Regression Model

We use Lasso regression to find SNPs in which genetic variation is associated with relative abundances of microbial taxa—SNP and taxa combinations where the taxa's relative abundances are correlated with the SNP's genotype. We focused on identifying correlations with an additive pattern, as is common in GWAS and QTL mapping studies.

A simple linear regression model is not appropriate for this task because there are more predictors (taxa) than samples, and because we expect a "sparse" model; at most a few taxa's abundances are expected to correlate with a given SNP's genotypes.

Lasso regression is the same as simple multivariate linear regression but with the addition of a penalty term that shrinks the regression coefficients. Most regression coefficients shrink to zero resulting in a sparse solution.

For n observations of p predictors, $x_{i,j}$, and n responses, y_i ($1 \leq i \leq n$, $1 \leq j \leq p$), Lasso regression, just like the usual least-squares regression, minimizes the residual sum of squares

$$\sum_{i=1}^{n}(y_i - \beta_0 - \sum_{j=1}^{p} \beta_j x_{ij})^2$$

except there is the additional condition that

$$\sum_{j=1}^{p} |\beta_j| \leq t$$

where $t \geq 0$ is a tuning parameter. This is equivalent to the Lagrangian form, where

$$\sum_{i=1}^{n}(y_i - \beta_0 - \sum_{j=1}^{p} \beta_j x_{ij})^2 + \alpha \sum_{j=1}^{p} |\beta_j|$$

is minimized to obtain the Lasso estimates $(\hat{\alpha}, \hat{\beta})$[15, 6]. When the Lasso tuning parameter, α, is zero, the model is identical to the usual multivariate linear regression model which has low bias but large variance. As α increases, fewer predictors (taxa) are retained in the model, creating a sparse model with the sacrifice of a little bias to reduce the variance[15]. Figure S1 shows an example of the effect of increasing α on the Lasso solution.

Here, we present HOMINID (**Ho**st-**mi**crobiome **in**teraction **id**entification), a program utilizing Lasso regression to identify host SNPs correlated with microbiome composition. Our Lasso regression program `HOMINID` (available at `https://github.com/blekhmanlab/hominid`) uses the Python (version 2.7/3.5+) machine-learning library scikit-learn[12] (version 0.16.0) to execute Lasso regression with microbiome

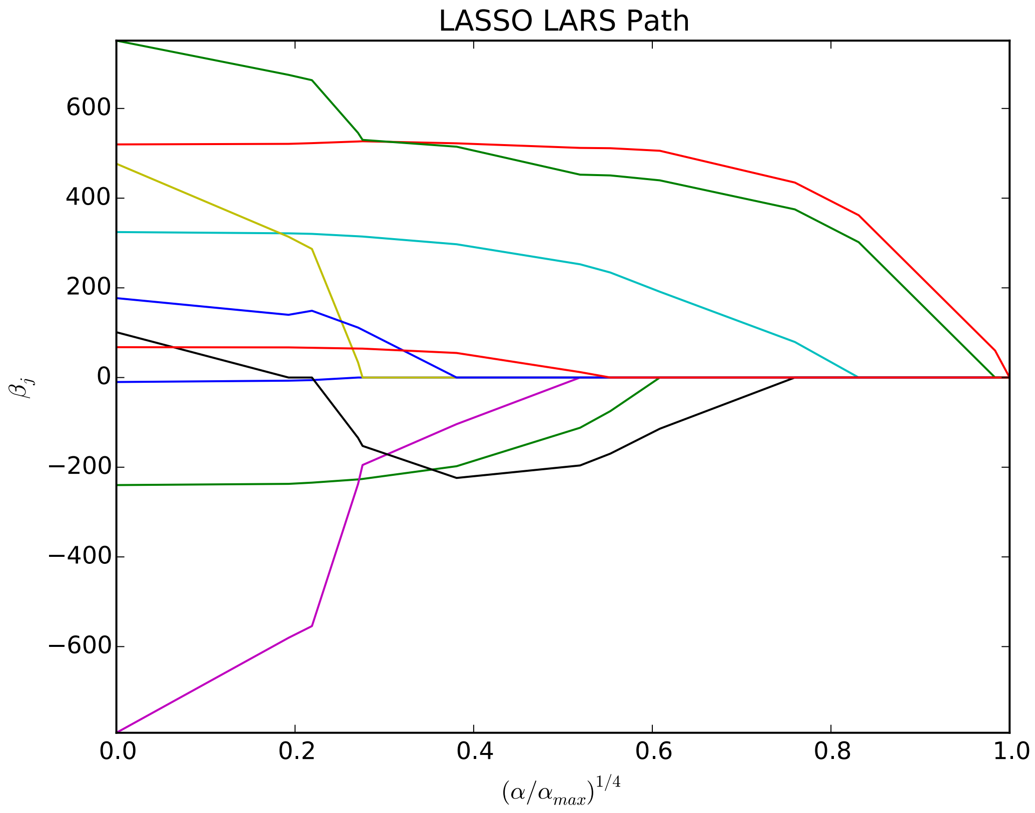

Figure S1: Lasso path for an example dataset with ten predictors, as calculated by the LARS algorithm. Each line corresponds to the regression coefficient, β_j, of a predictor, j. The Lasso parameter, α, is shown on the horizontal axis increasing from left to right. At low values of α all predictors are selected, as indicated by all coefficients having non-zero values. As α increases some coefficients are reduced to zero, removing some predictors from the Lasso model. At the maximum α all predictor coefficients are reduced to zero.

abundances as predictors, and SNP genotype counts as responses. A slightly modified version of the Least Angle Regression (LARS) implements Lasso efficiently [5]. Our program uses LARS with five-fold cross-validation to tune the Lasso parameter α. We use the coefficient of determination, R_L^2,

$$R_L^2 = 1 - \frac{\sum_i (y_i - \bar{y})^2}{\sum_i (y_i - \hat{y}_i)^2}$$

from the Lasso model as a measure of correlation[1]. $\bar{y}$ is the mean of the y_i, and $\hat{y}_i$ is the value predicted by the regression model.

In an early implementation of the algorithm, we observed high variance in R_L^2. Five- and ten-fold cross-validation did not reduce the variance sufficiently to give reproducible results. To reduce the variability in our calculations of R_L^2, we implemented a nested cross-validation procedure, randomly shuffled the samples 100 times (see figure S2) and repeated the Lasso fitting with internal five-fold cross-validation to obtain 100 R_L^2 values. Splits for both the internal five-fold cross-validation and the external 100-times resampling were constructed to maintain approximately the same genotype distribution as the overall data set. We assigned the median of the 100 R_L^2 values to be the R_L^2 for the Lasso regression model and calculated a 95th percentile bootstrap confidence interval of the median using 10,000 bootstrap samples.

To make predictions of which SNPs are prediction positive (predicted correlated to the microbiome) versus prediction negative (predicted uncorrelated to the microbiome), a cutoff value, R_c^2, of R_L^2 is chosen such that the q-value, $q(R_c^2)$, is equal to 0.1. If $R_L^2 \geq R_c^2$, the SNP is predicted positive (correlated); if $R_L^2 < R_c^2$, the SNP is predicted negative (predicted uncorrelated).

The q-value, $q(R_c^2)$, is calculated by permutation tests. For each SNP separately, the SNP sample labels were shuffled, the Lasso regression rerun 10 times with 10 different permutations and the results from all 10 permutation runs combined. To select R_c^2, $q(R_c^2)$ is calculated:

$$q(R_c^2) = \frac{\text{fraction of permuted SNPs predicted positive}}{\text{fraction of unpermuted SNPs predicted positive}}$$

R_c^2 is chosen such that $q(R_c^2) = 0.1$[2]. Note that if the data set has no correlated SNPs, its distribution of R_L^2 values will be the same as that of the permuted SNPs, and $q = 1$. Also, $q \geq$ False Discovery Rate (FDR) and $q \to$ FDR when there are very few condition positives (correlated SNPs) in the data set. By requiring that $q \leq 0.1$, FDR ≤ 0.1.

The program does not account for correlation between SNPs. If there are many SNPs with high linkage disequilibrium (LD) q may be affected. Calculation of $q(R_c^2)$ is determined from the distributions of R_L^2 for both the permuted and unpermuted SNPs. The numerator in the above equation, the fraction of permuted SNPs predicted positive, is unaffected by SNP-SNP correlations because permutation of SNP labels breaks both the SNP-SNP correlations

[1]Because R_L^2 is calculated from a different set of data (the test set) than the model is trained on (the training set), R_L^2 can be negative. Also, we use the subscript L to distinguish the Lasso R^2 from other R^2 values.

[2]In practice, because R_L^2 is calculated to finite precision, R_c^2 is chosen to be the smallest R_L^2 such that $q(R_c^2) \leq 0.1$

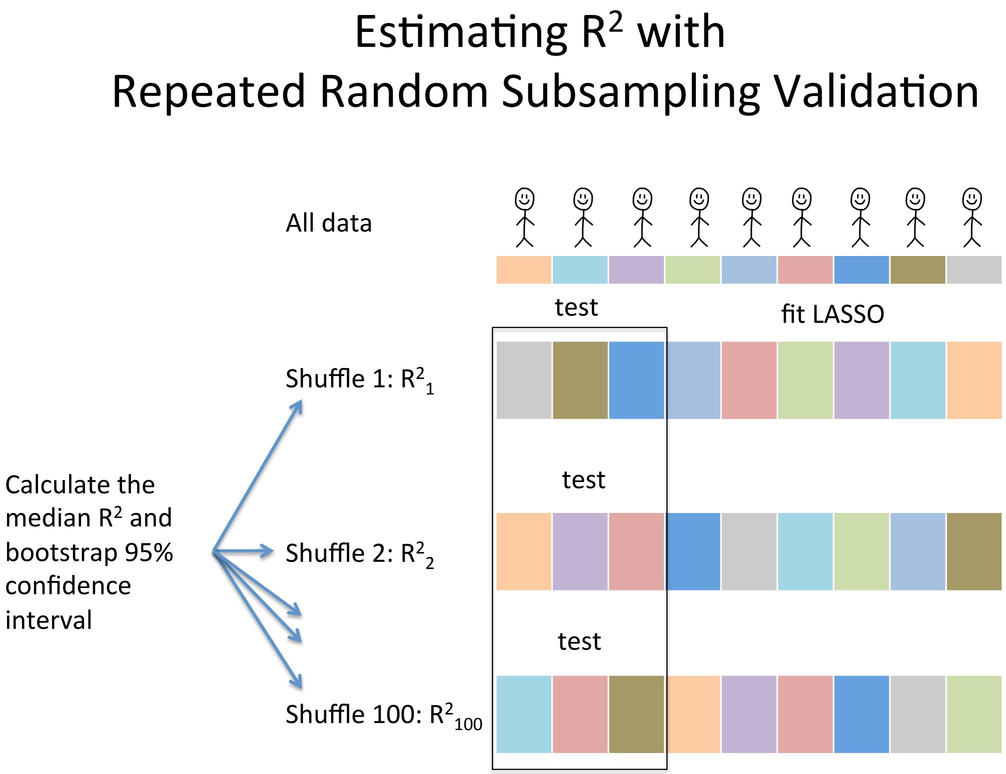

Figure S2: 100 times reshuffling the samples and rerunning the Lasso regression to reduce the variance in R_L^2.

and the SNP-microbiome correlations; thus all permuted SNPs become independent both of each other and of the microbiome. However, the denominator, the fraction of unpermuted SNPs predicted positive, may be affected by SNP-SNP correlations depending on how the correlated SNPs' R_L^2 values are distributed. It's the distribution of SNPs at high R_L^2 that primarily determine $q(R_c^2)$. If the SNPs with high LD have R_L^2 values that are randomly distributed (i.e., follow the same R_L^2 distribution as the low-LD SNPs), the numerator and hence q will be unaffected; if the SNPs with high LD are enriched or depleted at high R_L^2 $q(R_c^2)$ will be affected. Whether this is the case can be checked after the analysis is done when the user examines the SNPs that are predicted positive.

1.2 Selecting Taxa Most Strongly Associated With Host SNP Genotypes

For each host SNP in which genetic variation is significantly correlated to microbial taxon abundances, we identify the taxa most strongly contributing to the correlation. Lasso regression is sensitive to small variations of the predictors [see, e.g., 11] so it is common to use a resampling method to choose relevant predictors (taxa). We used stability selection with randomized Lasso[10]. Stability selection is not a Lasso-specific method but works with any regularized model-fitting algorithm. One advantage of stability selection is that it is less sensitive to the choice of the Lasso parameter, α, than other predictor-selection methods[10].

Randomized Lasso is a slight variation on the usual Lasso in that it incorporates random scaling factors in the penalty term:

$$\sum_{i=1}^{n}(y_i - \beta_0 - \sum_{j=1}^{p}\beta_j x_{ij})^2 + \alpha \sum_{j=1}^{p}\frac{|\beta_j|}{W_j}$$

The W_j are drawn randomly from the interval $[\gamma, 1]$ for a fixed "weakness" $\gamma \in (0, 1]$. The stability selection model works this way:

1. Choose a set of Lasso tuning parameter values $A = \{\alpha_1, \alpha_2, \cdots, \alpha_m\}$ where the α_i are in the neighborhood of the "best" Lasso α parameter.
2. For each $\alpha \in A$, repeat N times:
   (a) Randomly choose a subset of the data.
   (b) Fit the model to the data subset using α_i.
   (c) Record the selected predictors.
3. Count the frequency, f_p, each predictor was selected over all trials. f_p is the stability score for predictor (taxon) p.
4. Choose the predictors (taxa) that are selected in most trials, those whose stability score exceeds a stability-score threshold, $f_p > F$.

The idea here is that, when repeatedly perturbing the α and regression coefficients, β_j, if the same predictors are selected even when the odds are against them, then they are robust predictors. This variation of the Lasso model is only useful in a setting such as stability selection where the model is repeatedly fit and the results are combined. A simple example of a stability-score path is shown in figure S3.

We implemented stability selection (program `stability_selection.py` at `https://github.com/blekhmanlab/hominid`) with randomized Lasso using the python scikit-learn library[12] (version 0.16.0) with the default value of γ. It is necessary to provide a range of values of the Lasso tuning parameter, α, so we repeated the process of model fitting with LARS and 100-times random shuffling to get a series of α's in the neighborhood

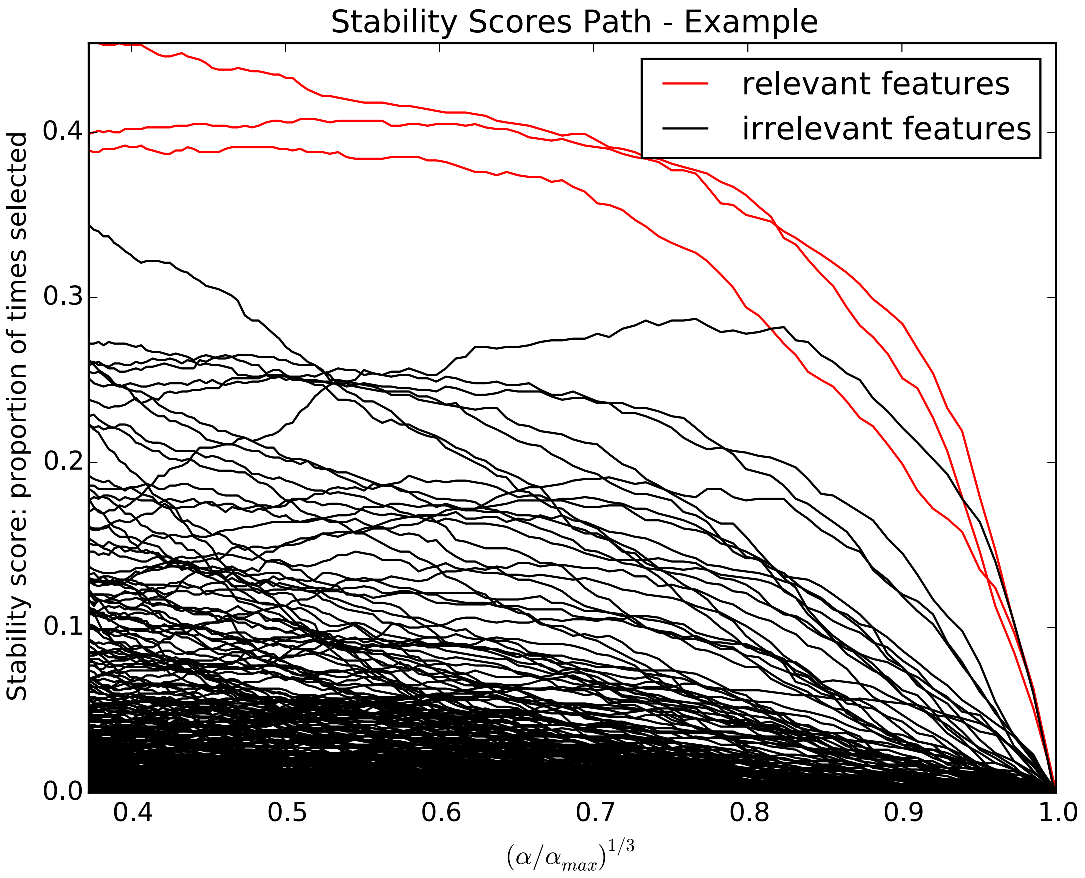

Figure S3: Stability paths for an example dataset with 500 predictors, three of which (shown in red) are correlated to the response. Each line represents the fraction of trials that a predictor is selected in the stability-selection step, as a function of the Lasso tuning parameter α. As α decreases from right to left, the Lasso regression behaves more like regular regression and more variables are selected. The three relevant predictors (red lines) are clearly selected more often than the irrelevant predictors (black lines).

of the “best” α. We kept the largest α, α_{max}, and constructed a set of ten evenly spaced values ranging from $0.3\alpha_{max}$ to α_{max}. This set contains relatively large values of α to favor retaining few predictors in each model. During the stability selection step, we ran $N = 1000$ trials per α_i. We randomly selected 80% of the data for each trial keeping the distribution of genotypes the same as in the entire data set.

We chose the stability-score threshold, F, to be 0.5, so that associated taxa are those selected in at least half the trials during the stability-selection step. With $F = 0.5$, almost all significant SNPs have at least one associated taxon, and most SNPs have only a few associated taxa (average[3] and maximum number of associated taxa for significant SNPs: 2.6 and 11, respectively).

[3]omitting SNPs with zero associated taxa

1.3 Controlling For Other Covariates

Other non-taxon-abundance covariates can also be controlled for using our software by adding the covariate to the taxon table as if it were another taxon.

To test whether population structure has an effect on the performance of HOMINID we conducted a second HOMINID analysis by also controlling for population substructure: The first five principal components (PCs) of the host genetic variation data [13, 14] were included as additional covariates in the Lasso regression. Supplementary Tables S1 and S2 report the HOMINID results with and without the genetic PCs as covariates, respectively. Any SNP that has a genetic PC as a significantly associated covariate was not included in Supplemental Table S1.

We also controlled for individual sex, setting the data values in the "taxon" table to 0 or 1 for female or male. Adding sex to the model rather than regressing it out of the predictors allows the Lasso regression process to determine if sex is a relevant predictor on a SNP-by-SNP basis. None of the SNPs that made a q-value cutoff of 0.2 associated with sex.

1.4 Identifying SNPs Associated with the Microbiome Composition

SNPs associated with the microbiome were defined as SNPs for which:

- SNPs whose 95th percentile confidence interval for R_L^2 does not encompass zero.
- $R_L^2 \geq R_c^2$ (ensuring that $q \leq 0.1$).
- At least one associated taxon meets the stability-score threshold.
- No genetic PC is a significant predictor, in the analysis where genetic PCs are included as covariates (see section 1.3).

1.5 HMP 16S Taxon Tables

The Human Microbiome Project [7, 8] (HMP) has collected and published microbiome data, derived from 16S rRNA gene sequencing, from several hundred healthy individuals at several body sites. OTU tables of taxonomic relative abundances were obtained from the HMP and included microbial taxa at the genus level. We only considered the fifteen HMP body sites that are not female-specific: anterior nares, attached keratinized gingiva, buccal mucosa, hard palate, left antecubital fossa, left retroauricular crease, palatine tonsils, right antecubital fossa, right retroauricular crease, saliva, stool, subgingival plaque, supragingival plaque, throat, and tongue dorsum. We processed these tables with the QIIME[4] (version 1.8.0) command `summarize_taxa.py -u 0.00005` to produce taxon tables including all taxonomic levels from genus to phylum, and trimming off OTUs that make up less than 0.005% of the total dataset. The remaining abundance tables were clustered (separately for each body site) using K-medoids clustering with a clustering threshold of 0.95 correlation. We retained the

medoid taxon of each cluster for the final table. The final taxonomic abundance data used here included an average of 133 taxa per body site (range: 71–303 taxa) in fifteen body sites. Taxon relative abundances were transformed with the arcsin sqrt function.

1.6 HMP Genetic Variation Data

Although host genetic variation was not profiled as part of the original HMP study, we were previously able to mine the deep shotgun metagenomics data generated by HMP for host DNA reads, and characterize host genetic variation for 93 individuals for whom bacterial abundance data are also available [3]. We annotated the previously described set of 4.2 million high-quality single nucleotide polymorphisms (SNPs) using ANNOVAR [16] and focused the analysis on a set of 32,696 protein-coding SNPs. We further filtered this set to include only SNPs with minor allele frequency of at least 10% and SNPs for which we had data for at least 50 individuals. The number of SNPs actually tested varies across body sites, ranging from 12400 to 14651 SNPs, with a mean of 14023.

2 Synthetic Data Results

2.1 Synthetic Data Sets

To assess HOMINID's performance and to compare its performance to those of PERMANOVA [1, 9] and MiRKAT [17] we created several synthetic data sets in which the SNP genotypes are chosen in such a way as to correlate with the abundances of select "taxa" of the synthetic "microbiome".

To assess how well HOMINID performs as a function of (a) minor allele frequency, (b) the number of correlated taxa, and (c) the total number of taxa in the taxon table, we created three different sets of synthetic data which vary minor allele frequency (data set MAF), number of correlated taxa or Correlated-Taxon Count (data set CTC), and total number of taxa in the taxon table, or Taxon Count (data set TC).

Each synthetic data set is composed of 500 correlated SNPs and 10,000 uncorrelated SNPs. All SNPs are independent of each other (there is no SNP-SNP correlation), mimicking tag SNPs. How the taxon table and correlated-SNP data were generated is described in sections 2.2 through 2.5.

2.2 Constructing Synthetic Data Set MAF

This synthetic data set was created to examine the effect of the minor allele frequency (MAF) on HOMINID's performance.

To create the taxon table, the absolute abundances (the taxon abundance "counts") were drawn from a log-series distribution, which is frequently used to represent species abundances

(see, e.g., [2]). The probability of abundance "count", k, follows this functional form

$$P(k) = \frac{-p^k}{k \ln(1-p)}$$

with $p = 0.99$. Typical taxon tables have many counts of 0 (taxon not present or not detected); for example, in the fifteen HMP body sites, 25% (tongue dorsum) to 73% (left antecubital fossa) of abundances are zero. However, $P(k)$ is not defined for $k = 0$. Therefore, after selecting the taxon abundances from the log-series distribution, subtract 1 from each count to include zeroes in the abundance "counts". 21% of abundances in the synthetic taxon table are abundance "count" of 0. To run HOMINID, the absolute abundances were converted to relative abundances.

Each synthetic SNP table is characterized by minor allele frequency, q_{maf}; number of samples (corresponding to human subjects), S; number of taxa correlated to the SNP's genotype, N_{ctc}; and noise level P ($0 \leq P < 1$). Synthetic SNP data were generated such that, for each SNP independently (there are no SNP-SNP correlations), N_{ctc} random taxa's abundances correlate with that SNP's genotypes. To create this correlation, for each SNP, assign genotypes to each sample in this manner:

1. Randomly select N_{ctc} taxa to correlate to the SNP's genotype.

2. For each correlated-taxon's abundance, t_i, randomly select its coefficient, c_i, from a standard normal distribution (mean 0, standard deviation 1).

3. Define the linear function $f(s)$ for that SNP and for sample, s:

$$f(s) = \sum_{i}^{N_{ctc}} c_i t_{i,s}$$

   where the sum is over all correlated taxa, i.

4. Evaluate $f(s)$ for all samples, s, and assign SNP genotypes according to the Hardy-Weinberg principle. Assign genotype 0 to the samples with $f(s)$ in the lower $(1-q_{maf})^2$ percentile, genotype 2 to the samples with $f(s)$ in the upper ${q_{maf}}^2$ percentile, and SNP genotype 1 to the remaining samples.

In this way, that SNP's genotype correlates with the correlated-taxa's abundances. The above creates a no-noise (noise 0.0) data set.

After choosing the genotypes for each SNP and sample pair, we calculate the coefficient of determination, R^2_{OLS}, for an ordinary least squares multiple regression between the correlated taxa's abundances and the SNP genotypes. Since R^2_{OLS} is a characteristic of the input data before analysis by HOMINID, PERMANOVA or MiRKAT, we call it the "input R^2" to distinguish it from the R^2 output by the HOMINID Lasso regression (aka the "output R^2" or R^2_L). R^2_{OLS} is a measure of the effect size.

Since we want to evaluate how HOMINID performs when the effect size/input R^2 is small, we introduce increasing levels of noise to drive R^2_{OLS} lower until $R^2_{OLS} \to 0$. In data sets with noise $P > 0$, the probability that a random sample's genotypes are *not* correlated with the correlated-taxa's abundances is P. We achieve this by selecting pairs of samples with different genotypes and swapping their genotypes. For noise level P, $0.5 \times P \times S$ sample pairs' genotypes are swapped. The larger P, the lower the input R^2.

The MAF synthetic data included 61 data sets, each containing 500 synthetic "taxa", three of which correlate to the SNP genotypes (and the remaining 497 are uncorrelated), and 1000 individuals. The minor allele frequencies of the different SNP files are 0.10, 0.15, 0.20, 0.25, 0.30, 0.35, 0.40, 0.45, and 0.50. The noise level required to drive $R^2_{OLS} \to 0$ depended on the MAF; maximum noise levels were $P = 0.3$ (MAF 0.10), 0.4 (MAF 0.15), 0.5 (MAF 0.20), 0.6 (MAF 0.25 and 0.30), and 0.7 (MAF 0.35, 0.40, 045, and 0.50).

2.3 Constructing Data Set CTC

These datasets were created to examine the effect of the number of correlated taxa or Correlated-Taxon Count (CTC) on HOMINID's performance.

The data were constructed in the same way as for the MAF synthetic data (section 2.2). Each synthetic data set comprises 500 taxa, 1000 samples, and MAF 0.30. The number of correlated taxa of the different SNP files are 5, 10, 15, and 20. The noise level required to drive $R^2_{OLS} \to 0$ was $P = 0.6$.

2.4 Constructing Data Set TC

This synthetic data set was created to examine the effect of the total number of taxa or Taxon Count (TC) on HOMINID's performance.

The data were constructed similarly to the MAF synthetic data except that the abundance distribution obeys an exponential transform of a random value drawn from a normal distribution with mean 0.0 and standard deviation 1.0.

Each synthetic data set comprises 100 samples with MAF 0.30. The taxon tables contain 100, 300, and 500 taxa, of which three are correlated to the SNP (and the remaining taxa uncorrelated). We examined three noise levels, 0.0, 0.05, and 0.10.

2.5 Constructing the Uncorrelated SNPs

The uncorrelated SNPs were created by permuting the sample IDs (permuted separately for each SNP). The minor allele frequency was kept the same; noise level is irrelevant (since noise is introduced by swapping the genotypes of sample pairs, which has the same effect as swapping sample IDs).

2.6 HOMINID's Performance as a Function of Input R^2

To see how HOMINID performs as the signal weakens (as the input R^2 gets smaller), we examine the data set with MAF=0.30, 500 total taxa, three of which are correlated and 497 uncorrelated.

Figure S4, comparing the input R^2 (R^2_{OLS}, "truth") versus the output R^2 (R^2_L, predicted), shows a strong correlation indicating that R^2_L is a good measure of the degree of correlation between microbiome and SNP genotype.

In figure S5, we plot the False Negative Rate ($FNR = FN/(TP+FN) = 1 - \text{sensitivity}$) as a function of input R^2. The data sets with different noise levels, and hence different ranges of R^2_{OLS}, are plotted as separate boxplots and in different colors. Each colored point represents a SNP at a particular noise level.

The FNR is zero—indicating that all correlated SNPs were correctly predicted to be correlated—until $R^2_{OLS} \lesssim 0.05$.

In figures S6, S7, S8, S9, S10, S11, and S12, are plotted other metrics of HOMINID's performance, as a function of input R^2:

- sensitivity ($= TP/(TP + FN) = 1 - FNR$)
- specificity ($= TN/(TN + FP)$)
- precision ($= TP/(TP + FP)$)
- negative predictive value ($NPV = TN/(TN + FN)$)
- false positive rate ($FPR = FP/(FP + TN) = 1 - \text{specificity}$)
- false discovery rate ($FDR = FP/(TP + FP) = 1 - \text{precision}$)
- accuracy ($= (TP + TN)/(TP + FP + FN + TN)$)

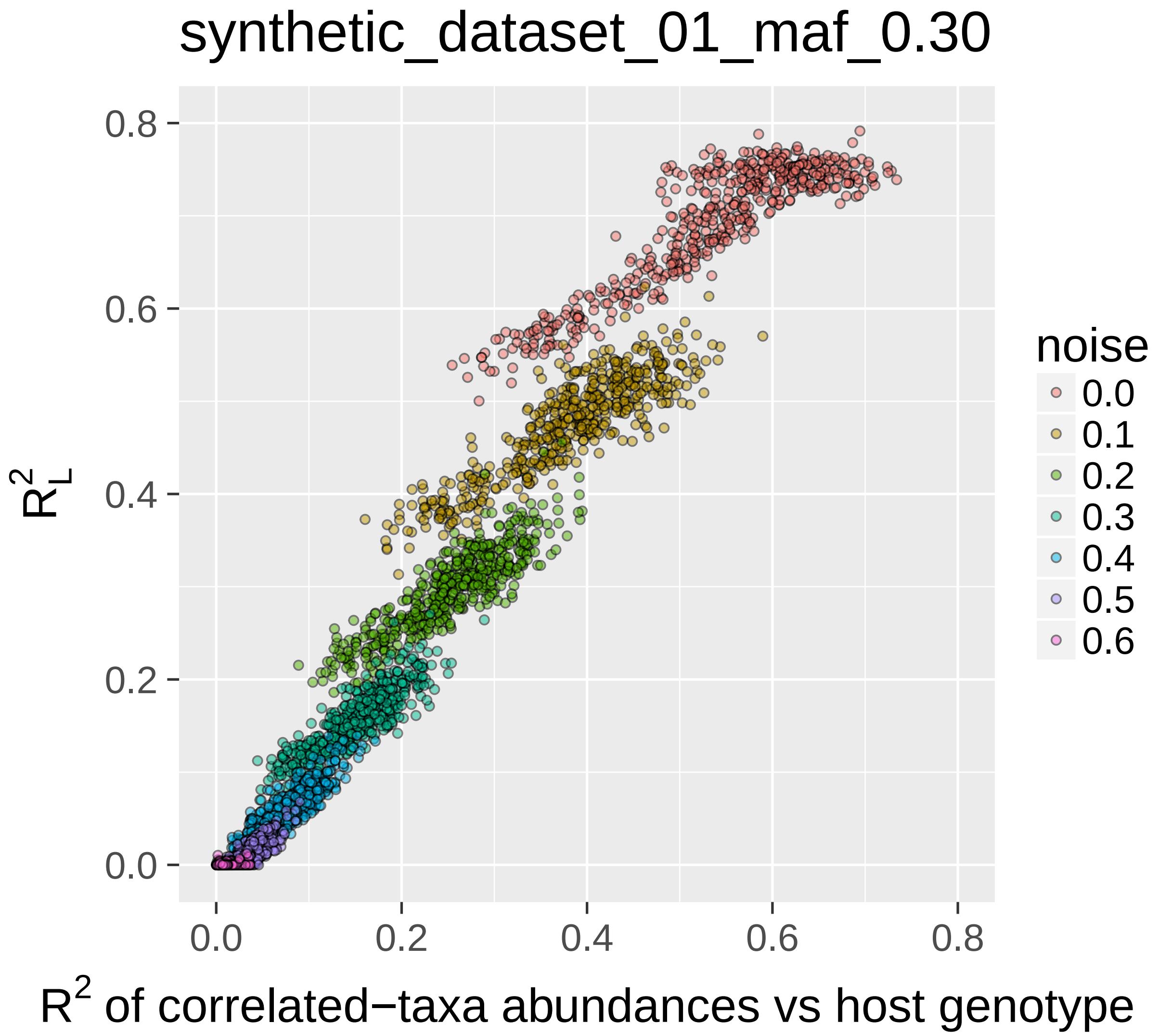

Figure S4: Simulated input R^2 (R^2_{OLS}, on the x-axis) versus output R^2 (R^2_L, on the y-axis).

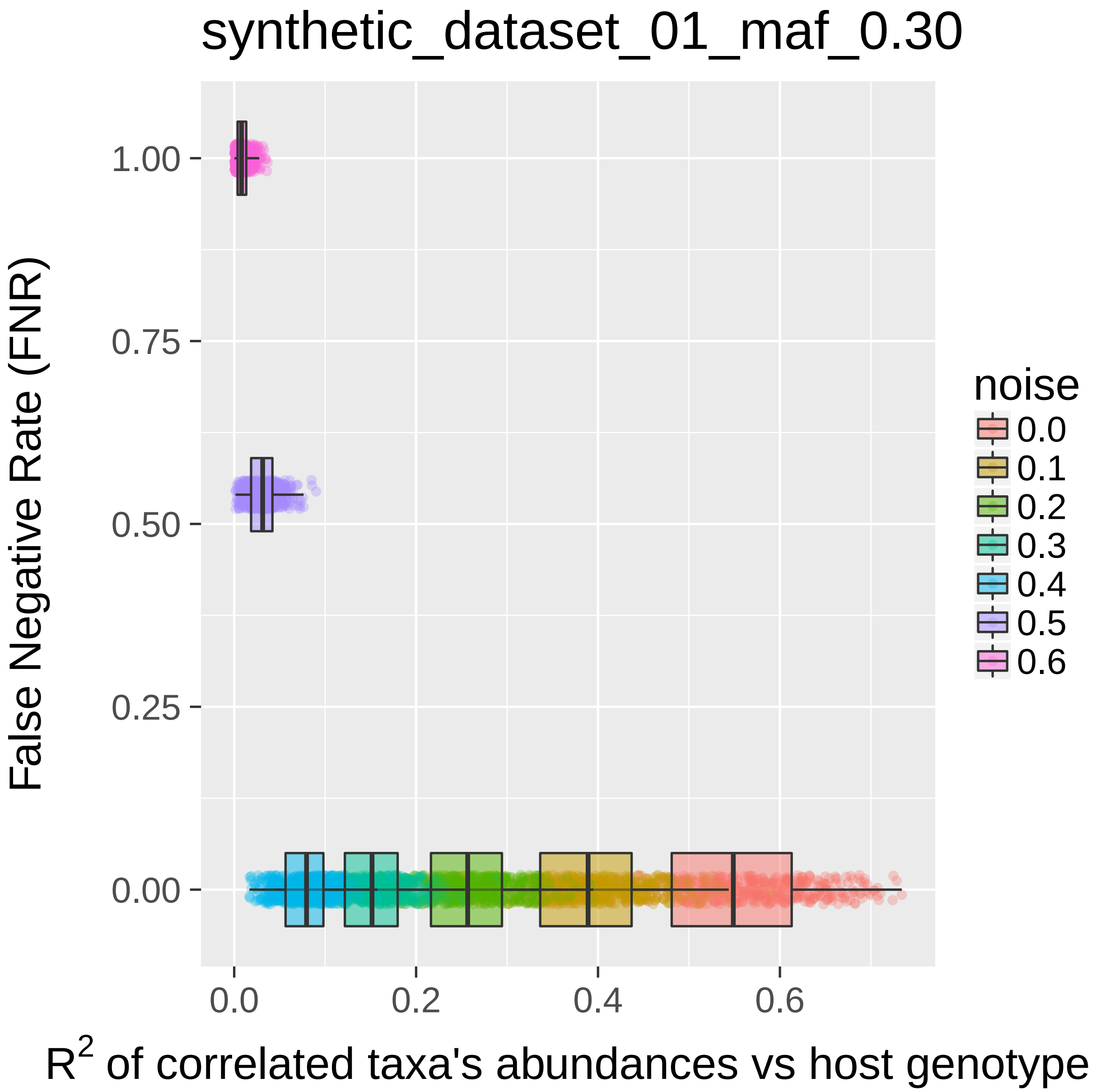

Figure S5: False Negative Rate (FNR) as a function of input R^2. All correlated SNPs are found (FNR = 0), except when R^2_{OLS} is low.

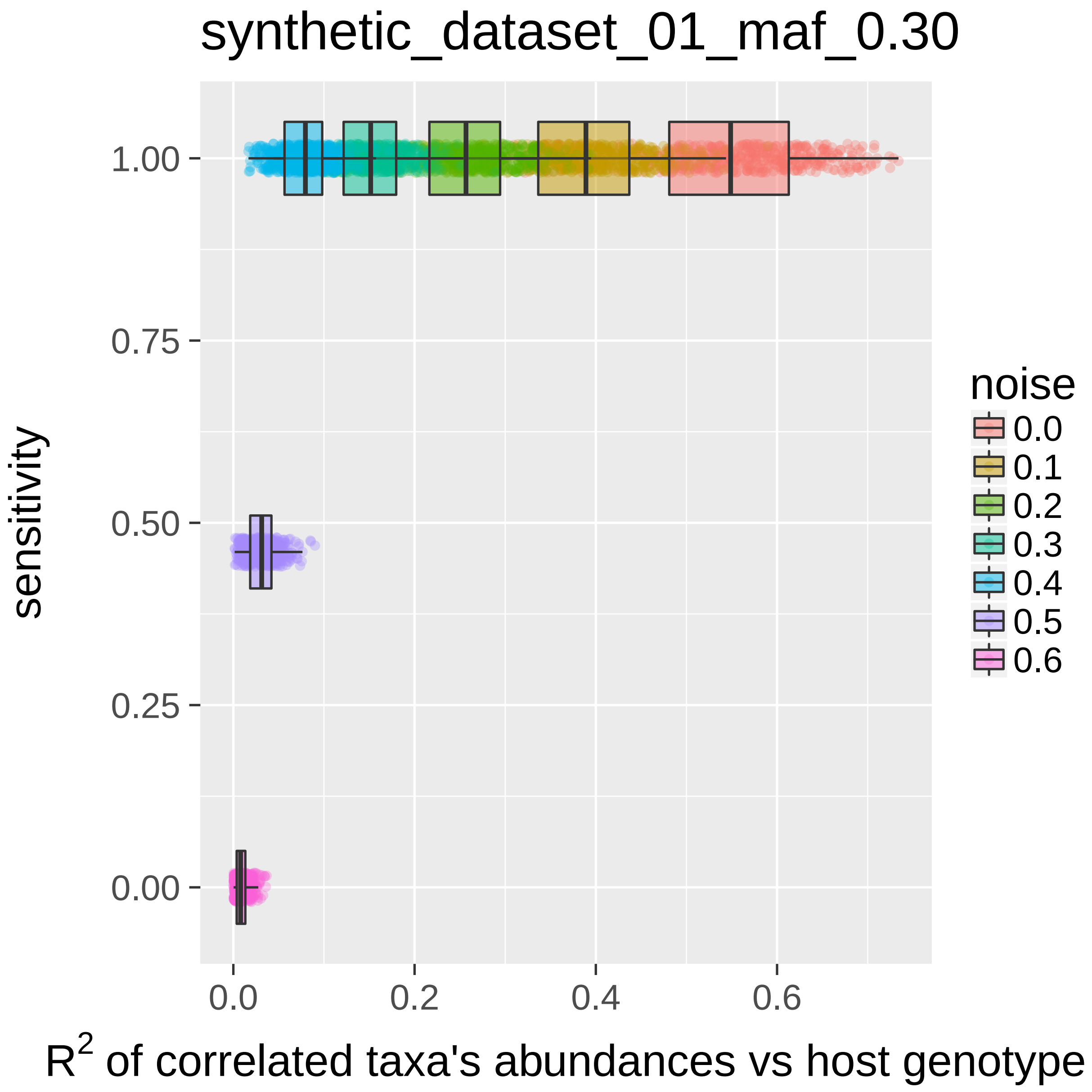

Figure S6: Sensitivity as a function of input R^2.

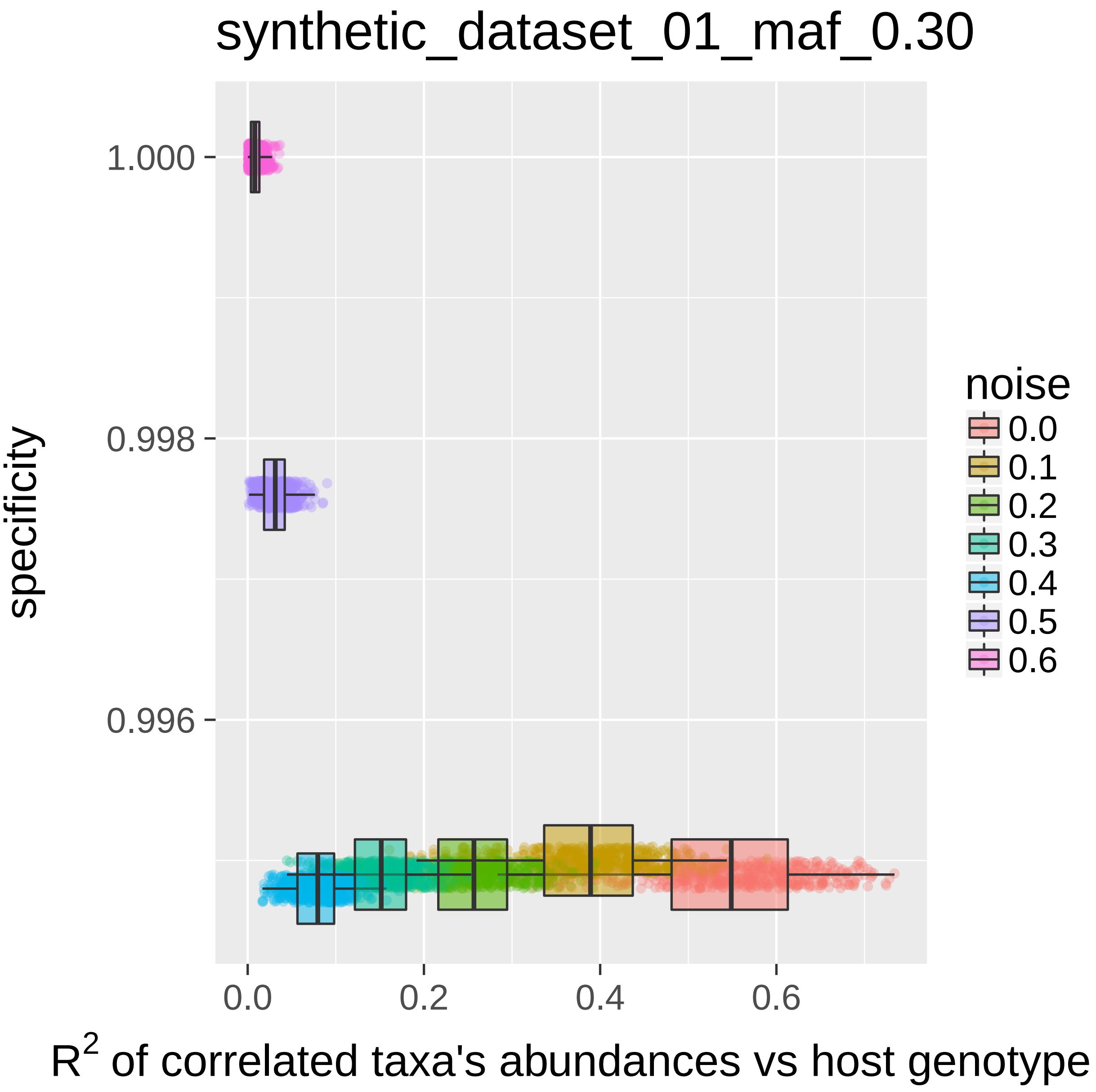

Figure S7: Specificity as a function of input R^2.

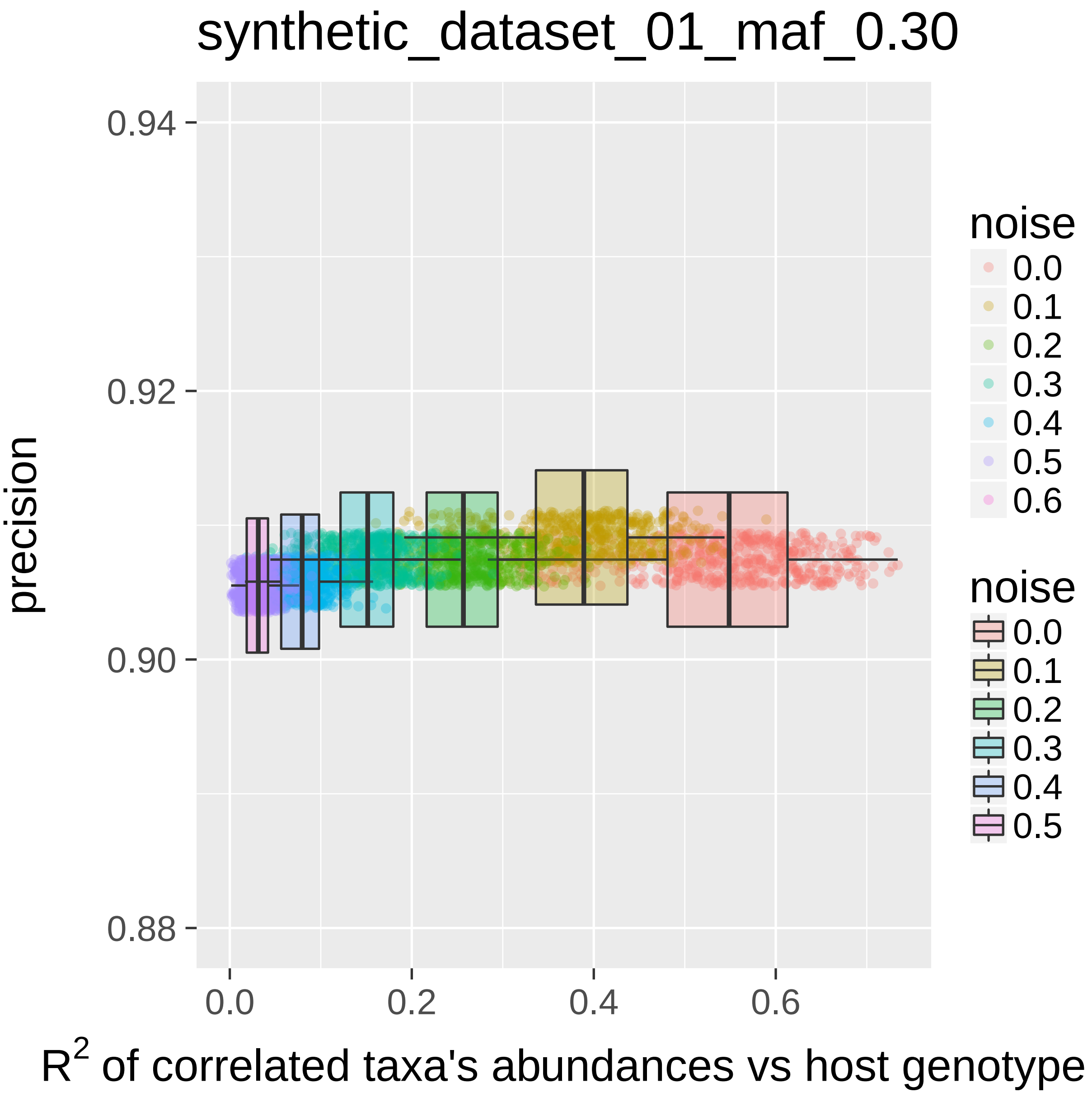

Figure S8: Precision as a function of input R^2.

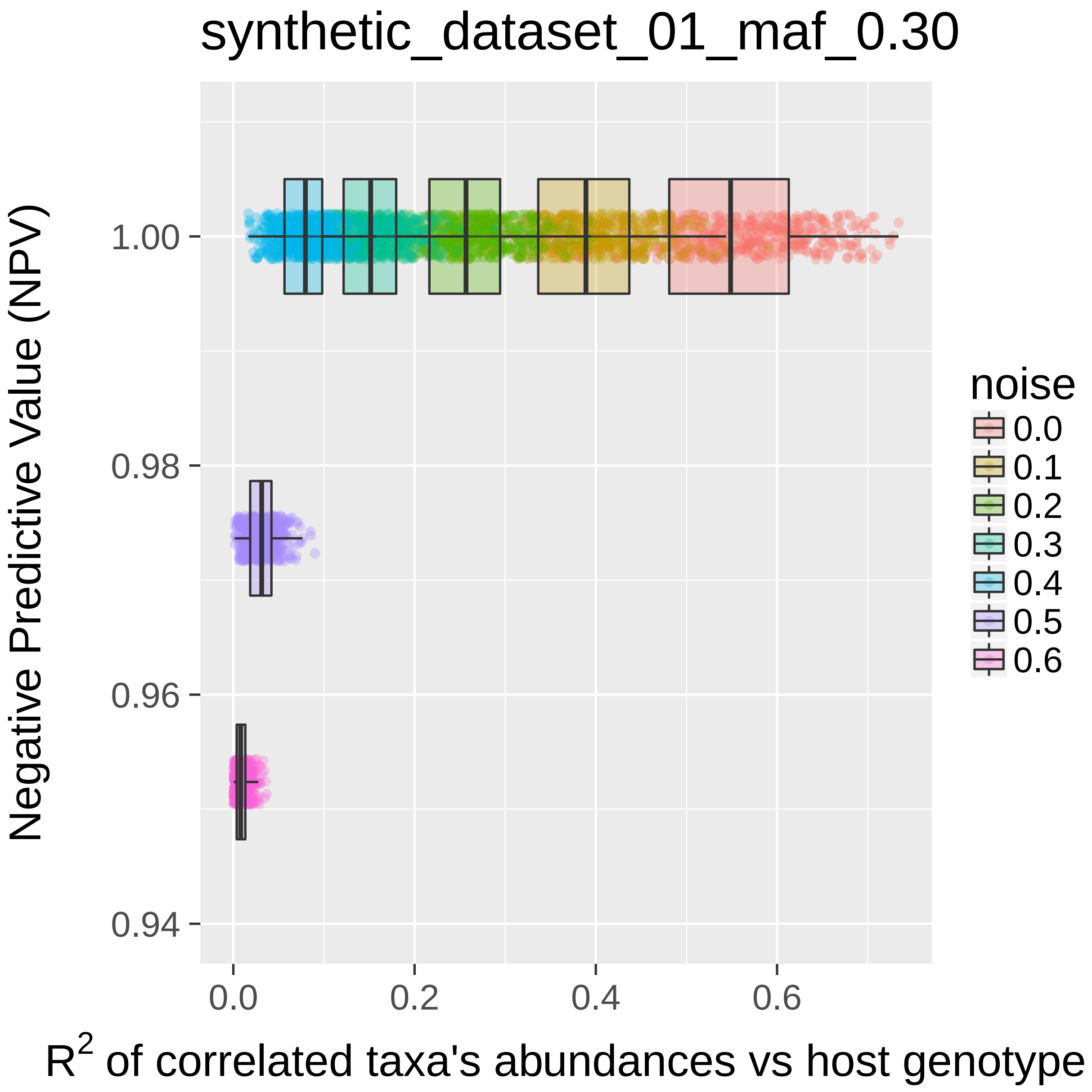

Figure S9: NPV as a function of input R^2.

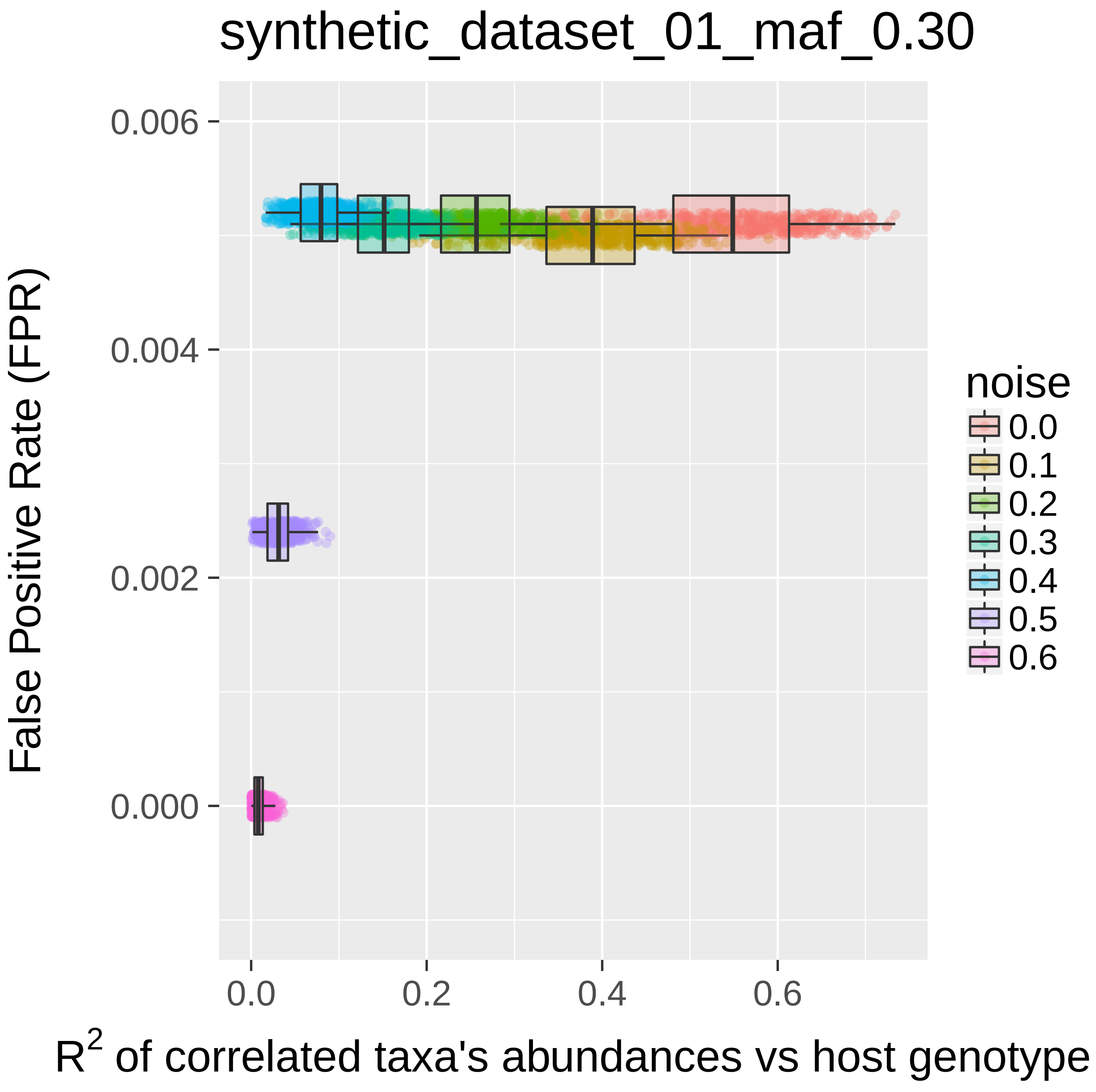

Figure S10: FPR as a function of input R^2.

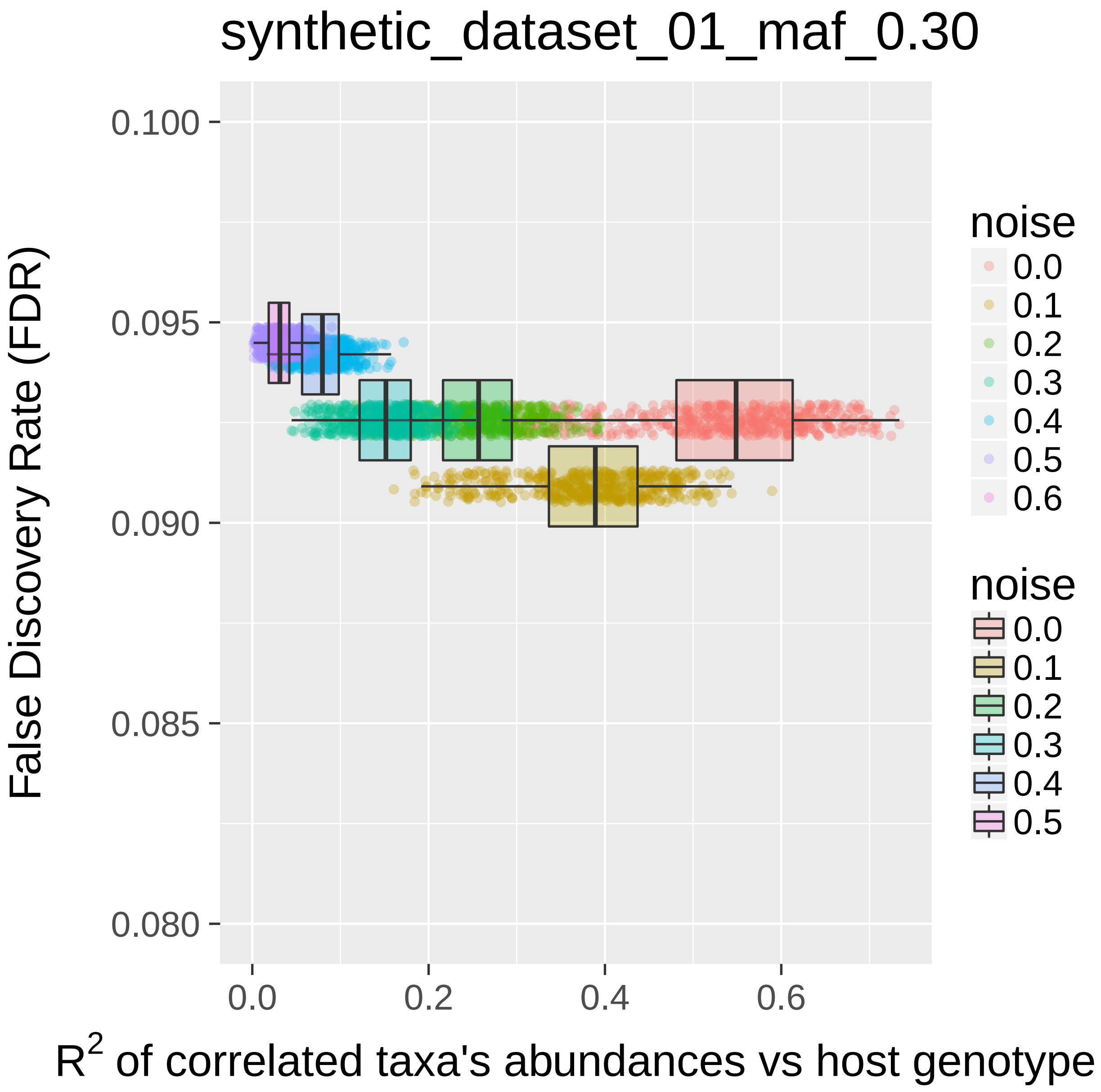

Figure S11: FDR as a function of input R^2.

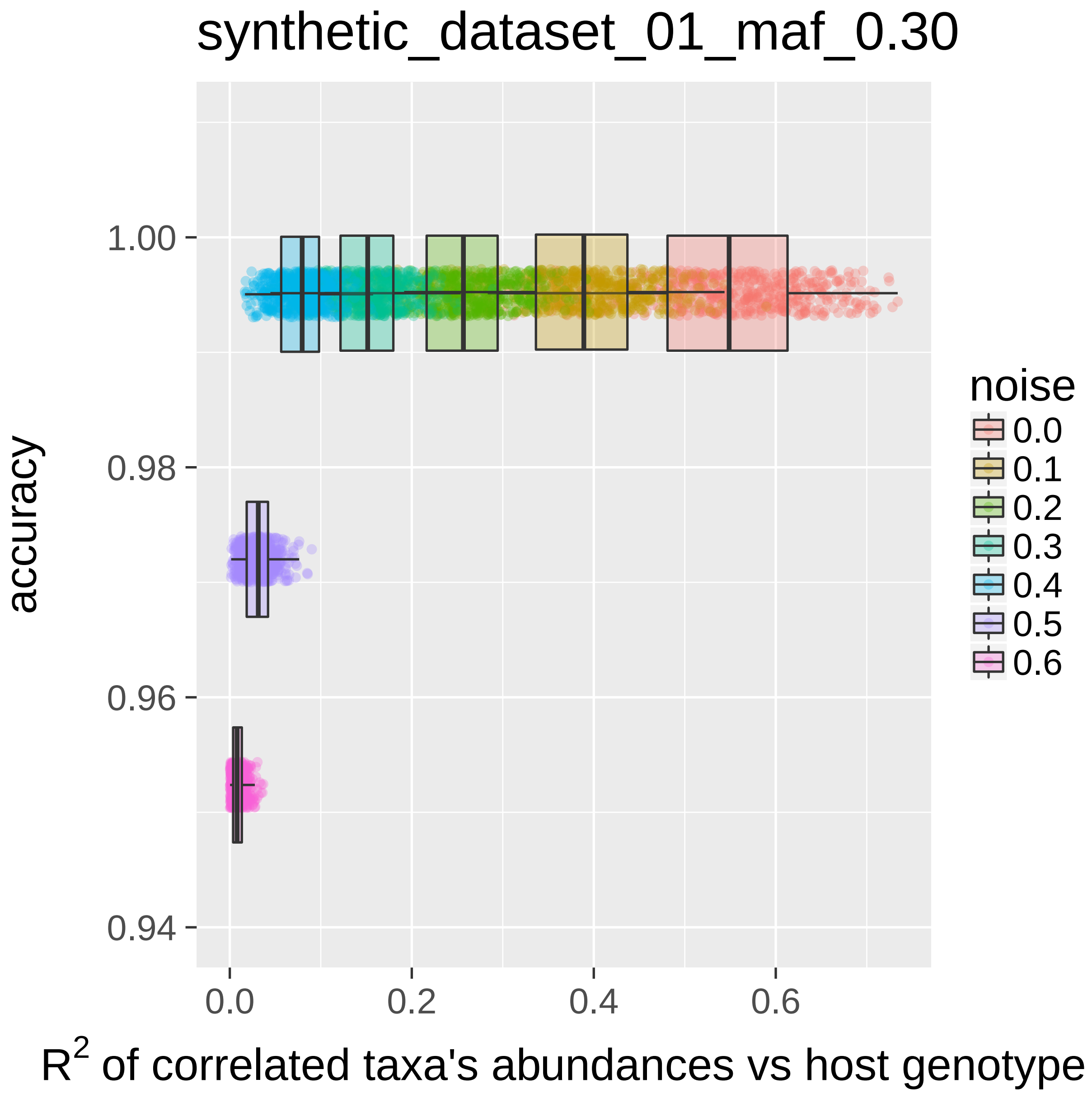

Figure S12: Accuracy as a function of input R^2.

2.7 Predicting Which Taxa are Associated with the Host Genetics

To evaluate how well HOMINID predicts the taxa that are correlated with the SNP genotypes, we examined two data sets with MAF = 0.30 and 500 total taxa. One data set has three taxa that correlate with the host genetics and 497 uncorrelated taxa. The second set has 20 taxa that correlate and 480 that don't.

We pulled out only the SNPs that were predicted to be correlated to the microbiome (i.e., each SNP's q-value $q \leq 0.1$) and omitted the SNPs predicted to be uncorrelated to the microbiome ($q > 0.1$).

Figure S13 shows the False Positive Rate ($FPR = FP/(FP + TN) = 1 -$ specificity) for feature selection, data for the set with three correlated taxa. Each point in the plot represents one SNP and the predictions of correlated (prediction positive) versus uncorrelated (prediction negative) for all 500 taxa, three of which correlate to the host genotype (condition positive) and 497 do not correlate (condition negative).

The FPR is zero—meaning no false positives, no uncorrelated taxa falsely predicted to be correlated—except at low input R^2, $R^2_{OLS} \lesssim 0.1$. Even at low input R^2, the FPR is low; HOMINID infrequently predicts an uncorrelated taxon to be correlated.

We see similar results when there are 20 correlated taxa (figure S14). With many more taxa correlated to the host SNP genotypes, HOMINID's FPR becomes non-zero at higher R^2_{OLS}, though the overall FPR is still low.

Figure S15 shows HOMINID's feature-selection sensitivity for the data set with three correlated taxa. Because there are only three correlated SNPs, sensitivity ($= TP/(TP + FN)$) has only three possible values: 1 (all three correlated SNPs correctly predicted to be correlated), 2/3 (two out of three correlated SNPs correctly predicted to be correlated, and one of the three is missed), and 1/3 (HOMINID selects only one correlated SNP and misses the other two). It's clear from this plot that HOMINID often misses correlated taxa.

Figure S16 shows the same thing, except for the data set with 20 correlated taxa. With more correlated taxa, sensitivity goes down.

How many correlated taxa HOMINID predicts does depend on effect size. The SNPs with poorer sensitivity are shifted toward lower R^2_{OLS}. This is particularly clear in the data set with 20 correlated taxa. But even at high R^2_{OLS}, HOMINID sometimes misses correlated taxa, especially if many taxa are correlated.

Taken together, HOMINID's prediction of correlated taxa is conservative. It often errs by missing correlated taxa, but it never or infrequently errs by predicting uncorrelated taxa to be correlated.

Figures S17, S18, S19, S20, S21, S22, S23, S24, S25, S26, S27, and S28 show other metrics of the performance of HOMINID's stability selection step, selecting the taxa associated with the host genetics. All plots except for NPV display points with vertical jitter.

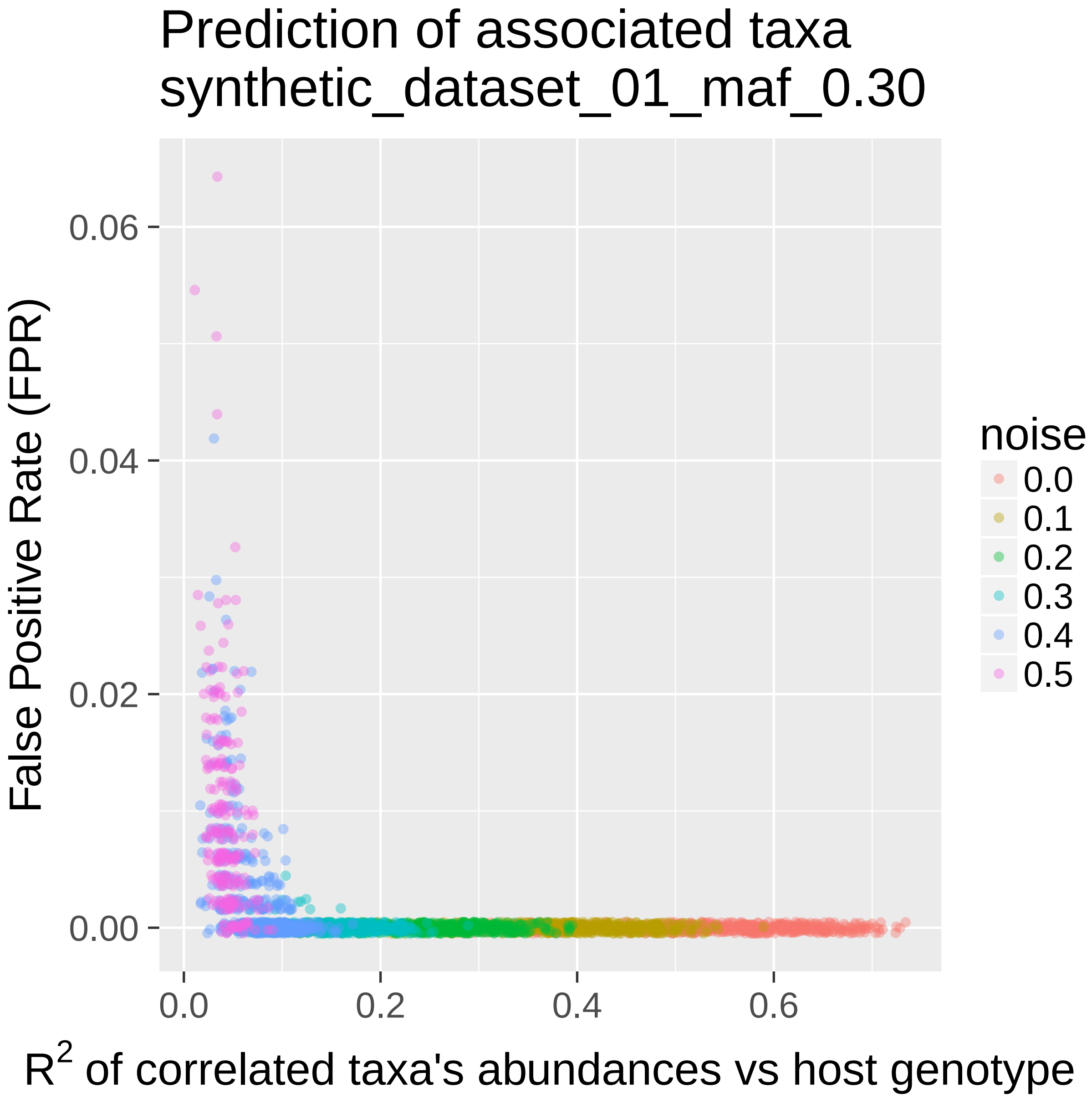

Figure S13: False Positive Rate (FPR) for feature selection, selecting the taxa that correlate to the host genetics, as a function of input R^2, for the data set with three correlated taxa. FPR is zero, i.e., no uncorrelated taxa were falsely predicted to be correlated, except at low input R^2. (There is vertical variation due to graphing "jitter", to better display the denseness of points at $FPR = 0$.)

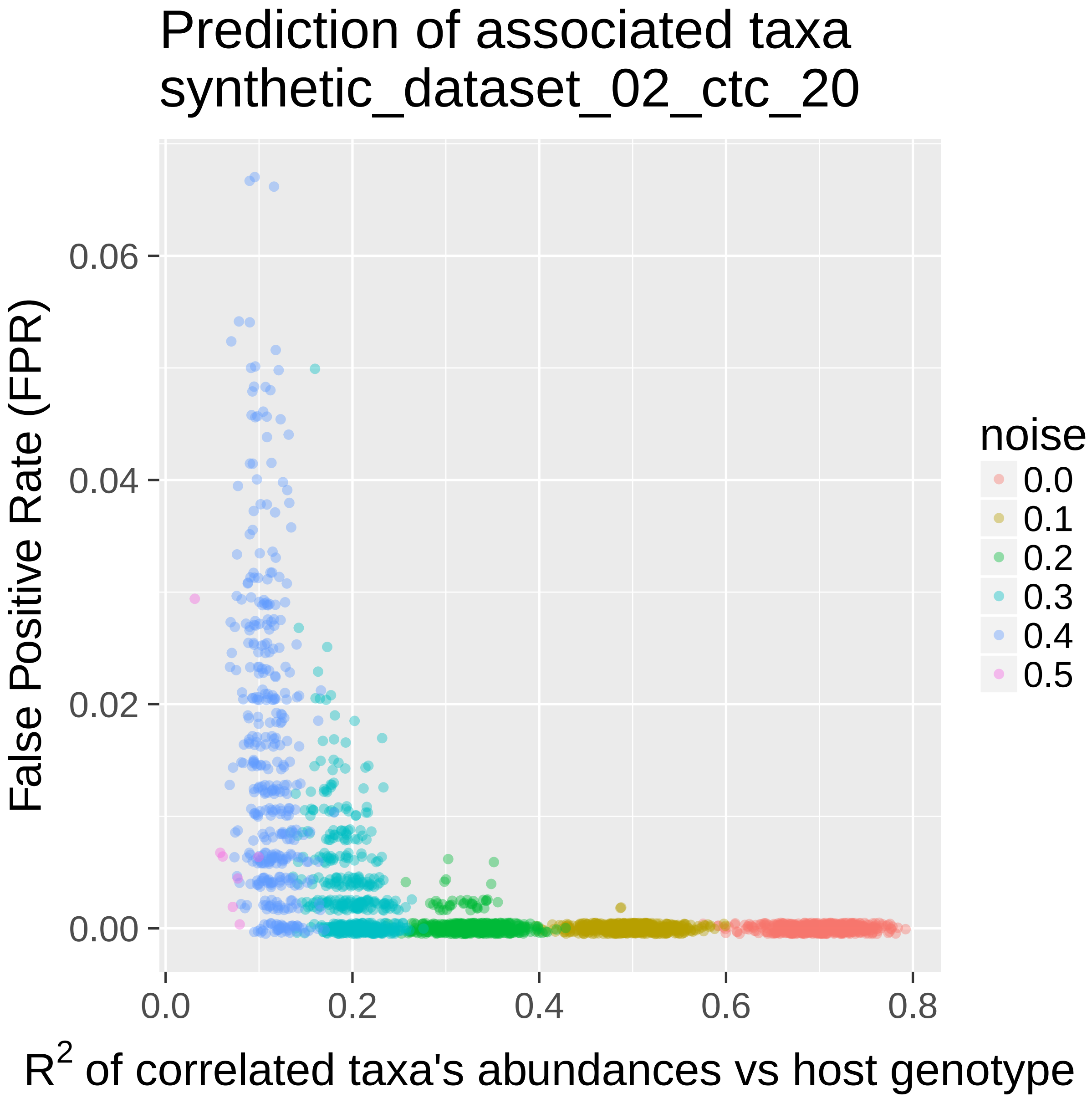

Figure S14: Same as figure S13 except this data set has 20 correlated taxa.

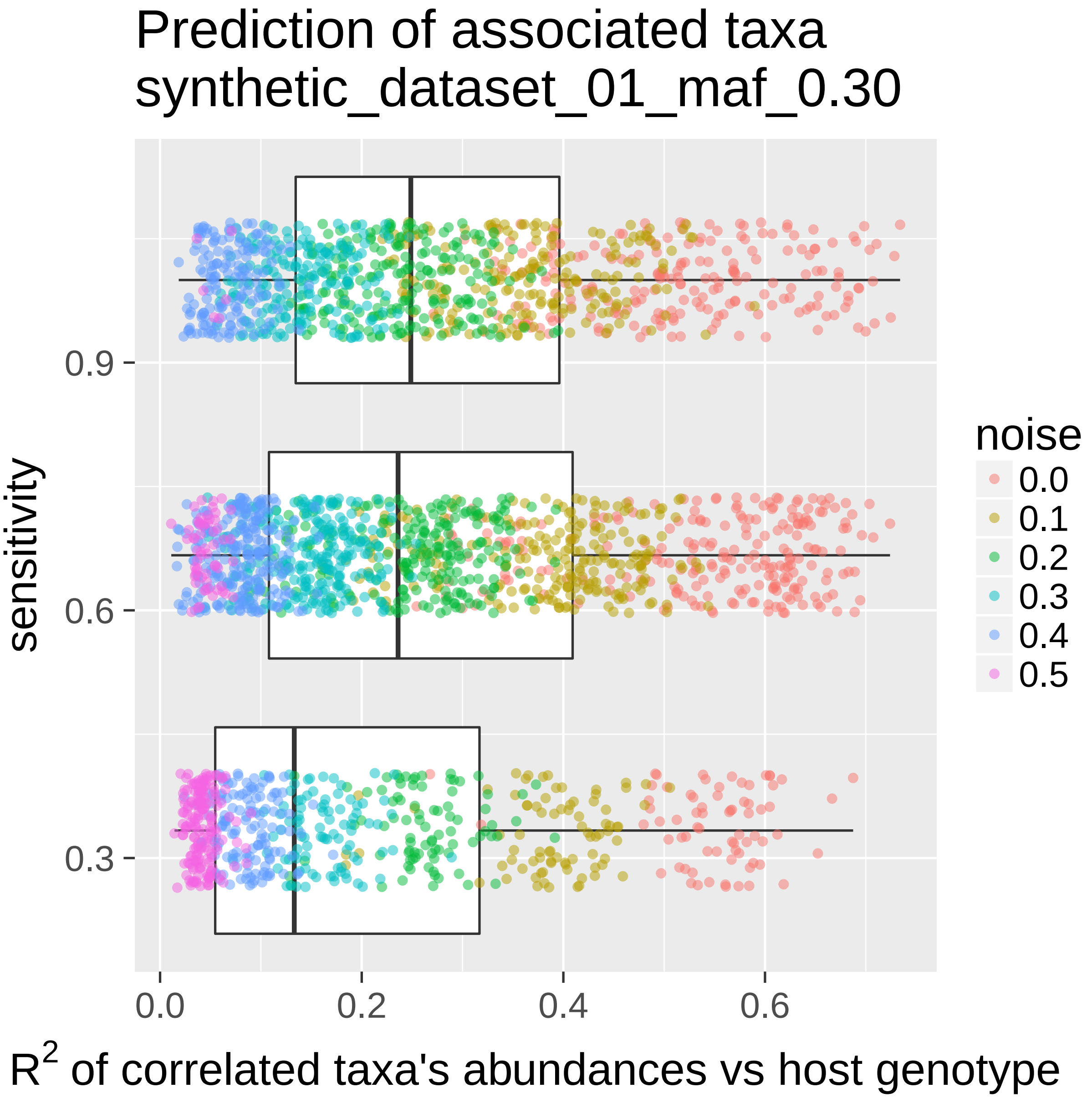

Figure S15: Sensitivity for feature selection, selecting the taxa that correlate to the host genetics, as a function of input R^2, for the data set with three correlated taxa.

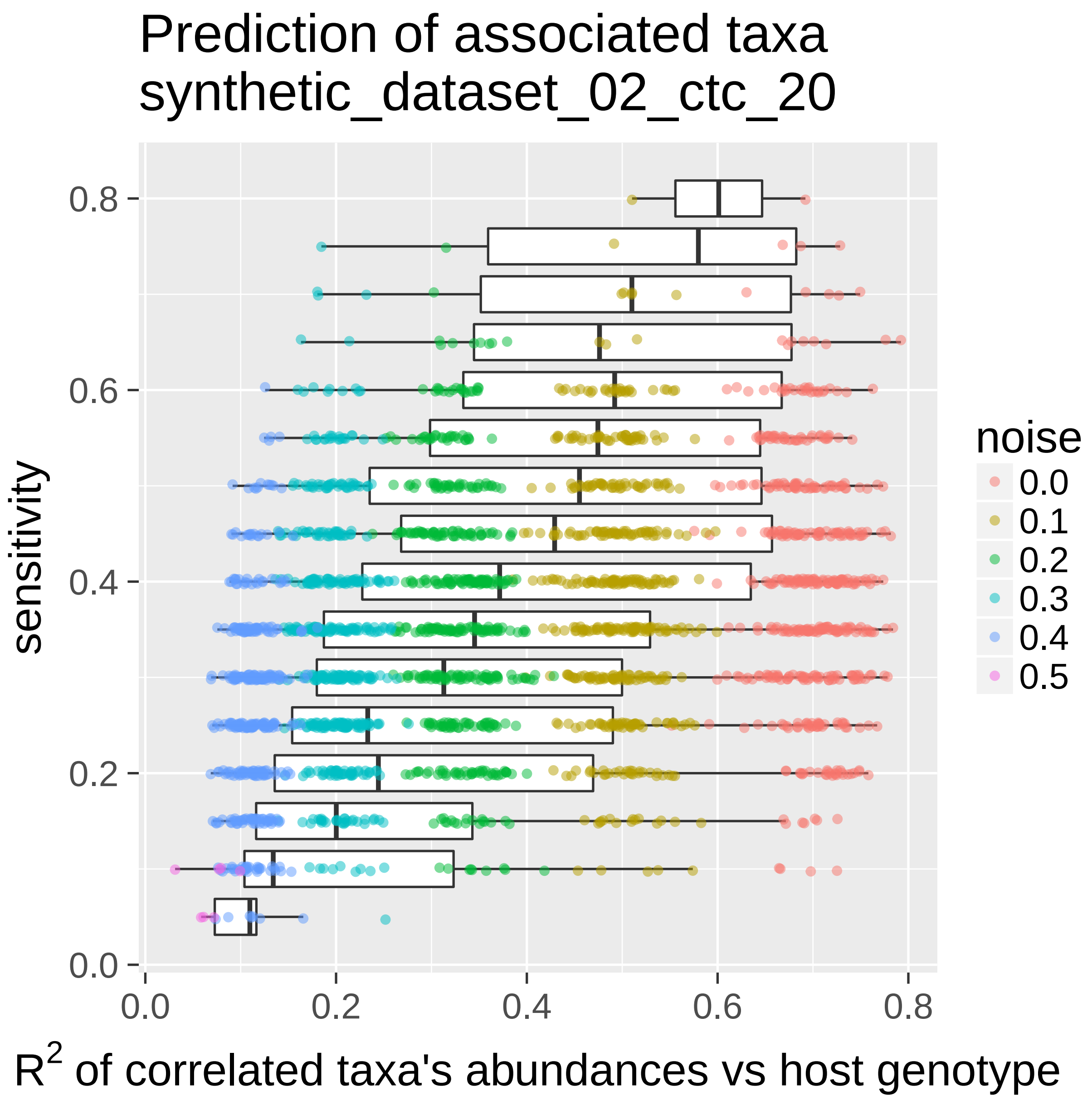

Figure S16: Same as figure S15, except for the data set with 20 correlated taxa.

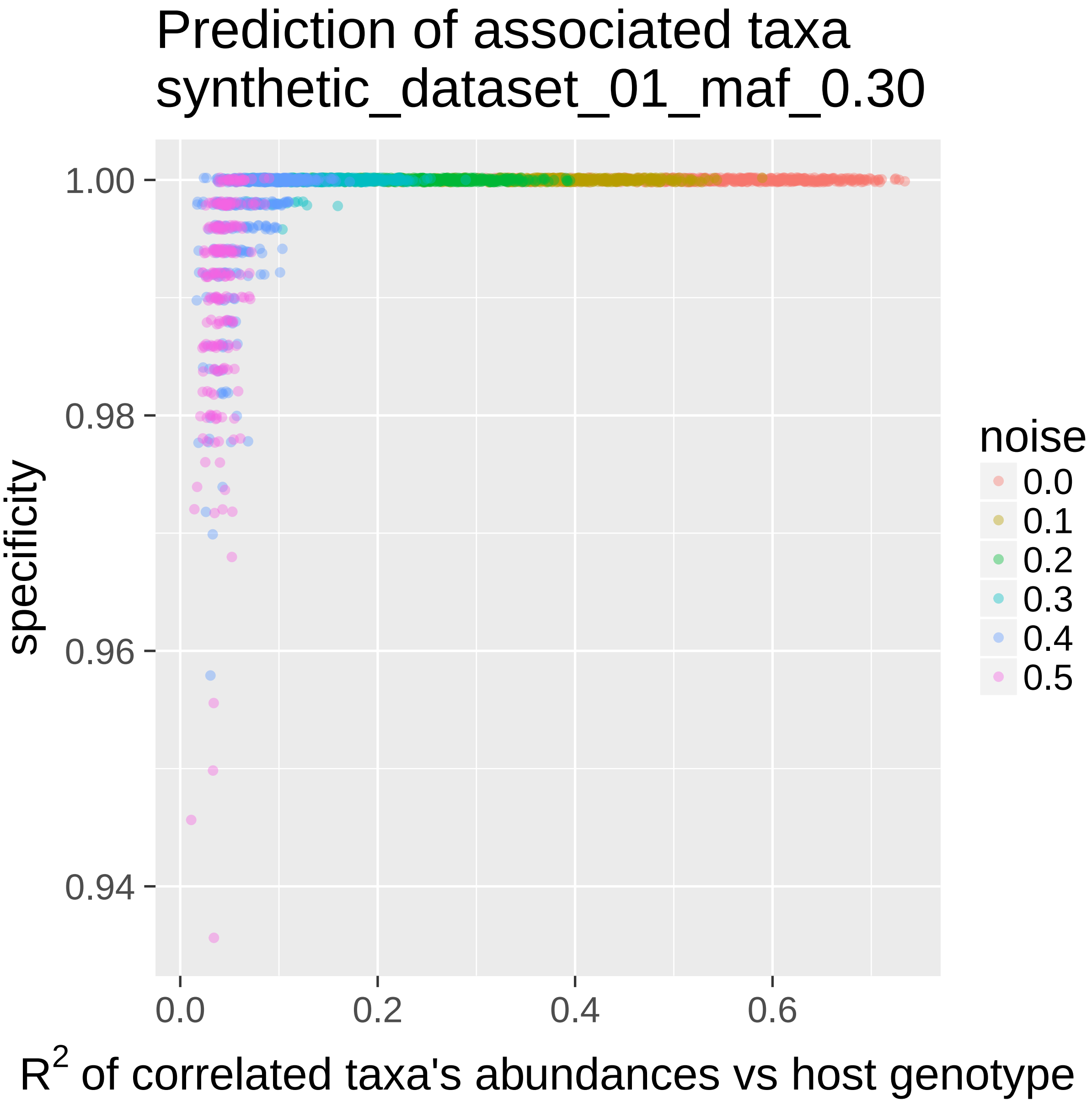

Figure S17: Specificity for feature selection, selecting the taxa that correlate to the host genetics, as a function of input R^2, for the data set with three correlated taxa.

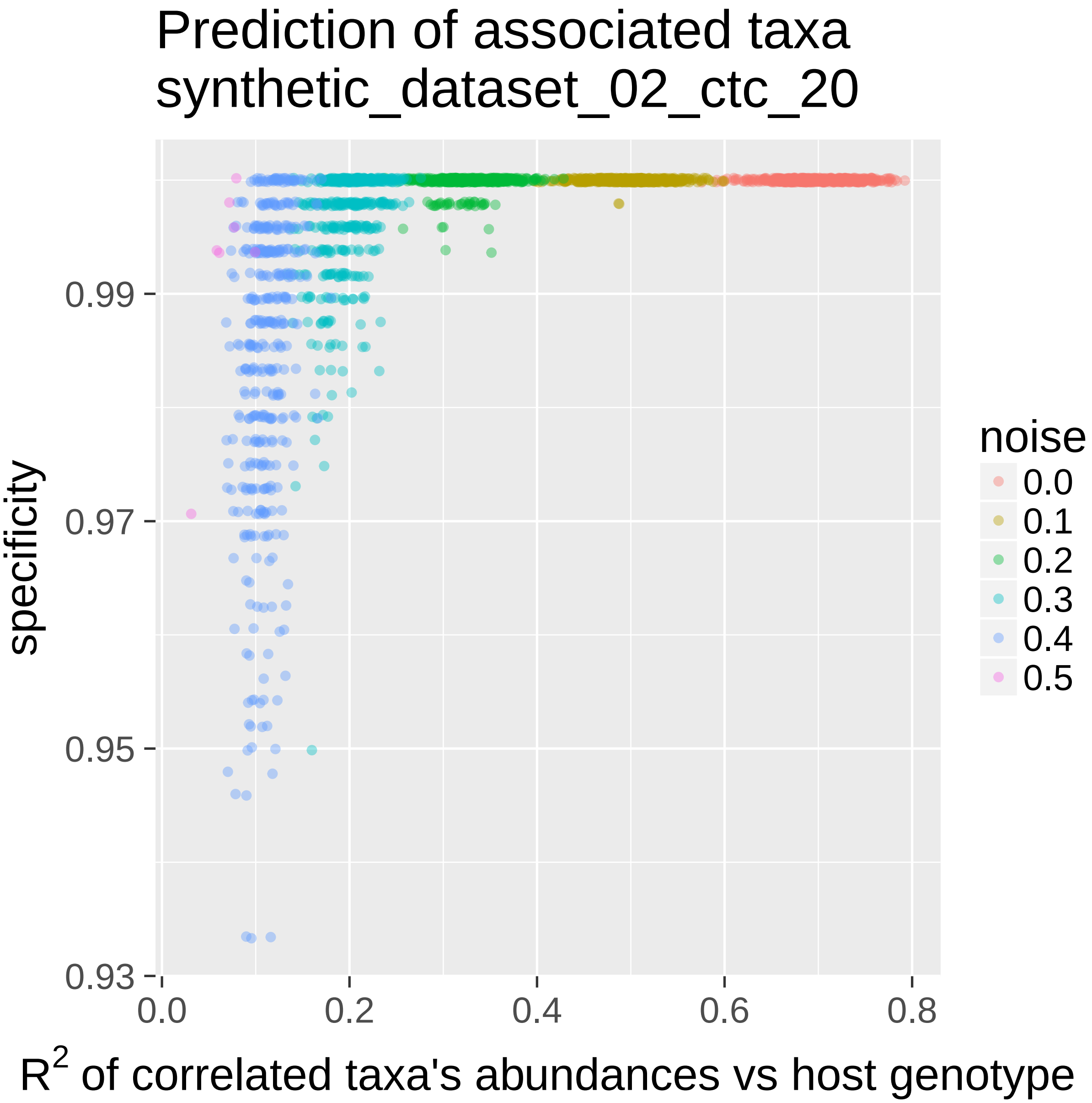

Figure S18: Same as figure S17, except for the data set with 20 correlated taxa.

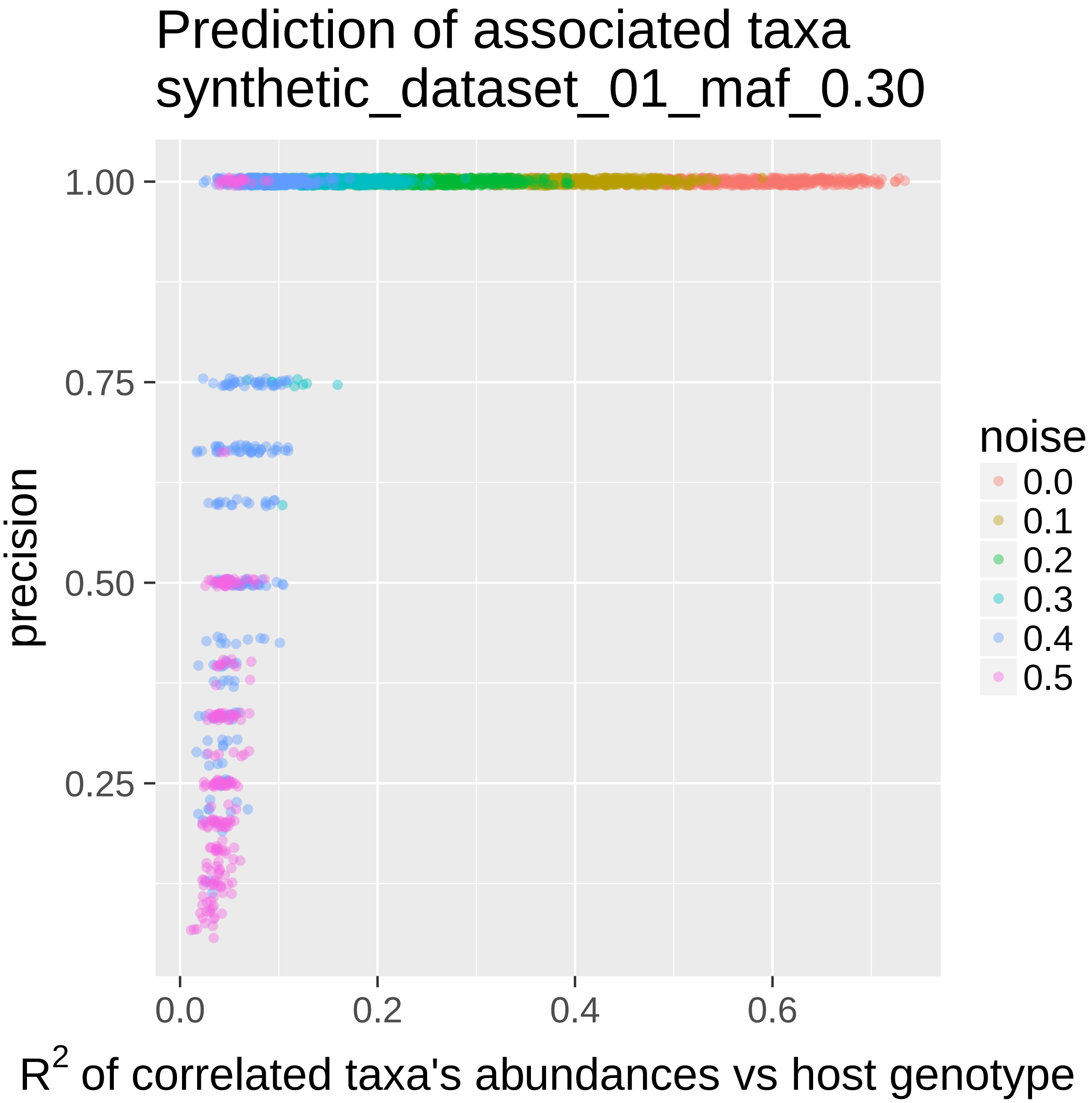

Figure S19: Precision for feature selection, selecting the taxa that correlate to the host genetics, as a function of input R^2, for the data set with three correlated taxa.

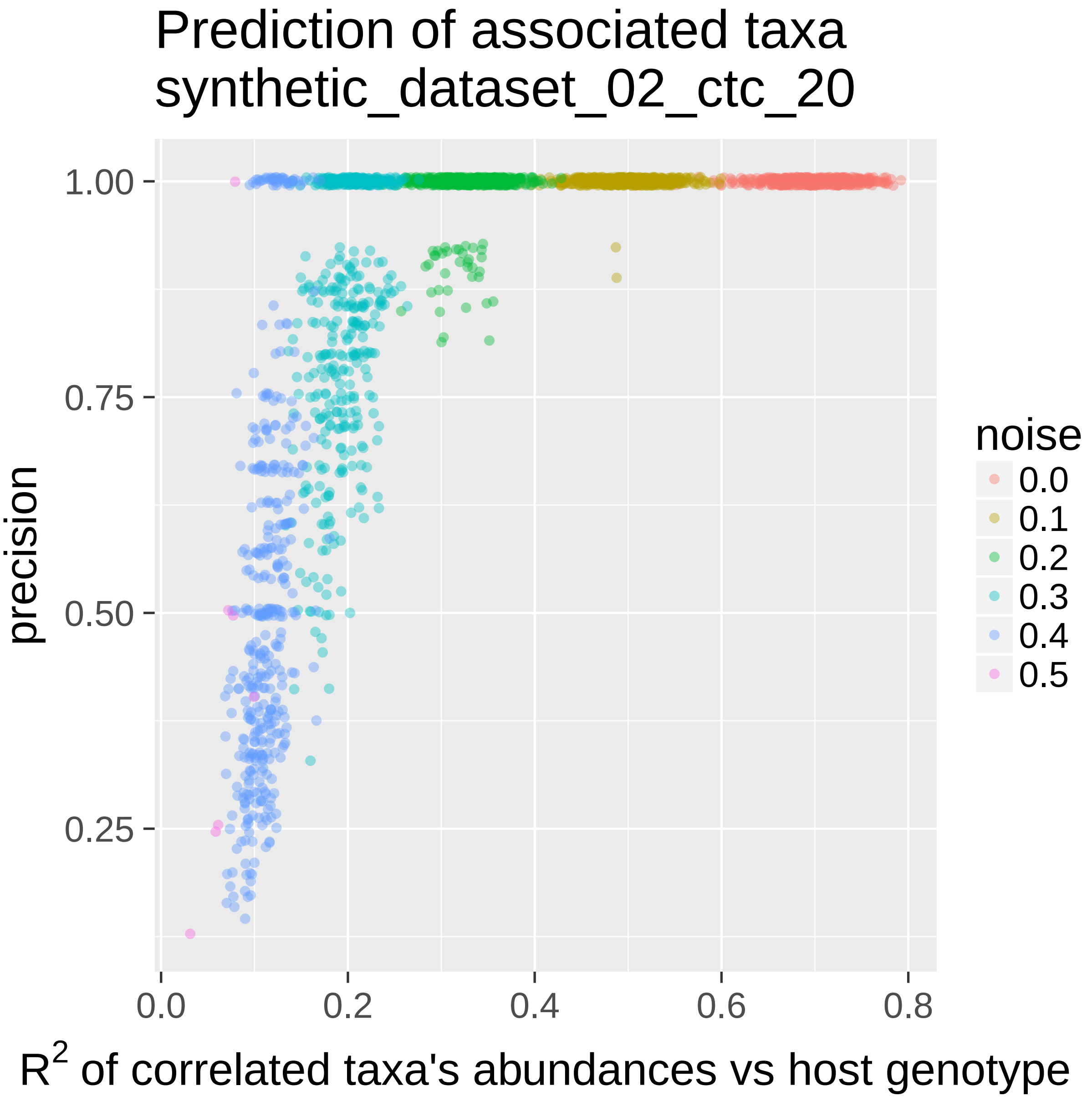

Figure S20: Same as figure S19, except for the data set with 20 correlated taxa.

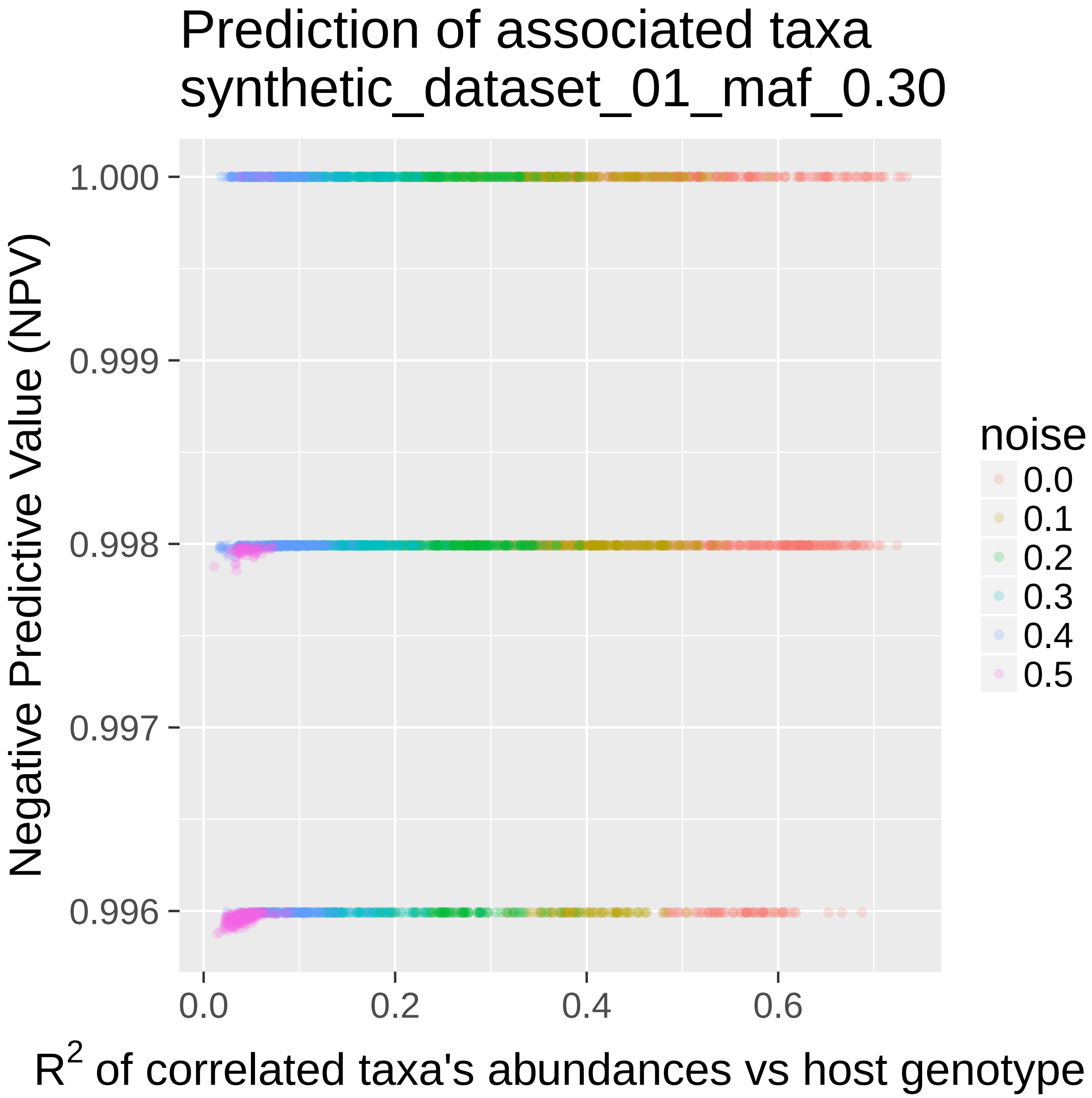

Figure S21: NPV for feature selection, selecting the taxa that correlate to the host genetics, as a function of input R^2, for the data set with three correlated taxa.

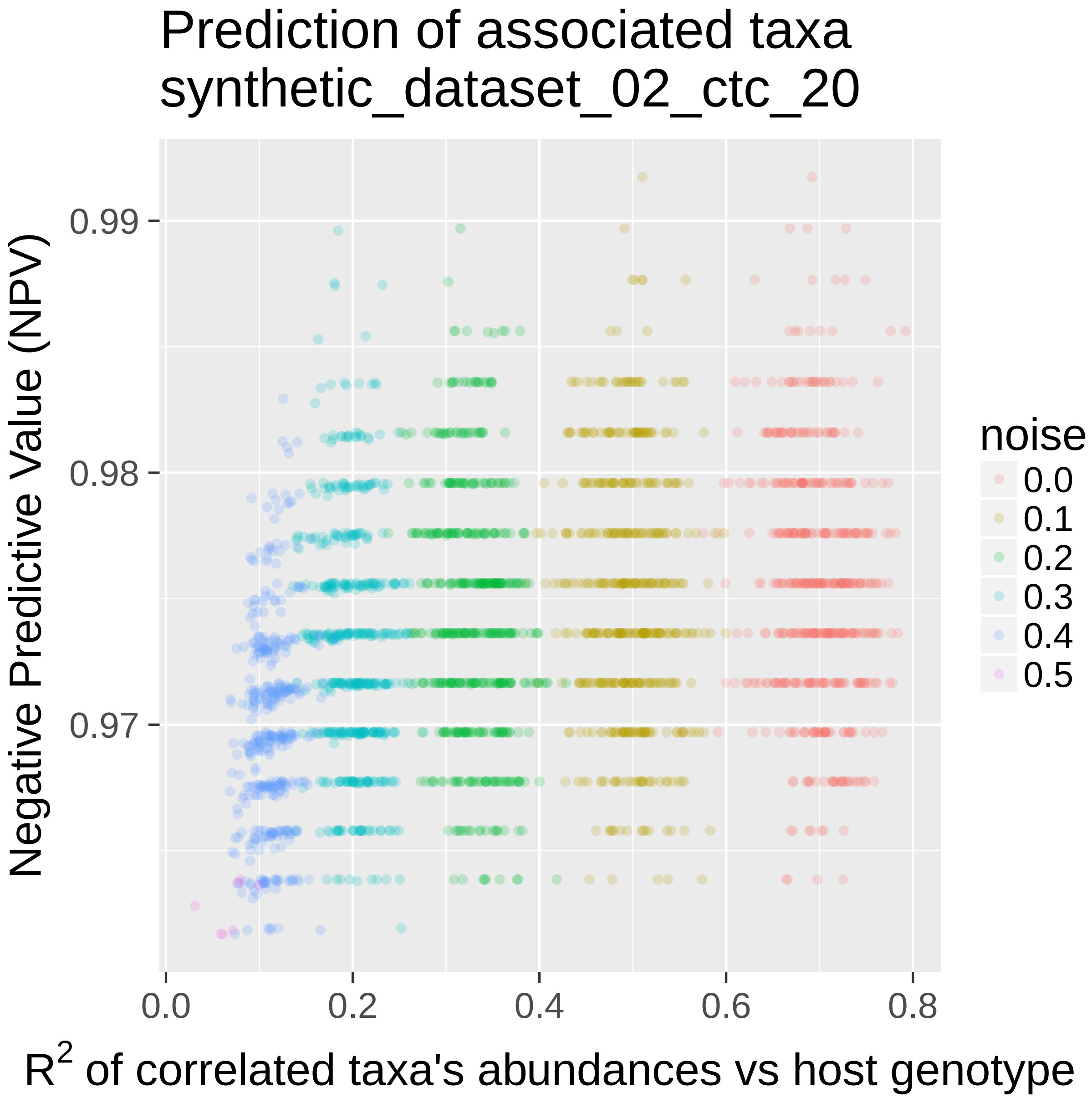

Figure S22: Same as figure S21, except for the data set with 20 correlated taxa.

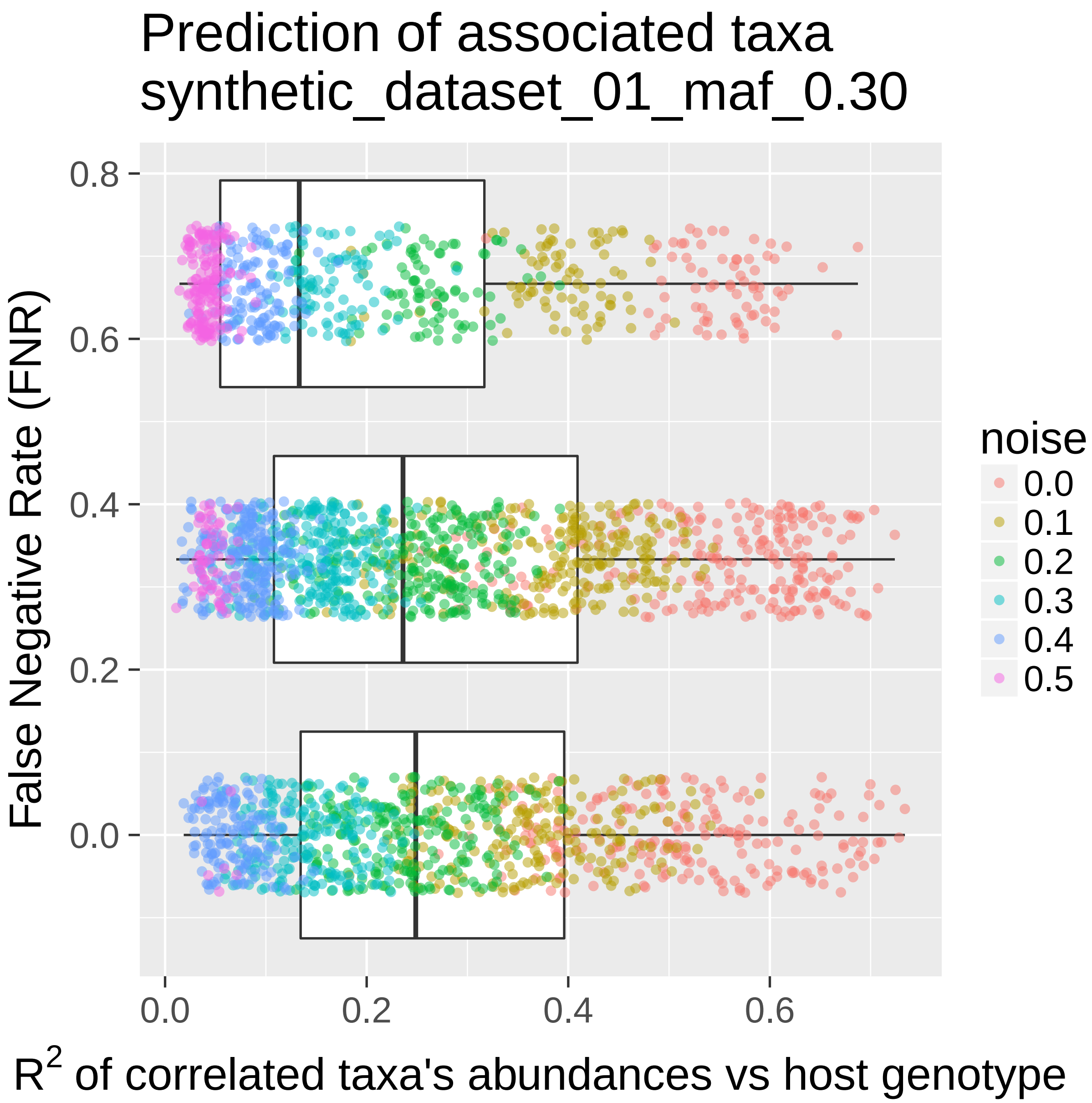

Figure S23: FNR for feature selection, selecting the taxa that correlate to the host genetics, as a function of input R^2, for the data set with three correlated taxa.

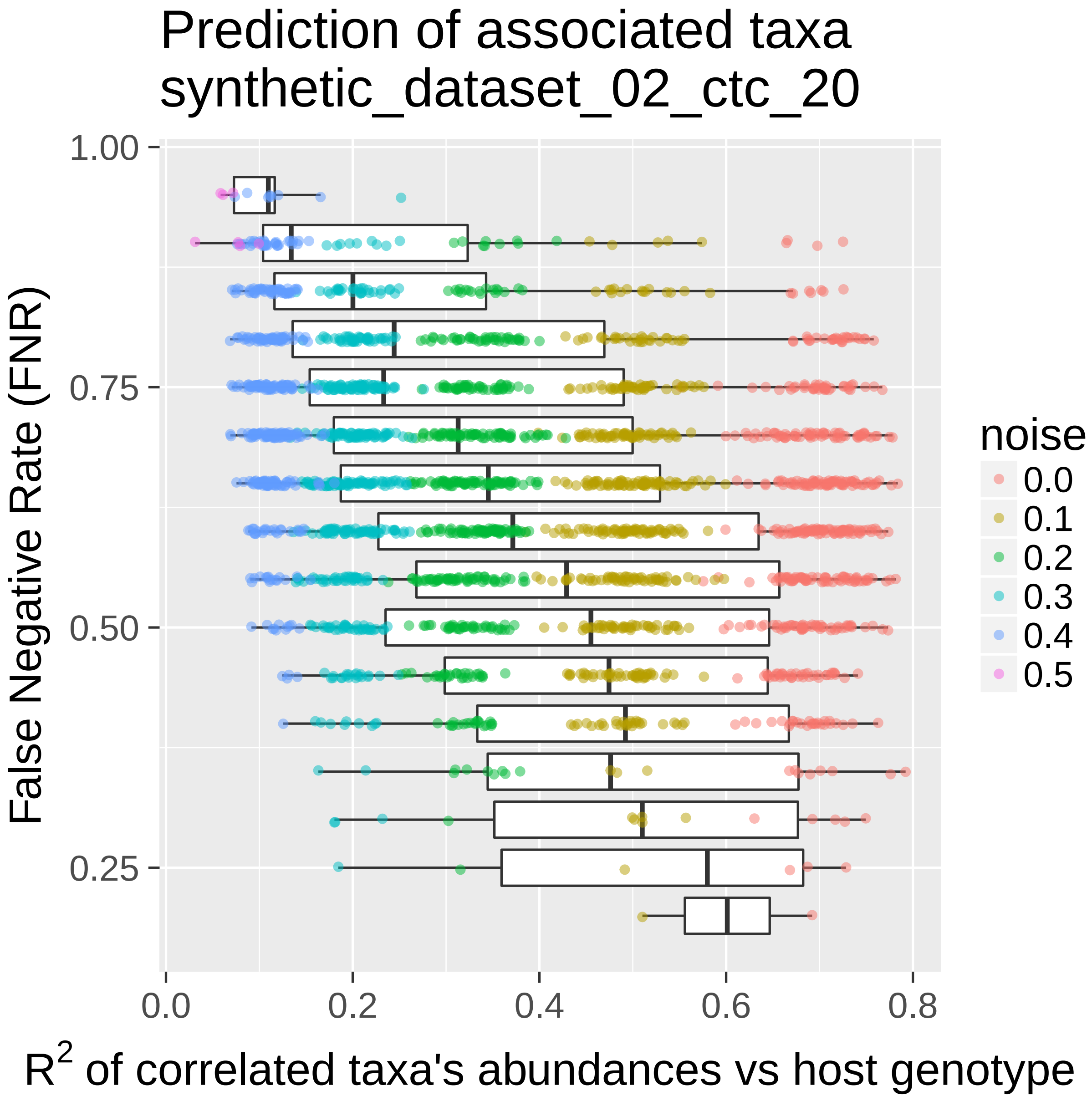

Figure S24: Same as figure S23, except for the data set with 20 correlated taxa.

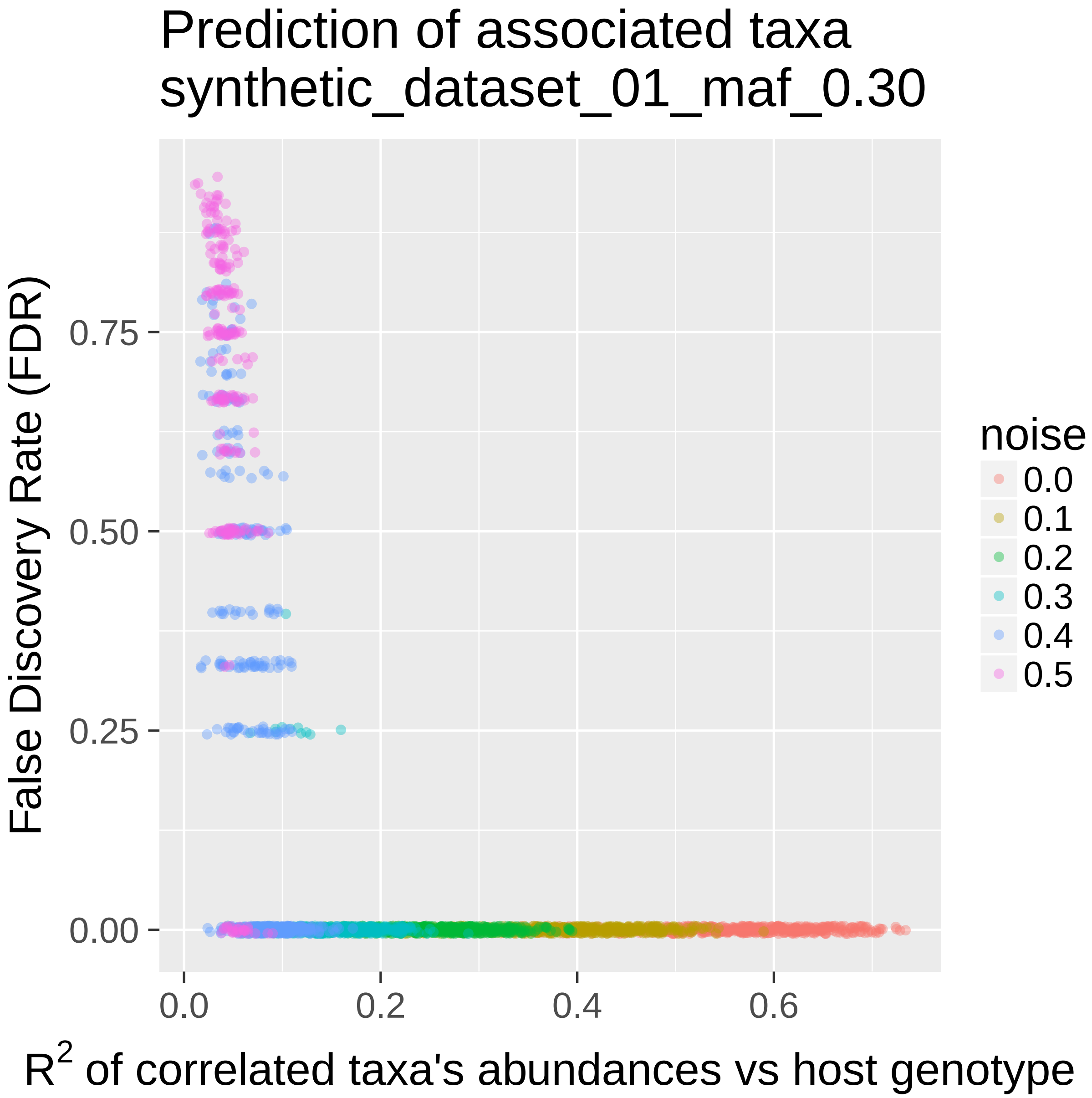

Figure S25: FDR for feature selection, selecting the taxa that correlate to the host genetics, as a function of input R^2, for the data set with three correlated taxa.

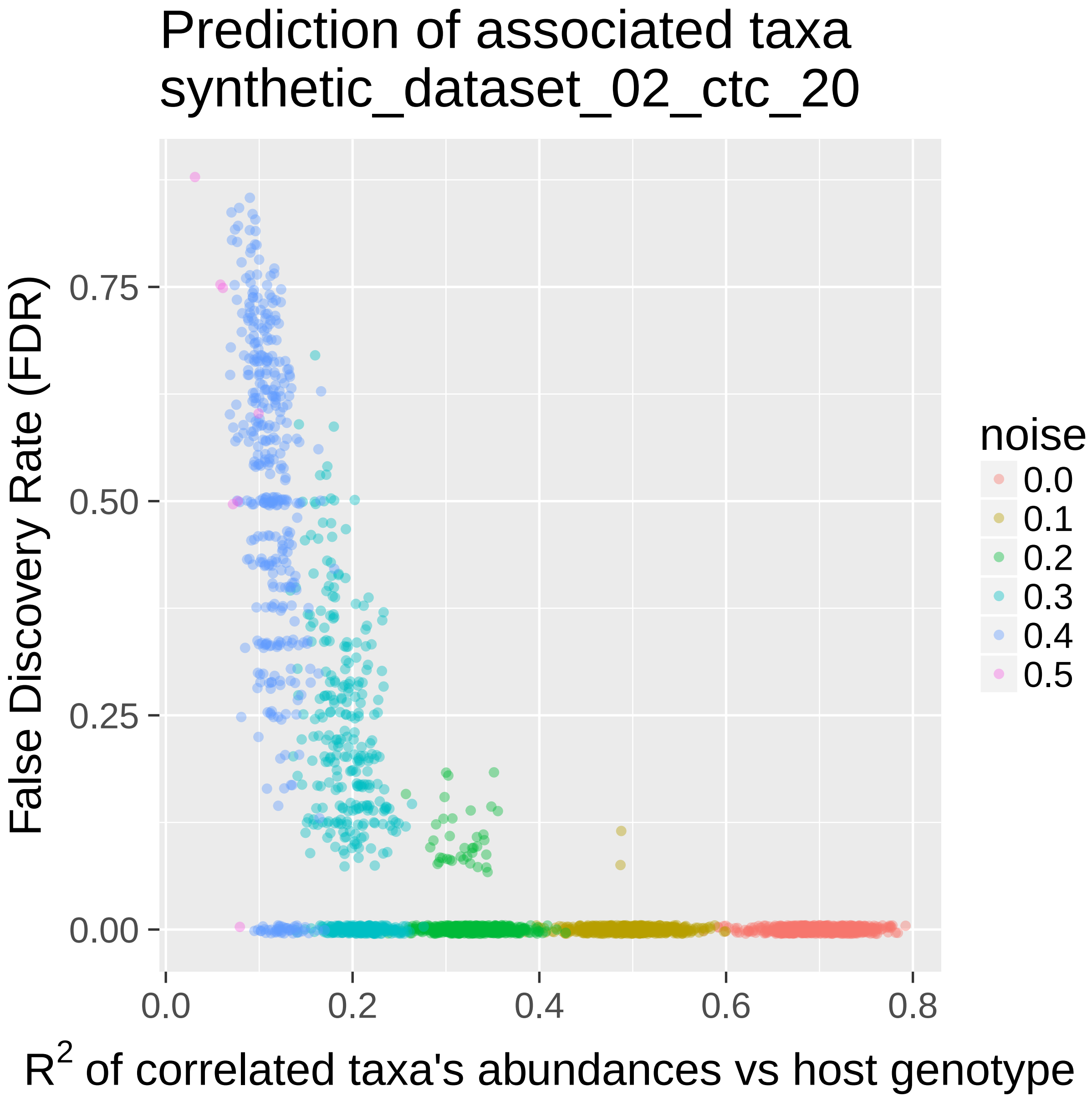

Figure S26: Same as figure S25, except for the data set with 20 correlated taxa.

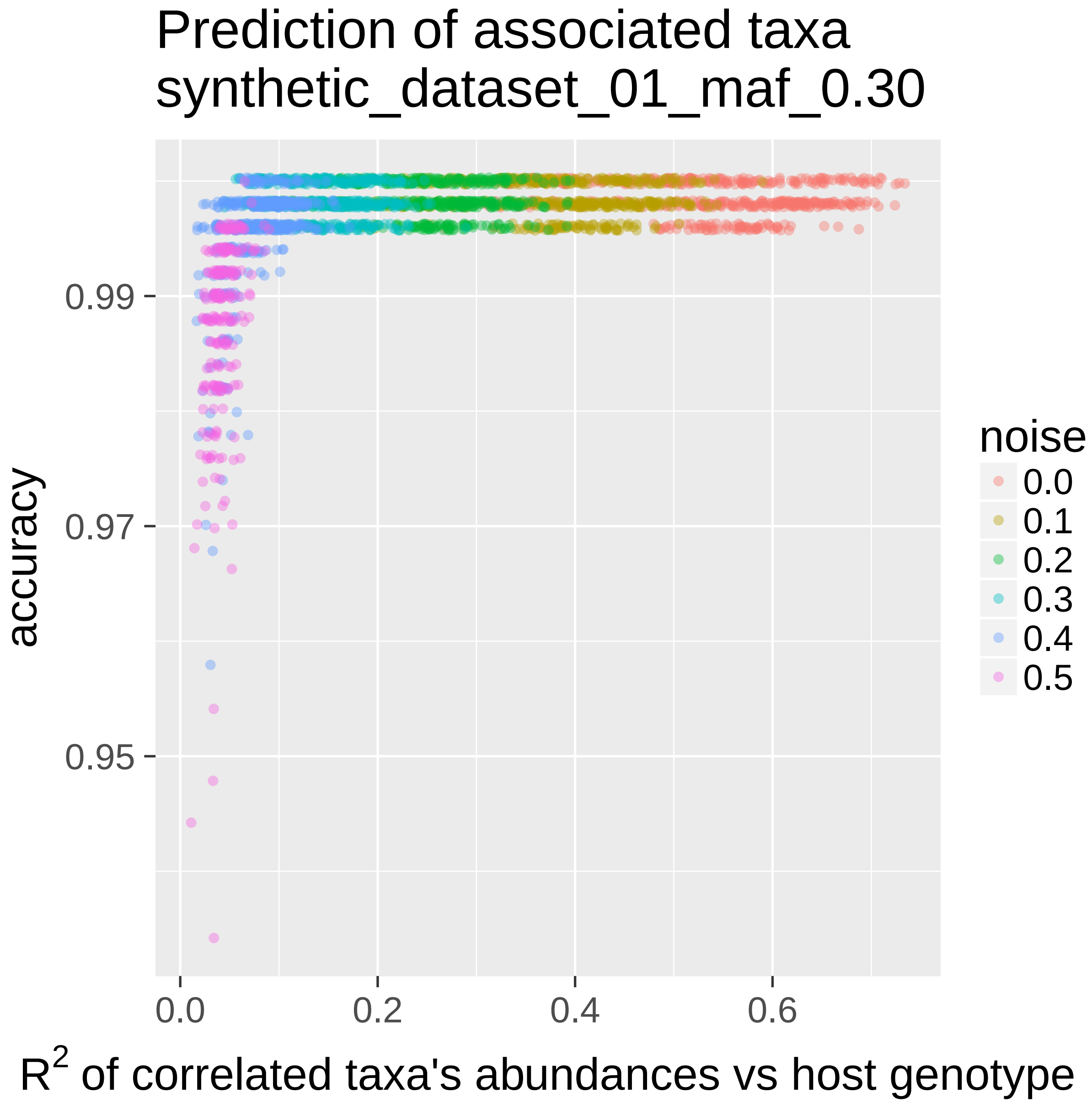

Figure S27: Accuracy for feature selection, selecting the taxa that correlate to the host genetics, as a function of input R^2, for the data set with three correlated taxa.

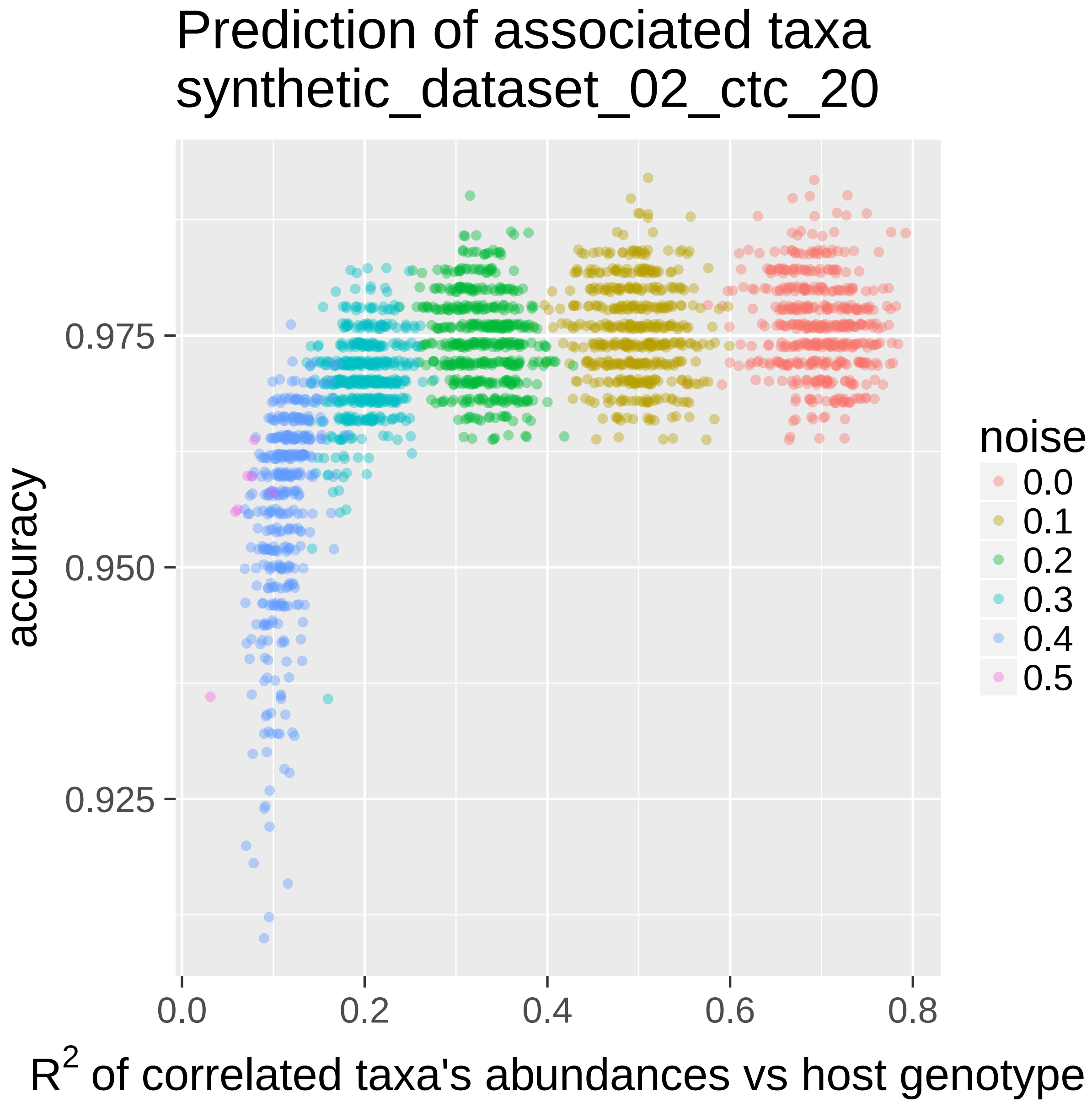

Figure S28: Same as figure S27, except for the data set with 20 correlated taxa.

2.8 HOMINID's Performance as a Function of Minor Allele Frequency (MAF)

To see what effect host SNP MAF has on HOMINID's performance, we analyzed data sets with MAF = 0.10, 0.15, 0.20, 0.25, 0.30, 0.35, 0.40, 0.45, and 0.50 (see section 2.2 for more details on these data sets). Figure S29 plots together FNR from data sets of all noise levels and all MAF values. The different MAF values are shown as different colored boxplots; different noise levels but same MAF are plotted in the same color.

FNR values from datasets with different MAF all follow the same trend, indicating that HOMINID's results are insensitive to MAF.

In figures S30, S31, S32, S33, S34, S35, and S36 are plotted other metrics of HOMINID's performance, again with different MAF values shown in different colors.

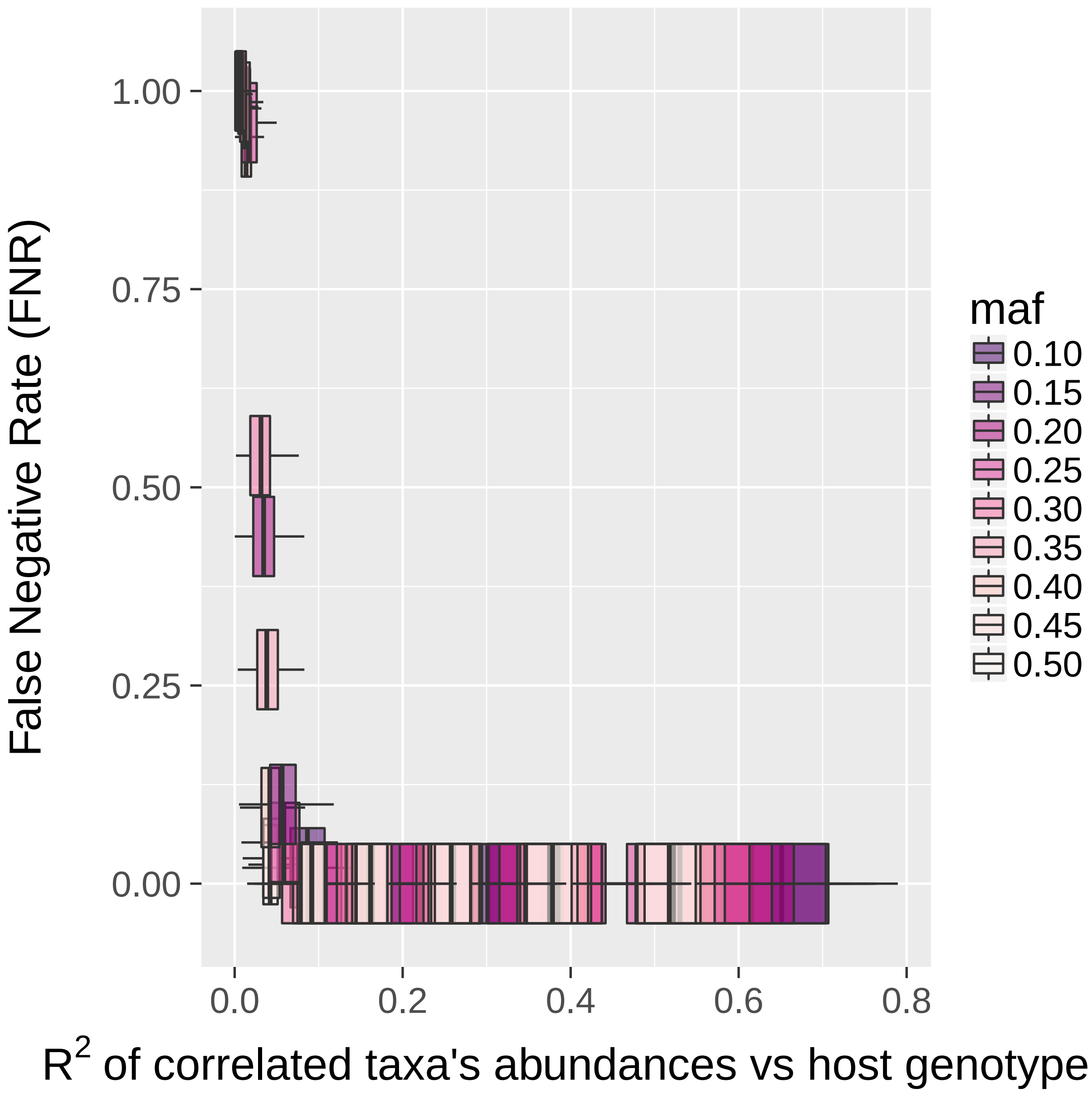

Figure S29: HOMINID performs equally well on data sets with different MAF: FNR as a function of input R^2, combining into the same plot data sets with MAF = 0.10, 0.15, 0.20, 0.25, 0.30, 0.35, 0.40, 0.45, and 0.50. Data sets with different MAF values are plotted in different colors. Data sets with the same MAF but different noise levels are plotted in the same color.

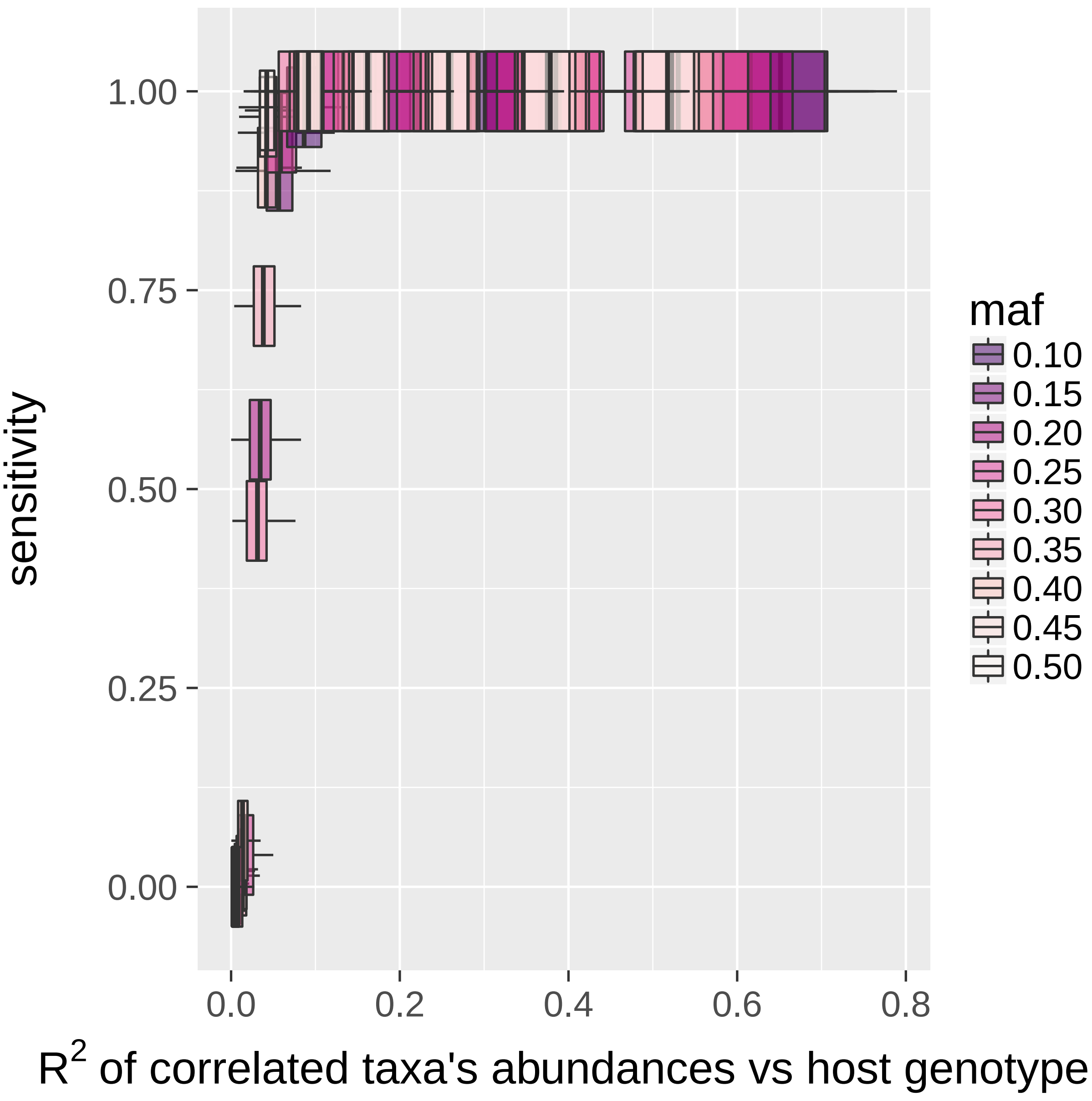

Figure S30: HOMINID performs equally well on data sets with different MAF: sensitivity as a function of input R^2, combining into the same plot data sets with MAF = 0.10, 0.15, 0.20, 0.25, 0.30, 0.35, 0.40, 0.45, and 0.50. Data sets with different MAF values are plotted in different colors. Data sets with the same MAF but different noise levels are plotted in the same color.

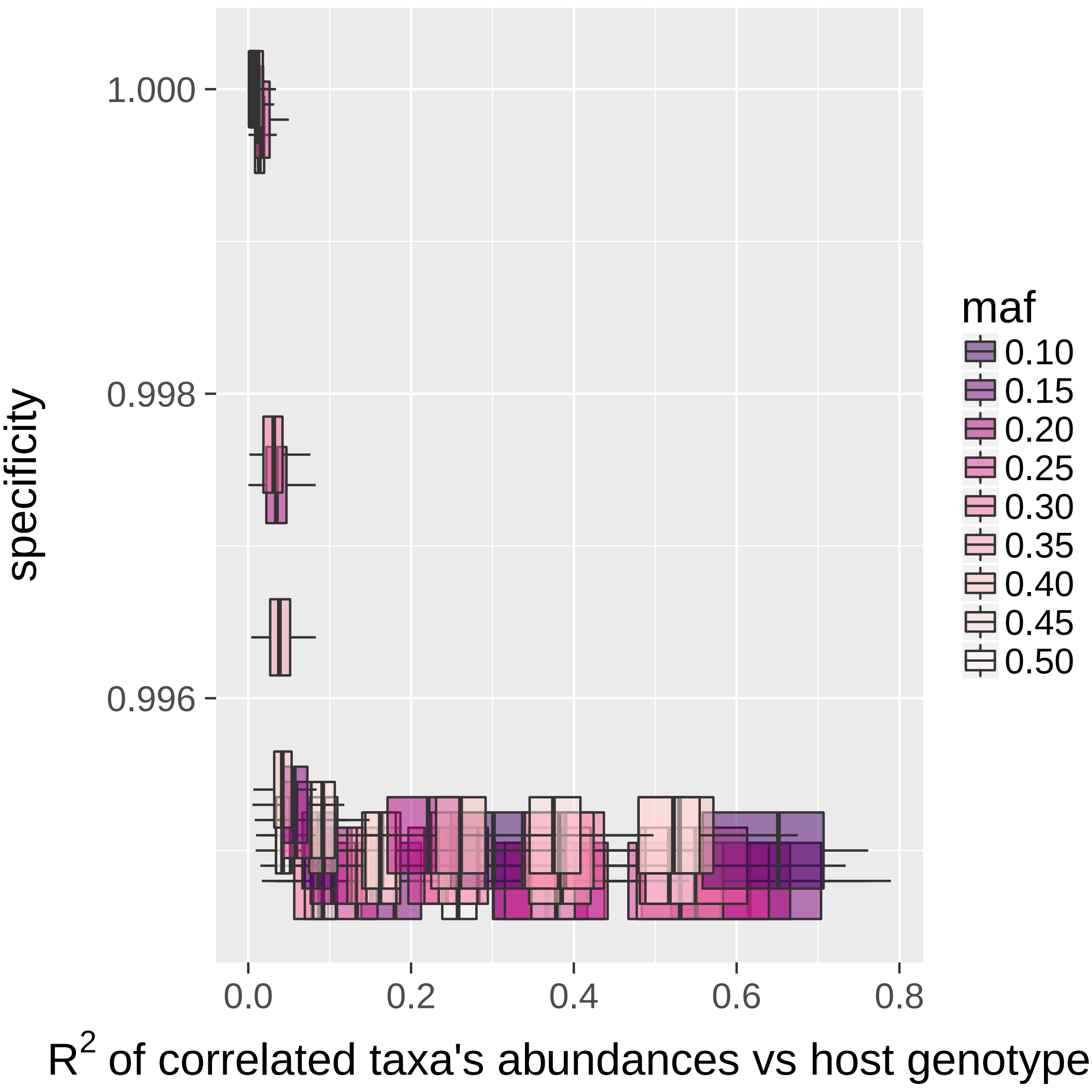

Figure S31: HOMINID performs equally well on data sets with different MAF: specificity as a function of input R^2, combining into the same plot data sets with MAF = 0.10, 0.15, 0.20, 0.25, 0.30, 0.35, 0.40, 0.45, and 0.50. Data sets with different MAF values are plotted in different colors. Data sets with the same MAF but different noise levels are plotted in the same color.

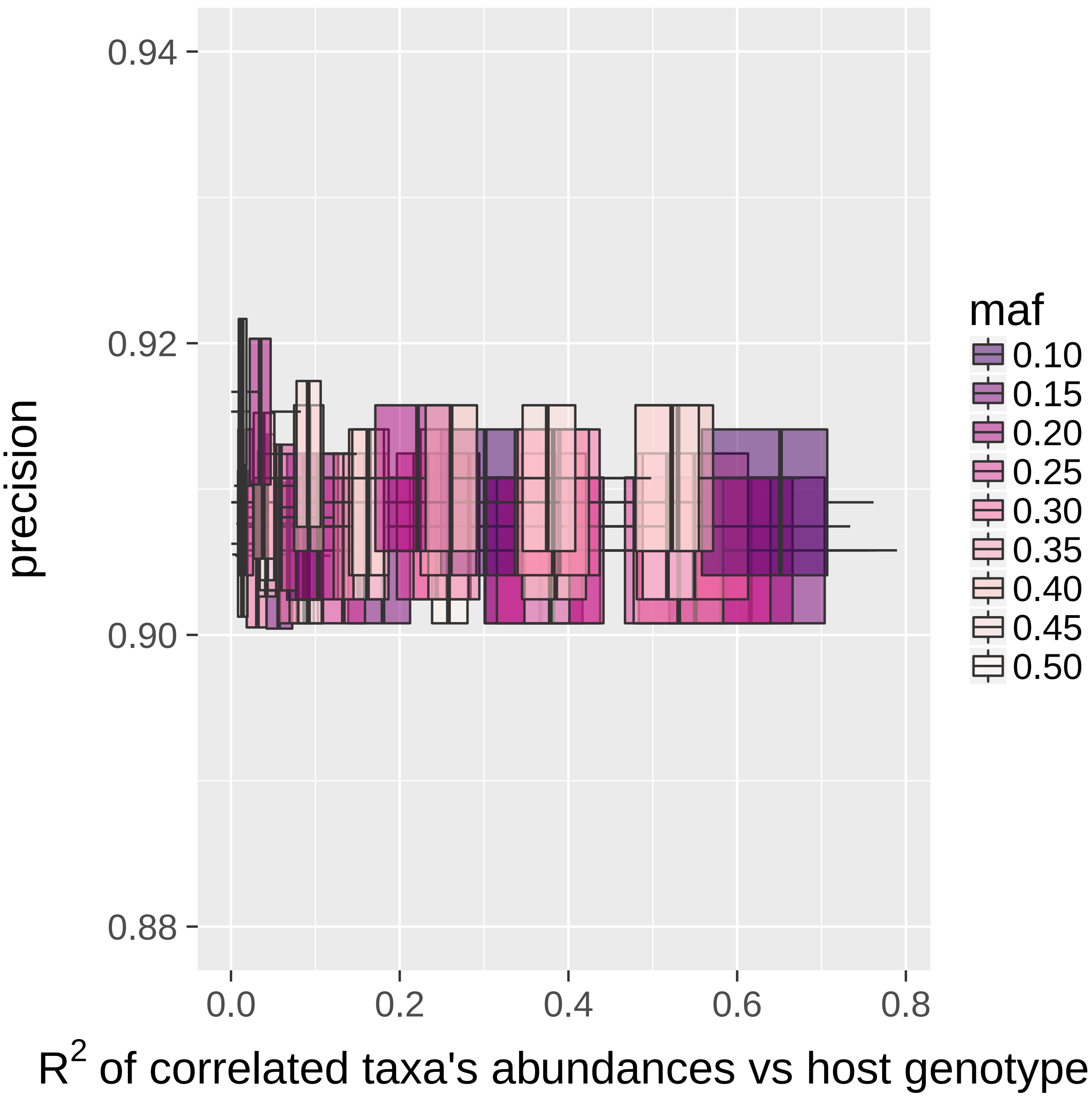

Figure S32: HOMINID performs equally well on data sets with different MAF: precision as a function of input R^2, combining into the same plot data sets with MAF = 0.10, 0.15, 0.20, 0.25, 0.30, 0.35, 0.40, 0.45, and 0.50. Data sets with different MAF values are plotted in different colors. Data sets with the same MAF but different noise levels are plotted in the same color.

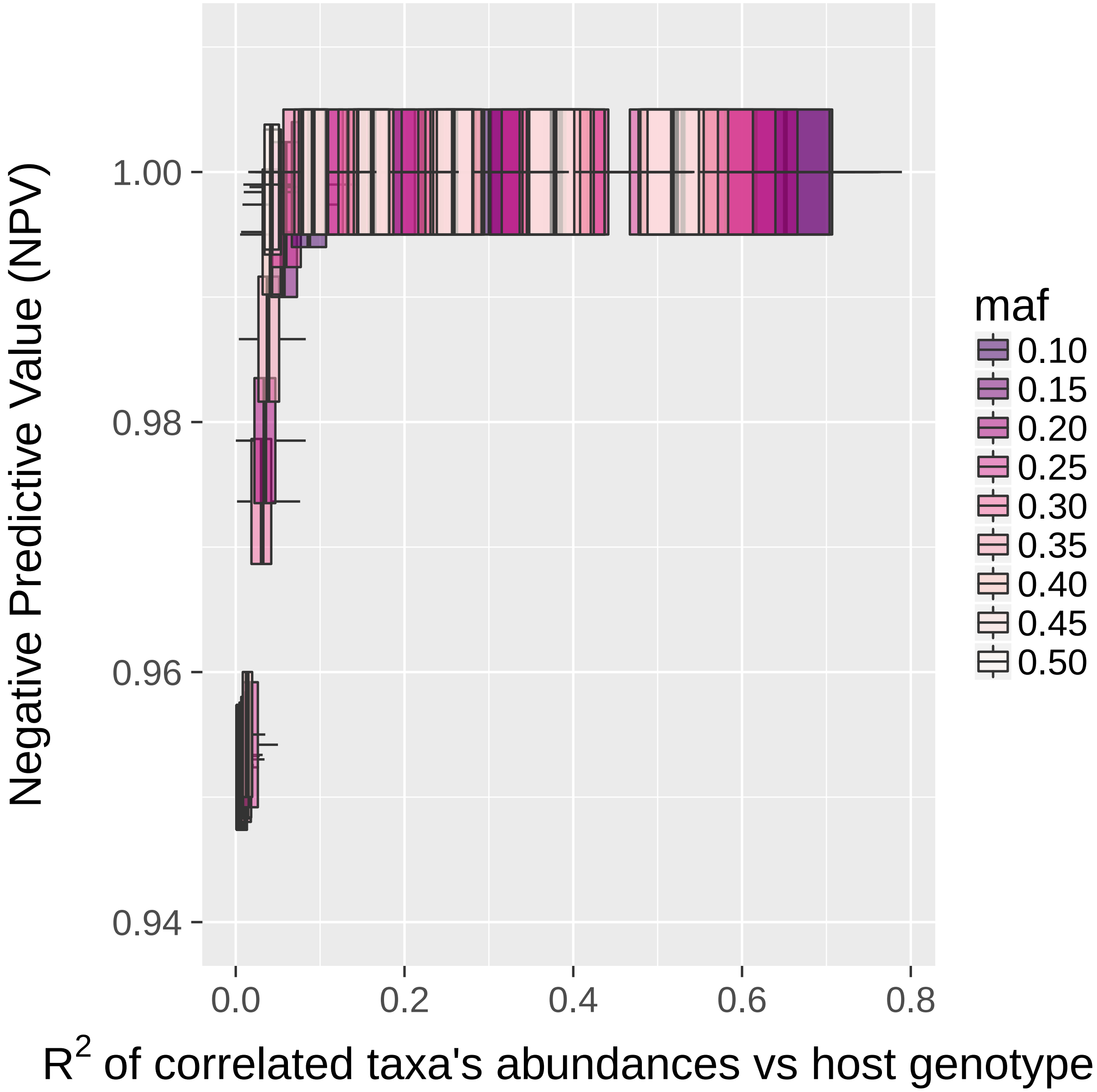

Figure S33: HOMINID performs equally well on data sets with different MAF: NPV as a function of input R^2, combining into the same plot data sets with MAF = 0.10, 0.15, 0.20, 0.25, 0.30, 0.35, 0.40, 0.45, and 0.50. Data sets with different MAF values are plotted in different colors. Data sets with the same MAF but different noise levels are plotted in the same color.

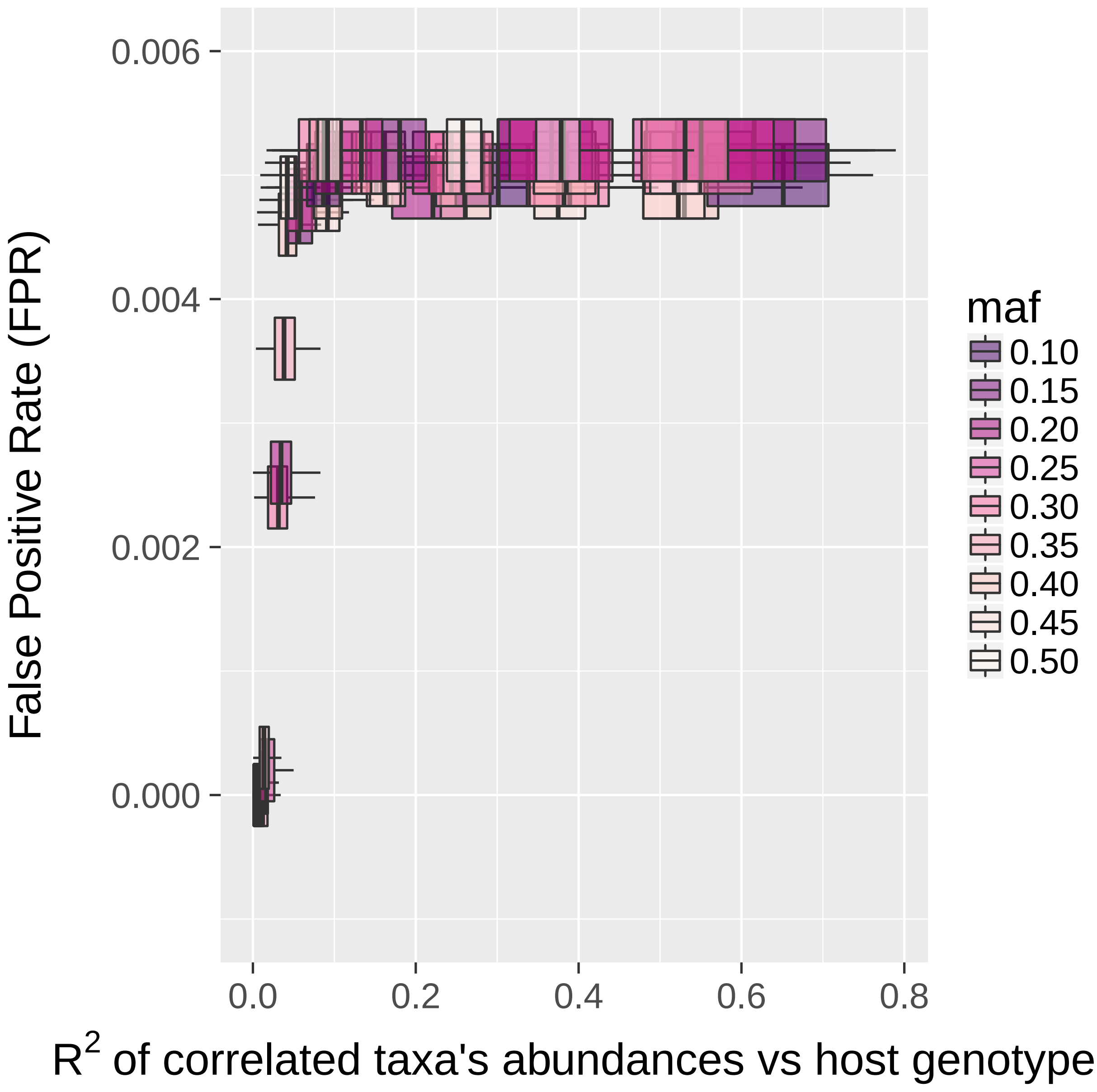

Figure S34: HOMINID performs equally well on data sets with different MAF: FPR as a function of input R^2, combining into the same plot data sets with MAF = 0.10, 0.15, 0.20, 0.25, 0.30, 0.35, 0.40, 0.45, and 0.50. Data sets with different MAF values are plotted in different colors. Data sets with the same MAF but different noise levels are plotted in the same color.

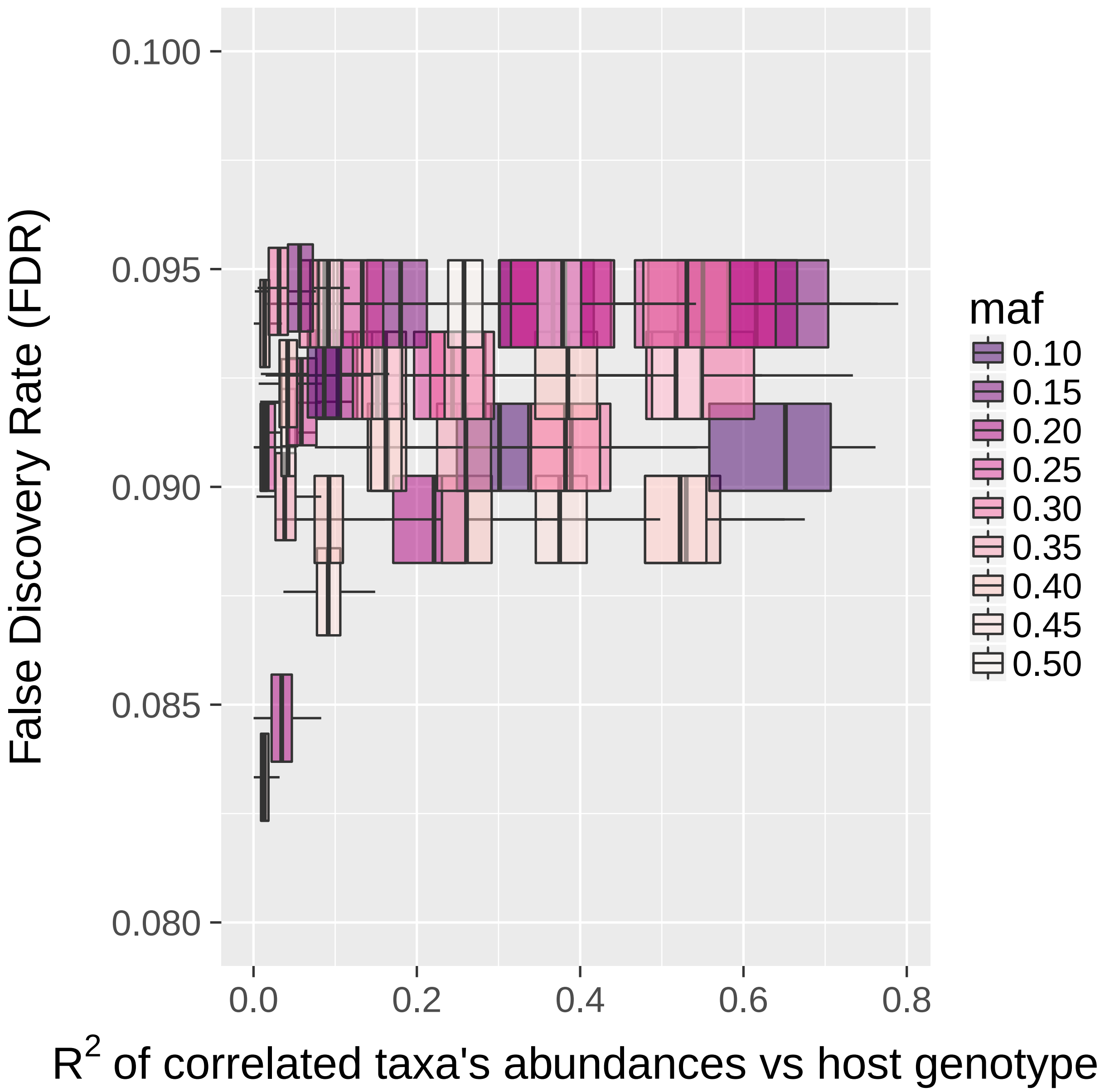

Figure S35: HOMINID performs equally well on data sets with different MAF: FDR as a function of input R^2, combining into the same plot data sets with MAF = 0.10, 0.15, 0.20, 0.25, 0.30, 0.35, 0.40, 0.45, and 0.50. Data sets with different MAF values are plotted in different colors. Data sets with the same MAF but different noise levels are plotted in the same color.

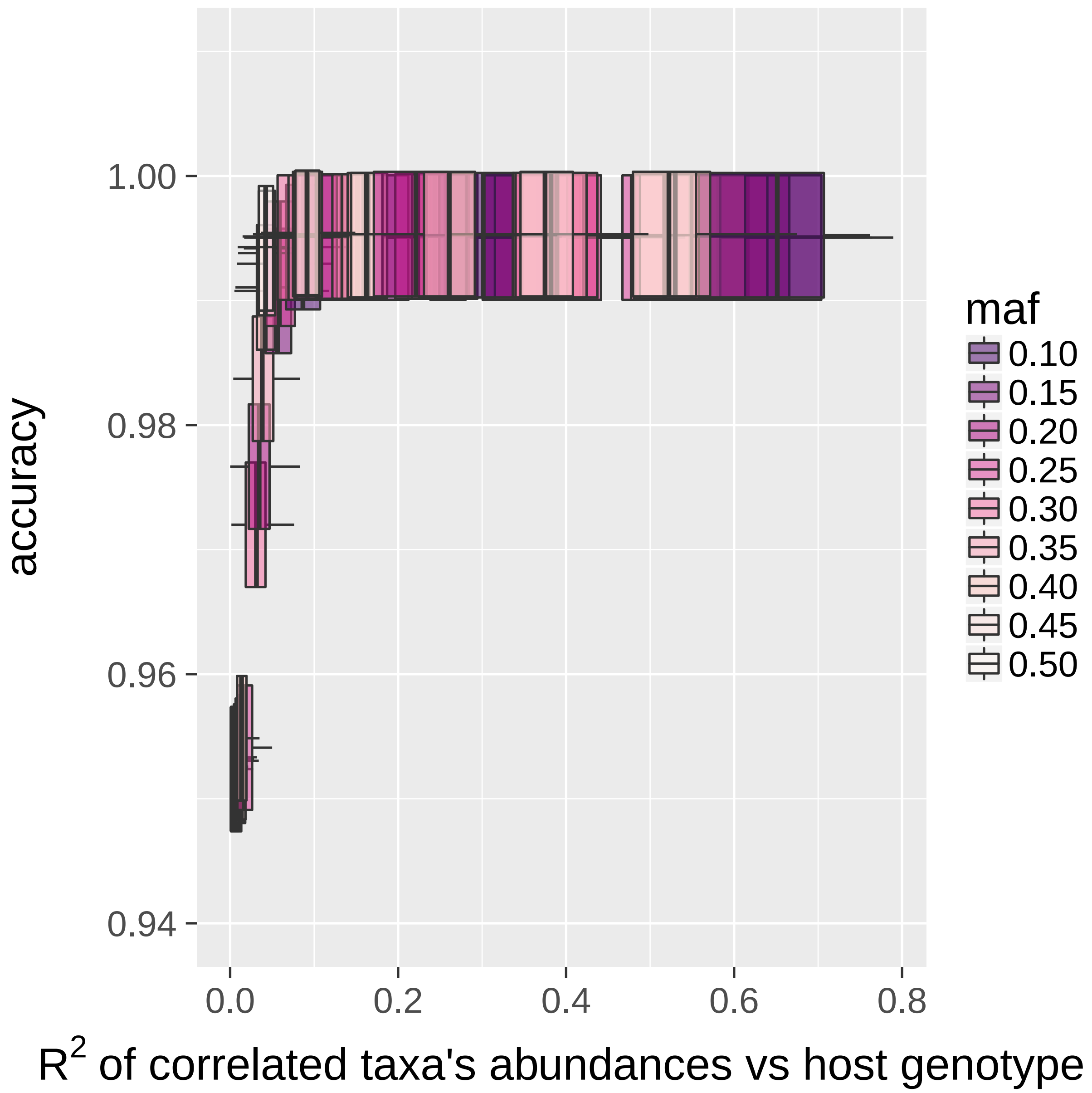

Figure S36: HOMINID performs equally well on data sets with different MAF: accuracy as a function of input R^2, combining into the same plot data sets with MAF = 0.10, 0.15, 0.20, 0.25, 0.30, 0.35, 0.40, 0.45, and 0.50. Data sets with different MAF values are plotted in different colors. Data sets with the same MAF but different noise levels are plotted in the same color.

2.9 HOMINID's Performance as a Function of Number of Correlated Taxa

To examine the effect of the number of correlated taxa on HOMINID's sensitivity, we compared results for data sets with varying numbers of correlated taxa, Correlated-Taxon Count (CTC) = 5, 10, 15, and 20. For all these data sets, MAF = 0.30 and there were 500 taxa, total (495, 490, 485, and 480 uncorrelated, respectively). See section 2.3 for a detailed description of these data sets.

Figure S37 shows the sensitivity for prediction of which SNPs are correlated. Note that this is not the sensitivity for feature selection (i.e. predicting which taxa are correlated which was shown in figures S15 and S16.) The different CTC values are shown as different colored boxplots; different noise levels but same CTC are plotted in the same color.

At high R^2_{OLS}, sensitivity is perfect (= 1). At $R^2_{OLS} \lesssim 0.12$, sensitivity is poorer when there are more correlated taxa. We suspect this is because, at the same overall input R^2, if there are many correlated taxa, on average each taxon has a weaker correlation to the host genetics than if there are few correlated taxa. HOMINID's ability to detect correlated taxa does depend on the strength of correlation between the taxa and host genetics (see section 2.11).

Figures S38, S39, S40, S41, S42, S43, and S44 show other metrics of HOMINID's performance, again with different CTC values shown in different colors.

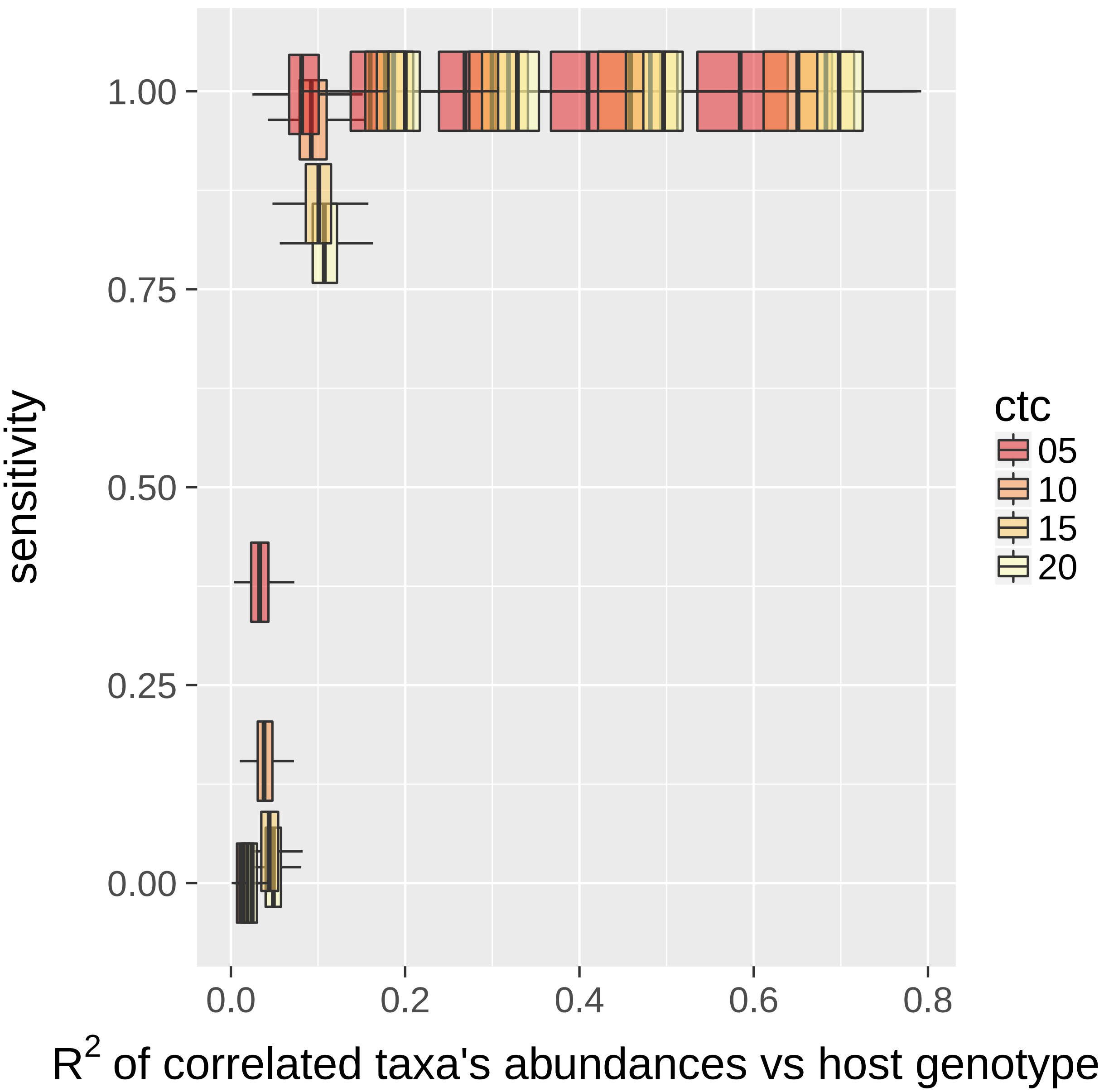

Figure S37: At low R^2_{OLS}, HOMINID performs slightly less well when there are more correlated taxa. Sensitivity as a function of input R^2, combining into the same plot data sets with different numbers of correlated taxa, Correlated Taxon Count (CTC) = 5, 10, 15, and 20. Data sets with different CTC values are plotted in different colors. Data sets with the same CTC but different noise levels are plotted in the same color.

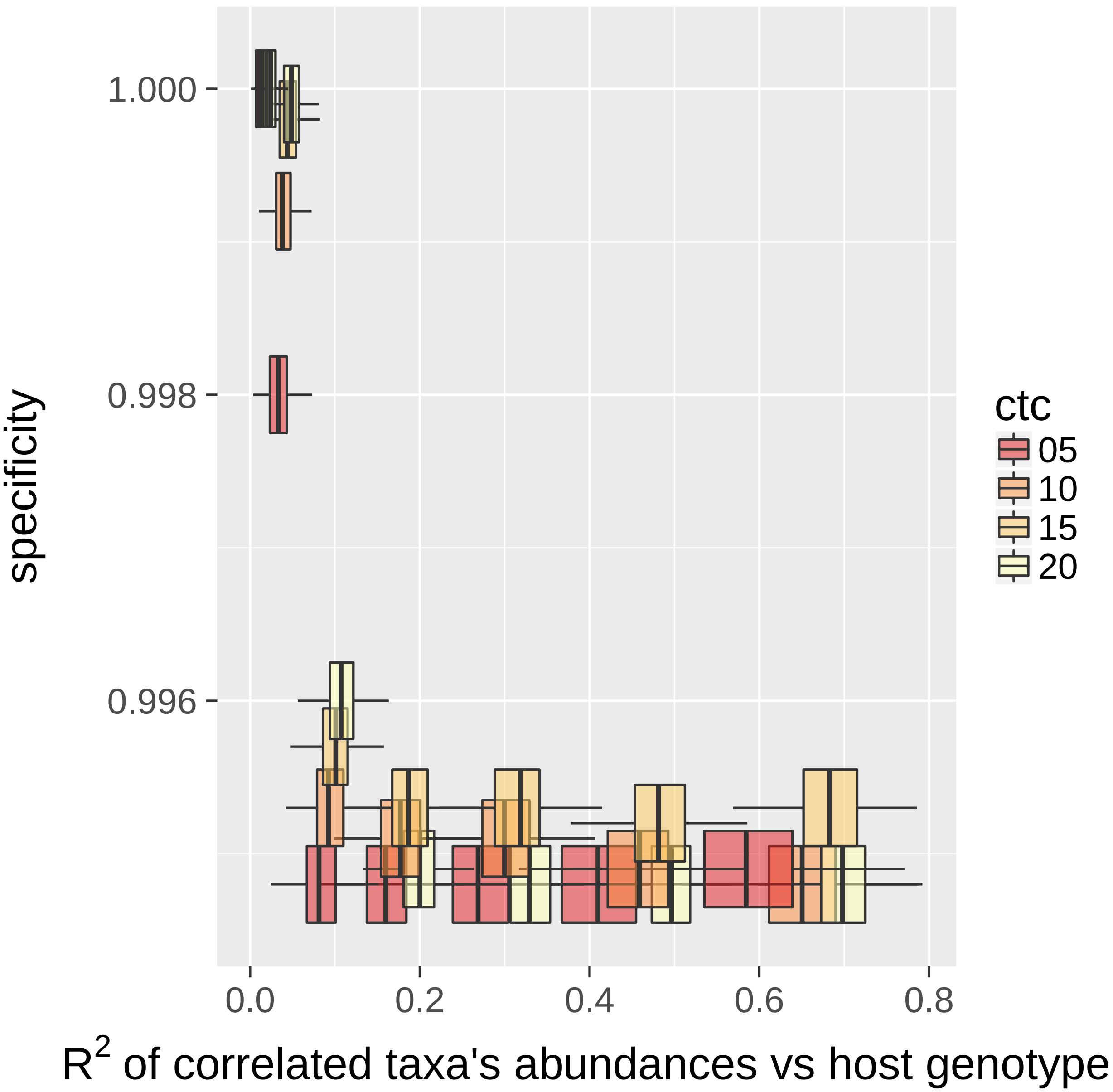

Figure S38: Specificity, for data sets with different numbers of correlated taxa (CTC).

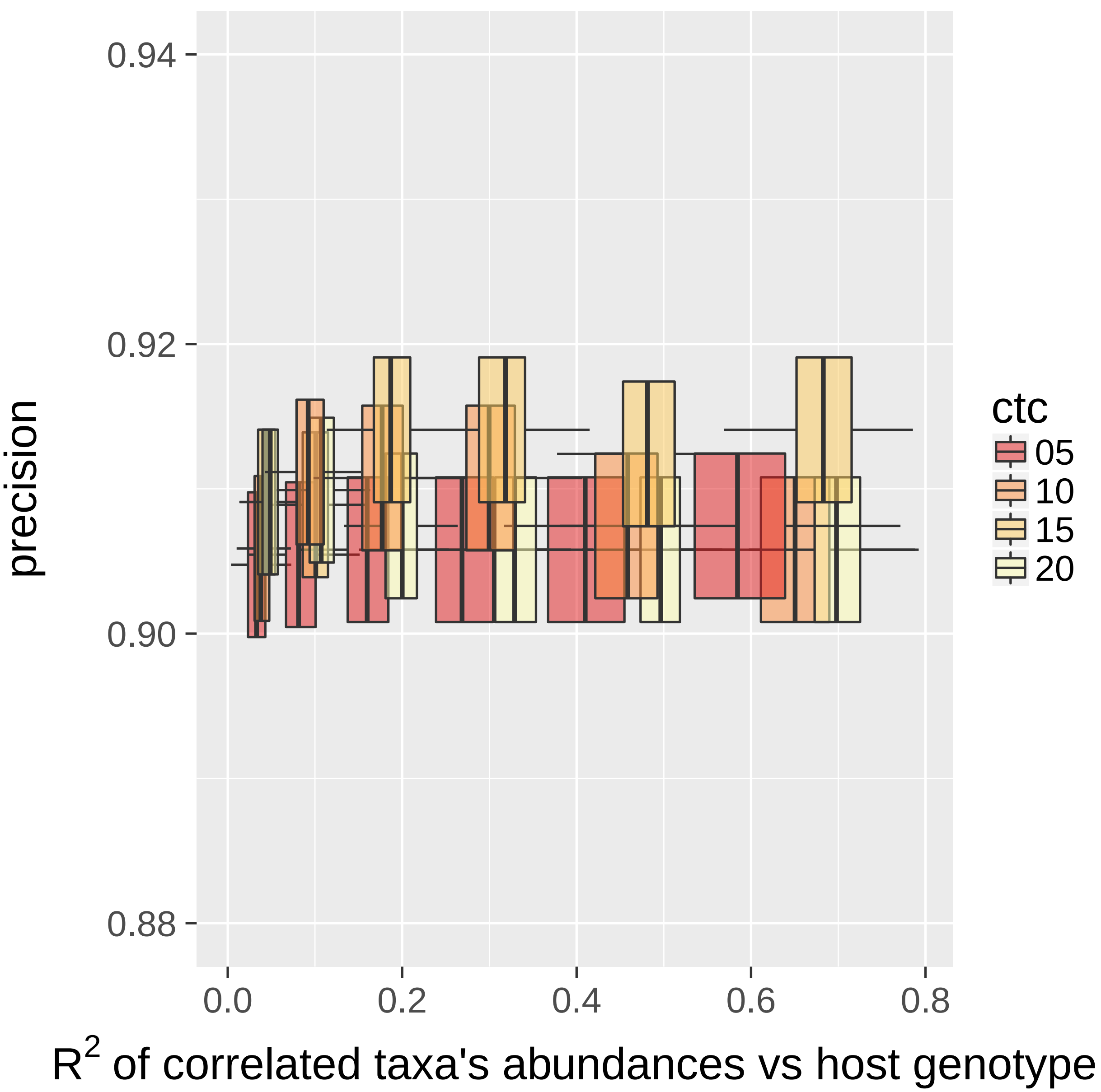

Figure S39: Precision, for data sets with different numbers of correlated taxa (CTC).

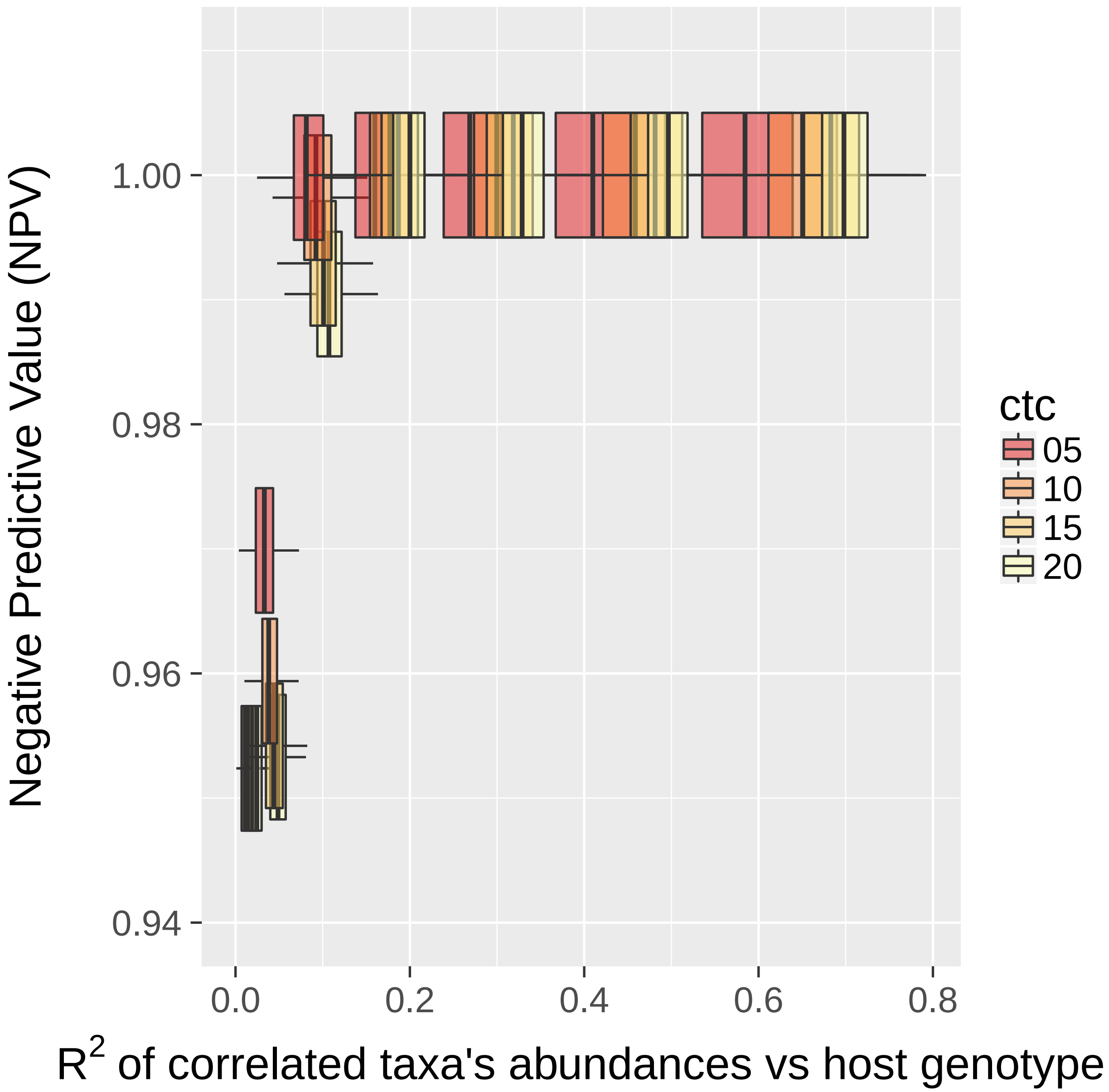

Figure S40: NPV, for data sets with different numbers of correlated taxa (CTC).

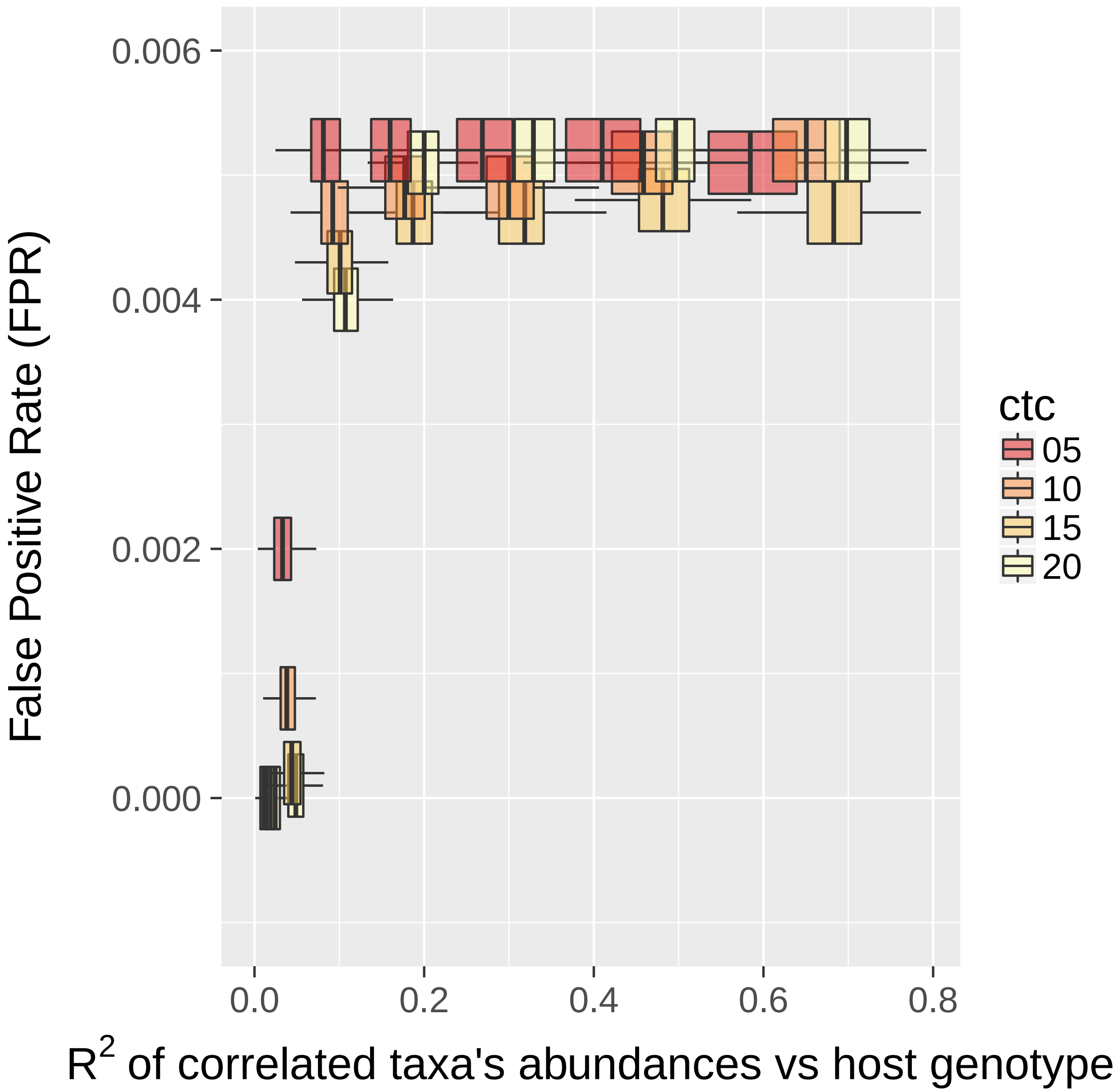

Figure S41: FPR, for data sets with different numbers of correlated taxa (CTC).

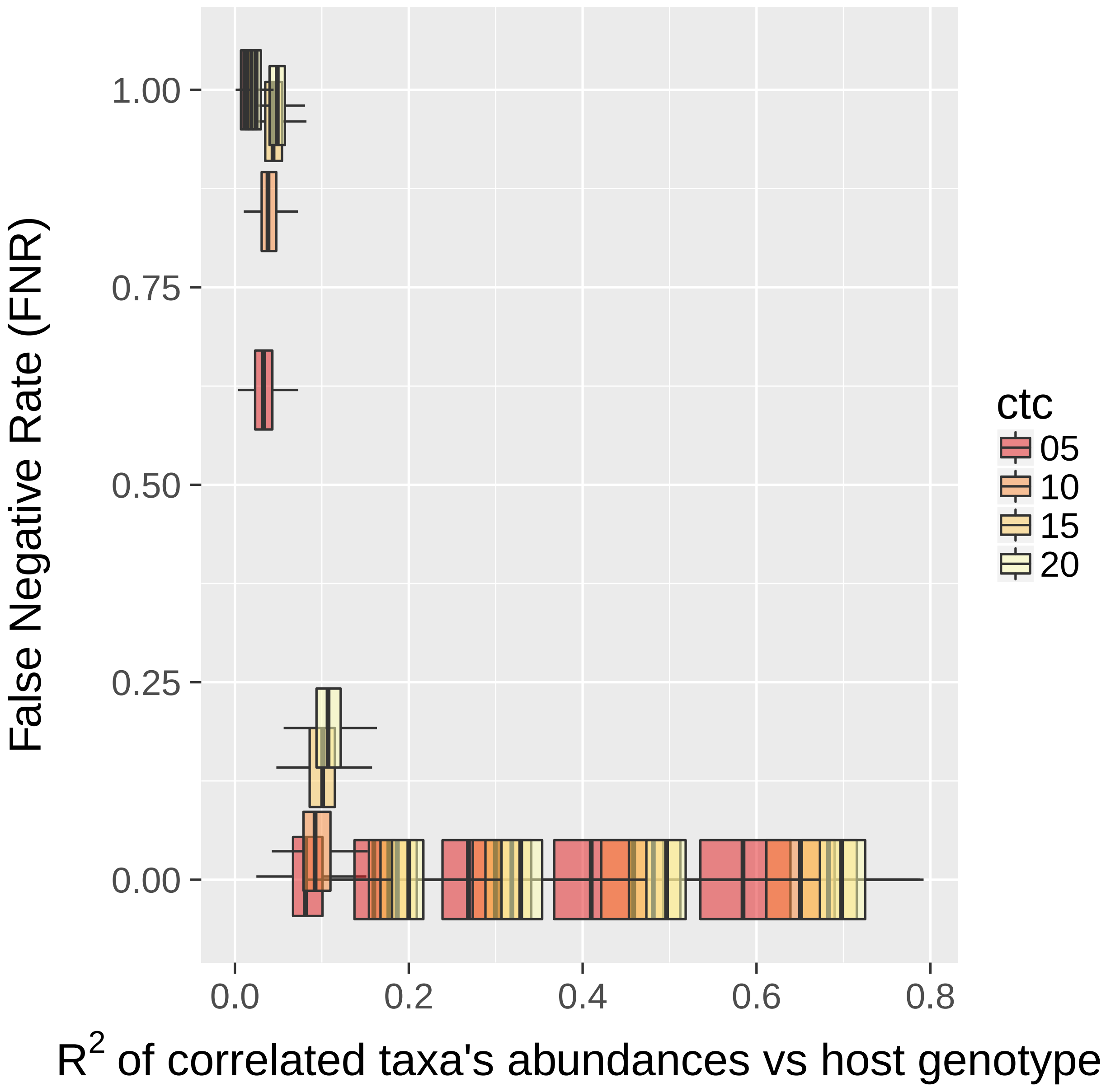

Figure S42: FNR, for data sets with different numbers of correlated taxa (CTC).

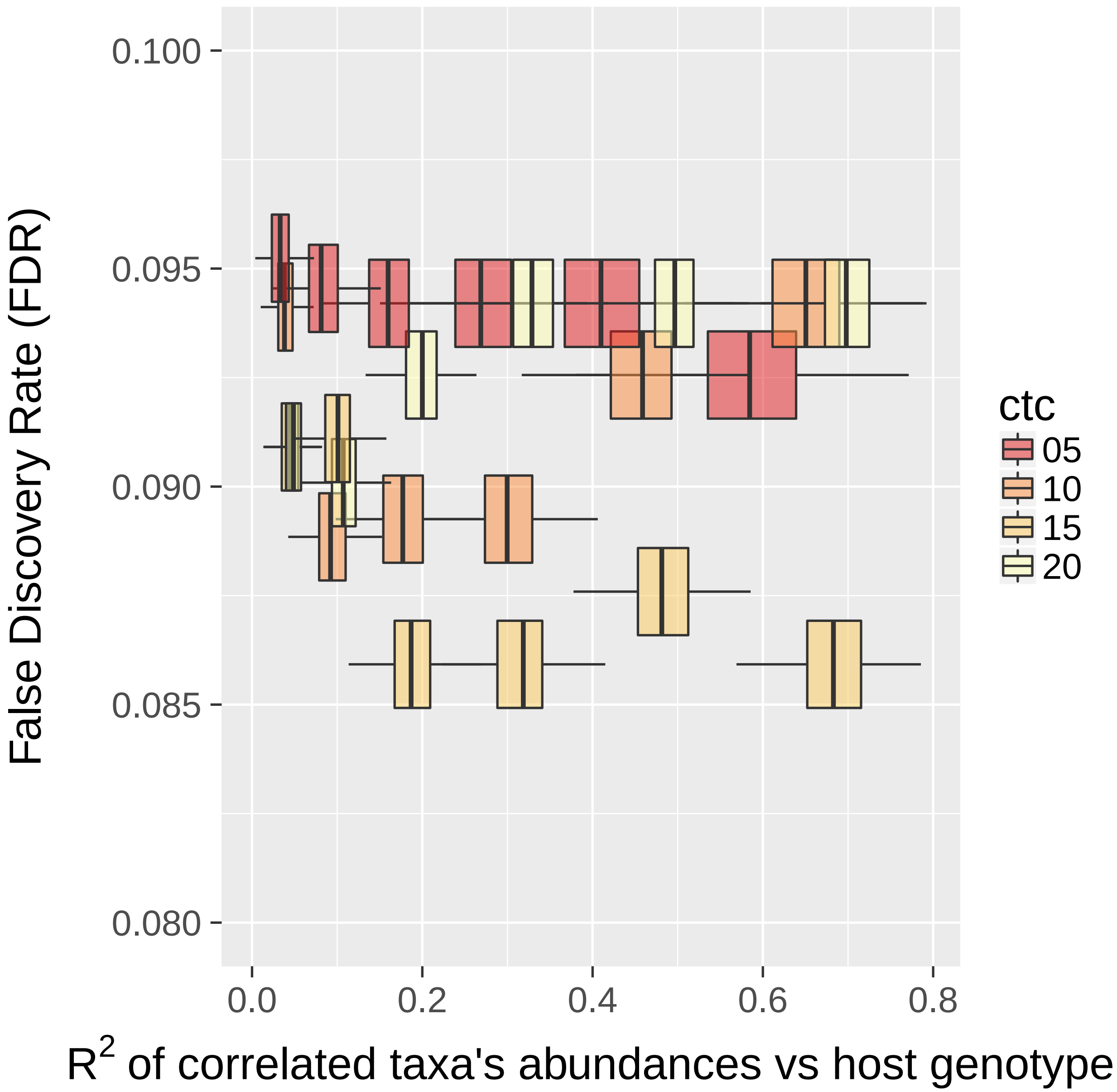

Figure S43: FDR, for data sets with different numbers of correlated taxa (CTC).

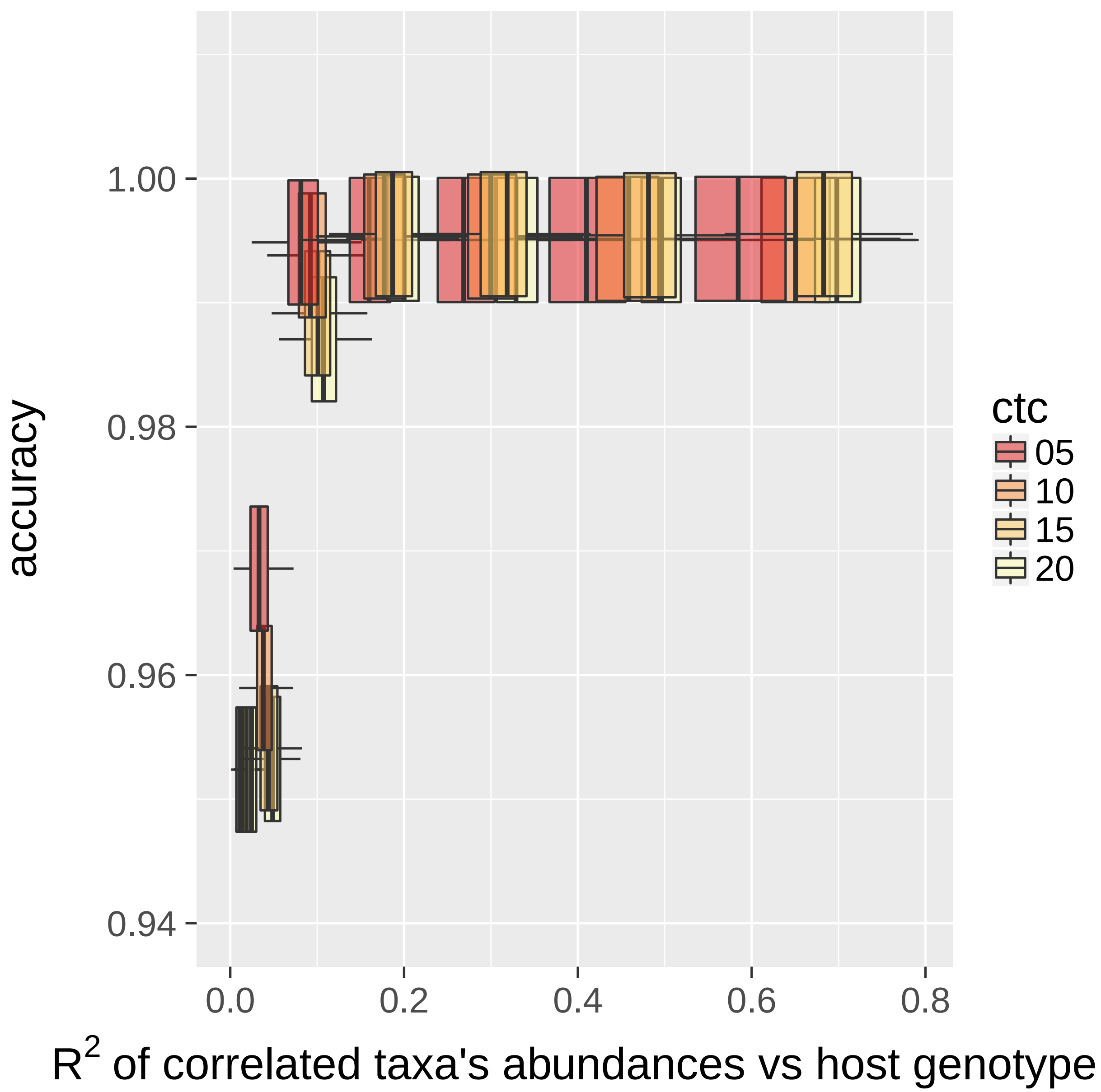

Figure S44: Accuracy, for data sets with different numbers of correlated taxa (CTC).

2.10 Synthetic Dataset TC

We constructed this dataset to investigate Lasso regression with varying total taxon count and SNP noise. This dataset consisted of 9 subsets of 500 correlated SNPs generated from all pairings of total taxon counts 100, 300, and 500 and SNP noise levels of 0.00, 0.05, and 0.10. Three subsets of 10,000 uncorrelated SNPs were generated, one for each total taxon count. The SNP minor allele frequency in all subsets was fixed at 0.30.

Figures S45 and S46 show sensitivity and specificity as functions of total taxon count. There is some fluctuation in sensitivity but no clear dependence on total taxon count or

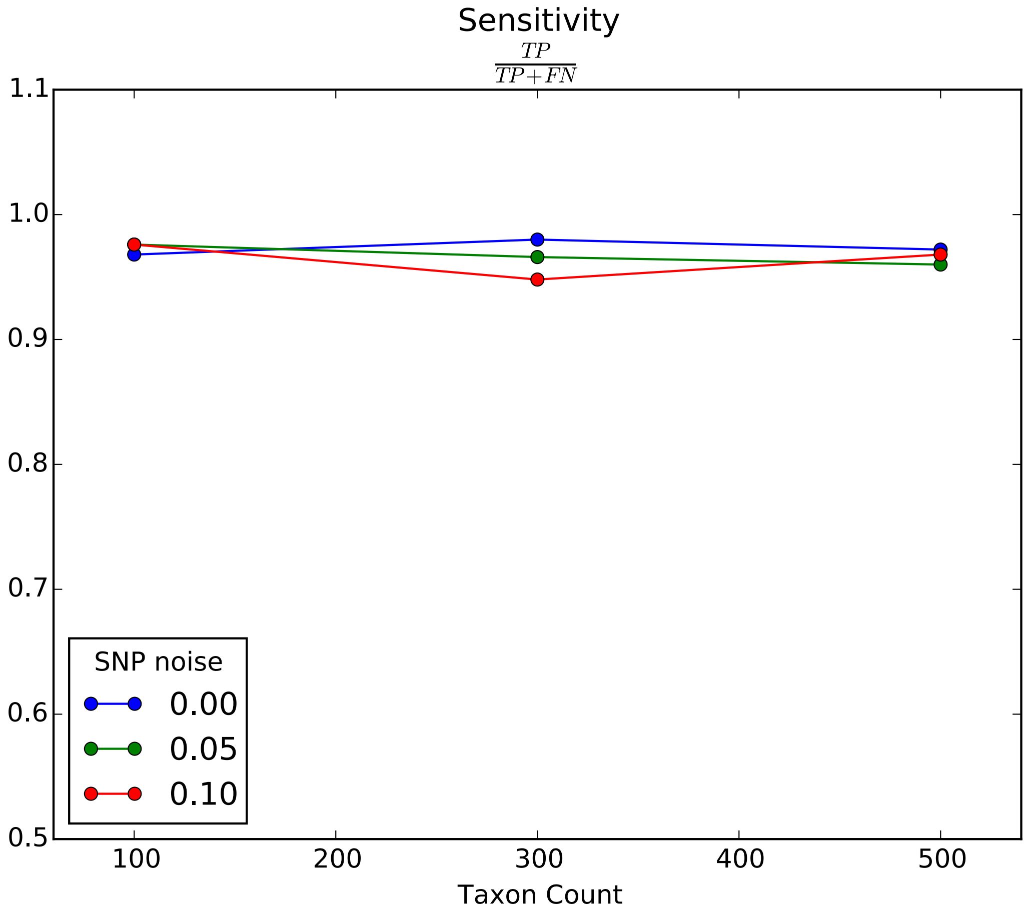

Figure S45: Sensitivity as a function of total taxon count in synthetic dataset TC.

SNP noise. Specificity is consistent across the different taxon counts.

This means our method is good at identifying SNPs correlated with taxon abundances even at larger taxon counts and in the presence of noise.

Figure S47 shows mean precision and mean recall per SNP for feature selection on synthetic dataset TC. The precision of feature selection is largely unaffected by the total taxon

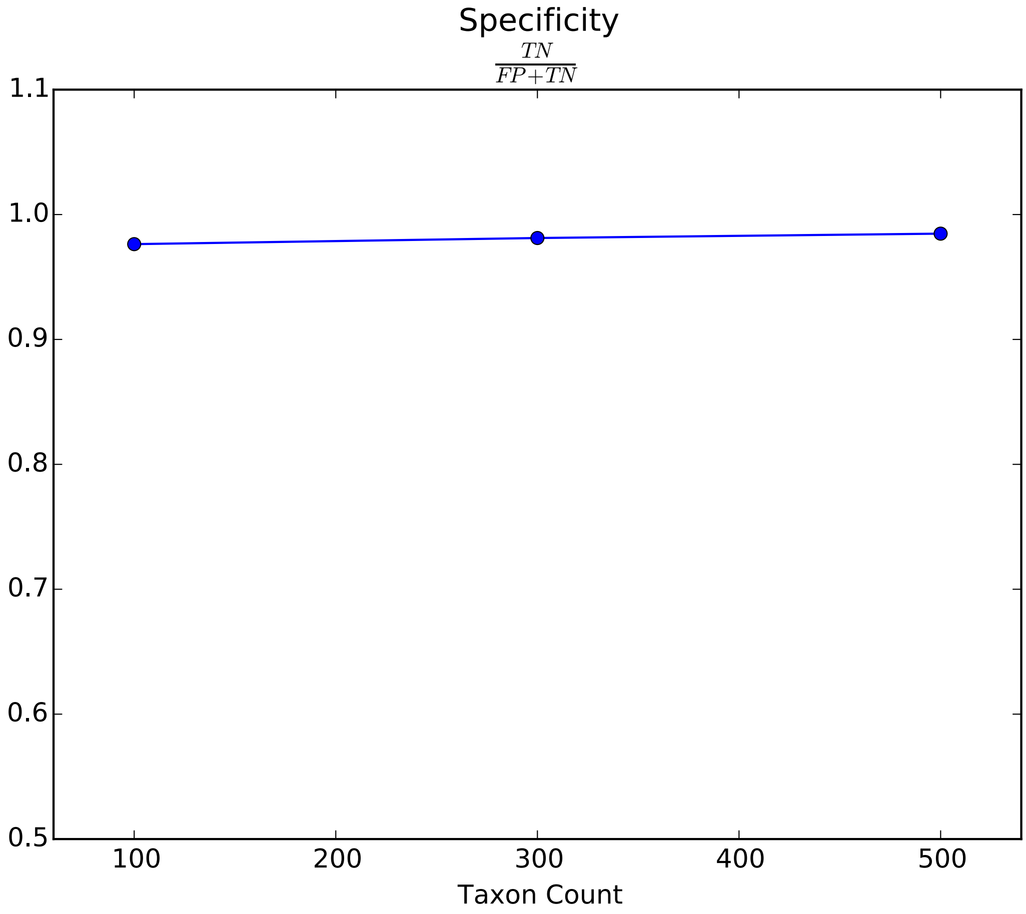

Figure S46: Specificity as a function of total taxon count in synthetic dataset TC.

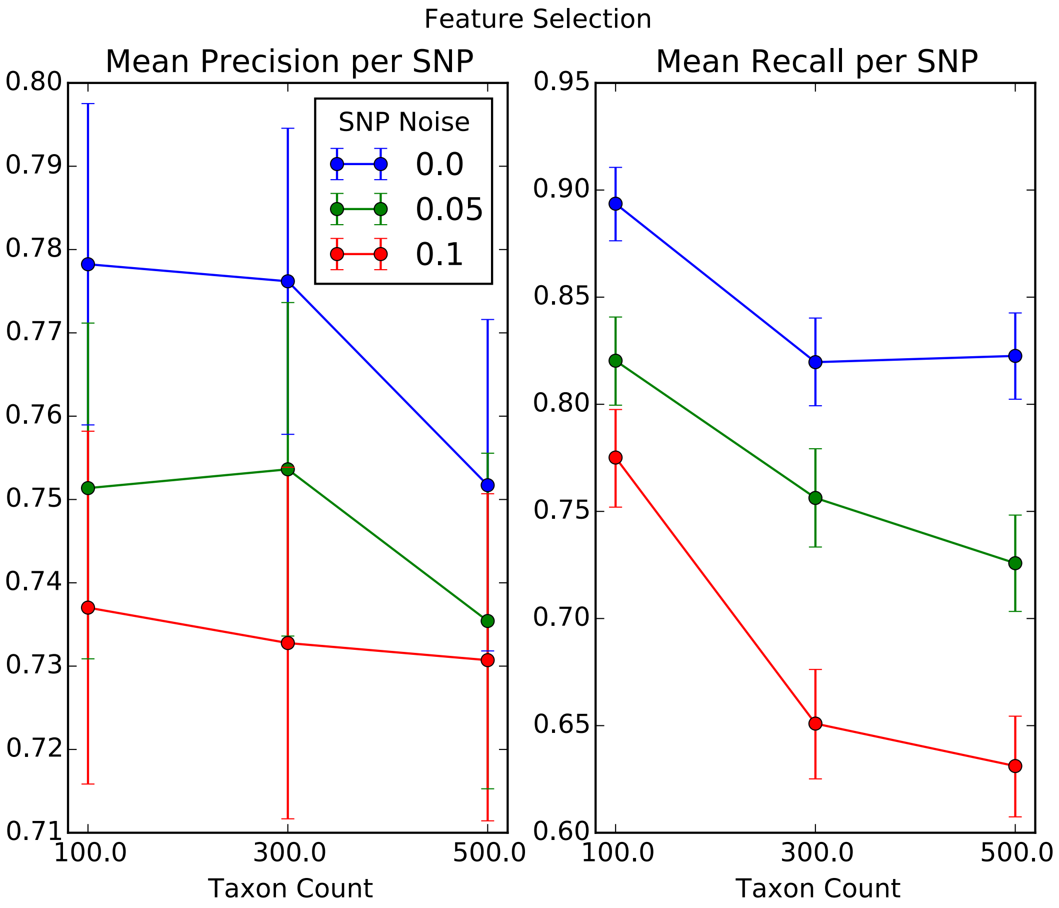

Figure S47: Mean feature selection precision and recall per SNP with 95% confidence intervals for synthetic dataset TC.

count and SNP noise has a consistent but small effect. Feature selection recall is sensitive to increasing taxon table size and to increasing SNP noise.

2.11 Assessing How Well HOMINID Detects Rare Correlated Taxa

To see whether HOMINID is able to detect correlated taxa that are rare (low abundance), we compared the abundances of correlated taxa that are predicted to be False Negative (FN; correlated taxa incorrectly predicted to be uncorrelated) versus taxa that are predicted to be True Positives (TP; correlated taxa correctly predicted to be correlated).

Figure S48 shows that the abundances of taxa predicted to be FN versus TP are the same. There is no bias of FNs toward lower-abundance taxa.

In figure S49, we plot FN and TP versus the taxon coefficients, c_i, from the linear function $f(s)$ on page 11. $|c_i| \sim 0$ means that taxon i is weakly correlated with the host genotypes. In figure S49, we see that FNs have $|c_i| \sim 0$ whereas TPs have larger $|c_i|$ values. Therefore what affects whether a taxon gets detected as correlated to the host genetics is the degree to which that taxon correlates with the host genotypes, weakly correlated taxa being less likely to be detected.

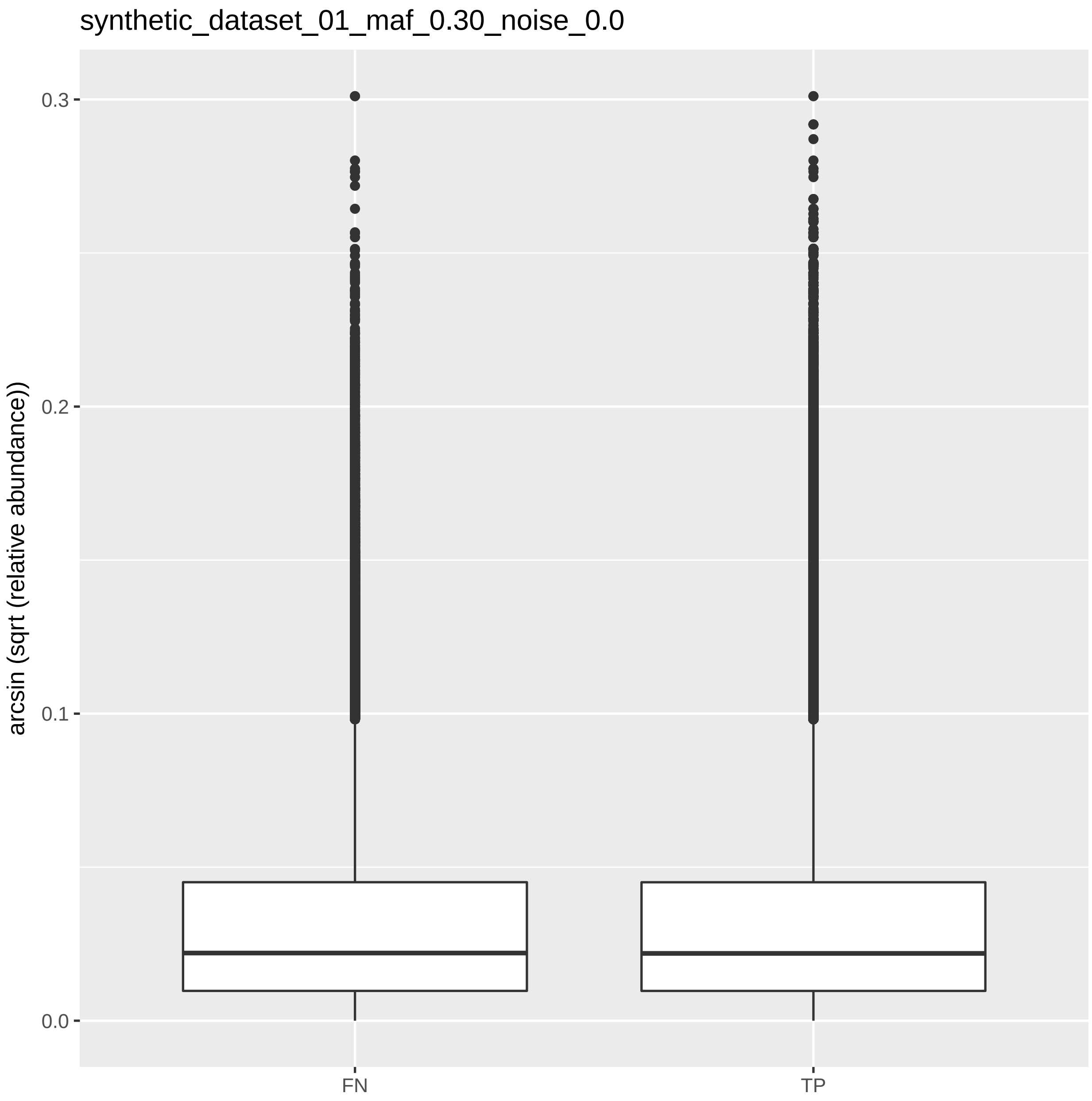

Figure S48: Comparing False Negatives (FNs, correlated taxa incorrectly predicted to be uncorrelated) and True Positives (TPs, correlated taxa correctly predicted to be correlated) versus taxon abundance: There is no difference between the abundances of FNs versus TPs, indicating that taxon abundance does not affect whether the taxon is correctly predicted to be correlated.

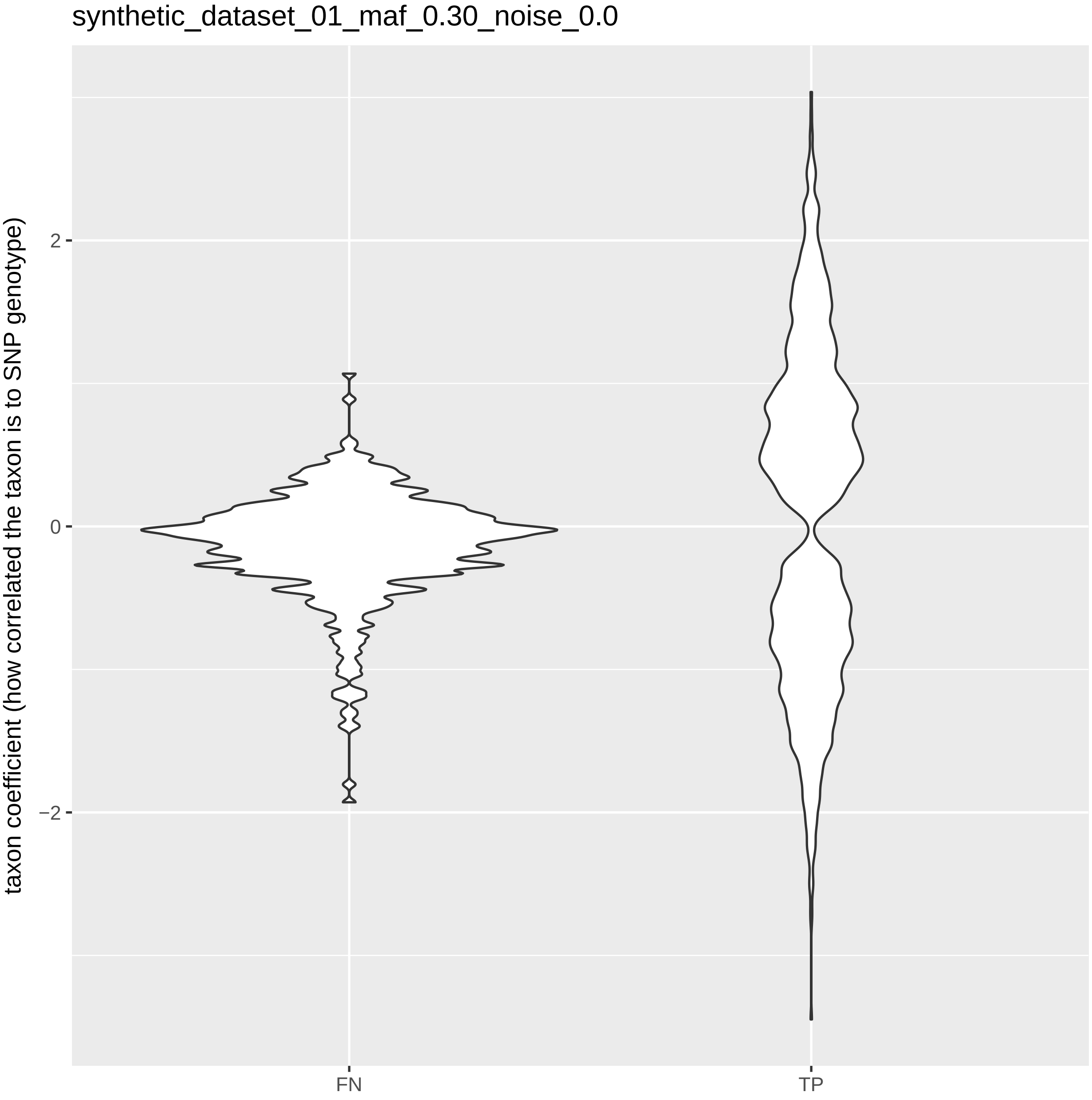

Figure S49: Comparing False Negatives (FNs, correlated taxa incorrectly predicted to be uncorrelated) and True Positives (TPs, correlated taxa correctly predicted to be correlated) versus taxon coefficient c_i, the degree to which the taxon correlates with host genotype: Weakly correlated taxa ($|c_i| \sim 0$) are more likely to be FN, incorrectly predicted to be uncorrelated.

2.12 Comparison to PERMANOVA and MiRKAT

To see how well HOMINID performs as compared to other methods, we ran PERMANOVA [1, 9] and MiRKAT [17] on the data set with MAF = 0.30 and 500 total taxa, three correlated and 497 uncorrelated. MiRKAT applies a semi-parametric kernel machine regression framework to evaluate the association, and is closely related to distance-based methods, such as PERMANOVA. We only evaluate how well these methods predict the SNPs that are correlated to the microbiome since neither PERMANOVA nor MiRKAT predicts the specific taxa that associate.

The PERMANOVA analysis was done in R with the adonis function in the vegan package. The SNP genotypes were numeric, not factors. In the formula for adonis, the arcsin-sqrt-transformed taxon table was set as the response variable and each SNP served as the predictor variable: `adonis (arcsinSqrtTaxonTable ~ SNP, method="bray", permutations=10000)`.

The MiRKAT analysis was performed using the MiRKAT package in R. First the Bray-Curtis dissimilarity matrix was computed on the arcsin-sqrt transformed taxon table: `D.BC = as.matrix(vegdist(arcsinSqrtTaxonTable, method="bray"))`. Then the dissimilarity matrix was converted to kernel matrix using the D2K function: `K.BC = D2K(D.BC)`. The MiRKAT() function was invoked for each SNP using the Bray-Curtis kernel computed above: `MiRKAT(y = SNP, Ks = K.BC, out_type = "C", method = "permutation", nperm = 10000)`. Thus, the association between each SNP and the overall microbiome composition was evaluated using 10,000 permutations and corresponding p-value was obtained.

Both PERMANOVA and MiRKAT were run on the 500 correlated SNPs in each data set, and on 10,000 permuted/uncorrelated SNPs.

For PERMANOVA and MiRKAT, we chose which SNPs are predicted positive/correlated versus negative/uncorrelated in a manner similar to the way we did with HOMINID. PERMANOVA and MiRKAT both output p-values as metrics of how well the taxon abundances correlate with the host genetics, whereas HOMINID outputs R_L^2. Similarly to how we chose the cutoff value, R_c^2, that separate prediction positive from prediction negative for HOMINID (see p. 4) we chose a p-value cutoff, p_c, such that $q(p_c) = 0.1$[4]. SNPs with p-value $\leq p_c$ are predicted positive (correlated) and SNPs with p-value $> p_c$ are predicted negative (uncorrelated).

Figure S50 shows that, at moderate-to-high input R^2 ($R_{OLS}^2 \gtrsim 0.12$), all three methods perform similarly and find most or all 500 correlated SNPs.

However, at low input R^2 ($R_{OLS}^2 \lesssim 0.10$), HOMINID has higher sensitivity than PERMANOVA and MiRKAT. For the noise 0.4 dataset (symbol ⊠; first quartile, median, and third quartile of R_{OLS}^2 equal to 0.056, 0.080, and 0.098, respectively), HOMINID's sensitivity is 1.0 whereas PERMANOVA's and MiRKAT's sensitivities are 0.29 and 0.19, respectively; HOMINID picked out all 500 correlated SNPs, whereas PERMANOVA correctly predicted

[4]In practice, because the p-value is calculated to finite precision, p_c is chosen to be the largest p-value such that $q(p_c) \leq 0.1$

144/500 and MiRKAT 93/500.

Also, HOMINID has an advantage over PERMANOVA and MiRKAT in that HOMINID predicts both the SNPs and *the taxa that are correlated to each associated SNP*, whereas PERMANOVA and MiRKAT only predict the SNPs that are correlated.

Figures S51, S52, S53, S54, S55, S56, and S57 show other statistical measures of performance of HOMINID, PERMANOVA and MiRKAT.

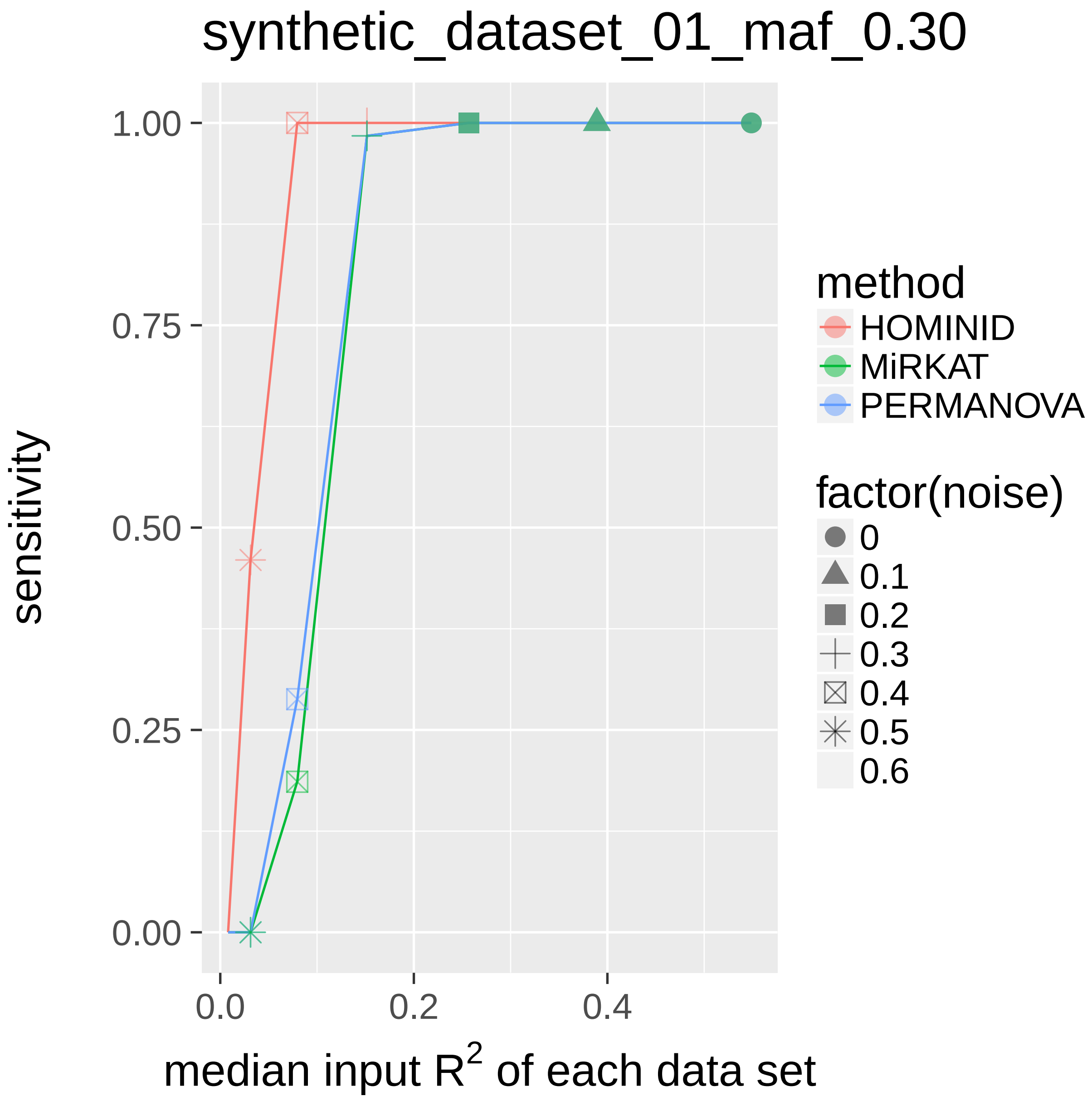

Figure S50: Comparision of sensitivity for HOMINID, PERMANOVA and MiRKAT.

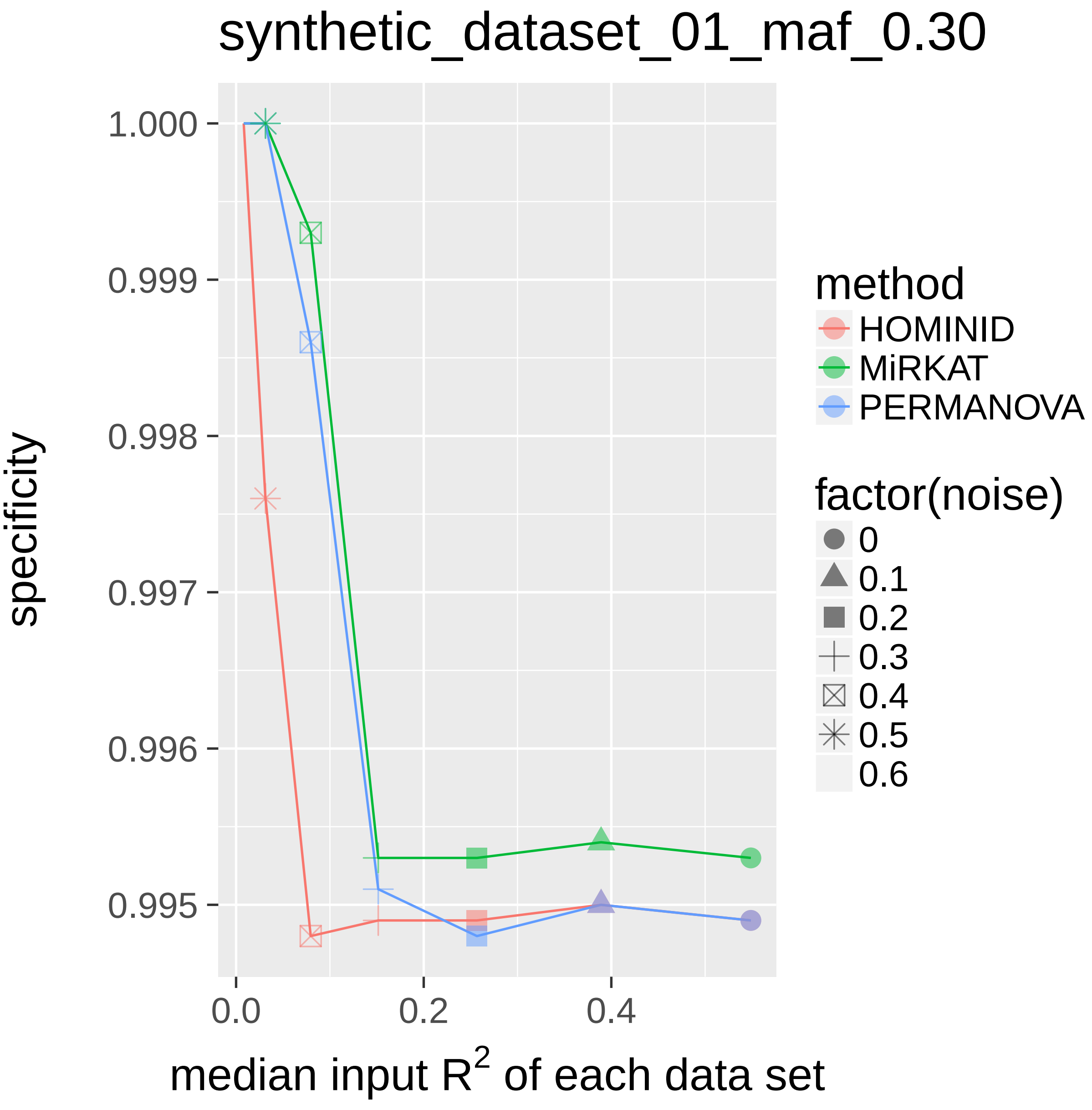

Figure S51: Comparision of specificity for HOMINID, PERMANOVA and MiRKAT.

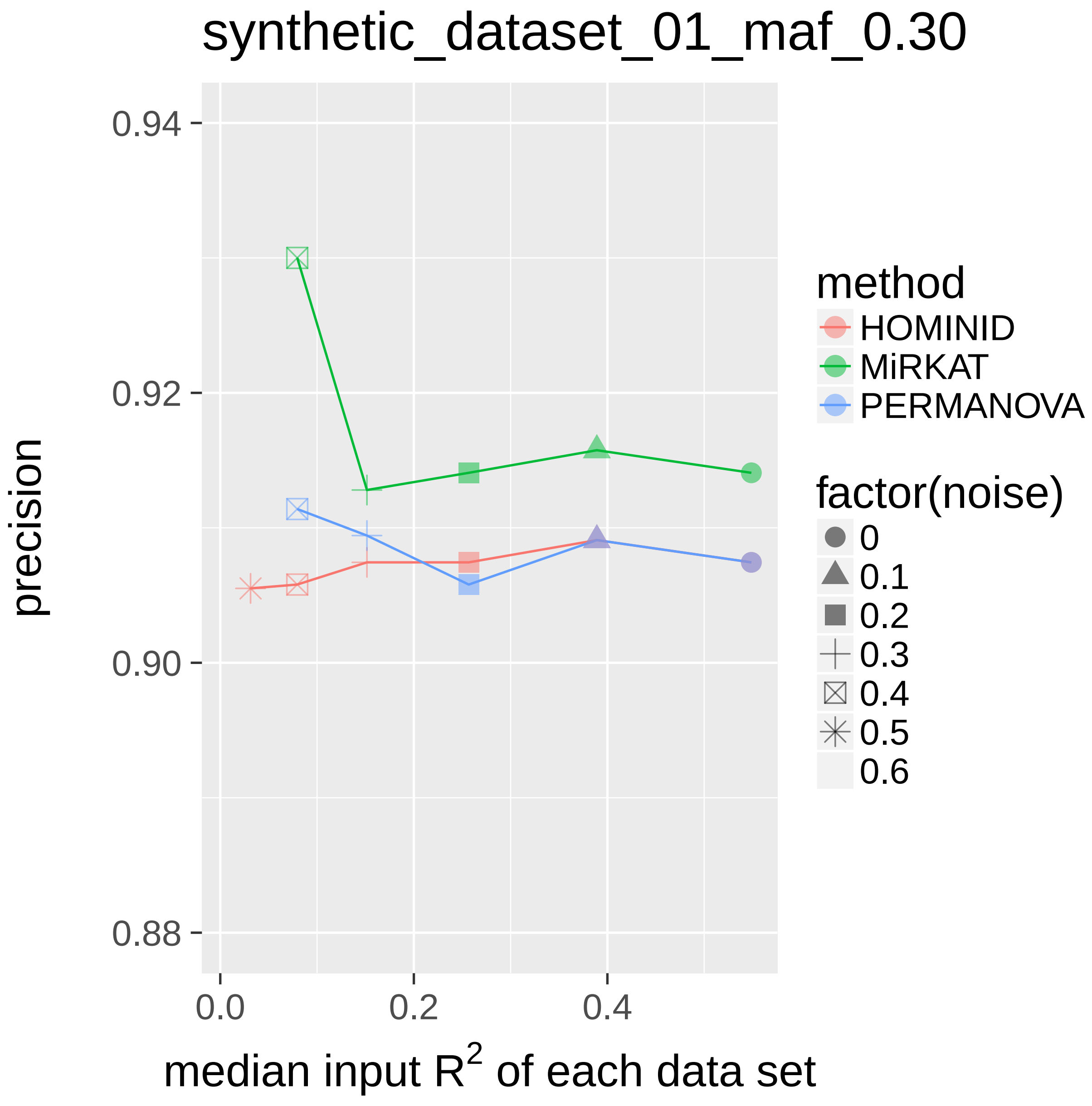

Figure S52: Comparision of precision for HOMINID, PERMANOVA and MiRKAT.

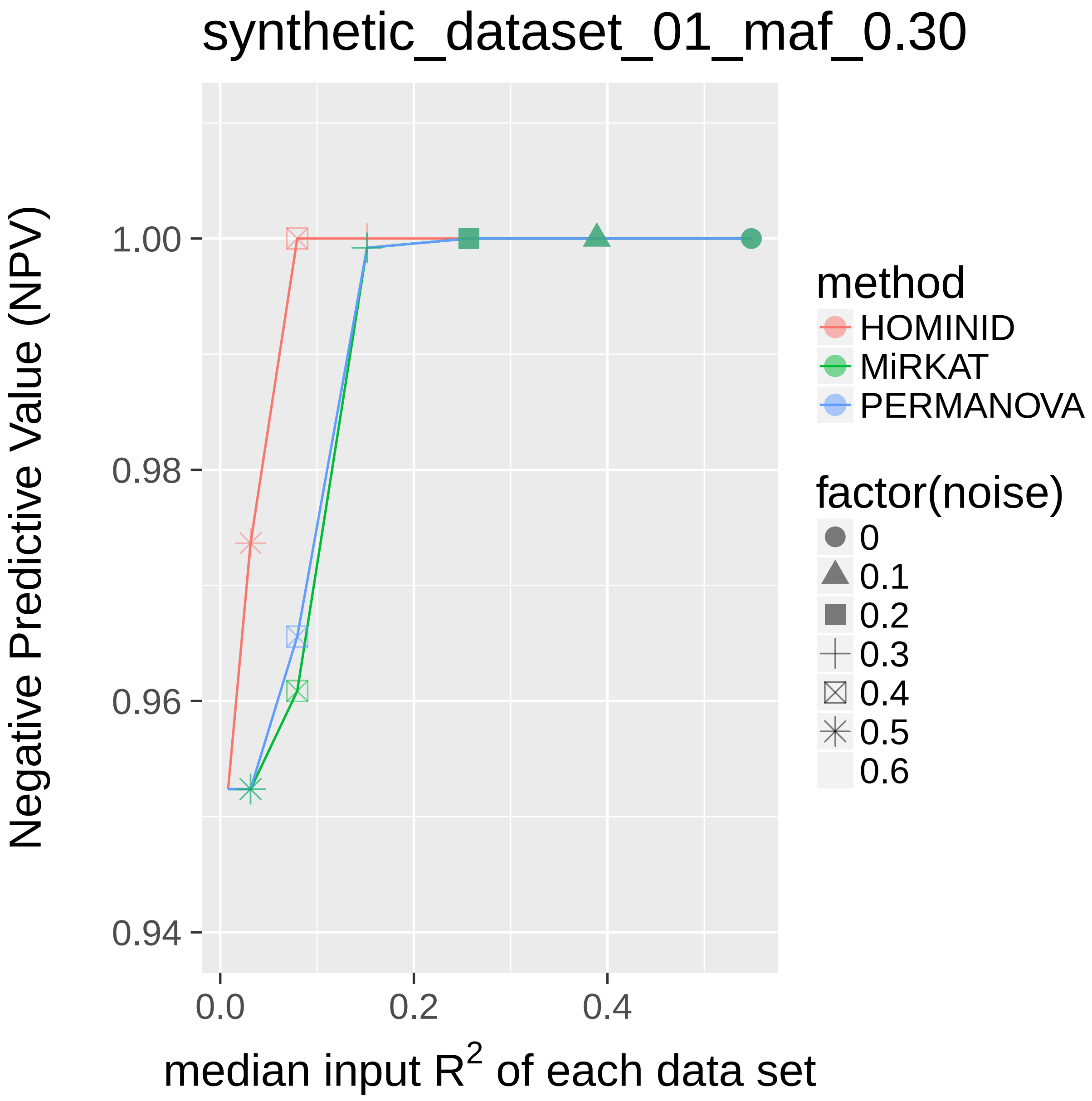

Figure S53: Comparision of NPV for HOMINID, PERMANOVA and MiRKAT.

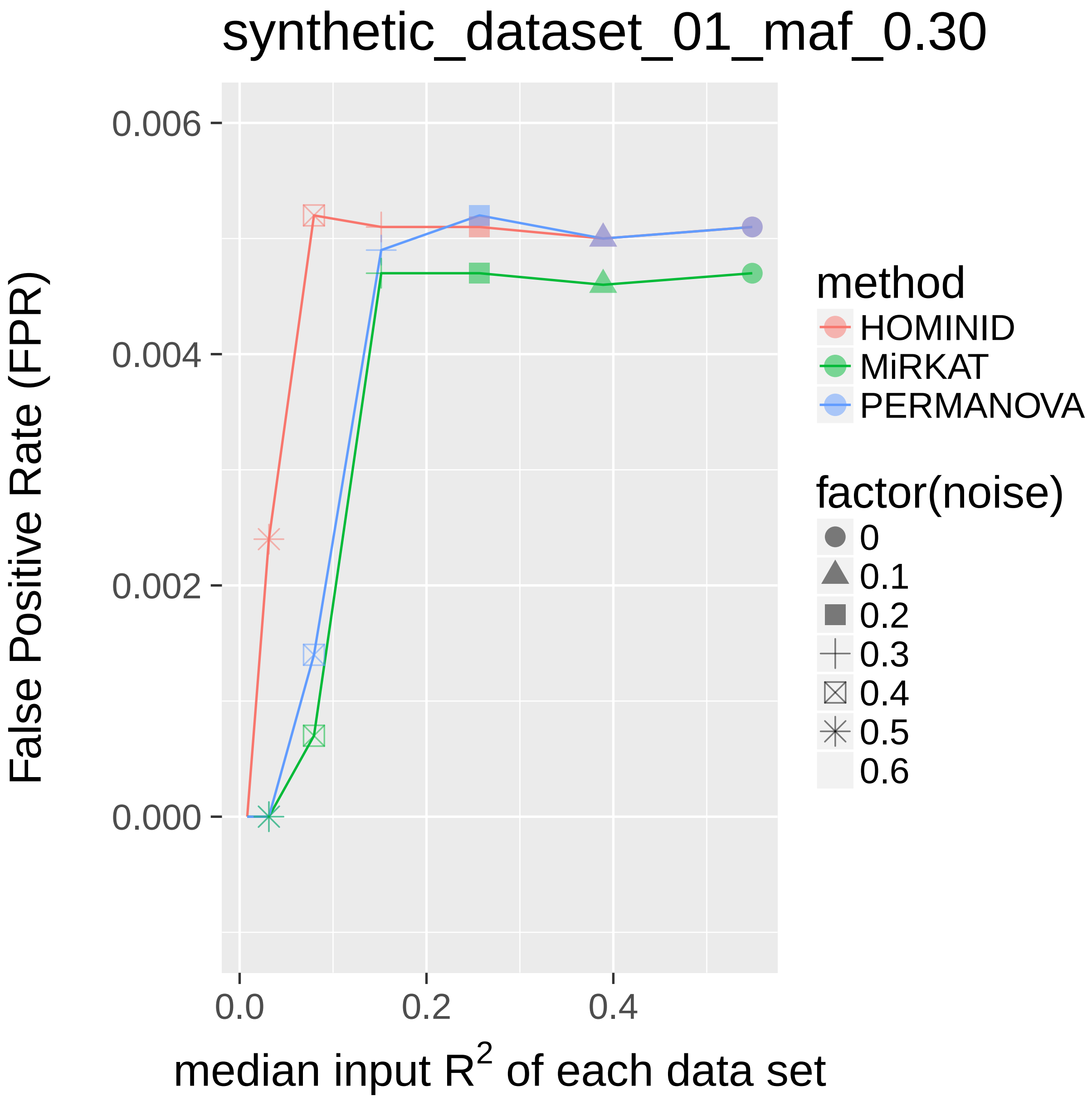

Figure S54: Comparision of FPR for HOMINID, PERMANOVA and MiRKAT.

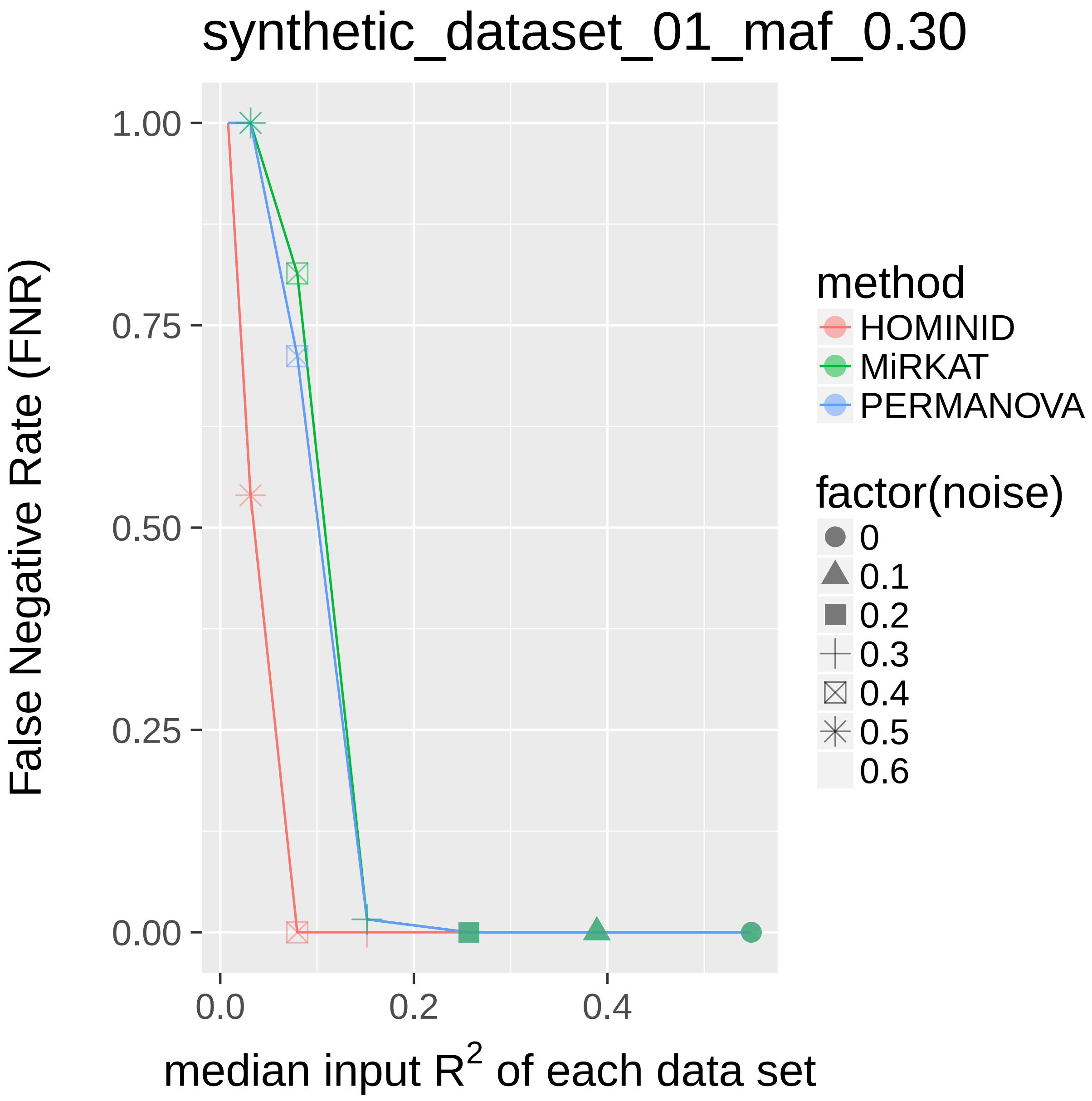

Figure S55: Comparision of FNR for HOMINID, PERMANOVA and MiRKAT.

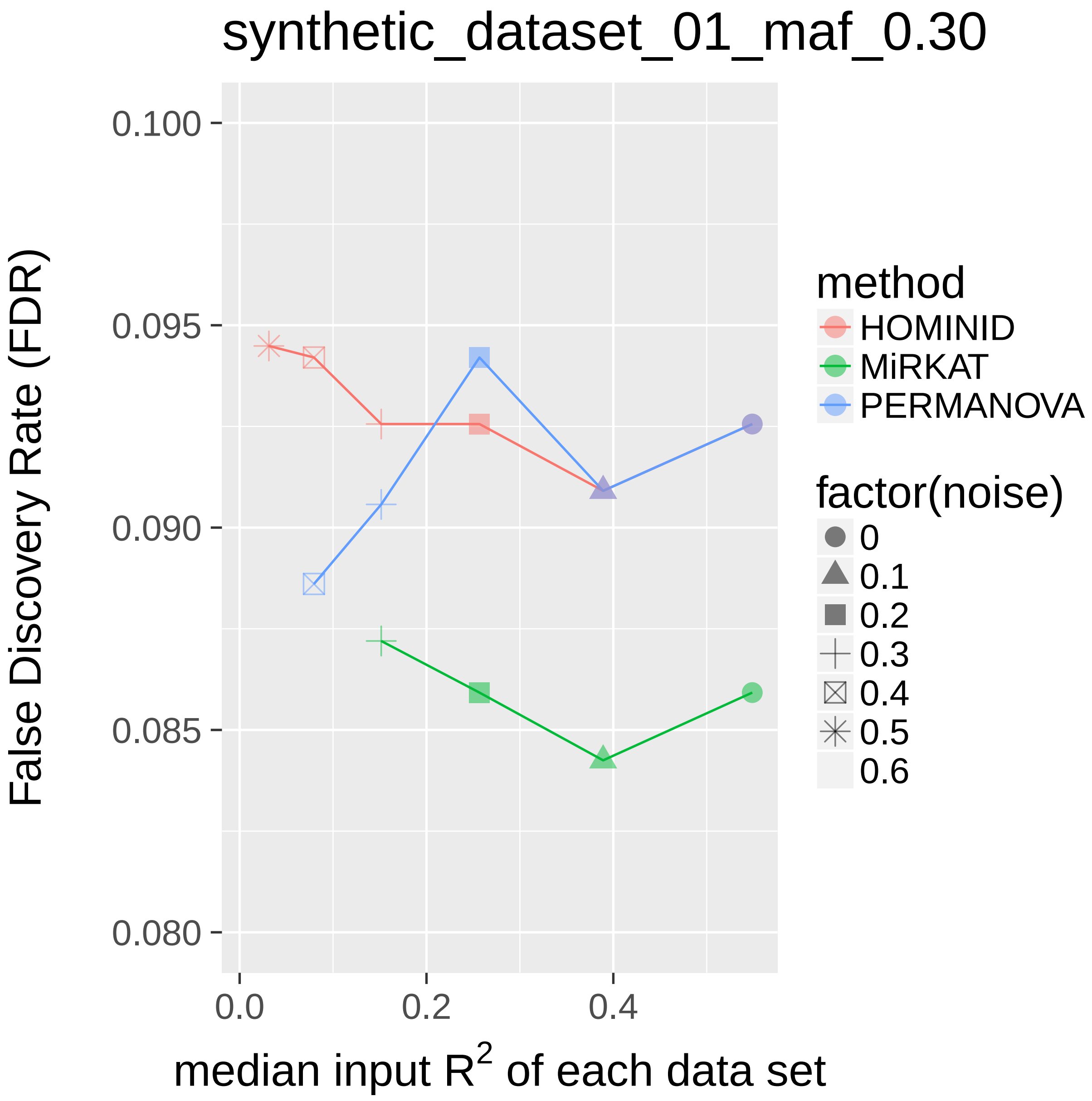

Figure S56: Comparision of FDR for HOMINID, PERMANOVA and MiRKAT.

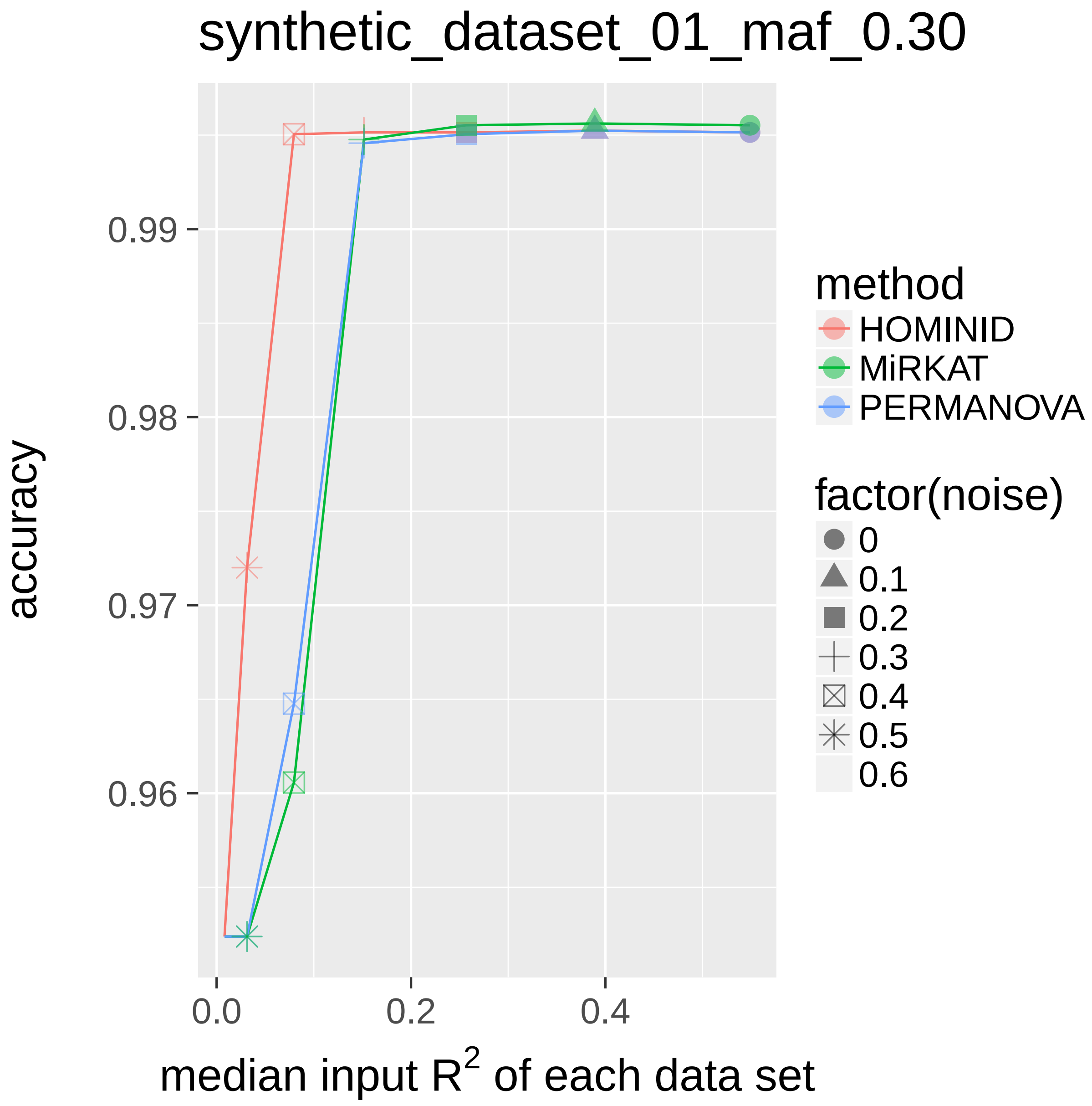

Figure S57: Comparision of accuracy for HOMINID, PERMANOVA and MiRKAT.

3 Additional HMP results

In the HOMINID analysis of HMP data, we focused our analysis on coding SNPs with MAF ≥ 0.2. The rationale for this cutoff is to make sure there is a sufficient number of individuals with each genotype. Specifically, HOMINID needs at least one individual who is homozygous for the minor allele in each of five cross-validation folds. The MAF cutoff that enables this will depend on the total number of individuals included in the analysis—the more individuals, the lower the MAF cutoff can be.

Supplemental Tables S1 and S2 contains the SNPs with $q \leq 0.1$, Lasso regression performed both with and without genetic PCs as covariates, respectively. We also include in both tables additional sheets containing the SNPs with the more generous cutoff of $q \leq 0.2$.

To show the results of the HMP data analysis compared to the permuted data, figure S58 is a Q-Q plot with the R_L^2 values of the permuted SNPs (SNPs uncorrelated to the microbiome) on the x-axis and the R_L^2 values for the real HMP data (unpermuted SNPs) on the y-axis. These data, comprising 12400 SNPs, are for Right antecubital fossa. The vast majority of the SNPs fall along the diagonal. Only the few SNPs with the highest R_L^2 values have larger R_L^2 values for the real data.

Lastly, in figure 4B of the main text, it's difficult to see the boxplot trends for low abundance taxa Alloscardovia (light blue) and Coriobacterieraceae (light green). We replotted the figure (figure S59), omitting the highest abundance taxon, Bacteroidetes (dark green), to better display the trends for the three lower abundance taxa.

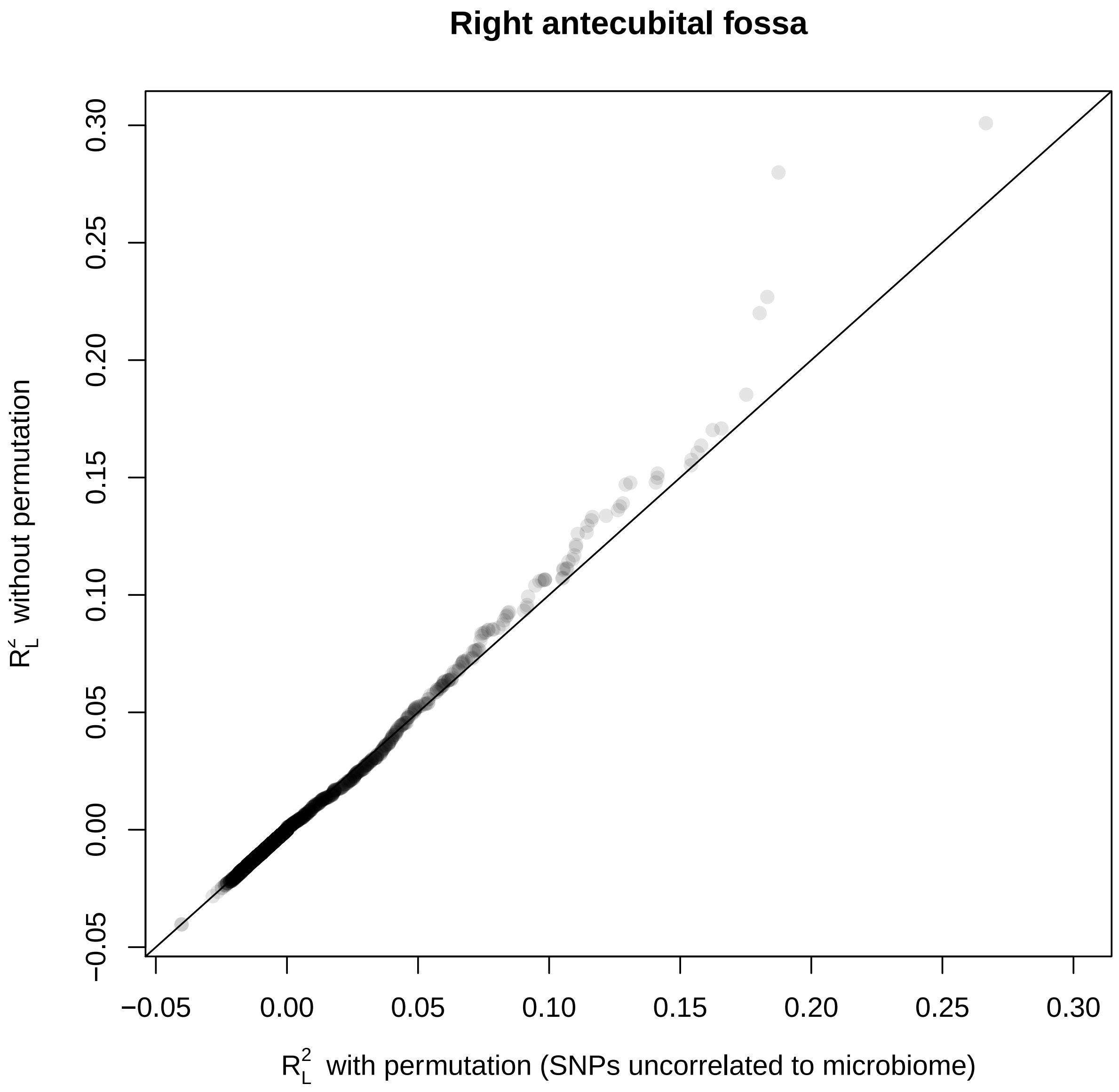

Figure S58: Q-Q plot of R_L^2 for permuted (x-axis) SNPs versus real (unpermuted) SNPs (y-axis).

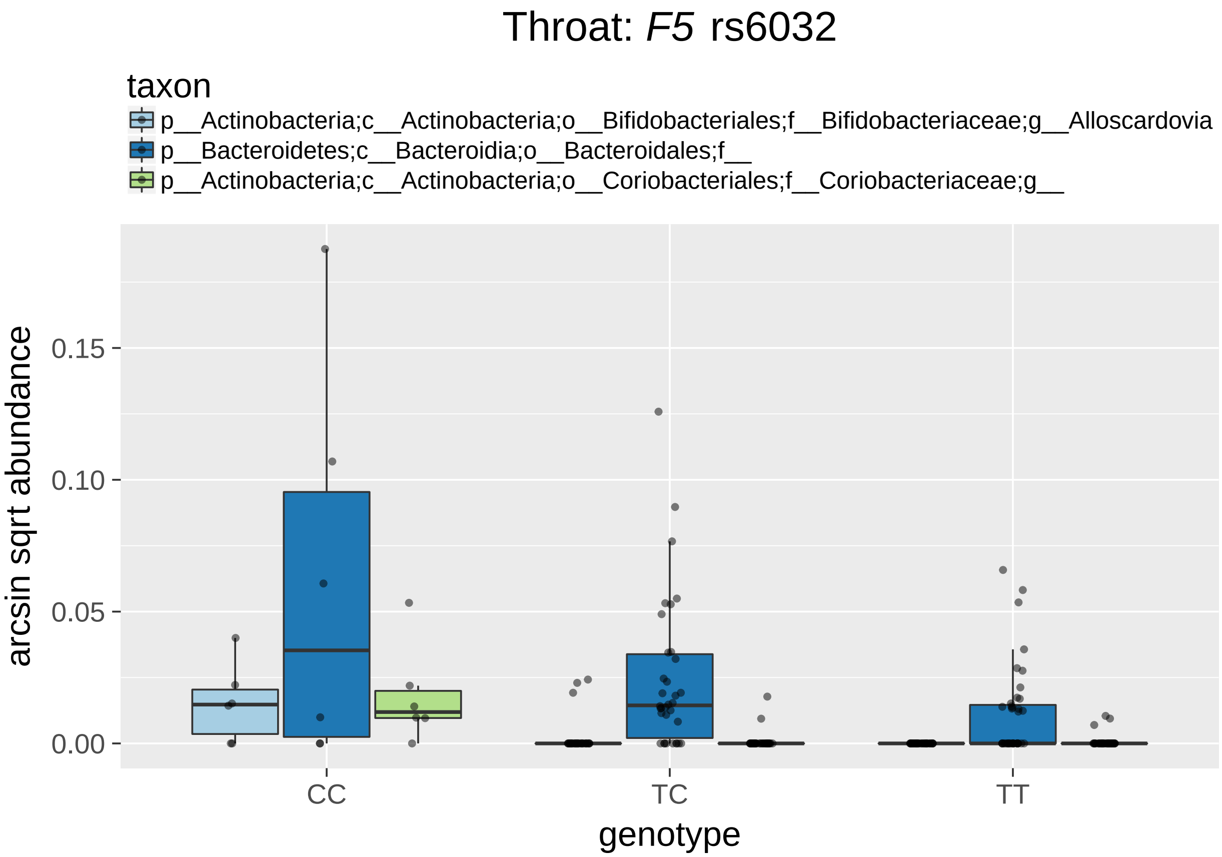

Figure S59: Same as main-text figure 4B, except taxon Bacteroidetes is omitted.

References

[1] Marti J. Anderson. A new method for non-parametric multivariate analysis of variance. *Austral. Ecology*, 26(1):32–46, 2001.

[2] Elita Baldridge, David J. Harris, Xiao Xiao, and Ethan P. White. An extensive comparison of species-abundance distribution models. *PeerJ*, 4:e2823, December 2016.

[3] Ran Blekhman et al. Host genetic variation impacts microbiome composition across human body sites. *Genome Biol.*, 16:191, 2015.

[4] J. Gregory Caporaso et al. QIIME allows analysis of high-throughput community sequencing data. *Nature Meth*, 7:335–336, 2010.

[5] Bradley Efron et al. Least angle regression. *Annals of Statistics*, 32:407–499, 2004.

[6] Trevor Hastie, Robert Tibshirani, and Jerome Friedman. *The Elements of Statistical Learning.* Springer-Verlag, 2nd edition, 2009.

[7] Human Microbiome Consortium. A framework for human microbiome research. *Nature*, 486:215, 2012.

[8] Human Microbiome Consortium. Structure, function and diversity of the healthy human microbiome. *Nature*, 486:207, 2012.

[9] Brian H. McArdle and Marti J. Anderson. Fitting multivariate models to community data: A comment on distance-based redundancy analysis. *Ecology*, 82(1):290–297, 2001.

[10] Nicolai Meinshausen and Peter Bühlmann. Stability selection. *J R Statist Soc B*, 72:417–473, 2010.

[11] Kevin P. Murphy. *Machine Learning: A Probabilistic Perspective.* Adaptive computation and machine learning series. MIT Press, 2012.

[12] Fabian Pedregosa et al. Scikit-learn: Machine learning in python. *J. Mach. Learn. Res.*, 12:2825–2830, 2011.

[13] Alkes L Price, Nick J Patterson, Robert M Plenge, Michael E Weinblatt, Nancy A Shadick, and David Reich. Principal components analysis corrects for stratification in genome-wide association studies. *Nat Genet*, 38:904–909, 2006.

[14] Jonathan K. Pritchard, Matthew Stephens, Noah A. Rosenberg, and Peter Donnelly. Association mapping in structured populations. *American Journal of Human Genetics*, 67:170–181, 2000.

[15] Robert Tibshirani. Regression shrinkage and selection via the Lasso. *J. R. Statist. Soc. B*, 1:267–288, 1996.

[16] Kai Wang, Mingyao Li, and Hakon Hakonarson. ANNOVAR: functional annotation of genetic variants from high-throughput sequencing data. *Nucleic Acids Research*, 38(16):e164, 2010.

[17] N. Zhao, J. Chen, I.M. Carroll, T. Ringel-Kulka, M.P. Epstein, H. Zhou, J.J. Zhou, Y. Ringel, H. Li, and M.C. Wu. Testing in microbiome-profiling studies with MiRKAT, the microbiome regression-based kernel association test. *Am. J. Hum. Genet.*, 96(5):797–807, 2015.
