# Supplementary material for: HOMINID: a framework for identifying associations between host genetic variation and microbiome composition
Source: Gigascience. 2017 Nov 8;6(12):1–7. doi: 10.1093/gigascience/gix107 (PMC5740987; doi:10.1093/gigascience/gix107)
Supplement: Response-to-Reviewer-Comments_Original-Submission.pdf [file gix107_response-to-reviewer-comments_original-submission.pdf]

Author's Response To Reviewer Comments

Response to Reviewers is appended to the Cover Letter, at the end of the PDF.
