# Supplementary material for: HOMINID: a framework for identifying associations between host genetic variation and microbiome composition
Source: Gigascience. 2017 Nov 8;6(12):1–7. doi: 10.1093/gigascience/gix107 (PMC5740987; doi:10.1093/gigascience/gix107)
Supplement: Response-to-Reviewer-Comments_Revision-2.pdf [file gix107_response-to-reviewer-comments_revision-2.pdf]

Author's Response To Reviewer Comments

We have reformatted the main text into a Technical Note, added a SciCrunch.org RRID, and are in corrrespondence with the GigaDB curator to host the HMP taxon data at GigaDB.
