# Supplementary material for: HOMINID: a framework for identifying associations between host genetic variation and microbiome composition
Source: Gigascience. 2017 Nov 8;6(12):1–7. doi: 10.1093/gigascience/gix107 (PMC5740987; doi:10.1093/gigascience/gix107)
Supplement: Reviewer-1-Report-(Original-Submission).pdf [file gix107_reviewer-1-report-(original-submission).pdf]

Reviewer Report

Title: HOMINID: A framework for identifying associations between host genetic variation and microbiome composition

Version: Original Submission **Date:** 11/22/2016

Reviewer name: Jun Chen

Reviewer Comments to Author:

In this manuscript, the authors presented a novel approach for identification of host genetic variants that are associated with microbiome composition based on a machine learning approach. An online tool for visualization of the associations has also been provided to facilitate the presentation and interpretation of the findings. The strength of their method is the achievement of both overall association tests and variable selection using lasso and stability selection. However, the major concern is the lack of rigor in statistics and weakness of the real data example. The current criteria for identifying "significant" SNPs without multiple testing correction might result in too many false positives and have an undesired impact on the field if it is widely adopted by the field. Overall, the procedure could be of potential interest to the community for future large-sample size studies if the authors stick to common statistical practice for high-dimensional data, use more realistic simulations, and compare to existing methods. I have the following specific comments:Major1. About the statistics.(1) The traditional p-value concerns one single hypothesis test. The current "p-value" based on permutation is misleading, and it is, in fact, an estimate of "false positive rate". The author should refrain from using the term "p-value" if it is different from the traditional meaning.(2) For high-dimensional data sets, the importance of control for false positives can never be overstated. Or the field will be full of false positives. That is the reason why the statistical genetics field usually uses the most conservative Bonferroni correction for GWAS. The author should make clear that the identification of significant SNPs should correct for multiple testing. At least, q-values should be used to control the false discovery rate if family-wise error rate control is not used due to low statistical power. Raw p-values should never be used. I also suggest the author implement the permutation-based family-wise error rate control.2. About the simulations.(1) Excessive zeros may be generated to mimics real microbiota data by using an overdispersed count model. The performance of linear models may be very sensitive to a large number of 0's or a large number of identical values after the proposed transformation. The data generated by the current exponential distribution could become very normal after arcsin sqrt transformation, which may not reflect the real data.(2) The simulated signal may be too strong. It is less likely that an SNP can have an R2 larger than 0.35. The human microbiome is highly variable and is strongly affected by numerous environmental factors such as diet. Based on existing epidemiological research, the R2 is usually very small (e.g. 2%). Thus much weaker correlation is expected. The sensitivity (a power of 0.95) is extraordinarily high.3. About the performance evaluation.(1) The evaluation of the performance should be based on type I error and power if the proposed method is a statistical test. Sensitivity and specificity may not be the best measure when there is multiple testing. A specificity of 98% will translate to a very low precision. Suppose we test 10,000 SNPs with 5% signals, a sensitivity of 30% and a false positive rate (1 -

specificity) of 2% will translate to ~200 false positives and 150 true positives. Thus the false discovery rate is more than 50%. In practice, the signal density and power may be much lower, and thus the majority may be false positives. Such high false positive rates are not acceptable, or the field will be full of false findings. The authors should tighten their criteria and use multiple testing correction procedure. The evaluation should be focused on the type I error control. For example, whether their method can control the false discovery rate at the nominal level using q-value approach. If not, cautions should be taken when applying the method.(2) The proposed method has not been compared to more straightforward methods such as PERMANOVA or MiRKAT test for each SNP locus, coupled by multiple testing correction. The power evaluation may not be very meaningful without comparing to some existing simple methods. The current simulation strategy may favor their method due to the model sparsity and unclustered signals. However, even in such scenarios, compared to PERMANOVA or MiRKAT with non-tree-based distance such as Bray-Curtis or Euclidean distance on their transformed abundance data will be very informative. I recommend the authors to compare to existing methods on both simulated data sets and real data sets.4. About the real data example.(1) The real data demonstration is very week. The authors used the raw "p-value" to select the "significant" SNP, which clearly results in many false positives. Possibly, the majority of the SNPs may be false positives. It is based on the observation that if their "p-value" (false positive rate) cutoff of 0.01 is used, there will be on average 1% false positives and approximately 32,696 * 0.01=327 "false" SNPs identified for each body loci. Looking at the number of identified SNPs for different body sites, it is similar (100 - 200 SNPs) to the number of expected false positives.(2) The number of genes that harbor SNPs that are correlated with the microbiome in more than one body site may also be a result of random noises. Since there are 15 body sites, there are 105 pairs. Assume that there is no real signal and that each body site identifies 1% significant genes randomly (using false positive rate of 0.01), there will be roughly1% * 1% * 105=1% genes show association with more than one body site. Suppose that we have 20,000 genes, and then it amounts to 200 genes, which is similar to the reported number.(3) The authors might consider permuting the genotypes and rerun the same procedure. If they can demonstrate the "NULL" model produces way less number of "significant" hits, the results will be more convincing.(4) The author may consider making the real data available so others can reproduce their findings and compare to their method in the future.Minor1. The author should state what q-value means and what type of error it controls. More detailed description of the procedure may be helpful. Some reference on the permutation-based false discovery control procedure may also be added.2. When using subsampling to calculate the median R2, the percentage of data used for test and training should be stated.3. Typo in "Supplementary data are available at Bioinformatics online."4. "Taxon count" may change to "number of taxa" for better clarity.5. Computation time should also be mentioned in the manuscript.

Level of Interest

Please indicate how interesting you found the manuscript: An article whose findings are important to those with closely related research interests

Quality of Written English

Please indicate the quality of language in the manuscript: Acceptable

Declaration of Competing Interests

Please complete a declaration of competing interests, considering the following questions:

- Have you in the past five years received reimbursements, fees, funding, or salary from an organisation that may in any way gain or lose financially from the publication of this manuscript, either now or in the future?
- Do you hold any stocks or shares in an organisation that may in any way gain or lose financially from the publication of this manuscript, either now or in the future?
- Do you hold or are you currently applying for any patents relating to the content of the manuscript?
- Have you received reimbursements, fees, funding, or salary from an organization that holds or has applied for patents relating to the content of the manuscript?
- Do you have any other financial competing interests?
- Do you have any non-financial competing interests in relation to this paper?

If you can answer no to all of the above, write 'I declare that I have no competing interests' below. If your reply is yes to any, please give details below.

I declare that I have no competing interests

I agree to the open peer review policy of the journal. I understand that my name will be included on my report to the authors and, if the manuscript is accepted for publication, my named report including any attachments I upload will be posted on the website along with the authors' responses. I agree for my report to be made available under an Open Access Creative Commons CC-BY license (http://creativecommons.org/licenses/by/4.0/). I understand that any comments which I do not wish to be included in my named report can be included as confidential comments to the editors, which will not be published.

I agree to the open peer review policy of the journal
