# Supplementary material for: HOMINID: a framework for identifying associations between host genetic variation and microbiome composition
Source: Gigascience. 2017 Nov 8;6(12):1–7. doi: 10.1093/gigascience/gix107 (PMC5740987; doi:10.1093/gigascience/gix107)
Supplement: Reviewer-1-Report-(Revision-1).pdf [file gix107_reviewer-1-report-(revision-1).pdf]

Reviewer Report

Title: HOMINID: A framework for identifying associations between host genetic variation and microbiome composition

Version: Revision 1 **Date:** 8/21/2017

Reviewer name: Jun Chen

Reviewer Comments to Author:

I really appreciate the authors' tremendous efforts to make their method more rigorous. They have addressed most of my concerns, and I am very pleased to see this new version. Certainly, the work will be a valuable contribution to the field. I only have very minor comments:

1. I could not find the plots of simulated R2 (truth) v.s. the estimated R2 (R^2_L). It 's nice to include a supplementary figure showing the relationship, which will give some idea about the potential bias;
2. The sample splitting for the calculation of R^2_L is still not very clear to me. I assume that the authors performed inner 5-fold CV on the training dataset (4/5 of the data) for tuning parameter selection and model building and used the remaining 1/ 5 of the data for R^2_L calculation (outer CV). The process was repeated by 100 times. If so, it is a nested cross-validation procedure.
3. I would suggest the author use 'alpha' instead of a fixed value such as '0.1' for the q-value cutoff. The users should decide the level of significance to use. I would also recommend the authors to set the default value of 'alpha' as '0.05' in their software.
4. For my previous comment, 'The authors might consider permuting the genotypes and rerun the same procedure. If they can demonstrate the "NULL" model produces way less number of "significant" hits, the results will be more convincing'. What I mean is to permute the sample labels of the HMP data (either SNP data or OTU data) to disrupt the SNP-Microbiome association and repeat the same process as you have done in the manuscript. This serves as a 'sanity check'. If the method is very robust, you should get much less or probably no hits.
5. In the results section, the authors wrote: "Currently, the website includes toy data representing all SNP-microbe associations with a nominal p-value <= 0.1 in the Human Microbiome Project data described above." However, in this new version, only q-value calculation was described. Did the author mean 'q-value'?

Methods

Are the methods appropriate to the aims of the study, are they well described, and are necessary controls included? Yes

Conclusions

Are the conclusions adequately supported by the data shown? Yes

Reporting Standards

Does the manuscript adhere to the journal's guidelines on [minimum standards of reporting?] Yes Choose an item.

Statistics

Are you able to assess all statistics in the manuscript, including the appropriateness of statistical tests used? Yes, and I have assessed the statistics in my report.

Quality of Written English

Please indicate the quality of language in the manuscript: Acceptable

Declaration of Competing Interests

Please complete a declaration of competing interests, considering the following questions:

- Have you in the past five years received reimbursements, fees, funding, or salary from an organisation that may in any way gain or lose financially from the publication of this manuscript, either now or in the future?
- Do you hold any stocks or shares in an organisation that may in any way gain or lose financially from the publication of this manuscript, either now or in the future?
- Do you hold or are you currently applying for any patents relating to the content of the manuscript?
- Have you received reimbursements, fees, funding, or salary from an organization that holds or has applied for patents relating to the content of the manuscript?
- Do you have any other financial competing interests?
- Do you have any non-financial competing interests in relation to this paper?

If you can answer no to all of the above, write 'I declare that I have no competing interests' below. If your reply is yes to any, please give details below.

I declare that I have no competing interests

I agree to the open peer review policy of the journal. I understand that my name will be included on my report to the authors and, if the manuscript is accepted for publication, my named report including any attachments I upload will be posted on the website along with the authors' responses. I agree for my report to be made available under an Open Access Creative Commons CC-BY license (http://creativecommons.org/licenses/by/4.0/). I understand that any comments which I do not wish to be included in my named report can be included as confidential comments to the editors, which will not be published.

I agree to the open peer review policy of the journal

To further support our reviewers, we have joined with Publons, where you can gain additional credit to further highlight your hard work (see: https://publons.com/journal/530/gigascience). On publication of this paper, your review will be automatically added to Publons, you can then choose whether or not to claim your Publons credit. I understand this statement.

Yes
