# Supplementary material for: HOMINID: a framework for identifying associations between host genetic variation and microbiome composition
Source: Gigascience. 2017 Nov 8;6(12):1–7. doi: 10.1093/gigascience/gix107 (PMC5740987; doi:10.1093/gigascience/gix107)
Supplement: Reviewer-2-Report-(Original-Submission).pdf [file gix107_reviewer-2-report-(original-submission).pdf]

Reviewer Report

Title: HOMINID: A framework for identifying associations between host genetic variation and microbiome composition

Version: Original Submission **Date:** 11/28/2016

Reviewer name: Katherine Pollard

Reviewer Comments to Author:

HOMINID: A framework for identifying associations between host genetic variation and microbiome compositionReviewers: Katherine Pollard and Nandita GarudSummary:HOMINID is a Lasso regression method for identifying human polymorphisms associated with the species' composition in the human microbiome. The authors applied the method to the Human Microbiome Project data to identify several candidate host SNPs associated with different species, some of which are already implicated with roles in diabetes and immunity. The authors generate synthetic data to test three different parameters that may impact the method's abilities: the allele frequency of the associated SNPs, the number of taxa associated with a SNP, and the total number of taxa present in a sample.Positives:With the extensive availability of microbiome data these days and the keen interest from the community to understand how human-associated microbiota impact human health, HOMINID is a valuable contribution to the community. Future work will certainly benefit from the framework laid out in this paper, especially if there are going to be extensions of this method for application to shotgun data, i.e. identifying associations between human SNPs and abundances of microbiome genes, and applications to other data sets. As it is in its current state, it is likely that HOMINID will be useful for scientists looking for associations of taxa with human SNPs without much further work. Finally, the online tool for searching and querying the results of this paper is easy to use, further enriching the resources available to the microbiome community.Major Critiques:1. The main suggestion we have is that the authors did not sufficiently justify the Lasso method through a comparison to other models. They mention some of the other methods in the literature in the introduction, but the simulations only show performance of their method. We recommend some benchmarking against other models or at least a discussion of why they chose this method.2. Population substructure: Blekhman et al. 2015 showed that the human polymorphisms most associated with microbiome composition were those that have the highest FST values across populations. How does HOMINID perform if there is population substructure? Does the number of false positives increase? A fourth simulation category exploring the effects of substructure would be helpful as most of the time human populations have complex demographic histories and hidden substructure and this may have an impact on results.3. Polygenic host effects on the microbiome: Many GWAS studies have the host SNPs as predictors rather than as response variables so that interactions (i.e. epistasis) between SNPs can be tested. It doesn't seem possible to test for epistasis in the author's framework with species as the predictors, yet it seems to be a very plausible that there would be epistatic effects. Could the authors comment on this limitation of their method in the main text as well explain as how testing for epistasis along with multiple species might further impact the multiple hypothesis-testing problem? Could the authors propose some suggestions on how one might

address this?4. Synthetic dataset improvements:* Data set 1: We recommend increasing the range of MAFs tested. It would be good to see a drop off in performance for Figures S4 and S5 (if there is a dropoff). We expect there to be lower performance for lower MAFs.* What is the impact of relative abundances of associated species? Do very rare species have less predictive power? It would be helpful to have another synthetic data set to test this as it is very possible that the rare species in the microbiome have important impacts on human health.Minor Critiques:1) Make it clearer that the predictor is the species rather than human SNP in the main text- usually it is the other way around2) In the methods and the main text, could the authors make clearer that the predictors are species abundances rather than presence/absence (if true)?3) In the main text, page 5: would be worth identifying which species are associated with the significant SNPs identified in ABCC8, the HLA genes, and ATL2.4) Introduction, page 3, line 21: Goodrich et al. calculated heritability for Christensenellaceae to be <0.4, so, it is heritable but not highly heritable.5) What was the rationale for testing for associations in different body sites separately? Couldn't the same host SNPs impact species composition in different body sites?

Level of Interest

Please indicate how interesting you found the manuscript: An article of importance in its field

Quality of Written English

Please indicate the quality of language in the manuscript: Acceptable

Declaration of Competing Interests

Please complete a declaration of competing interests, considering the following questions:

- Have you in the past five years received reimbursements, fees, funding, or salary from an organisation that may in any way gain or lose financially from the publication of this manuscript, either now or in the future?
- Do you hold any stocks or shares in an organisation that may in any way gain or lose financially from the publication of this manuscript, either now or in the future?
- Do you hold or are you currently applying for any patents relating to the content of the manuscript?
- Have you received reimbursements, fees, funding, or salary from an organization that holds or has applied for patents relating to the content of the manuscript?
- Do you have any other financial competing interests?
- Do you have any non-financial competing interests in relation to this paper?

If you can answer no to all of the above, write 'I declare that I have no competing interests' below. If your reply is yes to any, please give details below.

I declare that I have no competing interests

I agree to the open peer review policy of the journal. I understand that my name will be included on my report to the authors and, if the manuscript is accepted for publication, my named report including any attachments I upload will be posted on the website along with the authors' responses. I agree for my report to be made available under an Open Access Creative Commons CC-BY license (http://creativecommons.org/licenses/by/4.0/). I understand that any comments which I do not wish to be included in my named report can be included as confidential comments to the editors, which will not be published.

I agree to the open peer review policy of the journal
