# Supplementary material for: HOMINID: a framework for identifying associations between host genetic variation and microbiome composition
Source: Gigascience. 2017 Nov 8;6(12):1–7. doi: 10.1093/gigascience/gix107 (PMC5740987; doi:10.1093/gigascience/gix107)
Supplement: Reviewer-2-Report-(Revision-1).pdf [file gix107_reviewer-2-report-(revision-1).pdf]

Reviewer Report

Title: HOMINID: A framework for identifying associations between host genetic variation and microbiome composition

Version: Revision 1 **Date:** 8/18/2017

Reviewer name: Katherine Pollard

Reviewer Comments to Author:

Reviewers: Katherine Pollard and Nandita Garud

Summary:
The authors propose a new method called HOMINID to identify host SNPs that are associated with taxonomic abundances in the human microbiome. This paper is a resubmission in which the authors made some key modifications including (1) comparison to two existing methods, and (2) and expansion of the range of parameters used for the simulated data set to test the ability of their method. The manuscript is much improved, though we have a few further suggestions that we recommend addressing before acceptance for publication.

Minor Suggestions:

In the introduction, be explicit that taxon abundance (versus presence or some other characteristic) is the quantity that is tested for associations with SNPs or for heritability.

In the methods, "noise" is defined as "swapping the genotypes of pairs of samples, reducing the correlation between the N_ctc correlated taxa and the host SNP genotypes." In Figure 2, "noise" varies from 0.1 to 0.6. However, we cannot figure out what these numbers correspond to.

In the methods, explain what "ctc" means in N_ctc. This appears to be the number of taxa that are associated with a SNP, but the definition could be clearer.

It appears that SNPs in the synthetic data set are uncorrelated. Is the idea that these are tag SNPs per LD block? Clarify if they are indeed simulated independently and why.

We recommend discussing the assumptions for using the q-value multiple testing correction and why it may be a good choice in the presence of correlation between tests (i.e., SNPs with high LD).

Bottom of p.5 - In which analysis (synthetic or HMP) are SNPs associated with sex and why?

In Figure 2, it is difficult to tell whether there is any trend with MAF, R^2, and FDR/Sensitivity. In the text, you state that there is no trend. Perhaps this figure would be more easily interpretable if each

boxplot was a point representing the mean of each category, rather than a distribution (in the legend, please state how many points went into each boxplot distribution). Alternatively, different simulations could be plotted separately. As presented, the overlapping boxplots are difficult to interpret, especially in the second (MAF) column.

In Figure 2, it seems that MAF has little effect on the results. But later you threshold SNPs in the human microbiome project analysis to have a MAF of at least 0.2. What was the rationale for this?

The reference for Tong, Maomeng et al. 2014 has a typo: Tong, Maomeng shows up twice.

In Figure 4B, it is hard to see the boxplot trends for Alloscardocia and Coriobacterieraceae. Plotting on a log scale may help show the trend better for these bacteria.

Methods

Are the methods appropriate to the aims of the study, are they well described, and are necessary controls included? Yes

Conclusions

Are the conclusions adequately supported by the data shown? Yes

Reporting Standards

Does the manuscript adhere to the journal's guidelines on minimum standards of reporting? Yes Choose an item.

Statistics

Are you able to assess all statistics in the manuscript, including the appropriateness of statistical tests used? Yes, and I have assessed the statistics in my report.

Quality of Written English

Please indicate the quality of language in the manuscript: Acceptable

Declaration of Competing Interests

Please complete a declaration of competing interests, considering the following questions:

- Have you in the past five years received reimbursements, fees, funding, or salary from an organisation that may in any way gain or lose financially from the publication of this manuscript, either now or in the future?

- Do you hold any stocks or shares in an organisation that may in any way gain or lose financially from the publication of this manuscript, either now or in the future?
- Do you hold or are you currently applying for any patents relating to the content of the manuscript?
- Have you received reimbursements, fees, funding, or salary from an organization that holds or has applied for patents relating to the content of the manuscript?
- Do you have any other financial competing interests?
- Do you have any non-financial competing interests in relation to this paper?

If you can answer no to all of the above, write 'I declare that I have no competing interests' below. If your reply is yes to any, please give details below.

I declare that I have no competing interests

I agree to the open peer review policy of the journal. I understand that my name will be included on my report to the authors and, if the manuscript is accepted for publication, my named report including any attachments I upload will be posted on the website along with the authors' responses. I agree for my report to be made available under an Open Access Creative Commons CC-BY license (http://creativecommons.org/licenses/by/4.0/). I understand that any comments which I do not wish to be included in my named report can be included as confidential comments to the editors, which will not be published.

I agree to the open peer review policy of the journal

To further support our reviewers, we have joined with Publons, where you can gain additional credit to further highlight your hard work (see: https://publons.com/journal/530/gigascience). On publication of this paper, your review will be automatically added to Publons, you can then choose whether or not to claim your Publons credit. I understand this statement.

Yes
